# Chalcone Synthesis by Green Claisen–Schmidt Reaction in Cationic and Nonionic Micellar Media

**DOI:** 10.1021/acs.joc.4c02616

**Published:** 2025-02-18

**Authors:** Davide Dotta, Matteo Gastaldi, Andrea Fin, Nadia Barbero, Claudia Barolo, Francesca Cardano, Federica Rossi, Francesca Brunelli, Guido Viscardi, Gian Cesare Tron, Pierluigi Quagliotto

**Affiliations:** † Dipartimento di Chimica, Università di Torino, via P. Giuria 7, Torino 10125, Italy; ‡ NIS Interdepartmental Centre and INSTM Reference Centre, University of Torino, Via Gioacchino Quarello 15/a, Torino 10125, Italy; § Istituto di Scienza, Tecnologia e Sostenibilità per lo sviluppo dei Materiali Ceramici (ISSMC−CNR), Via Granarolo 64, RA, Faenza 48018, Italy; ∥ Dipartimento di Scienza e Tecnologia del Farmaco, Università di Torino, via P. Giuria 11, Torino 10125, Italy; ⊥ Dipartimento di Scienze del Farmaco, Università del Piemonte Orientale, Largo Donegani 2, Novara 28100, Italy

## Abstract

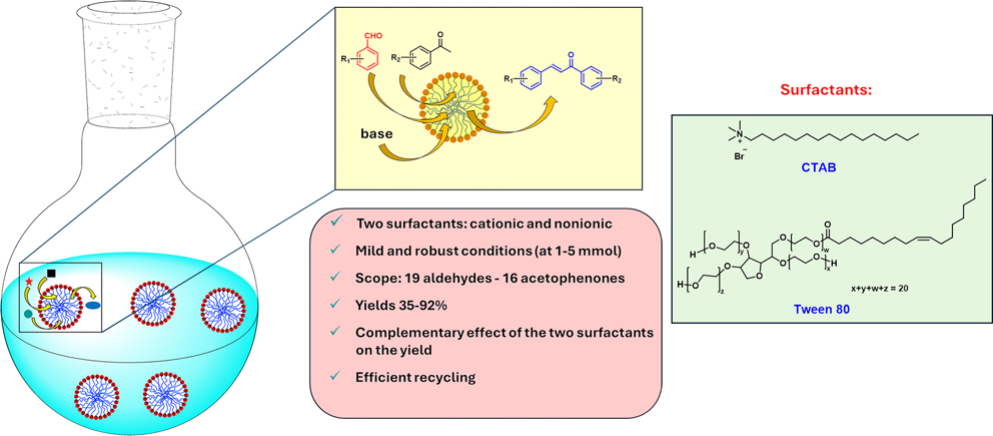

In this paper, micellar-mediated synthesis of chalcones was explored. After optimization of the reaction conditions, the cationic surfactant CTAB and the nonionic one, Tween 80, were taken into consideration. Both surfactants were used to study the scope of Claisen–Schmidt reactants, and a wide scope on both aromatic aldehydes and methyl ketones was explored, obtaining from good to very good yields in most cases and thus demonstrating that the chalcones can be proficiently synthesized in micellar solutions with a wide functional group tolerability. Often, when one surfactant did not perform well, the other surfactant performed better, demonstrating that the use of different surfactants can constitute a good alternative to overcome reactivity problems. Besides, Tween 80 can be proposed as a good and greener alternative to CTAB in most cases. Some reactions gave low yields, showing that some specific improvements would be needed to address the low reactivity. The micellar medium was studied by NMR to search for information about the association of the Claisen–Schmidt reactants with the micelles and their locations within them. Diffusion Ordered Spectroscopy (DOSY) was applied to assess the interaction and the percentage of incorporation of reactants into the micelles.

## Introduction

The urge to develop organic reactions in a greener and more sustainable way is a topic of current research and industrial application. The transition toward more sustainable processes is crucial also for the chemical industry and it is not deferrable; thus, a new way to conceive reactions is needed. Among the possible greener alternatives, one chance to go green is to eliminate the organic solvent, since in practice it is the most abundant substance used in a reaction and the largest source of pollution, danger, and waste production. Solvent-free grinding^1−3^ or microwaves were sometimes successful.^4,5^ Most reactions, however, still need to be performed in a solvent medium, and water is a valid alternative solvent since it is safe, not dangerous, and is normally not taken into account in the green metrics.^6^ While water cannot dissolve most of the organic compounds, peculiar effects on organic reactions were already evidenced by Breslow et al.^7^ The addition of a surfactant and the relative self-organizing properties in micelles can be a solution to this flaw. Surfactants and their micelles have been known to catalyze organic reactions since the 50s–60s,^8^ but their application in preparative organic chemistry was explored only after 2000.^9^ In 2008, Lipshutz and coworkers approached this topic from a new point of view,^10^ also preparing the so-called “designer surfactants”, tailor-made compounds for preparative organic chemistry.^10−17^ Since then, micellar organic reactions have become an emerging topic for the future of organic chemistry.^15,18^ A lot of important reactions were brought efficiently in micellar medium with interesting results:^18^ (i) Pd-catalyzed reactions;^15,19,20^ (ii) peptide coupling;^17,21−23^ (iii) reactions involving dehydration steps.^15,24,25^

Chalcones are among the most synthesized and studied compounds, showing applications in many fields, such as drugs,^26,27^ hole-transporting materials (HTMs),^28^ dyes for solar cells (DSSC),^29−32^ polymer precursors for organic photovoltaics (OPV),^33^ nonlinear optical (NLO) materials,^34−36^ organic light-emitting diodes (OLED),^37^ materials for organic electronic applications,^38^ and fluorophores.^39^ Chalcones are also the starting material for the preparation of several 5- and 6-membered heterocycles.^40^ These premises encouraged us to make the chalcone synthesis greener by performing the Claisen–Schmidt reaction in a micellar medium.

The main literature methods for chalcone synthesis are the Claisen–Schmidt reaction (the most common method), cross-coupling, the Friedel–Crafts reaction, and the photo-Fries rearrangement.^26,41^ Some green chemistry conditions, such as solvent-free grinding^2,3^ or microwaves were tried.^5^ The Claisen–Schmidt method is performed in an organic solvent, mostly an alcohol such as methanol or ethanol (considered a “green solvent”)^42−45^ using different bases such as NaOH,^26,42,43,45,46^ KOH,^26,47−49^ and Ca(OH)_2_.^50^ Sometimes, acid activation is used with HCl,^51^ p-toluenesulfonic acid,^52^ and Lewis acids like AlCl_3_^51^ and BF_3_.^53,54^ While the reaction is performed at high concentrations, thus limiting the use of solvents,^44^ the flammability and toxicity of the organic solvent remain an urgent problem. Often the chalcone precipitates and it can be separated by simple filtration, limiting the workup.

The aldol reaction has already been studied under micellar conditions,^55^ including for the chalcones.^56−58^ The surfactants used in the literature are quaternary ammonium surfactants (QAS), such as cetyltrimethylammonium bromide (CTAB), which, when studied at just the critical micellar concentration (CMC) and higher concentrations, have been shown to be the best-performing surfactants.^59−61^ However, an extended study of this topic is still lacking.

We planned a systematic study on chalcone synthesis by the micellar Claisen–Schmidt reaction with the aim to (i) elucidate the effects of reactants and surfactant structure, (ii) improve the yield and purity of the final product, (iii) control side reactions, and possibly (iv) save energy. We first studied the reaction in cetyltrimethylammonium bromide (CTAB) even though some concerns about the toxicity of quaternary ammonium surfactants were raised, due to their bacteriostatic effect^62^ and limited biodegradation.^63,64^ Therefore, we extended the study to a series of nonionic surfactants, known to be less harmful and often more biodegradable, choosing Tween 80 as a better alternative.

## Results and Discussion

**Scheme 1:**
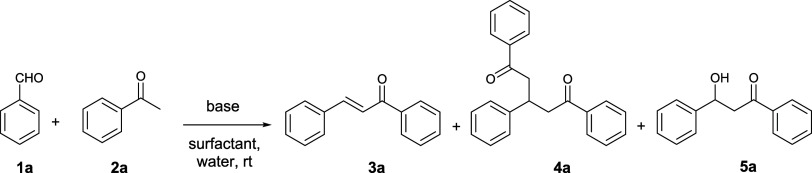
Claisen–Schmidt Optimization Reaction

The synthesis of simple chalcone **3a** by the Claisen–Schmidt method (Scheme 1) was studied to optimize reaction conditions. Reaction time was standardized at 24 h to evidence differences in the behavior of bases and surfactants during the screening.

CTAB was considered since it was the most reported one in the literature for the aldol reaction.^56,58−61,65−68^ Vessel dimensions and the stirring rate can have a significant influence on the reaction. An 8 mL vial was used, and the reaction was stirred at 1000 rpm.^14,69−71^ The 1 mmol scale and 1 mmol/mL (1M) concentration were adopted for benzaldehyde (1 equiv) and acetophenone (1 equiv) following the suggestion of Lipshutz et al.^15,18^ who reported working at a 0.5–1.0 mmol/mL range of reactant concentrations. Apart from a few cases (0.1^58^ and 0.2 mmol/mL),^57^ 0.5 mmol/mL was the concentration most often used for chalcone synthesis.^59−61,65,66,68^

### Screening of Bases

We started by comparing the use of a water CTAB solution with pure EtOH and water alone, taken as reference reactions, and optimizing the CTAB concentration. NaOH was chosen as a base for these first trials (reported in Table 1, entries 1–5) since it was the most used one in the literature.^26,42,43,45,46,50^

**Table 1:** Optimization of Conditions for the Chalcone Synthesis (1 mmol scale)^a^

			Yield (%)^b^	
Entry	Solvent/Surfactant	Base	**3a**	**4a**
1^44^	EtOH	NaOH	59	8
2	H_2_O	NaOH	70	0
3	CTAB^c^	NaOH	56	11
4	CTAB^d^	NaOH	65	9
5	CTAB^e^	NaOH	61	8
6	CTAB^d^	KOH	62	9
7	CTAB^d^	K_2_CO_3_	64	10
8	CTAB^d^	KOtBu	64	9
9	CTAB^d^	DBU^f^	34	15
10	CTAB^d^	DABCO^g^	49	4
11	CTAB^d^	Piperidine	44	3
12	CTAB^d^	TEA^h^	57	5
13	CTAB^d^	C_12_H_25_(CH_3_)_2_N	-	-

a Reaction conditions: 5 mL vial, benzaldehyde (1 mmol), acetophenone (1 mmol), base (1 mmol); CTAB solution (1 mL), rt, 24 h.

b The yields were estimated by ^1^H NMR with heptane as internal standard.

c (at CMC; 0.9 mM, 0.033%).

d (5 × CMC; 0.0045 M, 0.16%).

e (61 × CMC; 54.9 mM, 2%).

f 1,8-Diazabicyclo[5.4.0]undec-7-ene (DBU).

g 1,4-Diazabicyclo[2.2.2]octane (DABCO).

h Triethylamine (TEA).

Reference reaction in EtOH gave a 59% yield (entry 1), in agreement with the literature.^50^ Surprisingly, EtOH (entry 1) also promoted the formation of the Michael adduct **4a**, as a side reaction of the chalcone with the acetophenone enolate. Water gave a yield of 70% (entry 2), in a rough emulsion condition with no formation of Michael adduct **4a**.

According to recently published micellar reactions,^15,18^ three different CTAB concentrations were tested to check for the surfactant concentration effect. The yields in chalcone **3a** showed a bell-shaped trend: (i) at CMC (entry 3, yield 56%), (ii) at 5 times above the CMC (entry 4, yield 65%), (iii) at 2%, i.e., 61 times above the CMC (entry 5, yield 61%). This trend is a known behavior, where the kinetic constant *vs* the surfactant concentration plot is defined as a bell-shaped curve showing a maximum.^8,72,73^ The formation of Michael adduct **4a** was observed with comparable yields in all three experiments. In the literature, the Michael reaction was reported to occur in CTAB, in a 48-h one-pot reaction.^58^ Considering the formation of Michael adduct **4a** in EtOH too, this reactivity probably comes from the solubilization and compartmentalization of the chalcone inside the micelles, while the positive charges at the micellar surface attract the enolate anions, thus enabling the fast side reaction.

Inorganic bases such as NaOH (entry 3), KOH (entry 6), and K_2_CO_3_ (entry 7) were compared while working at a CTAB concentration of 5 times its CMC (0.16%), identified as the optimal value by previous experiments. Similar results (Table 1) for those bases were observed. The α-arylation of heteroarylketones^70^ performed in TPGS-750-M aqueous solution suggested the use of the water-sensitive KOtBu as a base (entry 8). We observed a yield (64%) comparable to that observed using KOH (entry 6, 62%). Probably, in the previously reported case,^70^ the use of an excess of KOtBu allowed it to survive partially to hydrolysis and to react within the micelles. On the contrary, in our experiment (entry 8), the equimolar KOtBu has been fully hydrolyzed to KOH and *t*-BuOH.

The comparable inorganic base effect and significant acetophenone solubility in water (around 5.5–6.3 g/L) suggest that the enolization occurs at the micellar surface, where the hydroxide anions interact with the positively charged headgroups of CTAB.^74,75^

Organic bases were also employed, such as DBU (entry 9), DABCO (entry 10), piperidine (entry 11), triethylamine (entry 12), and *N,N*-dimethyldodecylamine (entry 13), giving sluggish reactions and lower yields (34–57%) than those obtained with the inorganic bases. TEA worked better than the other organic bases in the trial, giving a 57% yield. Quite surprisingly, the *N,N*-dimethyldodecylamine did not react at all, while its hydrophobic character caused it to associate with the micelles. Even for organic bases, the Michael adduct was always produced, in a yield of 3% to 15% depending on the different bases, suggesting that the formation of the Michael adduct is peculiar for the surfactant medium. The aldol **5** was never detected.

Since the reaction also occurs just at the CMC, the simple hydrophobic effect can help to better dissolve the reactants by anticipating the CMC (see a more extended discussion in the Supporting Information),^76−78^ and the specific charge interaction between the cationic headgroups with both benzaldehyde and the enolate can keep the two reactive counterparts close enough together, to react efficiently.^58^ These data suggest that the optimal, greener, and cost-effective one is NaOH.

### Optimization of the Surfactant

Nonionic surfactants were investigated at a 2% concentration, usually the most reported in the literature (Table 2).^15^ First, we tried the surfactants developed by Lipshutz and coworkers (Figure S1): (i) TPGS-750-M (entry 3), (ii) PTS (entry 4), and (iii) Nok (entry 5).^15^ They performed in a nearly equivalent way and were similar to commercially available surfactants Brij 35^70^ (entry 6), Triton X-100^70,79^ (entry 7), and Kolliphor EL^79^ (entry 8), while Tween 80^80^ (entry 9) was the best one (85% yield).

**Table 2:** Optimization of the Surfactant for Chalcone Synthesis (1 mmol scale) with NaOH as the Base^a^

		Yield (%)^b^	
Entry	Surfactant (2%)	**3a**	**4a**
1	CTAB	61	8
2	SDS	83	0
3	TPGS	77	0
4	PTS	78	0
5	Nok	75	0
6	Brij 35	80	1
7	Triton X-100	76	0
8	Kolliphor EL	76	0
9	Tween 80	85	0

a Reaction conditions: 5 mL vial, benzaldehyde (1 mmol), acetophenone (1 mmol), NaOH (base) (1 mmol); surfactant solution (1 mL), rt, 24 h.

b The yields were estimated by ^1^H NMR with heptane as an internal standard.

The sodium dodecyl sulfate (SDS, entry 2) was compared to CTAB too. It also gave a very good yield (83%), which was quite unexpected for an anionic surfactant that should repel OH^–^ anions at its micellar surface. Unlike CTAB, all the nonionic surfactants and SDS did not promote the Michael reaction. At the end of the reaction optimization, we can remark on the improvement in sustainability, since the reaction performed in EtOH shows an E-factor of 15.7, while in CTAB and Tween 80 it is 8.6 and 6.6, respectively.

Due to these findings, the reaction scope was performed with NaOH and 2% CTAB or Tween 80.

### Scope of the Aldehydes

**Scheme 2:**
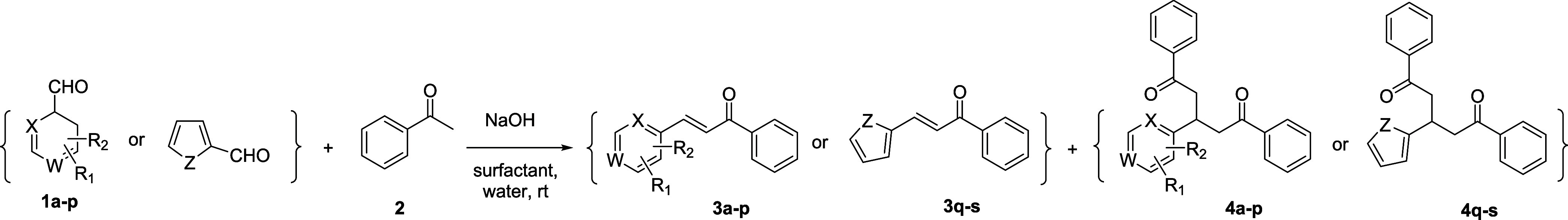
Scope of the Aldehydes

The scope of the aldehydes (see Scheme 2 and Table 3 for both CTAB and Tween 80) demonstrated that most of the functional groups are well tolerated, with the only exception being the ester group (entry 20, compound **3n**) which was hydrolyzed and yielded just 28% for CTAB and 11% for Tween 80, while the acetamido group (entry 2, compound **3b**) was hydrolyzed in minimal quantity.

**Table 3:** Scope of Aldehydes (1 mmol scale) in CTAB 2% and Tween 80 2% for 24 h^a^

						Yield(%)^b^							
Entry	Aldehyde	X	W	Z	T(°C)	CTAB				Tween 80			
1	H	CH	CH	-	25	**3a**	65	**4a**	10	**3a**	84	**4a**	0
2	4-AcNH	CH	CH	-	25	**3b**	79	**4b**	0	**3b**	85	**4b**	0
3	4-CH_3_	CH	CH	-	25	**3c**	80	**4c**	8	**3c**	78	**4c**	0
4	4-OCH_3_	CH	CH	-	25	**3d**	82	**4d**	9	**3d**	34	**4d**	0
5	4-OCH_3_	CH	CH	-	45	**3d**	-	**4d**	-	**3d**	66	**4d**	0
6	2-OCH_3_	CH	CH	-	25	**3e**	59	**4e**	0	**3e**	40	**4e**	0
7	2,6-diOCH_3_	CH	CH	-	25	**3f**	90	**4f**	0	**3f**	59	**4f**	0
8	2,6-diOCH_3_	CH	CH	-	45	**3f**	-	**4f**	-	**3f**	93	**4f**	0
9	4-OH^c^	CH	CH	-	25	**3g**	37	**4g**	0	**3g**	27	**4g**	0
10	4-OH^c^	CH	CH	-	45	**3g**	39	**4g**	0	**3g**	21	**4g**	0
11	4-Br	CH	CH	-	25	**3h**	82	**4h**	0	**3h**	30	**4h**	0
12	4-Br	CH	CH	-	45	**3h**	-	**4h**	-	**3h**	90	**4h**	0
14	3-Br	CH	CH	-	25	**3i**	90	**4i**	0	**3i**	76	**4i**	0
15	2-Br	CH	CH	-	25	**3j**	55	**4j**	12	**3j**	68	**4j**	0
16	2-Br	CH	CH	-	45	**3j**	53	**4j**	19	**3j**	84	**4j**	0
16	4-NO_2_	CH	CH	-	25	**3k**	69	**4k**	12	**3k**	87	**4k**	0
17	4-NO_2_	CH	CH	-	45	**3k**	73	**4k**	15	**3k**	-	**4k**	-
18	4-CN	CH	CH	-	25	**3l**	66	**4m**	0	**3m**	98	**4m**	0
19	4-COOH	CH	CH	-	25	**3m**	70	**4l**	0	**3l**	0	**4l**	0
20	4-COOCH_3_	CH	CH	-	25	**3n**	28^d^	**4n**	0	**3n**	11	**4n**	0
21	-	CH	N	-	25	**3o**	0	**4o**	23	**3o**	8	**4o**	21
22	-	N	CH	-	25	**3p**	14	**4p**	2	**3p**	47	**4p**	10
23	-	-	-	S	25	**3q**	83	**4q**	12	**3q**	69	**4q**	0
24	-	-	-	O	25	**3r**	54	**4r**	0	**3r**	49	**4r**	0
25	-	-	-	NH	25	**3s**	31	**4s**	0	**3s**	71	**4s**	0

a Reaction conditions: 5 mL vial, benzaldehyde (1 mmol), acetophenone (1 mmol), NaOH (1 equiv), surfactant solution (1 mL), rt or 45 °C, 24 h.

b Isolated yield.

c 2 equiv of NaOH were used.

d 29% of the derivative acid was also isolated.

Electron-donating substituents on the aldehyde gave high yields. In the presence of just an equivalent of the base, the 4-hydroxybenzaldehyde did not react in both surfactants, while when 2 equiv of the base were used, it gave 37% yield in CTAB and 27% in Tween 80 (entry 9). The 4-formylbenzoic acid, surprisingly, reacted well in CTAB with just 1 equiv of NaOH, giving **3m** in a 70% yield, while it did not react at all in Tween 80, even with 2 equiv of base (entry 19). Probably, the carboxylic group is fully deprotonated by the base and cannot interact with the nonionic micelles, thus staying mainly in water or the external part of the micelle, well far apart from the acetophenone. The yield was reduced for the 2-bromo and methoxy substituents (entries 4 and 11). The 2,6-dimethoxy substituted aldehyde reached a 90% yield, showing no issues due to steric hindrance around the aldehyde group. In a few cases, the low yields in Tween 80 were improved by raising the temperature to 45 °C for compounds **3e**, **3f**, **3h**, and **3j**, and slightly in the case of **3k** in CTAB, but not for **3g**. The reason for this beneficial effect is probably the increase in reactivity and the enhanced diffusion of reactants in the different regions of the micelles.

In CTAB, no increase in reactivity was observed, probably because the reaction is already fast due to the Coulombic attraction of the enolate with the cationic headgroups and the small micellar size. The increase in temperature can reduce the intensity of this interaction, loosening the micellar structure and thus reducing the residence time of the reactants in the micelles.

Finally, a specific discussion is opportune for the case involving heterocyclic aldehydes. The 2- and 4-pyridinecarboxaldehydes gave low to medium yields with opposite behavior. In CTAB, the 2-pyridinecarboxaldehyde gave both the chalcone **3p** and the Michael adduct **4p**, even if in low yield, while the 4-pyridinecarboxaldehyde gave only the Michael adduct **4o**, in 23% yield (entry 21). These reactants have already been shown to be quite problematic in EtOH, and their reaction conditions should be tuned with care.^81−83^

An increase in the yield was observed with Tween 80, where the 2-pyridinecarboxaldehyde gave both the chalcone **3p** at a 47% yield and 10% of the Michael adduct **4p** (entry 22). The 4-pyridinecarboxaldehyde gave 8% of the chalcone **3o** and 21% of the Michael adduct **4o** (entry 21). While CTAB seems to promote further reactions, the larger Tween 80 micelles slightly reduce the further reaction rate because of the lack of cationic headgroups. Besides, due to its higher hydrophobicity, the chalcone can enter deeply into the micellar core, being more protected from the enolate, which cannot further react. In general, for these heterocyclic aldehydes, a slight to outstanding increase in the yield can be detected, while some impurities of the Michael product are still present in both surfactants.

Unlike the pyridyl aldehydes, aldehydes from thiophene, furan, and pyrrole gave the expected chalcone in both CTAB and Tween 80. Aldehydes from thiophene, furan, and pyrrole gave **3q** with 83% and 69% (entry 23), **3r** with 54% and 49% (entry 24), and **3s** with 31% and 71% (entry 25) yields in CTAB and Tween 80, respectively.

In the case of thiophene, a better yield is achieved with CTAB, but pure product **3q** can be obtained only with Tween 80, while for the 2-furan and 2-pyrrole aldehydes, no Michael products were detected, and comparable or better yields were found. However, in general, further byproducts were found in the crude products, ranging from traces to substantial quantities, which explains the limited yields found in several cases.

The scope of the aldehydes in CTAB was also performed on a large scale to confirm that the reaction protocol is robust enough to be, in principle, scaled up. The reactions at a 5 mmol scale in CTAB at 25 °C (see Table S10) using a 20 mL vial substantially confirmed the results.

### Scope of the Acetophenones

**Scheme 3:**
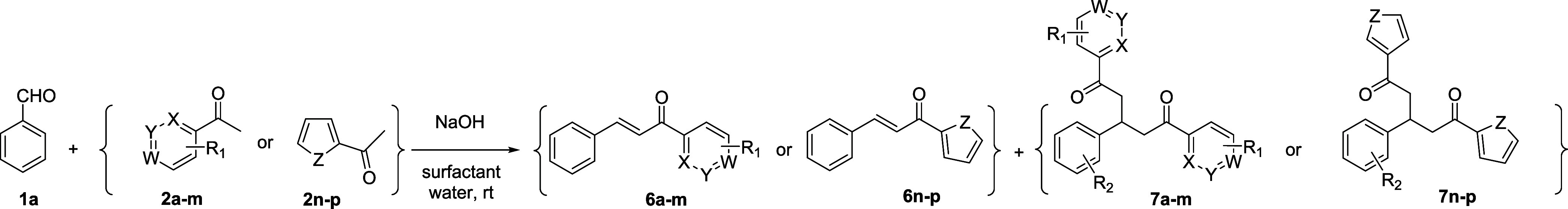
Scope of Acetophenones

The scope of the acetophenones was studied similarly to the aldehydes, and the results are reported in Table 4. The chalcones **3a** and **6b**–**p** were prepared, as depicted in Scheme 3. Using CTAB, in most cases, the yields were in the range of 65–92%, but the substituent on the acetophenone severely influenced the yield, as in the case of the NO_2_, COOH, and COOCH_3_ groups (**6h**, **6i**, and **6j** respectively) and, for the heterocycles, of acetylthiophene and acetylfuran (**6n** and **6o**). In Tween 80, the yields were very good, often better than in CTAB, e.g., for the compounds **3a** and **6b–6j**, in the range of 58–94%. However, an increase in temperature up to 45 °C can increase the final yields, as for **6b** and **6c**. The reaction can be promoted by the temperature, but sometimes, probably due to the high reactivity of one of the reactants and/or products, further reactions can occur, thus reducing the quantity of the expected chalcone, as in the case of the 4-nitroacetophenone (**6h**), for which the Michael adduct **7h** was also obtained (14%). This was a peculiar behavior since normally the Michael product was not formed in the nonionic surfactant.

**Table 4:** Scope of Acetophenones (1 mmol scale) in CTAB and Tween 80^a^

							Yield(%)^b^							
Entry	R	X	Y	W	Z	T(°C)	CTAB				Tween 80			
1	H	CH	CH	CH	-	25	**3a**	65	**4a**	10	**3a**	84	**4a**	0
2	4-CH_3_	CH	CH	CH	-	25	**6b**	84	**7b**	14	**6b**	39	**7b**	0
3	4-CH_3_	CH	CH	CH	-	45	**6b**	-	**7b**	-	**6b**	80	**7b**	0
4	4-OCH_3_	CH	CH	CH	-	25	**6c**	68	**7c**	0	**6c**	45	**7c**	0
5	4-OCH_3_	CH	CH	CH	-	45	**6c**	-	**7c**	-	**6c**	77	**7c**	0
6	2-OH	CH	CH	CH	-	25	**6d**	15	**7d**	0^c^	**6d**	82	**7d**	0^c^
7	4-Br	CH	CH	CH	-	25	**6e**	70	**7e**	14	**6e**	77	**7e**	0
8	3-Br	CH	CH	CH	-	25	**6f**	67	**7f**	0	**6f**	85	**7f**	7
9	2-Br	CH	CH	CH	-	25	**6g**	80	**7g**	0	**6g**	94	**7g**	0
10	4-NO_2_	CH	CH	CH	-	25	**6h**	26	**7h**	0	**6h**	58	**7h**	14
11	4-COOH	CH	CH	CH	-	25	**6i**	55	**7i**	0	**6i**	68	**7i**	0
12	4-COOCH_3_	CH	CH	CH	-	25	**6j**	15	**7j**	0	**6j**	19	**7j**	0
13	-	CH	CH	N	-	25	**6k**	0	**7k**	0^d^	**6k**	24	**7k**	0
14	-	CH	N	CH	-	25	**6l**	0	**7l**	0^d^	**6l**	46	**7l**	0
15	-	N	CH	CH	-	25	**6m**	21	**7m**	14	**6m**	84	**7m**	11
16	-	-	-	-	S	25	**6n**	30	**7n**	3	**6n**	79	**7n**	2
17	-	-	-	-	O	25	**6o**	38	**7o**	0	**6o**	59	**7o**	0
18	-	-	-	-	NH	25	**6p**	92	**7p**	0	**6p**	87	**7p**	0

a Reaction conditions: 5 mL vial, benzaldehyde (1 mmol), acetophenone (1 mmol), NaOH (base) (1 equiv); surfactant solution (1 mL), rt or 45 °C, 24h.

b Isolated yield.

c A flavanone was isolated for CTAB (10%) and Tween 80 (7%) instead of the Michael product.

d Cyclohexanol byproducts (3:2 acetophenone/benzaldehyde) were detected (see later in the discussion) for 4-acetylpyridine and 3-acetylpyridine.

Tween 80 gave better yields for the bromoacetophenones (**6e**–**g**), as well as for 4-nitroacetophenone (**6h**) and 4-carboxyacetophenone (**6i**). Interestingly, the 2-hydroxyacetophenone should be less reactive, since the phenolic hydrogen can interfere by reducing the acidity of CH_3_CO-Ph, thus hampering the formation of the enolate.^61^ In the CTAB solution, several byproducts were formed, with a very poor yield (**6d**, 15%). In Tween 80, the reaction was cleaner, and the **6d** product was easily isolated in a substantially enhanced yield (82%). No Michael product **7d** was produced, but around 7–10% of the corresponding flavanone **8d** was isolated (Figure S2), with both Tween 80 and CTAB, respectively.

The 4-acetylbenzoic acid reacted in both CTAB and Tween 80 surfactants, giving **6i**, while the 4-formylbenzoic acid (see above, **3m**) did not. Probably, in the presence of a base, the 4-formylbenzoic acid is ionized, and its hydrophobicity is too low to be incorporated into the Tween 80 micelles, while this does not seem to hold for the more hydrophobic 4-acetylbenzoic acid.

Indeed, the acetylpyridines did not give promising results in CTAB, with a poor yield of chalcone **6m** in the best case of 2-acetylpyridine.

The reaction of 4- and 3-acetylpyridine with benzaldehyde failed to give the expected chalcones **6k** and **6l** in CTAB. A different, major unknown product was isolated in both cases when Tween 80 was employed. By limiting the discussion to the case of the 3-acetylpyridine in CTAB, the product could be tentatively identified as the compound represented in Figure S3, or one of its stereoisomers, by the NMR of the crude (Figure S4). Constable et al., while attempting to prepare terpyridines in a one-pot reaction, already showed that 3-acetylpyridine with some aldehydes gave preferentially this kind of compound.^84^ This demonstrates that the compartmentalization of reactants into the micellar core is substantially ineffective in obtaining the desired product under those conditions, which should be properly optimized. A deeper and short discussion is reported in the Supporting Information.

In Tween 80, the 3-acetylpyridine gave the expected product **6l** in a 46% yield. Remarkably, Tween 80 drives the reaction toward the expected chalcone product, while CTAB yields the cyclohexanol reported in Figure S3, thus behaving like the ethanol solvent.^84^

For acetylpyridines, the applied conditions are not optimal and should be properly optimized. Also, 2-acetylfuran and 2-acetylthiophene produced small quantities of similar cyclohexanols (detected by NMR in the crude).

The nonionic nature of Tween 80, along with the larger dimension of the micelles, can host the chalcone deeper into the micelle, thus protecting it from further reactions.

Even in the case of the acetophenones, a scale-up to 5 mmol was performed to check for a confirmation of the results and the consistency of the synthetic method (see Tables S1–10). The yields given by the different acetophenones were often confirmed. Some improvements were observed for **6h** (31%), **6o** (67%), and **6p** (96%).

To support the general results obtained in CTAB, two reactions were also performed in Tween 80, where the yield of **6k** increased from 24% to 31% from 1 to 5 mmol scale, while the **6n** yield was substantially confirmed (83% vs 79% at the lower scale). In general, better yields and purer products are obtained by employing Tween 80 as a surfactant, and its use as a nonionic surfactant can be considered a new and viable alternative to cationic surfactants, whenever possible.

### Recycling of the Surfactant Solution

Recycling of the surfactant solution was performed on a 5 mmol scale. Compound **3h** was chosen as a representative compound that can be easily functionalized due to the presence of bromine as a substituent. At the end of the reaction, the precipitated product was separated by centrifugation. The reaction solution was further extracted once with methyl *tert*-butyl ether (MTBE) to remove the residual product, unreacted starting materials, and impurities. The surfactant solution was recycled for another reaction. Three consecutive trials were performed (Table 5).

**Table 5:** Recycling of the CTAB Solution to Synthesize Compound 3h^a^

	Yield (%)	
Trial	CTAB	Tween 80
	25 °C	45 °C
1	81	89
2	81	88
3	80	90
E-factor	4.2	3.7
PMI	5.2	4.7

a Reaction conditions: 4-bromobenzaldehyde (5 mmol), acetophenone (5 mmol), NaOH (5 mmol), surfactant solution 2% (5 mL), 24 h.

As an example, for the first cycle in CTAB at 25 °C, the total waste produced was 4.86 g, and the final yield was 1.17 g (81%). The E-factor was 4.2.

In Tween 80, the reaction was performed at 45 °C, and the yield was 88–90% and stable over the three trials, with an E-factor of 3.7. This demonstrates that the surfactant solution can be easily recycled, maintaining nearly constant yields.

### Synthesis of Representative Chalcones

**Chart 1:**
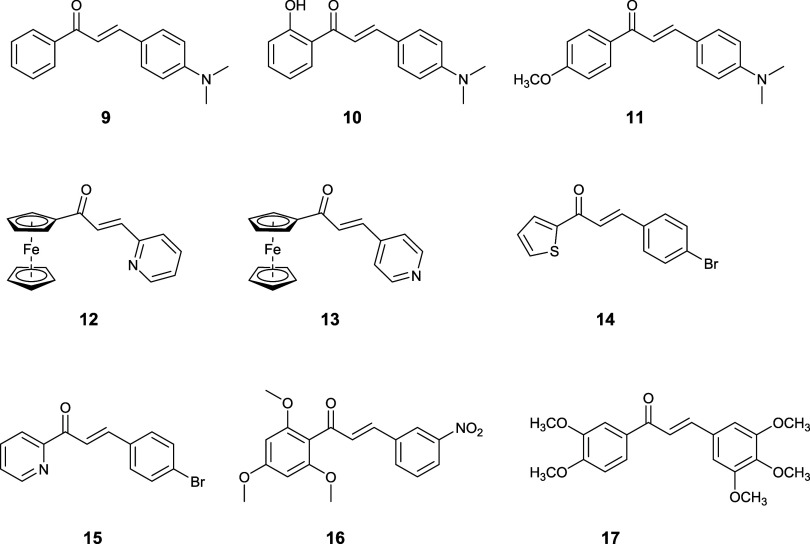
Structure of Representative Chalcones Prepared in this Work

Representative chalcones (**9**–**17**) were prepared as examples, trying to cover the main areas in which they can be exploited, such as materials and bioactive compounds like drugs (Chart 1 and Table 6).^26,85−95^

**Table 6:** Representative Chalcone Synthesis (1 mmol scale) in CTAB and Tween 80^a^

			Isolated Yield^b^ (%)	
Entry	Compound	*T* (°C)	CTAB	Tween 80
1	**9**	25	63 (11)	4
2	**10**^c^	25	1	7
3	**10**^d^	45	25	29
4	**11**	25	30	-
5	**11**	45	30 (4)	-
6	**12**	25	44	14
7	**13**	25	23	12
8	**13**	45	15	8
9	**14**	25	72 (15)	88 (7)
10	**15**	25	71 (19)	38 (24)
11	**16**	25	51	34
12	17	25	69 (2)	91

a Reaction conditions: 5 mL vial, benzaldehyde (1 mmol), acetophenone (1 mmol), NaOH (base) (1 equiv); surfactant solution (1 mL), rt or 45 °C, 24 h.

b Isolated yield (Michael in parentheses).

c NaOH (2 equiv).

d NaOH (4 equiv).

4-Dimethylaminobenzaldehyde gave sluggish reactions, with yields ranging from low to moderate since the strong donor effect of the dimethylamino substituent reduces the reactivity. Compounds **9** and **11** were obtained only with CTAB in 25–63% yields, while the Tween 80 simply did not work.

Compound **10** was challenging, since the 2′-hydroxyacetophenones give complicated reactions, and the target chalcone can further react to give flavanones. Reactions in both CTAB and Tween 80 at 25 °C and with 2 equiv of base gave just minimal yields. By working at 45 °C and with 4 equiv of base, however, no flavanone was detected, and better yields were obtained in CTAB (25%) and Tween 80 (29%), respectively, albeit still low.

Compounds **12**(96) and **13**(96) can be considered promising candidates as protective layers for perovskites used in perovskite solar cells (PSCs), where the pyridine nitrogen can suppress charge recombination by interacting with the lead of the perovskite.

Compounds **12** (44%) and **13** (23%) were obtained in CTAB at 25 °C, while Tween 80 gave minimal yields, still highlighting the different reactivity of 2- and 4-pyridinecarboxaldehydes. The increase in temperature to 45 °C did not improve the yield. Compounds **14–17** were obtained in about 50–72% yield in CTAB and 34–91% in Tween 80. For compounds **15** and **17**, our micellar reaction conditions gave better yields than the reported procedures,^89,95^ thus becoming a viable alternative to improve their preparation pathway. The micellar-based synthetic method worked well with the high structural variability of those chalcones and can surely be adapted to specific conditions, e.g., overcoming the low reactivity of specific starting materials, for which selective optimization can be performed.

### Dynamic Light Scattering (DLS)

The pristine surfactant solutions (CTAB or Tween 80 at 2% concentration) were analyzed to assess the micellar dimension before starting the reaction. The introduction of organic solutes and the interaction with inorganic salts can heavily modify the conditions of the solution, and different kinds of aggregates can appear.^97−100^ We limited the study to the determination of the micellar dimension before starting the reaction. The pure surfactants at 2% concentration gave diameters of about 1.7 nm for the CTAB and 8.9 nm for the Tween 80 (Figure S5), in reasonable agreement with the literature,^101−103^ demonstrating that Tween 80 gives larger aggregates.

### NMR Chemical Shift Measurements

The solute–micelle interaction can be studied by the variation of the surfactant’s NMR protons’ chemical shifts and by the broadening of the proton NMR signals by NMR.^104−106^

We analyzed the ^1^H NMR chemical shifts of CTAB and Tween 80 in D_2_O alone or in the presence of benzaldehyde and acetophenone at different surfactant concentrations above and below the CMC. Additionally, the effect of the base (NaOD, 1 equiv) was checked. The data are reported in Tables S12–S15.

As a result of this analysis, the reactants are incorporated into the micelles, mainly at the CTAB micellar surface or among the first methylene groups of the hydrophobic chain. For Tween 80, the benzaldehyde seems to locate with the carbonyl group close to the ester group of the Tween 80, while the ring is directed toward the internal core. The acetophenone seems to be located deeper in the internal core than the benzaldehyde. From those results, the reaction in CTAB can take place at the micellar surface with the cationic groups that interact with the aldehyde carbonyl and acetophenone enolate groups, thus favoring the reaction in a sort of organocatalytic phenomenon. In the nonionic Tween 80, the reaction probably occurs in the micelle, probably near the ester group of the surfactant.

A full and detailed discussion is reported in the Supporting Information.

### Diffusion Ordered Spectroscopy (DOSY)

Diffusion-ordered spectroscopy (DOSY) is fundamental to determining both the diffusion coefficient and the interaction of the solute with the micelle, such as the interaction of phenol with CTAB micelles.^107,108^

The simple assumptions of the proposed model (eq 1) are normally fulfilled. The solute dissolved in water diffuses freely with a diffusion coefficient (*D*_free_), while when it is associated with the micelles, it diffuses with them at their proper diffusion coefficient (*D*_sorbed_). Since the exchange of the solute between the free and sorbed state is fast over the time window of the NMR experiment, the solute average value *D*_obs_ is observed, which depends on the quantity of free and sorbed solute. By rearranging eqs 1 and 2, the percentage of the solute sorbed in the micelles, *p*, is obtained.
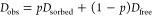
1
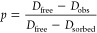
2

We measured the diffusion coefficient for the free solutes and three CTAB concentrations: 5 mM, 10 mM, and 54.8 mM (2%). The measurements at 5 mM and 54.8 mM can give information about the conditions used in previous syntheses, while the 10 mM concentration was chosen since it was shown to be an optimum condition for this kind of measurement.^108^

The surfactant diffusion coefficients were estimated by using the integral of the hydrophobic chain signals for each surfactant and fitting its decrease to the Stejskal–Tanner model (see Supporting Information for more details).^107^

For both CTAB and Tween 80, N^+^CH_3_ for CTAB and the ethylene oxide portion of Tween 80 showed more than a single diffusion coefficient.

This can account for the possible coexistence of micelles of different sizes for CTAB or, as demonstrated by Menjoge et al., for the DOSY detection of micelles that experienced at least one breakup/reconstitution event during the diffusion time of the DOSY experiment.^109^ For Tween 80, the presence of non-esterified ethoxylated sorbate can be the source of this effect, as demonstrated in the case of Tween 20.^110^ In our experiments, where a hydrophobic solute was added, a possible source for this behavior can be the association of one or more surfactant monomers (a smaller number than the micellar aggregation number) with the solute.^76−78^

The results are reported in Table 7. Representative DOSY plots for acetophenone (5 mM) alone or in CTAB (10 mM) are shown in Figures S6 and S7, respectively. *D*_free_ and *D*_obs_ were measured in the absence and presence of CTAB, respectively, while *D*_sorbed_ was the diffusion of micellized CTAB in the presence of the solute. The results obtained for the solutes are consistent with expectations (Table 7). The diffusion coefficient of the acetophenone in D_2_O was slightly higher than that for benzaldehyde, but very close to it, as expected for their similar structure.

**Table 7:** DOSY Determined the Diffusion Coefficients for Benzaldehyde (5 mM) and Acetophenone (5 mM) in CTAB at Different Concentrations

D	CTAB						
10^–10^ cm^2^ s^–1^	5 mM	10 mM	54.8 mM (2%)	5 mM	10 mM	10 mM + NaOD	54.8 mM (2%)
	Benzaldehyde			Acetophenone			
*D*_free_	7.51	7.51	7.51	7.89	7.89	7.89	7.89
*D*_obs_	6.69	6.41	4.10	7.03	6.35	5.61	3.23
*D*_sorbed_	1.09	0.98	0.82	1.16	1.10	1.01	0.85
*p* (%)	12.8	16.9	51.0	12.8	23.7	33.3	66

The percentage of sorbed solute *p* grows with the increase of the CTAB concentration, with the concentration of micelles, i.e., with the increase of the total volume of micelles, as in the case of classical solvent extraction.

The reaction, however, occurs for both 5 and 54 mM of CTAB, showing that this requires the continuous incorporation of reactants into the micelles and the expulsion of the product from the micelle in a highly dynamic process. Under the actual reaction conditions, at a 1 M concentration, the system is difficult to study by NMR, probably owing to emulsification. However, the reaction occurs, showing that a reasonable incorporation of reactants is sufficient to drive the reaction to completion.

The same measurements were performed for the interaction between CTAB and acetophenone. In the presence of 10 mM CTAB, the acetophenone diffuses with a slightly smaller diffusion coefficient than benzaldehyde. These data suggest a greater preference for acetophenone for micelles, with a greater incorporation into them (24% vs 17% for benzaldehyde).

When NaOD was introduced into this solution, the diffusion coefficient of the acetophenone decreased, and the percentage of the sorbed solute increased from 24% of the acetophenone to 33% of the enolate.

Such a huge increase (around 37%) was attributed to the electrostatic interactions between the enolate anion and the cationic surface of the CTAB micelles.

As in the case of CTAB, DOSY measurements on Tween 80 demonstrated the interaction of benzaldehyde and acetophenone with the micelles (Table 8). The internalization in micelles increases with the concentration of surfactant, i.e., of micelles in solution. The percentage of internalized benzaldehyde in Tween 80 at 54.8 mM was consistently lower (24%) than in CTAB (51%), while acetophenone is just lower (59% vs 66%).

**Table 8:** DOSY Determined Diffusion Coefficients for Benzaldehyde and Acetophenone (5 mM) in Tween 80 at Different Concentrations

D	Tween 80						
10^–10^ cm^2^ s^–1^	5 mM	10 mM	54.8 mM (2%)	5 mM	10 mM	10 mM + NaOD	54.8 mM (2%)
	Benzaldehyde			Acetophenone			
*D*_free_	7.51	7.51	7.51	7.89	7.89	7.89	7.89
*D*_obs_	6.83	6.11	5.83	7.14	6.66	3.70	3.58
*D*_mic_	0.62	0.67	0.60	1.05	0.9	0.85	0.69
*p* (%)	9.9	20.5	24.2	11.0	17.7	59.5	59.9

The lower level of incorporation of one reactant in Tween 80 can be the reason for the slower reaction rate for the reaction in Tween 80 compared to that in CTAB surfactant micelles. However, the internalization of a reactant increases with the concentration of surfactant, i.e., with the concentration of micelles in solution. Quite surprisingly, the incorporation of the enolate in Tween 80 was higher than that for benzaldehyde, attaining 59.5%. This can be tentatively explained by the tendency of the Na^+^ cation to interact with ethoxy moieties, as in the case of crown ethers.^111−113^ This probably occurs in the Tween 80 solution, and the enolate can be internalized close to the surfactant ester group and close enough to the more internal ethoxy groups. This can confirm the previous hypothesis made about the localization of the acetophenone enolate in Tween 80 micelles by chemical shift analysis.

From the results shown, we can hypothesize that the slower reactivity of Tween 80 with respect to CTAB can be interpreted as being due to the low incorporation of benzaldehyde and/or a different localization of reactants within the large micelles of Tween 80. This reduces the probability of bringing the two reactants to the same place in a micelle, making them less prone to react.

## Conclusions

This paper addresses the synthesis of chalcones through the Claisen–Schmidt reaction of aromatic aldehydes and methyl ketones, performed in a micellar medium using two surfactants, CTAB and Tween 80, in the presence of NaOH, at room temperature, and for 24 h.

This straightforward procedure performed well with wide functional group tolerability, giving, in general, good to very good yields. In most cases, the products could be isolated by simple filtration, often obtaining a product with good purity; otherwise, a single organic solvent extraction was sufficient to recover the product. Remarkably, the CTAB was prone to promote the reaction, probably related to the surfactant cationic headgroup attraction for both the hydroxide anion and the enolate. However, Tween 80 performed quite well and, in several cases, outperformed the CTAB results and, whenever possible, can be proposed as a reliable alternative to cationic surfactants.

Critical behavior was shown by the aromatic aldehyde bearing free hydroxy substituents, which required two equiv of the base to react, giving moderate yields. Pyridinecarboxaldehydes showed problematic reactivity since sometimes the chalcone was further transformed into the Michael adduct because CTAB was prone to promote domino reactions and can be proposed to effect multicomponent reactions, where multiple steps should be performed in sequence. On the contrary, no Michael adduct was isolated when the reaction was performed in nonionic surfactants, such as Tween 80. This reaction probably occurs in a different region with respect to CTAB, and the chalcone is protected in the micelle from the enolate, avoiding further reactivity. Further experiments are required to confirm this hypothesis. Information about the micelles and micellar interaction with the reactants was obtained from DLS and NMR measurements by studying the interaction of model reactants with both surfactants through chemical shift analysis and DOSY experiments. It was demonstrated that the reactants are concentrated at the surface of CTAB micelles, while in Tween 80 they are probably located in a more internal region of the micelle. Benzaldehyde is more strongly associated with CTAB than with Tween 80, and this can be the main reason for explaining failures or low yields in Tween 80. These results showed that the reaction can be influenced by the surfactant and that, probably, specific surfactants and conditions should be used to improve the reactivity of some substrates, such as the heterocyclic aldehydes. The recycling of the surfactant solution gave excellent performance with a constant level of yield over successive trials.

## Experimental Section

All the experimental details are reported in the Supporting Information.

## Data Availability

The data underlying this study are available in the published article and its Supporting Information.

## Supporting Information Available

Materials and methods, synthetic procedures, ^1^H, ^13^C, and DOSY NMR spectra, E-Factor and PMI calculations, more extended discussions (PDF)
