# Supplementary material for: Chalcone Synthesis by Green Claisen–Schmidt Reaction in Cationic and Nonionic Micellar Media
Source: J Org Chem. 2025 Feb 18;90(8):2915–26. doi: 10.1021/acs.joc.4c02616 (PMC11877513; doi:10.1021/acs.joc.4c02616)
Supplement: Supplementary file 1 — jo4c02616_si_001.pdf [file jo4c02616_si_001.pdf]

Supporting Information

Chalcones synthesis by green Claisen-Schmidt reaction in cationic and nonionic micellar media

Davide Dotta,[a] Matteo Gastaldi,[a,‡] Andrea Fin,[a] Nadia Barbero,[a,b] Claudia Barolo,[a,b,c] Francesca Cardano,[a] Federica Rossi,[d] Francesca Brunelli,[e] Guido Viscardi,[a,b] Gian Cesare Tron,[e] Pierluigi Quagliotto[a,b]

[a] *Dipartimento di Chimica, Università di Torino, via P. Giuria 7, 10125, Torino, Italy*

[b] *NIS Interdepartmental Centre and INSTM Reference Centre, University of Torino, Via Gioacchino Quarello 15/a, Torino 10125, Italy*

[c] *Istituto di Scienza, Tecnologia e Sostenibilità per lo sviluppo dei Materiali Ceramici (ISSMC-CNR), Via Granarolo 64, RA, Faenza, 48018, Italy*

[d] *Dipartimento di Scienza e Tecnologia del Farmaco, Università di Torino, via P. Giuria 11, 10125, Torino, Italy*

[e] *Dipartimento di Scienze del Farmaco, Università del Piemonte Orientale, Largo Donegani 2, 28100, Novara, Italy*

[‡] *Present address: GAME Lab, Department of Applied Science and Technology (DISAT), Politecnico di Torino, Corso Duca degli Abruzzi 24, Torino, 10129, Italy*

corresponding author e-mail: pierluigi.quagliotto@unito.it

Contents

Experimental S-4
Materials and Methods S-4
Synthesis S-5
General procedure for the optimization of the synthesis of the chalcones S-5
General procedure for the synthesis of the chalcones S-5
Procedure for the recycling of the surfactant solution (case of compound 3h) S-5
Photostability of compound 11 .... S-6
SERIES 3 .... S-7
SERIES 4 .... S-17
SERIES 6 .... S-21
SERIE 7 .... S-28
Other Compounds (8-17) S-31
NMR Spectra (at 600 MHz for 1H and 151 MHz for 13C{1H}) S-37
^{1}H and ^{13}C{1H} spectra of series 3 .... S-37
^{1}H and ^{13}C{1H} spectra of series 4 .... S-56
^{1}H and ^{13}C{1H} spectra of series 6 .... S-64
^{1}H and ^{13}C{1H} spectra of series 7 .... S-79
^{1}H and ^{13}C{1H} spectra of compounds 8-17 .... S-85
Determination of the Green Metrics S-95
E-Factor calculation S-95
Synthesis of 3a in Ethanol S-95
Synthesis in CTAB S-96
Chalcones from the Scope of the Aldehydes S-96
Chalcones from the Scope of the Acetophenones S-106
Synthesis in TWEEN 80 .... S-122
Chalcones from the Scope of the Aldehydes S-122
Chalcones from the Scope of the Acetophenones S-134
Representative Chalcones S-143
E-factor from recycling experiments S-151
Process Mass Intensity calculation S-154
Scale-up for Aldehydes S-158
Further Material and Discussions S-159
Structure of side products S-160
Tentative identification of a side-product (Fig S3) by NMR of the reaction crude. S-160
Optimization of the base concentration S-161
Characterization of the micellar solutions S-162

DLS Measurements S-162
NMR characterization: chemical shift S-162
CTAB S-162
Tween 80 .... S-164
NMR Characterization: DOSY S-167
References S-168

Experimental

Materials and Methods

All chemicals were purchased from Merck, VWR and TCI and used as received. Surfactants solutions were prepared by dissolving the proper quantity of surfactant into a 100 mL flask and adding half of the required water. The dissolution of CTAB was done by heating over 26°C to overcome the surfactant Krafft point.[1] Tween 80 is a viscous liquid, which was dissolved by alternating heating at 40°C and sonication, up to complete disappearance of the surfactant viscous phase. After complete dissolution of the surfactant, the volume was brought to 100 mL with water. Surfactants solutions were used for a maximum of 15 days before replacing with new ones.

Reactions were performed in either 8 mL vials (16 mm diameter) for 1 mmol scale reactions or 20 mL MW Biotage vials for 5 mmol scale reactions. Liquid reactants were introduced by a Hamilton syringe.

Purification of products was performed with flash chromatography with a Biotage Isolera chromatograph on 10 g SNAP ULTRA Silica gel columns, working using a gradient of petroleum ether /ethyl acetate.

Melting points were measured with a Stuart Scientific SMP3 melting point apparatus.

NMR spectra were recorded in $CDCl_3$, on a Bruker AVANCE 400 working at 400 MHz for ^1H and 100 MHz for $^{13}C\{1H\}$ for reaction optimization and on a JEOL ECZ-R 600, working at 600 MHz for ^1H and 151 MHz for $^{13}C\{1H\}$ for final characterization.

The ^1H spectra of some Michael products obtained after crystallization from ethanol showed the presence of ethanol in a 1:1 ratio with the Michael product. The crystals were left a few hours under high vacuum pump, but the quantity of ethanol was just slightly reduced. This indicated that probably the crystals are containing ethanol as part of the crystalline unit cell. Their NMR spectra were thus reported with the indication of the ethanol peaks.

DOSY spectra were acquired with the following conditions:

For pure benzaldehyde and acetophenone in D_2O, spectra were acquired with diffusion time 50 ms, delta 1 ms and recovering 16 spectra with a logarithmic set of gradients from 0.2 mT/m to 800 mT/m.

For CTAB micellar samples at all concentrations spectra were acquired with diffusion time 50 ms (150 ms for 2% / 55mM), delta 2 ms and recovering 16 spectra with a logarithmic set of gradients from 0.2 mT/m to 800 mT/m.

For CTAB spectra containing either benzaldehyde or acetophenone (5mM) spectra were acquired with diffusion time 50 ms, delta 2 ms (1 ms for CTAB 5 mM) and recovering 32 spectra with a logarithmic set of gradients from 0.2 mT/m to 800 mT/m.

For CTAB 10 mM containing acetophenone (5mM) and NaOD (5mM) spectra were acquired with diffusion time 50 ms, delta 2 ms and recovering 32 spectra with a logarithmic set of gradients from 0.2 mT/m to 800 mT/m.

For Tween 80 micellar samples at all concentrations (0.005, 5, 10 mM and 2%) spectra were acquired with diffusion time 50 ms, delta 2 ms and recovering 16 spectra with a logarithmic set of gradients from 0.2 mT/m to 800 mT/m.

Tween 80 spectra containing either benzaldehyde or acetophenone (5mM) were acquired with diffusion time 50 ms, delta 2 ms and recovering 32 spectra with a logarithmic set of gradients from 0.2 mT/m to 800 mT/m.

HR-MS analyses were performed using an Orbitrap IQ-X mass spectrometer (Thermo Fisher Scientific) equipped with a heated electrospray ionization (HESI) source. MS parameters were optimized to enhance ionization and to minimize in-source fragmentation. The ion source was operated in positive ion mode using the following settings: spray voltage of 3.3 kV (+), ion transfer tube temperature of 290°C and vaporizer temperature of 300 °C. Nitrogen was used as both sheath gas and auxiliary gas, with flow rates set at 40 and 10 arbitrary units (a.u.), respectively.

Synthesis

General procedure for the optimization of the synthesis of the chalcones

In an 8 mL vial the benzaldehyde (1 mmol, 1 eq.), the acetophenone (1 mmol, 1 eq.) and a 2 % surfactant solution (1 mL) were introduced along with a stir bar and the vial was closed with a plastic cap. The mixture was stirred with a magnetic stirrer for 1-2 minutes and the NaOH (100 mL of a 40 % solution, 40 mg, 1 mmol, 1 eq.) was added. The mixture was stirred for the required time at high-speed rate (around 1000 rpm) to enhance the transfer rate of reactants from water to the micelles. After the 24 h, the reaction was diluted with 1 mL of ethyl acetate and stirred for 2 minutes. The mixture was filtered through a short pad of silica gel and the organic phase was evaporated and the crude was analysed by NMR with heptane as internal standard.

General procedure for the synthesis of the chalcones

In an 8 mL vial the benzaldehyde (1 mmol, 1 eq.), the acetophenone (1 mmol, 1 eq.) and a 2% surfactant solution (1 mL) were introduced along with a stir bar and the vial was closed with a plastic cap. The mixture was stirred with a magnetic stirrer for 1-2 minutes and the NaOH (100 mL of a 40% solution, 40 mg, 1 mmol, 1 eq.) was added. The mixture was stirred for 24h at high-speed rate (around 1000 rpm) to enhance the transfer rate of reactants from water to the micelles. After 24 h, the reaction was diluted with 1 mL of ethyl acetate and stirred for 2 minutes. The organic phase was separated and dried with Na_2SO_4 and filtered through a short pad of silica. The organic phase was evaporated. The product was purified by column flash chromatography on Biotage with a gradient of petroleum ether /ethyl acetate. When the product precipitated out from the solution it was filtered. In these cases, no appreciable loss of yield was found by recovering the precipitate with respect to the extraction with ethyl acetate.

Procedure for the recycling of the surfactant solution (case of compound 3h)

The reactants were inserted into a Biotage 20 mL MW vial the reactants were introduced in this order: 4-bromobenzaldehyde (925 mg, 5 mmol, 1 eq.), acetophenone (601 mg, mL, 583 microliters, 5 mmol, 1 eq.), CTAB (2 %) or Tween 80 (2 %) 5 mL, NaOH 10 M (200 mg, 0.5 mL, 5 mmol, 1 eq.) solution. The reaction was stirred for 24 h at rt for CTAB and at rt or 45 °C for Tween 80. At the end, the solid was separated by centrifugation. The product was nearly pure and can be further by purified by crystallization from ethanol.

The water phase was extracted with MTBE (5 mL) and the solvent was separated, dried and evaporated. The small quantity of product contained in this fraction was recovered by column chromatography on silica with petroleum ether / ethyl acetate gradient. The water phase was just left to evaporate the remaining traces of the organic solvent, transferred back into the reactor, further aliquots of reactants were added, and the reaction was repeated.

From CTAB: Yield: 1.165 g (81.1 %).

From Tween 80 at 45°C: Yield: 1.279 mg (89 %).

Photostability of compound 11.

When we recorded the NMR spectrum of compound **11** after purification by column chromatography and by crystallization from ethanol, some minimal impurities seemed to be present. Measuring the ^{1}H NMR spectrum on the same sample after some time showed an increase of signals, which were not superimposed to the compound signals. This was more evident on the compound crystallized by ethanol, for which some ethanol remained in the solid probably as part of the crystalline cell. The analysis of the spectrum showed that the main compound obtained by purification was the E isomer (coupling constant for the alkene protons: 15.4 Hz), while the emerging compound (impurity) was the Z isomer (coupling constant for the alkene protons: 12.8 Hz). A further experiment on the same sample showed that by irradiating with a UV lamp for 45 minutes the pristine sample where the ratio E:Z was around 2.5:1, the spectrum showed a ratio of about 1.1:1. People who need to produce and/or manipulate this compound should consider to not expose it even to ambient light when working.

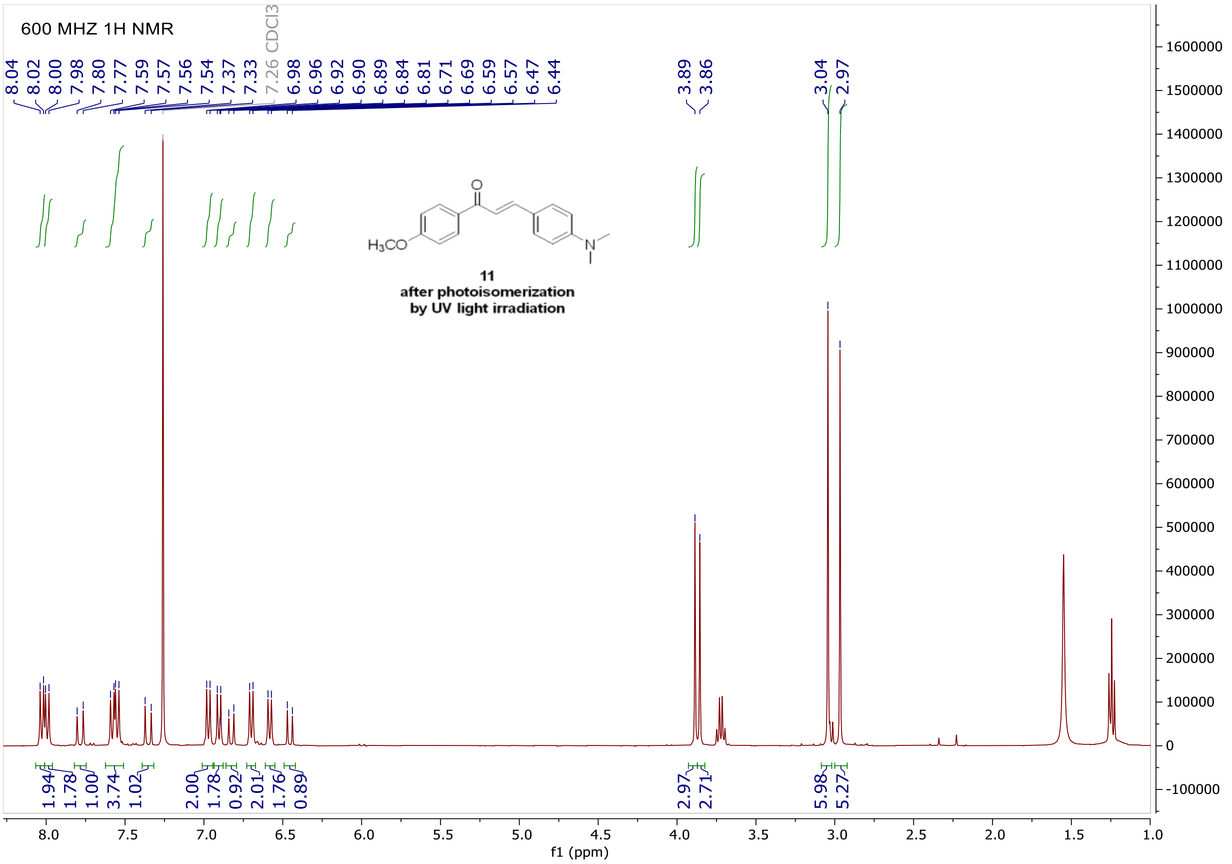

SERIES 3

(E)-1,3-diphenylprop-2-en-1-one (3a) - Reaction in ethanol.[2]

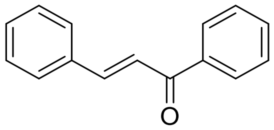

In an 8 mL vial, benzaldehyde (106 mg, 102 µl, 1 mmol, 1 eq.), acetophenone (120 mg, 117 µl, 1 mmol, 1 eq.) and 1mL of absolute ethanol were introduced along with a stir bar and the vial was closed with a cap. The mixture was stirred for 1-2 minutes and the NaOH (100 µl of a 40 % solution, 40 mg NaOH, 1 mmol, 1 eq.) was added. The mixture was stirred for 24 h at high-speed rate (around 1000 rpm) to enhance the transfer rate of reactants from water to the micelles. After 24 h, the reaction was diluted with 1 mL of ethyl acetate and stirred for 2 minutes. mixture was filtered through a short pad of silica gel and the organic phase was evaporated and the crude was further purified by column chromatography with a gradient of petroleum ether and ethyl acetate. A final crystallization from ethanol gave the pure compound.

(E)-1,3-diphenylprop-2-en-1-one (3a)[2]

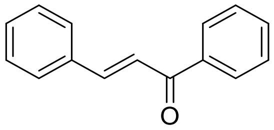

The reaction was performed following the general procedure by introducing in sequence benzaldehyde (106 mg, 102 µl, 1 mmol, 1 eq.), acetophenone (120 mg, 117 µl, 1 mmol, 1 eq.), 1mL of CTAB 2 % or Tween 80 2 %, and NaOH (100 µl of a 40% solution, 40 mg NaOH, 1 mmol, 1 eq). The crude was further purified by column chromatography with a gradient of petroleum ether and ethyl acetate. A final crystallization from ethanol gave the pure compound.

From CTAB: Yield: 134.5 mg (65 %).

From Tween 80: Yield: 174.9 mg (84 %).

White solid, m.p. 59-61 °C (Lit. 60-61 °C[2]).

^{1}H NMR (600 MHz, $CDCl_3$) δ 8.03 (dd, J = 8.4, 1.3 Hz, 2H), 7.82 (d, J = 15.8 Hz, 1H), 7.65 (m, 2H), 7.59 (m, 1H), 7.54 (d, J = 15.8 Hz, 1H), 7.51 (m, 2H), 7.45 – 7.41 (m, 3H).

^{13}C{1H} NMR (151 MHz, $CDCl_3$) δ 190.7, 145.0, 138.3, 135.00, 132.9, 130.7, 129.1, 128.8, 128.63, 128.58, 122.2.

HRMS (ESI+) m/z: [M + H]+ Calcd for C15H13O 209.0966; Found 209.0960.

(E)-N-(4-(3-oxo-3-phenylprop-1-en-1-yl)phenyl)acetamide (3b)[3]

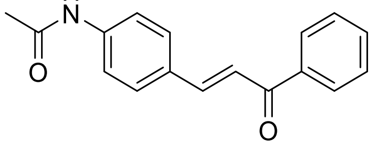

The reaction was performed following the general procedure by introducing in sequence in the vial: 4-acetamidobenzaldehyde (163 mg, 1 mmol, 1 eq.), acetophenone (120 mg, 117 ml, 1 mmol, 1 eq.), 1mL of CTAB 2% or Tween 80 2%, and NaOH (100 µl of a 40 % solution, 40 mg NaOH, 1 mmol, 1 eq). The crude was further purified by column chromatography with a gradient of petroleum ether and ethyl acetate. A final crystallization from ethanol gave the pure compound.

From CTAB: Yield: 210.1 mg (79 %).

From Tween 80: Yield: 241.4 mg (85 %).

Yellow solid, m.p. 180-181 °C (Lit. 181-182 °C[3]).

^{1}H NMR (600 MHz, $CDCl_3$) δ 8.01 (dd, J = 8.3, 1.3 Hz, 2H), 7.77 (d, J = 15.7 Hz, 1H), 7.60 (m, 5H), 7.51 (m, 2H), 7.47 (d, J = 15.7 Hz, 1H), 7.41 (s, 1H), 2.21 (s, 3H).

^{13}C{1H} NMR (151 MHz, $CDCl_3$) δ 190.7, 168.5, 144.4, 140.2, 138.4, 132.9, 130.85, 129.6, 128.8, 128.6, 121.2, 119.81, 24.9.

HRMS (ESI+) m/z: [M + H]+ Calcd for C17H16NO2 266.1181; Found 266.1179.

(E)-1-phenyl-3-(4-tolyl)prop-2-en-1-one (**3c**)[4, 5]

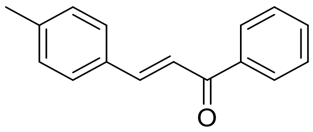

The reaction was performed following the general procedure by introducing in sequence in the vial: 4-methylbenzaldehyde (120 mg, 118 µl, 1 mmol, 1 eq.), acetophenone (120 mg, 117 ml, 1 mmol, 1 eq.), 1mL of CTAB 2% or Tween 80 2%, and NaOH (100 µl of a 40 % solution, 40 mg NaOH, 1 mmol, 1 eq.). The crude was further purified by column chromatography with a gradient of petroleum ether and ethyl acetate. A final crystallization from ethanol gave the pure compound.

From CTAB: Yield: 177.8 mg (80 %).

From Tween 80: Yield: 172.9 mg (78 %).

White solid, m.p. 86-87 °C (Lit. 85-87 °C[5]).

^{1}H NMR (600 MHz, $CDCl_3$) δ 8.01 (m, 2H), 7.80 (d, J = 15.7 Hz, 1H), 7.58 (m, 1H), 7.55 (d, J = 8.2 Hz, 2H), 7.50 (m, 3H), 7.24 (m, 2H), 2.40 (s, 3H).

^{13}C{1H} NMR (151 MHz, $CDCl_3$) δ 190.9, 145.1, 141.3, 138.5, 132.8, 132.3, 129.9, 128.7, 128.6, 128.6, 121.3, 21.7.

HRMS (ESI+) m/z: [M + H]+ Calcd for C16H15O 223.1123; Found 223.1119.

(E)-3-(4-methoxyphenyl)-1-phenylprop-2-en-1-one (**3d**)[6]

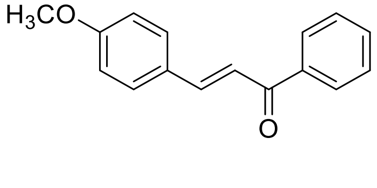

The reaction was performed following the general procedure by introducing in sequence in the vial: 4-methoxybenzaldehyde (136 mg, 123 µl, 1 mmol, 1 eq.), acetophenone (120 mg, 117 ml, 1 mmol, 1 eq.), 1mL of CTAB 2% or Tween 80 2%, and NaOH (100 µl of a 40 % solution, 40 mg NaOH, 1 mmol, 1 eq.). The crude was further purified by column chromatography with a gradient of petroleum ether and ethyl acetate. A final crystallization from ethanol gave the pure compound.

From CTAB: Yield: 195.4 mg (82 %).

From Tween 80 at 25°: Yield: 81.0 mg (34 %).

From Tween 80 at 45°C: Yield: 158.2 mg (66 %).

White solid, m.p. 62-64 °C (Lit. 61-63 °C[6], 65 °C[7]).

^{1}H NMR (600 MHz, $CDCl_3$) δ 8.01 (m, 2H), 7.79 (d, J = 15.6 Hz, 1H), 7.61 (d, J = 8.8 Hz, 2H), 7.57 (m, 1H), 7.50 (m, 2H), 7.42 (d, J = 15.6 Hz, 1H), 6.94 (d, J = 8.7 Hz, 2H), 3.86 (s, 3H).

^{13}C{1H} NMR (151 MHz, $CDCl_3$) δ 190.8, 161.8, 144.9, 138.7, 132.7, 130.4, 128.7, 128.6, 127.8, 119.9, 114.6, 55.6.

HRMS (ESI+) m/z: [M + H]+ Calcd for C16H15O2 239.1072; Found 239.1069.

(E)-3-(2-methoxyphenyl)-1-phenylprop-2-en-1-one (3e)[8]

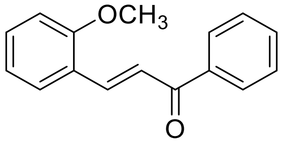

The reaction was performed following the general procedure by introducing in sequence in the vial: 2-methoxybenzaldehyde (136 mg, 121 µl, 1 mmol, 1 eq.), acetophenone (120 mg, 117 ml, 1 mmol, 1 eq.), 1mL of CTAB 2% or Tween 80 2 %, and NaOH (100 µl of a 40 % solution, 40 mg NaOH, 1 mmol, 1 eq.). The crude was further purified by column chromatography with a gradient of petroleum ether and ethyl acetate. A final crystallization from ethanol gave the pure compound.

From CTAB: Yield: 141.1 mg (59 %).

From Tween 80: Yield: 95.3 mg (40 %).

White solid, m.p. 53-54 °C (Lit. 53-54 °C[9], 52-53 °C[8]).

^{1}H NMR (600 MHz, $CDCl_3$) δ 8.13 (d, J = 15.9 Hz, 1H), 8.02 (dd, J = 8.3, 1.3 Hz, 2H), 7.64 (m, 2H), 7.57 (m, 1H), 7.50 (m, 2H), 7.38 (ddd, J = 8.3, 7.4, 1.7 Hz, 1H), 7.00 (td, J = 7.5, 1.0 Hz, 1H), 6.95 (dd, J = 8.3, 1.1 Hz, 1H), 3.92 (s, 3H).

^{13}C{1H} NMR (151 MHz, $CDCl_3$) δ 191.3, 158.9, 140.6, 138.7, 132.7, 131.9, 129.4, 128.7, 124.0, 123.0, 120.9, 111.4, 55.7.

HRMS (ESI+) m/z: [M + H]+ Calcd for C16H15O2 239.1072; Found 239.1068.

(E)-3-(2,6-dimethoxyphenyl)-1-phenylprop-2-en-1-one (3f)[10]

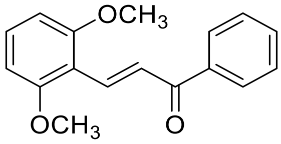

The reaction was performed following the general procedure by introducing in sequence in the vial: 2,6-dimethoxybenzaldehyde (166 mg, 1 mmol, 1 eq.), acetophenone (120 mg, 117 ml, 1 mmol, 1 eq.), 1mL of CTAB 2 % or Tween 80 2 %, and NaOH (100 µl of a 40 % solution, 40 mg NaOH, 1 mmol, 1 eq.). Th The crude was further purified by column chromatography with a gradient of petroleum ether and ethyl acetate.

From CTAB: Yield: 237.0 mg (90 %).

From Tween 80 at 25°C: Yield: 140.1 mg (59 %).

From Tween 80 at 45°C: Yield: 221.6 mg (93 %).

Viscous yellow oil.

^{1}H NMR (600 MHz, $CDCl_3$) δ 8.27 (d, J = 16.0 Hz, 1H), 8.01 (m, 3H), 7.55 (m, 1H), 7.49 (m, 2H), 7.30 (t, J = 8.4 Hz, 1H), 6.59 (d, J = 8.4 Hz, 2H), 3.92 (s, 6H).

^{13}C{1H} NMR (151 MHz, $CDCl_3$) δ 192.4, 160.5, 139.1, 136.0, 132.4, 131.7, 128.7, 128.6, 125.1, 113.0, 103.9, 56.0.

HRMS (ESI+) m/z: [M + H]+ Cald for C17H17O3 269.1178; Found 269.1171.

(E)-3-(4-hydroxyphenyl)-1-phenylprop-2-en-1-one (3g)[4, 6, 7]

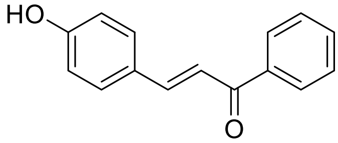

The reaction was performed following the general procedure by introducing in sequence in the vial: 4-hydroxyoxybenzaldehyde (122 mg, 1 mmol, 1 eq.), acetophenone (120 mg, 117 ml, 1 mmol, 1 eq.), 1mL of CTAB 2% or Tween 80 2%, and NaOH (200 µl of a 40 % solution, 80 mg NaOH, 1 mmol, 1 eq.). The crude was further purified by column chromatography with a gradient of petroleum ether and ethyl acetate. A final crystallization from ethanol gave the pure compound.

From CTAB at 25°C: Yield: 83.2 mg (37 %).

From CTAB at 45°C: Yield: 88.2 mg (39 %).

From Tween 80 at 25°C: Yield: 59.9 mg (27 %).

From Tween 80 at 45°C: Yield: 46.0 mg (21 %).

Yellow solid, m.p. 179-180 °C (Lit. 179-180 °C[6]).

^{1}H NMR (600 MHz, $CDCl_3$) δ 8.01 (d, J = 6.9 Hz, 2H), 7.78 (d, J = 15.6 Hz, 1H), 7.57 (m, 3H), 7.51 (m, 2H), 7.41 (d, J = 15.6 Hz, 1H), 6.89 (d, J = 8.6 Hz, 2H), 5.52 (s, 1H).

^{13}C{1H} NMR (151 MHz, $CDCl_3$) δ 191.0, 158.2, 145.0, 138.6, 132.8, 130.7, 128.8, 128.6, 127.9, 120.0, 116.2.

HRMS (ESI+) m/z: [M + H]+ Calcd for C15H13O2: 225.0916; Found 225.0912.

(E)-3-(4-bromophenyl)-1-phenylprop-2-en-1-one (3h)[11-14]

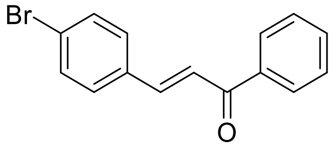

The reaction was performed following the general procedure by introducing in sequence in the vial: 4-bromobenzaldehyde (185 mg, 1 mmol, 1 eq.), acetophenone (120 mg, 117 ml, 1 mmol, 1 eq.), 1mL of CTAB 2 % or Tween 80 2 %, and NaOH (100 µl of a 40 % solution, 40 mg NaOH, 1 mmol, 1 eq.). The crude was further purified by column chromatography with a gradient of petroleum ether and ethyl acetate. A final crystallization from ethanol gave the pure compound.

From CTAB: Yield: 234.7 mg (82 %).

From Tween 80 at 25°C: Yield: 86.1 mg (30 %).

From Tween 80 at 45°C: Yield: 258.4 mg (90 %).

White solid, m.p. 126-127 °C (Lit. 127-128 °C[11], 120-122 °C[14]).

^{1}H NMR (600 MHz, $CDCl_3$) δ 8.03 (dd, J = 8.4, 1.3 Hz, 2H), 7.82 (d, J = 15.8 Hz, 1H), 7.65 (m, 2H), 7.59 (m, 1H), 7.54 (d, J = 15.8 Hz, 1H), 7.51 (m, 2H), 7.42 (m, 3H).

^{13}C{1H} NMR (151 MHz, $CDCl_3$) δ 190.4, 143.5, 138.2, 134.0, 133.1, 132.4, 129.95, 128.8, 128.7, 125.0, 122.7.

HRMS (ESI+) m/z: (M + H)]+ Calcd for C15H12BrO 287.0072, 289.0046; Found 287.0070, 289.0047.

(E)-3-(3-bromophenyl)-1-phenylprop-2-en-1-one (3i)[15]

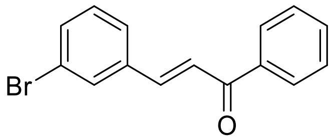

The reaction was performed following the general procedure by introducing in sequence in the vial: 3-bromobenzaldehyde (185 mg, 117 µl, 1 mmol, 1 eq.), acetophenone (120 mg, 117 ml, 1 mmol, 1 eq.), 1mL of CTAB 2 % or Tween 80 2 %, and NaOH (100 µl of a 40 % solution, 40 mg NaOH, 1 mmol, 1 eq.). The crude was further purified by column chromatography with a gradient of petroleum ether and ethyl acetate. A final crystallization from ethanol gave the pure compound.

From CTAB: Yield: 258.4 mg (90 %).

From Tween 80: Yield: 217.4 mg (76 %).

White solid, m.p. 82-84 °C (Lit. 84 °C[16]).

^{1}H NMR (600 MHz, $CDCl_3$) δ 8.03 (dd, J = 8.4, 1.3 Hz, 1H), 7.80 (t, J = 1.8 Hz, 1H), 7.73 (d, J = 15.7 Hz, 1H), 7.63 – 7.58 (m, 1H), 7.57 – 7.50 (m, 4H), 7.30 (t, J = 7.8 Hz, 1H).

$^{13}C\{1H\}$ NMR (151 MHz, $CDCl_3$) δ 190.3, 143.1, 138.6, 137.2, 133.4, 133.2, 131.0, 130.6, 128.9, 128.7, 127.4, 123.4, 123.3.

HRMS (ESI+) m/z: [M + H]+ Calcd for C15H12BrO 287.0072, 289.0046; Found 287.0068, 289.0045.

(E)-3-(2-bromophenyl)-1-phenylprop-2-en-1-one (3j)[17]

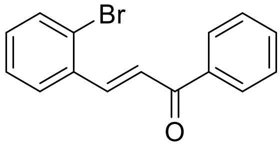

The reaction was performed following the general procedure by introducing in sequence in the vial: 2-bromobenzaldehyde (185 mg, 117 µl, 1 mmol, 1 eq.), acetophenone (120 mg, 117 ml, 1 mmol, 1 eq.), 1mL of CTAB 2 % or Tween 80 2 %, and NaOH (100 µl of a 40 % solution, 40 mg NaOH, 1 mmol, 1 eq.). The crude was further purified by column chromatography with a gradient of petroleum ether and ethyl acetate. A final crystallization from ethanol gave the pure compound.

From CTAB: Yield: 156.8 mg (55 %).

From Tween 80 at 25°C: Yield: 195.3 mg (68 %).

From Tween 80 at 45°C: Yield: 241.2 mg (84 %).

White solid, m.p. 71-73 °C (Lit. 72 °C[18]).

^1H NMR (600 MHz, $CDCl_3$) δ 8.13 (d, J = 15.7 Hz, 1H), 8.02 (dd, J = 8.3, 1.3 Hz, 2H), 7.74 (dd, J = 7.8, 1.6 Hz, 1H), 7.64 (m, 1H), 7.60 (m, 1H), 7.52 (ddd, J = 8.5, 7.1, 0.6 Hz, 2H), 7.43 (d, J = 15.7 Hz, 1H), 7.36 (m, 1H), 7.26 (m, 1H),

$^{13}C\{1H\}$ NMR (151 MHz, $CDCl_3$) δ 190.6, 143.4, 138.0, 135.2, 133.7, 133.1, 131.5, 128.8, 128.0, 127.9, 126.0, 125.2.

HRMS (ESI+) m/z: [M + H]+ Calcd for C15H12BrO 287.0072, 289.0046; Found 287.0067, 289.0044.

(E)-3-(4-nitrophenyl)-1-phenylprop-2-en-1-one (3k)[4, 6]

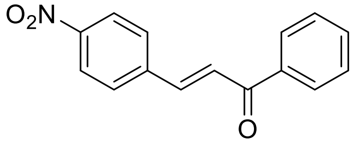

The reaction was performed following the general procedure by introducing in sequence in the vial: 4-nitrobenzaldehyde (151 mg, 1 mmol, 1 eq.), acetophenone (120 mg, 117 µl, 1 mmol, 1 eq.), 1mL of CTAB 2 % or Tween 80 2 %, and NaOH (100 µl of a 40 % solution, 40 mg NaOH, 1 mmol, 1 eq.). The crude was further purified by column chromatography with a gradient of petroleum ether and ethyl acetate. A final crystallization from ethanol gave the pure compound.

From CTAB: Yield: 172.2 mg (69 %).

From CTAB at 45°C: Yield: 184.6 mg (73 %).

From Tween 80: Yield: 220.3 mg (87 %).

Yellow solid, m.p. 164-166 °C (Lit. 163-164°C[6], 164-167°C[16]).

^{1}H NMR (600 MHz, $CDCl_3$) δ 8.29 (d, J = 8.7 Hz, 2H), 8.04 (m, 2H), 7.83 (d, J = 15.8 Hz, 1H), 7.80 (m, 2H), 7.65 (d, J = 15.8 Hz, 1H), 7.63 (t, J = 7.4 Hz, 1H), 7.54 (m, 2H).

^{13}C{1H} NMR (151 MHz, $CDCl_3$) δ 189.8, 148.7, 141.7, 141.2, 137.7, 133.6, 129.10, 129.0, 128.8, 125.9, 124.4.

HRMS (ESI+) m/z: [M + H]+ Calcd for C15H12NO3 254.0817; Found 254.0813.

(E)-4-(3-oxo-3-phenylprop-1-en-1-yl)benzonitrile (3l)[19]

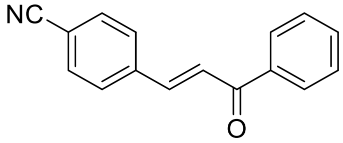

The reaction was performed following the general procedure by introducing in sequence in the vial: 4-cyanobenzaldehyde (131 mg, 1 mmol, 1 eq.), acetophenone (120 mg, 117 µl, 1 mmol, 1 eq.), 1mL of CTAB 2 % or Tween 80 2 %, and NaOH (100 µl of a 40 % solution, 40 mg NaOH, 1 mmol, 1 eq.). The crude was further purified by column chromatography with a gradient of petroleum ether and ethyl acetate. A final crystallization from ethanol gave the pure compound.

From CTAB: Yield: 154.9 mg (66 %).

From Tween 80: Yield: 227.7 mg (98 %).

White solid, m.p. 152-154 °C (Lit. 151-153°C[16]).

^{1}H NMR (600 MHz, $CDCl_3$) δ 8.03 (dd, *J* = 8.3, 1.4 Hz, 2H), 7.78 (d, *J* = 15.7 Hz, 1H), 7.73 (d, *J* = 3.9 Hz, 4H), 7.62 (t, *J* = 7.4, 1.3 Hz, 1H), 7.61 (d, *J* = 15.8 Hz, 1H), 7.53 (dd, *J* = 8.0, 7.4 Hz, 2H).

^{13}C{1H} NMR (151 MHz, $CDCl_3$) δ 189.9, 142.2, 139.4, 137.8, 133.5, 132.9, 129.0, 128.9, 128.7, 125.2, 118.5, 113.7.

HRMS (ESI+) m/z: [M + H]+ Calcd for C16H12NO 234.0919; Found 234.0917.

(E)-4-(3-oxo-3-phenylprop-1-en-1-yl)benzoic acid (3m)[20]

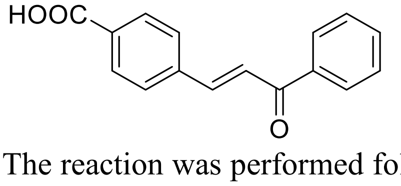

The reaction was performed following the general procedure by introducing in sequence in the vial: 4-formylbenzoic acid (150 mg, 1 mmol, 1 eq.), acetophenone (120 mg, 117 µl, 1 mmol, 1 eq.), 1mL of CTAB 2 % or Tween 80 2 %, and NaOH (100 µl of a 40 % solution, 40 mg NaOH, 1 mmol, 1 eq.). A crystallization from ethanol gave the pure compound.

From CTAB: Yield: 176.6 mg (70 %).

From Tween 80: Yield: 0 mg (0 %).

White solid, m.p. 227-228 °C (Lit. 227-229 °C[20]).

^{1}H NMR (600 MHz, DMSO-d_6) δ 8.17 (dd, *J* = 7.0, 1.6 Hz, 2H), 8.05 (d, *J* = 15.7 Hz, 1H), 8.00 (d, *J* = 3.2 Hz, 4H), 7.78 (d, *J* = 15.7 Hz, 1H), 7.69 (t, *J* = 7.4 Hz, 1H), 7.59 (t, *J* = 7.6 Hz, 2H).

^{13}C{1H} NMR (151 MHz, DMSO-d_6) δ 189.2, 166.9, 142.6, 138.8, 137.4, 133.4, 132.2, 129.7, 129.0, 128.9, 128.7, 124.2.

HRMS (ESI+) m/z: [M + H]+ Calcd for C16H13O3 253.0865; Found 253.0862.

Methyl (E)-4-(3-oxo-3-phenylprop-1-en-1-yl)benzoate (3n)[21]

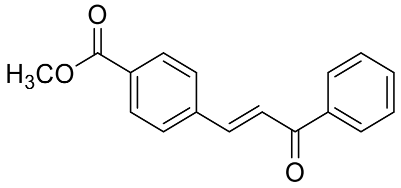

The reaction was performed following the general procedure by introducing in sequence in the vial: methyl 4-formylbenzoate acid (164 mg, 1 mmol, 1 eq.), acetophenone (120 mg, 117 µl, 1 mmol, 1 eq.), 1mL of CTAB 2 % or Tween 80 2 %, and NaOH (100 µl of a 40 % solution, 40 mg NaOH, 1 mmol, 1 eq.). The crude was further purified by column chromatography with a gradient of petroleum ether and ethyl acetate. A final crystallization from ethanol gave the pure compound.

From CTAB: Yield: 74.6 mg (28 %).

From Tween 80: Yield: 29.3 mg (11 %).

White solid, m.p. 125-126 °C (Lit. 126-128 °C[21]).

^{1}H NMR (600 MHz, DMSO-d_6) δ 7.35 (d, *J* = 7.1 Hz, 2H), 7.23 (s, 1H), 7.20 (m, 4H), 6.95 (d, *J* = 15.7 Hz, 1H), 6.86 (m, 1H), 6.76 (t, *J* = 7.7 Hz, 2H), 3.05 (s, 3H).

^{13}C{1H} NMR (151 MHz, DMSO-d_6) δ 189.2, 165.8, 142.4, 139.2, 137.3, 133.4, 130.8, 129.6, 129.1, 128.9, 128.7, 124.5, 52.3.

HRMS (ESI+) m/z: [M +H]+ Calcd for C17H15O3 267.1021; Found 267.1017.

(E)-1-phenyl-3-(pyridin-2-yl)prop-2-en-1-one (3o)[22]

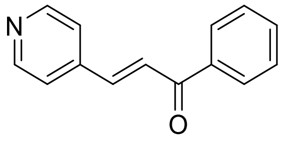

The reaction was performed following the general procedure by introducing in sequence in the vial: 4-pyridinecarboxaldehyde (107 mg, 94 µl, 1 mmol, 1 eq.), acetophenone (120 mg, 117 µl, 1 mmol, 1 eq.), 1mL of CTAB 2 % or Tween 80 2 %, and NaOH (100 µl of a 40 % solution, 40 mg NaOH, 1 mmol, 1 eq.). The crude was further purified by column chromatography with a gradient of petroleum ether and ethyl acetate. A final crystallization from ethanol gave the pure compound.

From CTAB: Yield: 0 mg (0 %).

From Tween 80: Yield: 16.1 mg (8 %).

Light brown crystals, m.p. 60-62 °C (Lit. 61-62 °C[23]).

^{1}H NMR (600 MHz, $CDCl_3$) δ 8.81 (d, *J* = 5.5 Hz, 2H), 8.04 (dd, *J* = 7.2, 0.8 Hz, 2H), 7.83 – 7.73 (m, 4H), 7.66 (td, *J* = 7.6, 1.1 Hz, 1H), 7.56 (m, 2H).

$^{13}C\{1H\}$ NMR (151 MHz, $CDCl_3$) δ 189.3, 147.3, 146.0, 139.8, 137.3, 133.9, 129.1, 128.8, 128.4, 123.4.

HRMS (ESI+) m/z: [M + H]+ Calcd for C14H12NO 210.0919; Found 210.0914.

(E)-1-phenyl-3-(pyridin-2-yl)prop-2-en-1-one (3p)[23]

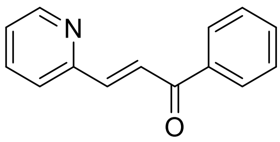

The reaction was performed following the general procedure by introducing in sequence in the vial: 2-pyridinecarboxaldehyde (107 mg, 95 µl, 1 mmol, 1 eq.), acetophenone (120 mg, 117 µl, 1 mmol, 1 eq.), 1mL of CTAB 2 % or Tween 80 2 %, and NaOH (100 µl of a 40 % solution, 40 mg NaOH, 1 mmol, 1 eq.). The crude was further purified by column chromatography with a gradient of petroleum ether and ethyl acetate. A final crystallization from ethanol gave the pure compound.

From CTAB: Yield: 29.1 mg (14 %).

From Tween 80: Yield: 98.1 mg (47 %).

Light brown crystals, m.p. 78-80 °C (Lit. 77-78 °C[23]).

^{1}H NMR (600 MHz, $CDCl_3$) δ 8.70 (m, 1H), 8.15 (d, *J* = 15.3 Hz, 1H), 8.11 (m, 2H), 7.77 (m, 2H), 7.60 (m, 1H), 7.51 (m, 3H), 7.32 (ddd, *J* = 7.6, 4.8, 1.1 Hz, 1H).

$^{13}C\{1H\}$ NMR (151 MHz, $CDCl_3$) δ 190.6, 153.3, 150.2, 142.7, 138.0, 137.3, 133.3, 128.9, 128.8, 125.9, 125.6, 124.6.

HRMS (ESI+) m/z: [M + H]+ Calcd for C14H12NO 210.0919; Found 210.0916.

(E)-1-phenyl-3-(thiophen-2-yl)prop-2-en-1-one (3q)[24, 25]

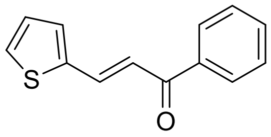

The reaction was performed following the general procedure by introducing in sequence in the vial: 2-thiophenecarboxaldehyde (112 mg, 93 µl, 1 mmol, 1 eq.), acetophenone (120 mg, 117 µl, 1 mmol, 1 eq.), 1mL of CTAB 2 % or Tween 80 2 %, and NaOH (100 µl of a 40 % solution, 40 mg NaOH, 1 mmol, 1 eq.). The crude was further purified by column chromatography with a gradient of petroleum ether and ethyl acetate. A final crystallization from ethanol gave the pure compound.

From CTAB: Yield: 175.1 mg (83 %).

From Tween 80: Yield: 146.8 mg (69 %).

Light yellow solid, m.p. 69-70 °C (Lit. 67-69 °C[24]).

^{1}H NMR (600 MHz, $CDCl_3$) δ 8.00 (dd, *J* = 8.3, 1.4 Hz, 2H), 7.95 (d, *J* = 15.3 Hz, 1H), 7.58 (ddt, *J* = 8.6, 6.8, 1.3 Hz, 1H), 7.50 (m, 2H), 7.37 (m, 2H), 7.33 (d, *J* = 15.3 Hz, 1H), 7.08 (dd, *J* = 5.0, 3.6 Hz, 1H).

$^{13}C\{1H\}$ NMR (151 MHz, $CDCl_3$) δ 190.0, 140.5, 138.2, 137.3, 132.9, 132.2, 128.9, 128.7, 128.5, 128.5, 120.9.

HRMS (ESI+) m/z: [M + H]+ Calcd for C13H11OS 215.0531; Found 215.0528.

(E)-3-(furan-2-yl)-1-phenylprop-2-en-1-one (**3r**)[25, 26]

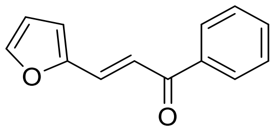

The reaction was performed following the general procedure by introducing in sequence in the vial: furfural (96.1 mg, 83 µl, 1 mmol, 1 eq.), acetophenone (120 mg, 117 µl, 1 mmol, 1 eq.), 1mL of CTAB 2 % or Tween 80 2 %, and NaOH (100 µl of a 40 % solution, 40 mg NaOH, 1 mmol, 1 eq.). The crude was further purified by column chromatography with a gradient of petroleum ether and ethyl acetate.

From CTAB: Yield: 107.4 mg (54 %).

From Tween 80: Yield: 97.1 mg (49 %).

Viscous reddish oil, which solidifies on standing after a few days, m.p. 39-42 °C (Lit. 44-45 °C[27]).

^{1}H NMR (600 MHz, $CDCl_3$) δ 8.03 (m, 2H), 7.59 (m, 2H), 7.50 (m, 4H), 6.72 (dd, *J* = 3.5, 0.7 Hz, 1H), 6.51 (dd, *J* = 3.4, 1.8 Hz, 1H).

$^{13}C\{1H\}$ NMR (151 MHz, $CDCl_3$) δ 190.0, 151.8, 145.1, 138.3, 132.9, 130.8, 128.7, 128.6, 119.4, 116.4, 112.8.

HRMS (ESI+) m/z: [M + H]+ Calcd for C13H11O2 199.0759; Found 215.0528.

(E)-1-phenyl-3-(1H-pyrrol-2-yl)prop-2-en-1-one (**3s**)[24, 25]

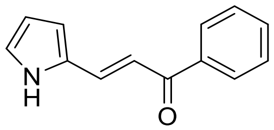

The reaction was performed following the general procedure by introducing in sequence in the vial: 2-pyrrolecarboxaldehyde (95.1 mg, 1 mmol, 1 eq.), acetophenone (120 mg, 117 µl, 1 mmol, 1 eq.), 1mL of CTAB 2 % or Tween 80 2 %, and NaOH (100 µl of a 40 % solution, 40 mg NaOH, 1 mmol, 1 eq.). The crude was further purified by column chromatography with a gradient of petroleum ether and ethyl acetate. A final crystallization from ethanol gave the pure compound.

From CTAB: Yield: 61.9 mg (31 %).

From Tween 80: Yield: 140.0 mg (71 %).

Light green solid, m.p. 135-137 °C (Lit. 134-137 °C[24]).

^{1}H NMR (600 MHz, $CDCl_3$) δ 9.01 (s, 1H), 7.98 (dd, *J* = 8.4, 1.3 Hz, 1H), 7.75 (d, *J* = 15.6 Hz, 1H), 7.56 (ddt, *J* = 8.0, 6.8, 1.3 Hz, 1H), 7.48 (dd, *J* = 8.2, 6.9 Hz, 2H), 7.16 (d, *J* = 15.5 Hz, 1H), 7.00 (td, *J* = 2.7, 1.3 Hz, 1H), 6.72 (ddd, *J* = 3.8, 2.5, 1.4 Hz, 1H), 6.34 (dt, *J* = 3.7, 2.5 Hz, 1H).

^{13}C{1H} NMR (151 MHz, $CDCl_3$) δ 190.7, 138.8, 134.8, 132.5, 129.4, 128.7, 128.4, 123.3 115.9, 115.5, 111.7.

HRMS (ESI+) m/z: [M + H]+ Calcd for C13H12NO 198.0919; Found 198.0914.

SERIES 4

1,3,5-triphenylpentane-1,5-dione (4a)[28]

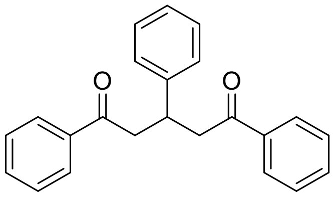

The product was isolated from the synthesis of **3a**. A final crystallization from ethanol gave the pure compound.

From CTAB: Yield: 32.8 mg (10 %).

White solid, m.p. 81-83 °C (Lit. 82-84 °C[29]).

^{1}H NMR (600 MHz, $CDCl_3$) δ 7.95 (m, 4H), 7.54 (m, 2H), 7.44 (dd, *J* = 8.3, 7.4 Hz, 4H), 7.28 (m, 4H), 7.19 (m, 1H), 4.07 (p, *J* = 7.0 Hz, 1H), 3.49 (dd, *J* = 16.6, 7.0 Hz, 2H), 3.36 (dd, *J* = 16.6, 7.1 Hz, 2H).

.

^{13}C{1H} NMR (151 MHz, $CDCl_3$) δ 198.7, 144.0, 137.0, 133.2, 128.8, 128.7, 128.3, 127.6, 126.8, 45.05, 37.30.

HRMS (ESI+) m/z: [M + H]+ Calcd for C23H21O2 329.1536; Found 329.1535.

1,5-diphenyl-3-(p-tolyl)pentane-1,5-dione (4c)[30]

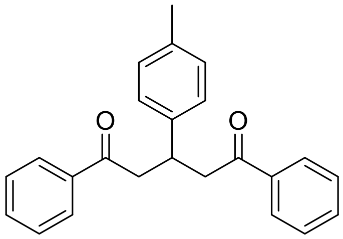

The product was isolated from the synthesis of **3c**. A final crystallization from ethanol gave the pure compound.

From CTAB: Yield: 25.7 mg (8 %).

White solid, m.p. 101-103 °C (Lit. 102-104 °C[31]).

^{1}H NMR (600 MHz, $CDCl_3$) δ 7.95 (dd, J = 8.5, 1.3 Hz, 4H), 7.54 (m, 2H), 7.44 (m, 4H), 7.17 (d, J = 8.1 Hz, 2H), 7.08 (dd, J = 8.4, 0.7 Hz, 2H), 4.03 (p, J = 7.0 Hz, 1H), 3.47 (dd, J = 16.6, 7.0 Hz, 2H), 3.33 (dd, J = 16.6, 7.1 Hz, 2H), 2.28 (d, J = 0.8 Hz, 3H).

$^{13}C\{1H\}$ NMR (151 MHz, $CDCl_3$) δ 198.8, 140.9, 137.1, 136.3, 133.2, 129.5, 128.7, 128.3, 127.4, 45.2, 37.0, 21.2.

HRMS (ESI+) m/z: [M + H]+ Calcd for C24H23O2 343.1693; Found 343.1685.

3-(4-methoxyphenyl)-1,5-diphenylpentane-1,5-dione (**4d**)[30]

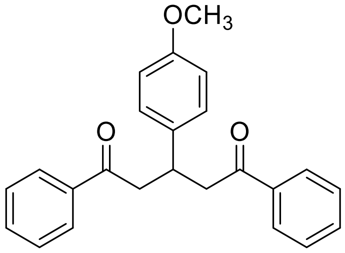

The product was isolated from the synthesis of **3d**. A final crystallization from ethanol gave the pure compound.

From CTAB: Yield: 31.2 mg (9 %).

Yellow solid, m.p. 101-102 °C (Lit. 100-102 °C[31])

^{1}H NMR (600 MHz, $CDCl_3$) δ 7.95 (dd, *J* = 8.4, 1.3 Hz, 4H), 7.54 (m, 2H), 7.45 (m, 14H), 7.20 (d, *J* = 8.7 Hz, 2H), 6.81 (d, *J* = 8.7 Hz, 2H), 4.02 (p, *J* = 7.0 Hz, 1H), 3.75 (s, 3H), 3.47 (dd, *J* = 16.5, 7.0 Hz, 2H), 3.32 (dd, *J* = 16.5, 7.1 Hz, 2H).

$^{13}C\{1H\}$ NMR (151 MHz, $CDCl_3$) δ 198.9, 158.3, 137.0, 135.9, 133.2, 128.7, 128.5, 128.3, 114.1, 55.3, 45.3, 36.6.

HRMS (ESI+) m/z: [M + H]+ Calcd for C24H23O3 329.1642; Found 359.1641.

3-(2-bromophenyl)-1,5-diphenylpentane-1,5-dione (**4j**)[32]

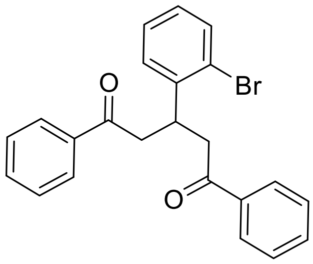

The product was isolated from the synthesis of **3j**. A final crystallization from ethanol gave the pure compound.

From CTAB: Yield: 49.7 mg (12 %).

From CTAB at 45 °C: Yield: 78.7 mg (19 %).

White solid, m.p. 121-123 °C (Lit. 120-122 °C[33]).

^{1}H NMR (600 MHz, CDCl3) δ 7.97 (dd, *J* = 8.4, 1.4 Hz, 4H), 7.59 – 7.53 (m, 1H), 7.45 (dd, *J* = 8.2, 7.3 Hz, 3H), 7.33 (dd, *J* = 7.8, 1.7 Hz, 4H), 7.24 (td, *J* = 7.6, 1.3 Hz, 1H), 7.05 (ddd, *J* = 8.0, 7.3, 1.7 Hz, 1H), 4.54 (t, *J* = 7.0 Hz, 1H), 3.53 (dd, *J* = 16.8, 7.3 Hz, 2H), 3.44 (dd, *J* = 16.9, 6.7 Hz, 2H).

^{13}C{1H} NMR (151 MHz, CDCl3) δ 198.6, 142.6, 136.9, 133.5, 133.3, 128.7, 128.3, 128.2, 127.9, 124.6, 43.3, 36.3.

HRMS (ESI+) m/z: [M + H]+ Calcd for C23H20BrO2 407.0641; Found 407.0644.

3-(4-nitrophenyl)-1,5-diphenylpentane-1,5-dione (4k)[34]

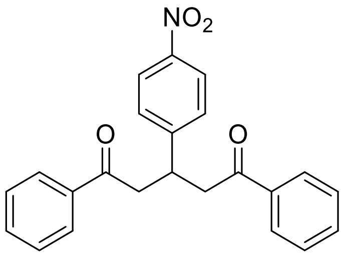

The product was isolated from the synthesis of **3k**. A final crystallization from ethanol gave the pure compound.

From CTAB at 25°C: Yield: 44.8 mg (12 %).

From CTAB at 45°C: Yield: 55.0 mg (15 %).

Yellow solid, m.p. 109-111 °C (Lit. 110-112 °C[34]).

^{1}H NMR (600 MHz, CDCl3) δ 8.15 (d, *J* = 8.8 Hz, 1H), 7.93 (dd, *J* = 8.5, 1.3 Hz, 2H), 7.57 (m, 1H), 7.47 (m, 3H), 4.21 (p, *J* = 7.0 Hz, 1H), 3.55 (dd, *J* = 17.2, 6.5 Hz, 1H), 3.40 (dd, *J* = 17.1, 7.5 Hz, 1H).

^{13}C{1H} NMR (151 MHz, CDCl3) δ 197.7, 151.8, 146.8, 136.7, 133.6, 128.9, 128.70, 128.22, 124.0, 44.4, 36.8.

HRMS (ESI+) m/z: [M + H]+ Calcd for C23H20NO4 374.1387; Found 374.1384.

1,5-diphenyl-3-(pyridin-4-yl)pentane-1,5-dione (4o)[35]

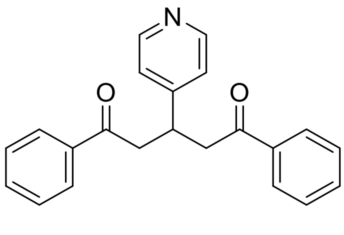

The product was isolated from the synthesis of **3o**. A final crystallization from ethanol gave the pure compound.

From CTAB: Yield: 74.4 mg (23 %).

From Tween 80: Yield: 68.2 mg (21 %).

Light pink solid, m.p. 124-125 °C (Lit. 125-126 °C[36]).

^1H NMR (600 MHz, $CDCl_3$) δ 8.48 (d, *J* = 6.1 Hz, 2H), 7.92 (d, *J* = 7.3 Hz, 4H), 7.55 (td, *J* = 7.5, 1.1 Hz, 2H), 7.44 (t, *J* = 7.7 Hz, 4H), 7.24 (d, *J* = 6.1 Hz, 2H), 4.08 (p, *J* = 6.9 Hz, 1H), 3.50 (dd, *J* = 17.2, 6.6 Hz, 2H), 3.38 (dd, *J* = 17.2, 7.3 Hz, 2H).

$^{13}C\{1H\}$ NMR (151 MHz, $CDCl_3$) δ 197.7, 153.2, 150.0, 136.6, 133.5, 128.8, 128.1, 123.1, 43.9, 36.2.

HRMS (ESI+) m/z: [M + H]+ Calcd for C22H20NO2 330.1489; Found 330.1487

1,5-diphenyl-3-(pyridin-2-yl)pentane-1,5-dione (4p)[37]

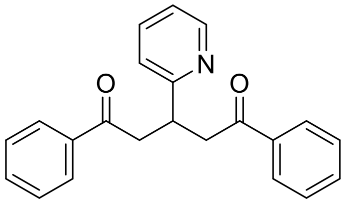

The product was isolated from the synthesis of **3p**. A final crystallization from ethanol gave the pure compound.

From CTAB: Yield: 40.5 mg (12 %).

From Tween 80: Yield: 32.6 mg (10 %).

White solid, m.p. 119-121 °C (Lit. 120-121 °C[38]).

^1H NMR (600 MHz, $CDCl_3$) δ 8.48 (ddd, *J* = 4.9, 1.8, 0.9 Hz, 1H), 7.95 (d, *J* = 7.2 Hz, 2H), 7.60 (t, *J* = 7.6 Hz, 1H), 7.56 – 7.49 (m, 2H), 7.47 – 7.37 (m, 4H), 7.09 (dd, *J* = 7.8, 4.8 Hz, 1H), 4.24 (tt, *J* = 7.8, 5.9 Hz, 1H), 3.68 (dd, *J* = 17.2, 7.8 Hz, 2H), 3.47 (dd, *J* = 17.4, 5.9 Hz, 2H).

$^{13}C\{1H\}$ NMR (151 MHz, $CDCl_3$) δ 198.8, 163.1, 149.3, 137.1, 136.5, 133.2, 128.7, 128.3, 124.3, 121.7, 43.7, 38.3.

HRMS (ESI+) m/z: [M + H]+ Calcd for C22H20NO2 330.1489; Found 330.1487.

1,5-diphenyl-3-(thiophen-2-yl)pentane-1,5-dione (4q)[39]

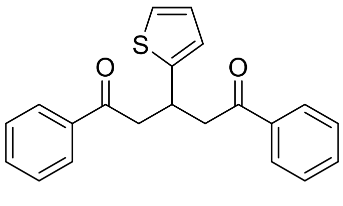

The product was isolated from the synthesis of **3q**. A final crystallization from ethanol gave the pure compound.

From CTAB: Yield: 40.8 mg (12 %).

White-off solid, m.p. 90-92 °C (Lit. 89-91 °C[31])

^1H NMR (600 MHz, $CDCl_3$) δ 7.97 (dd, *J* = 8.5, 1.2 Hz, 4H), 7.56 (m, 2H), 7.45 (m, 4H), 7.11 (dd, *J* = 5.1, 1.3 Hz, 1H), 6.90 (ddd, *J* = 3.5, 1.3, 0.6 Hz, 1H), 6.88 (dd, *J* = 5.0, 3.5 Hz, 1H), 4.44 (p, *J* = 6.8 Hz, 1H), 3.53 (dd, *J* = 16.9, 6.8 Hz, 2H), 3.44 (dd, *J* = 16.9, 6.9 Hz, 2H).

$^{13}C\{^1H\}$ NMR (151 MHz, $CDCl_3$) δ 198.2, 147.6, 136.9, 133.3, 128.7, 128.2, 126.8, 124.4, 123.4, 45.7, 32.5.

HRMS (ESI+) m/z: [M + H]+ Calcd for C21H19O2S 335.1100; Found 335.1098.

SERIES 6

(E)-3-phenyl-1-(p-tolyl)prop-2-en-1-one (6b)[40, 41]

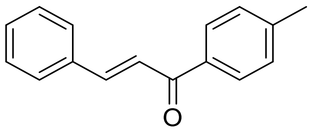

The reaction was performed following the general procedure by introducing in sequence in the vial: benzaldehyde (106 mg, 102 µl, 1 mmol, 1 eq.), 4-methylacetophenone (134 mg, 134 µl, 1 mmol, 1 eq.), 1mL of CTAB 2 % or Tween 80 2 %, and NaOH (100 µl of a 40 % solution, 40 mg NaOH, 1 mmol, 1 eq.). The crude was further purified by column chromatography with a gradient of petroleum ether and ethyl acetate. A final crystallization from ethanol gave the pure compound.

From CTAB: Yield: 186.1 mg (84 %).

From Tween 80 at 25°C: Yield: 86.7 mg (39 %).

From Tween 80 at 45°C: Yield: 177.8 mg (80 %).

Yellow solid, m.p. 73-75 °C (Lit. 74-75 °C[16]).

^1H NMR (600 MHz, $CDCl_3$) δ 7.94 (d, J = 8.2 Hz, 2H), 7.81 (d, J = 15.7 Hz, 1H), 7.65 (m, 2H), 7.54 (d, J = 15.7 Hz, 1H), 7.42 (m, 3H), 7.31 (m, 2H), 2.44 (s, 3H).

$^{13}C\{^1H\}$ NMR (151 MHz, $CDCl_3$) δ 190.2, 144.6, 143.8, 135.8, 135.2, 130.6, 129.5, 129.1, 128.8, 128.6, 122.3, 21.8.

HRMS (ESI+) m/z: [M + H]+ Calcd for C16H15O 223.1117; Found 223.1115.

(E)-1-(4-methoxyphenyl)-3-phenylprop-2-en-1-one (6c)[42, 43]

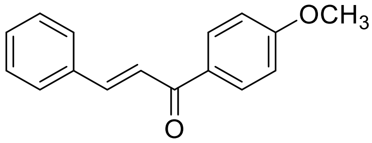

The reaction was performed following the general procedure by introducing in sequence in the vial: benzaldehyde (106 mg, 102 µl, 1 mmol, 1 eq.), 4-methoxyacetophenone (150 mg, 1 mmol, 1 eq.), 1mL of CTAB 2 % or Tween 80 2 %, and NaOH (100 µl of a 40 % solution, 40 mg NaOH, 1 mmol, 1 eq.). The crude was further purified by column chromatography with a gradient of petroleum ether and ethyl acetate. A final crystallization from ethanol gave the pure compound.

From CTAB: Yield: 162.0 mg (68 %).

From Tween 80 at 25°C: Yield: 107.2 mg (45 %).

From Tween 80 at 25°C: Yield: 184.9 mg (77 %).

Light yellow solid, m.p. 103-105 °C (Lit. 104-106 °C[16]).

^{1}H NMR (600 MHz, $CDCl_3$) δ 8.05 (d, J = 8.9 Hz, 2H), 7.81 (d, J = 15.6 Hz, 1H), 7.65 (m, 2H), 7.55 (d, J = 15.6 Hz, 1H), 7.42 (m, 3H), 6.99 (d, J = 8.8 Hz, 2H), 3.90 (s, 3H).

^{13}C{1H} NMR (151 MHz, $CDCl_3$) δ 188.9, 163.6, 144.1, 135.2, 131.3, 131.0, 130.5, 129.1, 128.5, 122.0, 114.0, 55.7.

HRMS (ESI+) m/z: [M+ H]+ Calcd for C16H15O2 239.1067; Found 239.1067.

(E)-1-(2-hydroxyphenyl)-3-phenylprop-2-en-1-one (6d)[2]

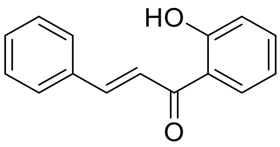

The reaction was performed following the general procedure by introducing in sequence in the vial: benzaldehyde (106 mg, 102 µl, 1 mmol, 1 eq.), 2-hydroxyacetophenone (136 mg, 1 mmol, 1 eq.), 1mL of CTAB 2 % or Tween 80 2 %, and NaOH (200 µl of a 40 % solution, 80 mg NaOH, 1 mmol, 1 eq.). The crude was further purified by column chromatography with a gradient of petroleum ether and ethyl acetate. A final crystallization from ethanol gave the pure compound.

From CTAB: Yield: 33.6 mg (15 %).

From Tween 80 at 25°C: Yield: 184.6 mg (82 %).

Yellow solid, m.p. 80-81 °C (Lit. 79-80 °C[44]).

^{1}H NMR (600 MHz, $CDCl_3$) δ 7.94 (m, 1H), 7.68 (m, 1H), 7.51 (ddd, J = 8.7, 7.2, 1.6 Hz, 1H), 7.45 (m, 2H), 7.04 (dd, J = 8.4, 1.2 Hz, 1H), 6.96 (ddd, J = 8.2, 7.2, 1.2 Hz, 1H).

^{13}C{1H} NMR (151 MHz, $CDCl_3$) δ 193.9, 163.8, 145.6, 136.6, 134.7, 131.1, 129.8, 129.2, 128.8, 120.3, 120.2, 119.0, 118.8.

HRMS (ESI+) m/z: [M + H]+ Calcd for C15H13O2 225.0910; Found 225.0910.

(E)-1-(4-bromophenyl)-3-phenylprop-2-en-1-one (6e)[14]

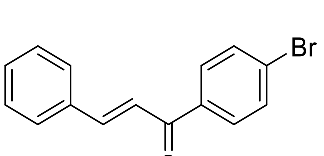

The reaction was performed following the general procedure by introducing in sequence in the vial: benzaldehyde (106 mg, 102 µl, 1 mmol, 1 eq.), 4-bromoacetophenone (199 mg, 1 mmol, 1 eq.), 1mL of CTAB 2 % or Tween 80 2 %, and NaOH (100 µl of a 4 0% solution, 40 mg NaOH, 1 mmol, 1 eq.). The crude was further purified by column chromatography with a gradient of petroleum ether and ethyl acetate. A final crystallization from ethanol gave the pure compound.

From CTAB: Yield: 202.2 mg (70 %).

From Tween 80 at 25°C: Yield: 221.1 mg (77 %).

White solid, m.p. 98-100 °C (Lit. 96-98 °C[14]).

^{1}H NMR (600 MHz, $CDCl_3$) δ 7.89 (d, *J* = 8.7 Hz, 2H), 7.82 (d, *J* = 15.7 Hz, 1H), 7.65 (m, 4H), 7.48 (d, *J* = 15.7 Hz, 1H), 7.43 (m, 3H).

^{13}C{1H} NMR (151 MHz, $CDCl_3$) δ 189.6, 145.6, 137.1, 134.8, 132.1, 130.9, 130.2, 129.2, 128.7, 128.1, 121.6.

HRMS (ESI+) m/z: [M + H]+ Calcd for C15H12BrO 287.0066; Found 287.0068.

(E)-1-(3-bromophenyl)-3-phenylprop-2-en-1-one (6f)[45]

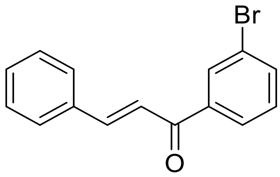

The reaction was performed following the general procedure by introducing in sequence in the vial: benzaldehyde (106 mg, 102 µl, 1 mmol, 1 eq.), 3-bromoacetophenone (199 mg, 132 µl, 1 mmol, 1 eq.), 1mL of CTAB 2 % or Tween 80 2 %, and NaOH (100 µl of a 40 % solution, 40 mg NaOH, 1 mmol, 1 eq.). The crude was further purified by column chromatography with a gradient of petroleum ether and ethyl acetate. A final crystallization from ethanol gave the pure compound.

From CTAB: Yield: 191.8 mg (67 %).

From Tween 80 at 25°C: Yield: 244.1 mg (85 %).

White solid, m.p. 92-94 °C (Lit. 92-95 °C[14]).

^{1}H NMR (600 MHz, $CDCl_3$) δ 8.14 (t, *J* = 1.8 Hz, 1H), 7.94 (ddd, *J* = 7.7, 1.6, 1.0 Hz, 1H), 7.83 (d, *J* = 15.7 Hz, 1H), 7.71 (ddd, *J* = 8.0, 2.0, 1.0 Hz, 1H), 7.66 (m, 2H), 7.47 (d, *J* = 15.7 Hz, 1H), 7.44 (m, 2H), 7.39 (t, *J* = 7.8 Hz, 1H).

^{13}C{1H} NMR (151 MHz, $CDCl_3$) δ 189.2, 145.9, 140.2, 135.8, 134.8, 131.7, 131.0, 130.4, 129.2, 128.7, 127.2, 123.12 121.6.

HRMS (ESI+) m/z: [M + H]+ Calcd for C15H12BrO 287.0066; Found 287.0067.

(E)-1-(2-bromophenyl)-3-phenylprop-2-en-1-one (6g)[46]

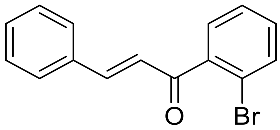

The reaction was performed following the general procedure by introducing in sequence in the vial: benzaldehyde (106 mg, 102 µl, 1 mmol, 1 eq.), 2-bromoacetophenone (199 mg, 135 µl, 1 mmol, 1 eq.), 1 mL of CTAB 2 % or Tween 80 2 %, and NaOH (100 µl of a 40 % solution, 40 mg NaOH, 1 mmol, 1 eq.). The crude was further purified by column chromatography with a gradient of petroleum ether and ethyl acetate. A final crystallization from ethanol gave the pure compound.

From CTAB: Yield: 229.7 mg (80 %).

From Tween 80 at 25°C: Yield: 268.5 mg (94 %).

Yellow oil.

^{1}H NMR (600 MHz, $CDCl_3$) δ 7.65 (d, J = 8.0 Hz, 1H), 7.56 (m, 2H), 7.42 (m, 6H), 7.34 (m, 1H), 7.10 (d, J = 16.1 Hz, 1H).

^{13}C{1H} NMR (151 MHz, $CDCl_3$) δ 194.9, 146.8, 141.3, 134.5, 133.6, 131.5, 131.1, 129.3, 129.1, 128.7, 127.5, 126.3, 119.6.

HRMS (ESI+) m/z: [M + H]+ Calcd for C15H12BrO 287.0066; Found 287.0068.

(E)-1-(4-nitrophenyl)-3-phenylprop-2-en-1-one (6h)[47]

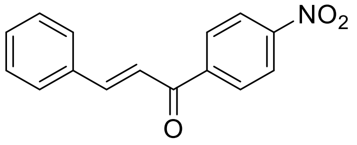

The reaction was performed following the general procedure by introducing in sequence in the vial: benzaldehyde (106 mg, 102 µl, 1 mmol, 1 eq.), 4-nitroacetophenone (163 mg, 1 mmol, 1 eq.), 1 mL of CTAB 2 % or Tween 80 2 %, and NaOH (100 µl of a 40 % solution, 40 mg NaOH, 1 mmol, 1 eq.). The crude was further purified by column chromatography with a gradient of petroleum ether and ethyl acetate. A final crystallization from ethanol gave the pure compound.

From CTAB: Yield: 65.8 mg (26 %).

From Tween 80 at 25°C: Yield: 146.9 mg (58 %).

Yellow solid, m.p. 145-147 °C (Lit. 122-124 °C[47], 144-146 °C[21]).

^{1}H NMR (600 MHz, $CDCl_3$) δ 8.36 (d, J = 8.9 Hz, 2H), 8.15 (d, J = 8.9 Hz, 2H), 7.85 (d, J = 15.7 Hz, 1H), 7.67 (m, 2H), 7.49 (d, J = 15.7 Hz, 1H), 7.46 (m, 3H).

^{13}C{1H} NMR (151 MHz, $CDCl_3$) δ 189.2, 150.2, 147.0, 143.2, 134.4, 131.4, 129.6, 129.3, 128.9, 124.0, 121.5.

HRMS (ESI+) m/z: [M + H]+ Calcd for C15H12NO3 254.0812; Found 254.0812.

4-cinnamoylbenzoic acid (6i)[20]

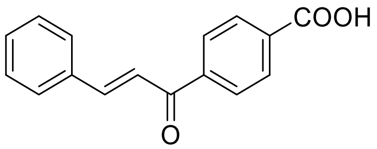

The reaction was performed following the general procedure by introducing in sequence in the vial: benzaldehyde (106 mg, 102 µl, 1 mmol, 1 eq.), 4-acetylbenzoic acid (164 mg, 1 mmol, 1 eq.), 1 mL of CTAB 2 % or Tween 80 2 %, and NaOH (100 µl of a 40 % solution, 40 mg NaOH, 1 mmol, 1 eq.). A crystallization from ethanol gave the pure compound.

From CTAB: Yield: 138.7 mg (55 %).

From Tween 80: Yield: 171.5 mg (68 %).

White solid, m.p. 219-221 °C (Lit. 217-220 °C[20]).

^{1}H NMR (600 MHz, DMSO-d_6) δ 8.12 (d, J = 8.4 Hz, 2H), 8.02 (d, J = 8.3 Hz, 2H), 7.95 (d, J = 15.7 Hz, 1H), 7.92 – 7.88 (m, 2H), 7.75 (d, J = 15.6 Hz, 1H), 7.47 (dd, J = 5.0, 1.9 Hz, 3H).

^{13}C{1H} NMR (151 MHz, DMSO-d_6) δ 189.0, 166.8, 144.8, 140.7, 134.7, 134.5, 130.9, 129.6, 129.1, 129.0, 128.7, 122.0.

HRMS (ESI+) m/z: [M+ H]+ Calcd for C16H13O3 253.0859; Found 253.0854.

Methyl 4-cinnamoylbenzoate (6j)[48, 49]

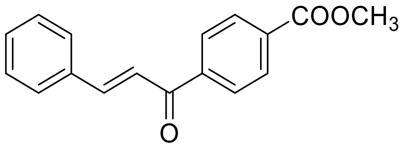

The reaction was performed following the general procedure by introducing in sequence in the vial: benzaldehyde (106 mg, 102 µl, 1 mmol, 1 eq.), methyl 4-acetylbenzoate acid (178 mg, 1 mmol, 1 eq.), 1 mL of CTAB 2 % or Tween 80 2 %, and NaOH (100 µl of a 40 % solution, 40 mg NaOH, 1 mmol, 1 eq.). The crude was further purified by column chromatography with a gradient of petroleum ether and ethyl acetate. A final crystallization from ethanol gave the pure compound.

From CTAB: Yield: 39.9 mg (15 %).

From Tween 80: Yield: 50.6 mg (19 %).

White solid, m.p. 123-124 °C (Lit. 123-125 °C[49]).

^{1}H NMR (600 MHz, $CDCl_3$) δ 8.17 (m, 2H), 8.06 (m, 2H), 7.83 (d, J = 15.7 Hz, 1H), 7.66 (ddd, J = 5.4, 3.1, 1.3 Hz, 2H), 7.51 (d, J = 15.7 Hz, 1H), 7.44 (m, 3H), 3.97 (s, 3H).

^{13}C{1H} NMR (151 MHz, $CDCl_3$) δ 190.3, 166.5, 146.0, 141.8, 134.8, 133.7, 131.0, 130.0, 129.2, 128.73 128.3, 122.0, 52.6.

HRMS (ESI+) m/z: [M + H]+ Calcd for C17H15O3 267.1016; Found 267.1013.

(E)-3-phenyl-1-(pyridin-4-yl)prop-2-en-1-one (6k)[50]

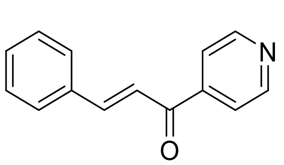

The reaction was performed following the general procedure by introducing in sequence in the vial: benzaldehyde (106 mg, 102 µl, 1 mmol, 1 eq.), 4-acetylpyridine (121 mg, 111 µl, 1 mmol, 1 eq.), 1 mL of CTAB 2 % or Tween 80 2 %, and NaOH (100 µl of a 40 % solution, 40 mg NaOH, 1 mmol, 1 eq.). The crude was further purified by column chromatography with a gradient of petroleum ether and ethyl acetate. A final crystallization from ethanol gave the pure compound.

From CTAB: Yield: 0 mg (0%).

From Tween 80: Yield: 50.8 mg (24%).

Light yellow-brown solid, m.p. 89-91 °C (Lit. 172-174 °C[50], 89.0-89.2 °C[51]).

^{1}H NMR (600 MHz, $CDCl_3$) δ 8.82 (d, J = 4.7 Hz, 2H), 7.80 (d, J = 0.7 Hz, 1H), 7.76 (d, J = 6.1 Hz, 2H), 7.64 (m, 2H), 7.42 (m, 4H).

$^{13}C\{1H\}$ NMR (151 MHz, $CDCl_3$) δ 190.0, 150.9, 147.0, 144.5, 134.4, 131.3, 129.2, 128.8, 121.6, 121.3.

HRMS (ESI+) m/z: [M + H]+ Calcd for C14H12NO 210.0913; Found 210.0913.

(E)-3-phenyl-1-(pyridin-3-yl)prop-2-en-1-one (6l)[14]

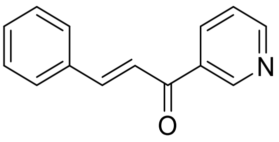

The reaction was performed following the general procedure by introducing in sequence in the vial: benzaldehyde (106 mg, 102 µl, 1 mmol, 1 eq.), 3-acetylpyridine (121 mg, 110 µl, 1 mmol, 1 eq.), 1 mL of CTAB 2 % or Tween 80 2 %, and NaOH (100 µl of a 40 % solution, 40 mg NaOH, 1 mmol, 1 eq.). The crude was further purified by column chromatography with a gradient of petroleum ether and ethyl acetate. A final crystallization from ethanol gave the pure compound.

From CTAB: Yield: 0 mg (0 %).

From Tween 80: Yield: 97.1 mg (46 %).

Light yellow-brown solid, m.p. 72-73 °C (Lit. 72-74°C[52]).

^{1}H NMR (600 MHz, $CDCl_3$) δ 9.24 (d, J = 1.0 Hz, 1H), 8.81 (d, J = 4.8 Hz, 1H), 8.29 (dd, J = 7.9, 1.5 Hz, 1H), 7.85 (d, J = 15.7 Hz, 1H), 7.66 (m, 2H), 7.49 (d, J = 15.7 Hz, 1H), 7.44 (m, 5H).

$^{13}C\{1H\}$ NMR (151 MHz, $CDCl_3$) δ 189.3, 153.3, 149.9, 146.2, 136.1, 134.6, 133.7, 131.2, 129.2, 128.8, 123.8, 121.5.

HRMS (ESI+) m/z: [M + H]+ Calcd for C14H12NO 210.0913; Found 210.0913.

(E)-3-phenyl-1-(pyridin-2-yl)prop-2-en-1-one (6m)[52]

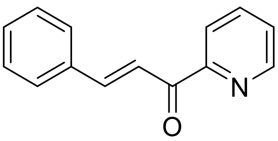

The reaction was performed following the general procedure by introducing in sequence in the vial: benzaldehyde (106 mg, 102 µl, 1 mmol, 1 eq.), 2-acetylpyridine (121 mg, 112 µl, 1 mmol, 1 eq.), 1 mL of CTAB 2 % or Tween 80 2%, and NaOH (100 µl of a 40 % solution, 40 mg NaOH, 1 mmol, 1 eq.). The crude was further purified by column chromatography with a gradient of petroleum ether and ethyl acetate. A final crystallization from ethanol gave the pure compound.

From CTAB: Yield: 44.8 mg (21 %).

From Tween 80: Yield: 175.4 mg (84 %).

Dark green solid, m.p. 72-74 °C (Lit. 72-74 °C[52], 74-75 °C).

^{1}H NMR (600 MHz, $CDCl_3$) δ 8.75 (ddd, J = 4.7, 1.7, 0.9 Hz, 1H), 8.31 (d, J = 16.0 Hz, 1H), 8.19 (dt, J = 7.8, 1.1 Hz, 1H), 7.95 (dd, J = 16.1, 0.5 Hz, 1H), 7.88 (td, J = 7.7, 1.7 Hz, 1H), 7.73 (m, 2H), 7.49 (ddd, J = 7.5, 4.7, 1.3 Hz, 1H), 7.42 (m, 3H).

$^{13}C\{1H\}$ NMR (151 MHz, $CDCl_3$) δ 189 7, 154.4, 149.0, 145.0, 137.2, 135.3, 130.7, 129.0, 129.0, 127.0, 123.1, 121.0.

HRMS (ESI+) m/z: [M+ H]+ Calcd for C14H12NO 210.0913; Found 210.0914.

(E)-3-phenyl-1-(thiophen-2-yl)prop-2-en-1-one (6n)[53]

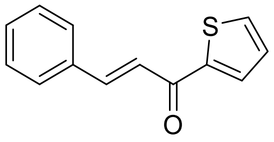

The reaction was performed following the general procedure by introducing in sequence in the vial: benzaldehyde (106 mg, 102 µl, 1 mmol, 1 eq.), 2-acetylthiophene (126 mg, 108 µl, 1 mmol, 1 eq.), 1 mL of CTAB 2 % or Tween 80 2 %, and NaOH (100 µl of a 40 % solution, 40 mg NaOH, 1 mmol, 1 eq.). The crude was further purified by column chromatography with a gradient of petroleum ether and ethyl acetate. A final crystallization from ethanol gave the pure compound.

From CTAB: Yield: 64.1 mg (30 %).

From Tween 80: Yield: 169.9 mg (79 %).

Yellow solid, m.p. 73-75 °C (Lit. 55 °C[53], 73-74 °C[27]).

^{1}H NMR (600 MHz, $CDCl_3$) δ 7.87 (m, 2H), 7.69 (m, 1H), 7.65 (m, 2H), 7.43 (m, 4H), 7.19 (ddd, J = 5.0, 3.8, 0.6 Hz, 1H).

$^{13}C\{1H\}$ NMR (151 MHz, $CDCl_3$) δ 182.2, 145.7, 144.2, 134.9, 134.1, 132.0, 130.8, 129.1, 128.6, 128.4, 121.8.

HRMS (ESI+) m/z: [M + H]+ Calcd for C13H11OS 215.0525; Found 215.0526.

(E)-1-(furan-2-yl)-3-phenylprop-2-en-1-one (6o)[54]

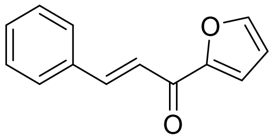

The reaction was performed following the general procedure by introducing in sequence in the vial: benzaldehyde (106 mg, 102 µl, 1 mmol, 1 eq.), 2-acetylfuran (110 mg, 100 µl, 1 mmol, 1 eq.), 1 mL of CTAB 2 % or Tween 80 2 %, and NaOH (100 µl of a 40 % solution, 40 mg NaOH, 1 mmol, 1 eq.). The crude was further purified by column chromatography with a gradient of petroleum ether and ethyl acetate. A final crystallization from ethanol gave the pure compound.

From CTAB: Yield: 75.7 mg (38 %).

From Tween 80 at 25°C: Yield: 117.3 mg (59 %).

White-off solid, m.p. 88-89 °C (Lit. 87-89 °C[54, 55], 89-90 °C[27]).

^{1}H NMR (600 MHz, $CDCl_3$) δ 7.89 (d, J = 15.8 Hz, 1H), 7.68 – 7.64 (m, 3H), 7.46 (d, J = 15.8 Hz, 1H), 7.44 – 7.41 (m, 3H), 7.34 (dd, J = 3.6, 0.8 Hz, 1H), 6.60 (dd, J = 3.6, 1.7 Hz, 1H).

^{13}C{1H} NMR (151 MHz, $CDCl_3$) δ 178.2, 153.9, 146.7, 144.2, 134.9, 130.8, 129.1, 128.7, 121.3, 117.7, 112.7.

HRMS (ESI+) m/z: [M + H]+ Calcd for C13H11O2 199.0754 u.ma.; Found 199.0755.

(E)-3-phenyl-1-(1H-pyrrol-2-yl)prop-2-en-1-one (6p)[55]

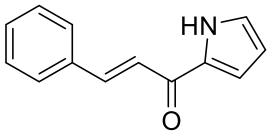

The reaction was performed following the general procedure by introducing in sequence in the vial: benzaldehyde (106 mg, 102 µl, 1 mmol, 1 eq.), 2-acetylpyrrole (109 mg, 1 mmol, 1 eq.), 1 mL of CTAB 2 % or Tween 80 2 %, and NaOH (100 µl of a 40 % solution, 40 mg NaOH, 1 mmol, 1 eq.). The crude was further purified by column chromatography with a gradient of petroleum ether and ethyl acetate. A final crystallization from ethanol gave the pure compound.

From CTAB: Yield: 181.5 mg (92 %).

From Tween 80 at 25°C: Yield: 172.0 mg (87 %).

Light yellow solid, m.p. 196-197 °C (Lit. 195-197°C[55]).

^{1}H NMR (600 MHz, $CDCl_3$) δ 9.67 (s, 1H), 7.83 (dd, J = 15.7, 0.7 Hz, 1H), 7.74 – 7.58 (m, 2H), 7.44 – 7.38 (m, 3H), 7.36 (dd, J = 15.7, 1.0 Hz, 1H), 7.13 – 7.06 (m, 2H), 6.36 (dt, J = 3.8, 2.5 Hz, 1H).

^{13}C{1H} NMR (151 MHz, $CDCl_3$) δ 179.0, 142.5, 135.2, 133.3, 130.4, 129.1, 128.5, 125.5, 122.1, 116.5, 111.2.

HRMS (ESI+) m/z: [M + H]+ Calcd for C13H12NO 198.0913; Found 198.0914.

SERIE 7

3-phenyl-1,5-di-p-tolylpentane-1,5-dione (7b)[39]

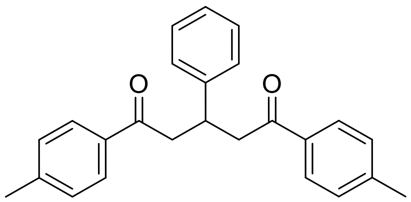

The product was isolated from the synthesis of **6b**. A final crystallization from ethanol gave the pure compound.

From CTAB: Yield: 50.6 mg (14 %).

White solid, m.p. 109-111 °C (Lit. 110-111°C[56]).

^{1}H NMR (600 MHz, $CDCl_3$) δ 7.84 (d, *J* = 8.2 Hz, 4H), 7.27 (m, 4H), 7.23 (dd, *J* = 8.6, 0.7 Hz, 4H), 7.17 (ddd, *J* = 8.6, 5.7, 2.6 Hz, 1H), 4.04 (p, *J* = 7.1 Hz, 1H), 3.45 (dd, *J* = 16.5, 7.0 Hz, 2H), 3.31 (dd, *J* = 16.5, 7.1 Hz, 2H), 2.40 (s, 6H).

$^{13}C\{1H\}$ NMR (151 MHz, DMSO-d_6) δ 198.4, 144.1, 144.0, 134.6, 129.4, 128.7, 128.4, 127.6, 126.7, 45.0, 37.5, 21.8.

HRMS (ESI+) m/z: [M + H]+ Calcd for C25H25O2 357.1849; Found 357.1850.

1,5-bis(4-bromophenyl)-3-phenylpentane-1,5-dione (7e)[39]

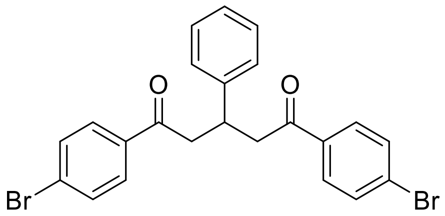

The product was isolated from the synthesis of **6e**. A final crystallization from ethanol gave the pure compound.

From CTAB: Yield: 49.2 mg (14 %).

Light yellow solid, m.p. 129-131 °C (Lit. 128-130 °C[34]).

^{1}H NMR (600 MHz, $CDCl_3$) δ 7.80 (d, *J* = 8.6 Hz, 2H), 7.58 (d, *J* = 8.6 Hz, 2H), 7.26 (m, 3H), 7.20 (m, 1H), 4.00 (m, 1H), 3.45 (dd, *J* = 16.7, 7.0 Hz, 1H), 3.30 (dd, *J* = 16.7, 6.9 Hz, 2H).

$^{13}C\{1H\}$ NMR (151 MHz, $CDCl_3$) δ 197.7, 143.5, 135.7, 132.1, 129.8, 128.9, 128.5, 127.5, 127.6, 44.9, 37.3.

HRMS (ESI+) m/z: [M + H]+ Calcd for C23H19Br2O2 484.9746; Found 484.9726.

1,5-bis(3-bromophenyl)-3-phenylpentane-1,5-dione (7f)

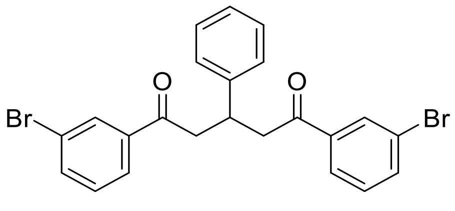

The product was isolated from the synthesis of **6f**. A final crystallization from ethanol gave the pure compound.

From Tween 80: Yield: 34.0 mg (7 %).

White-off solid, m.p. 108-110 °C

^{1}H NMR (600 MHz, $CDCl_3$) δ 8.05 (m, 2H), 7.87 (ddd, *J* = 7.8, 1.7, 1.0 Hz, 2H), 7.67 (ddd, *J* = 7.9, 2.0, 1.0 Hz, 2H), 7.33 (t, *J* = 7.9 Hz, 2H), 7.28 (m, 4H), 7.20 (m, 1H), 4.03 (p, *J* = 6.9 Hz, 1H), 3.45 (dd, *J* = 16.9, 7.0 Hz, 2H), 3.33 (dd, *J* = 16.9, 6.9 Hz, 2H).

$^{13}C\{1H\}$ NMR (151 MHz, $CDCl_3$) δ 197.2, 143.5, 138.7, 136.2, 131.4, 130.4, 128.9, 127.5, 127.1, 126.8, 123.1, 44.9, 37.0.

HRMS (ESI+) m/z: [M + H]+ Calcd for C23H19Br2O2 484.9746; Found 484.9742.

3-(4-nitrophenyl)-1,5-diphenylpentane-1,5-dione (7h)[57]

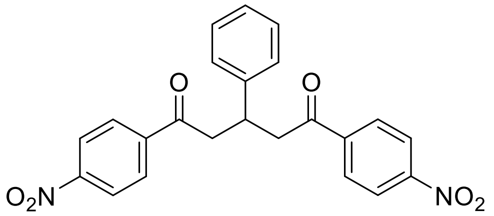

The product was isolated from the synthesis of **6h**. A final crystallization from ethanol gave the pure compound.

From Tween 80: Yield: 59.4 mg (14 %).

Light yellow solid, m.p. 111-113 °C.

^{1}H NMR (600 MHz, $CDCl_3$) δ 8.30 (d, *J* = 9.0 Hz, 4H), 8.09 (d, *J* = 9.0 Hz, 4H), 7.30 (m, 2H), 7.27 (m, 2H), 7.22 (m, 2H), 4.05 (p, *J* = 6.9 Hz, 1H), 3.56 (dd, *J* = 16.9, 7.1 Hz, 2H), 3.41 (dd, *J* = 16.9, 6.8 Hz, 2H).

^{13}C{1H} NMR (151 MHz, $CDCl_3$) δ 197.1, 150.5, 142.8, 141.2, 129.3, 129.1, 127.5, 127.4, 124.0, 45.3, 37.1.

HRMS (ESI+) m/z: [M+ H]+ Calcd for C23H19N2O6 419.1238; Found 419.1240.

3-phenyl-1,5-di(pyridin-2-yl)pentane-1,5-dione (7m)[58, 59]

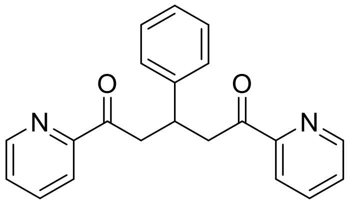

The product was isolated from the synthesis of **6m**. A final crystallization from ethanol gave the pure compound.

From CTAB: Yield: 47.6 mg (14 %).

From Tween 80: Yield: 36.0 mg (11 %).

White-transparent crystals, m.p. 149-151 °C (Lit. 149-151 °C[60]).

^{1}H NMR (600 MHz, $CDCl_3$) δ 8.64 (ddd, *J* = 4.7, 1.7, 0.9 Hz, 2H), 7.95 (dt, *J* = 7.8, 1.1 Hz, 2H), 7.78 (td, *J* = 7.7, 1.7 Hz, 2H), 7.43 (ddd, *J* = 7.5, 4.7, 1.3 Hz, 2H), 7.38 – 7.35 (m, 2H), 7.24 (t, *J* = 7.7 Hz, 2H), 7.17 – 7.10 (m, 1H), 4.15 (q, *J* = 7.1 Hz, 1H), 3.76 (dd, *J* = 17.6, 7.5 Hz, 2H), 3.64 (dd, *J* = 17.6, 6.8 Hz, 2H).

^{13}C{1H} NMR (151 MHz, $CDCl_3$) δ 200.2, 153.6, 149.0, 137.0, 128.5, 127.9, 127.2, 126.5, 122.0, 44.3, 36.3.

HRMS (ESI+) m/z: [M + H]+ Calcd for C21H19N2O2 331.1441; Found 331.1441.

3-phenyl-1,5-di(thiophen-2-yl)pentane-1,5-dione (**7n**)[61]

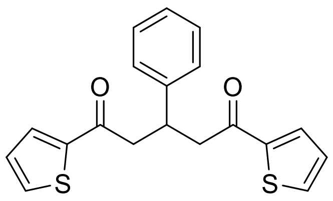

The product was isolated from the synthesis of **6n**. A final crystallization from ethanol gave the pure compound.

From CTAB: Yield: 10.2 mg (3 %).

From Tween 80: Yield: 7.8 mg (2 %).

Yellow solid, m.p. 101-102 °C (Lit. 101-102 °C[62]).

^{1}H NMR (600 MHz, $CDCl_3$) δ 7.75 (dd, *J* = 3.8, 1.1 Hz, 2H), 7.61 (dd, *J* = 4.9, 1.1 Hz, 2H), 7.28 (m, 4H), 7.19 (ddt, *J* = 6.6, 5.7, 2.5 Hz, 1H), 7.11 (dd, J = 5.0, 3.8 Hz, 2H), 4.05 (t, J = 7.1 Hz, 1H), 3.43 (dd, J = 16.0, 7.0 Hz, 2H), 3.28 (dd, J = 16.0, 7.1 Hz, 2H).

^{13}C{1H} NMR (151 MHz, $CDCl_3$) δ 191.5, 144.4, 143.3, 133.9, 132.3, 128.8, 128.3, 127.6, 127.0, 45.5, 38.1.

HRMS (ESI+) m/z: [M + H]+ Calcd for C19H17O2S2 341.0664; Found 341.0664.

Other Compounds (8-17)

2-phenylchroman-4-one (8d)[63]

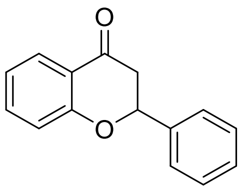

The product was isolated from the synthesis of **6d**. A final crystallization from ethanol gave the pure compound

From Tween 80: Yield: 16.4 mg (7. %).

White solid, m.p. 75-77 °C (Lit. 76-77°C[63]).

^{1}H NMR (600 MHz, $CDCl_3$) δ 7.94 (dd, *J* = 8.1, 1.8 Hz, 1H), 7.51 (m, 3H), 7.45 (m, 2H), 7.40 (m, 1H), 5.50 (dd, *J* = 13.4, 2.8 Hz, 1H), 3.10 (dd, *J* = 16.9, 13.4 Hz, 1H), 2.90 (dd, *J* = 16.8, 2.9 Hz, 1H).

^{13}C{1H} NMR (151 MHz, $CDCl_3$) δ 192.2, 161.7, 138.9, 136.4, 129.02, 128.95, 127.2, 126.3, 121.8, 121.1, 118.3, 79.8, 44.8.

HRMS (ESI+) m/z: [M+ H]+ Calc for C15H13O2 225.0910; Found 225.0905.

(E)-3-(4-(dimethylamino)phenyl)-1-phenylprop-2-en-1-one (9)[64]

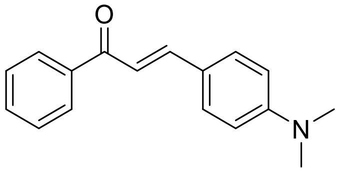

The reaction was performed following the general procedure by introducing in sequence in the vial: 4-(N.N-dimethylamino)benzaldehyde (149 mg, 1 mmol, 1 eq.), acetophenone (120 mg, 117 µl, 1 mmol, 1 eq.), 1 mL of CTAB 2 % or Tween 80 2 %, and NaOH (100 µl of a 4 0% solution, 40 mg NaOH, 1 mmol, 1 eq.). The crude was further purified by column chromatography with a gradient of petroleum ether and ethyl acetate. A final crystallization from ethanol gave the pure compound.

From CTAB: Yield: 158.1 mg (63 %).

From Tween 80: Yield: 8.8 mg (4 %).

Orange solid, m.p. 110-111 °C (Lit. 111-112°C[3]).

^{1}H NMR (600 MHz, $CDCl_3$) δ 8.00 (dd, J = 8.3, 1.3 Hz, 1H), 7.79 (d, J = 15.5 Hz, 1H), 7.55 (m, 1H), 7.49 (t, J = 7.7 Hz, 1H), 7.34 (d, J = 15.5 Hz, 1H), 6.70 (d, J = 8.9 Hz, 1H), 3.05 (s, 3H).

^{13}C{1H} NMR (151 MHz, $CDCl_3$) δ 190.9, 152.2, 146.0, 139.2, 132.3, 130.6, 128.6, 128.5, 122.8, 117.1, 112.0, 40.3.

HRMS (ESI+) m/z: [M + H]+ Calcd for C17H18NO 252.1383; Found 252.1372.

(E)-3-(4-(dimethylamino)phenyl)-1-(2-hydroxyphenyl)prop-2-en-1-one (**10**)[65]

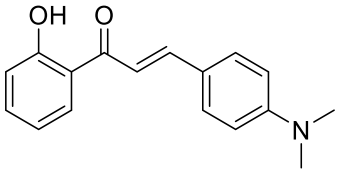

The reaction was performed following the general procedure by introducing in sequence in the vial: 4-(N.N-dimethylamino)benzaldehyde (149 mg, 1 mmol, 1 eq.), 2-hydroxyacetophenone (136 mg, 120 µl, 1 mmol, 1 eq.), 1 mL of CTAB 2 % or Tween 80 2 %, and NaOH (400 µl of a 40 % solution, 160 mg NaOH, 1 mmol, 1 eq.). The crude was further purified by column chromatography with a gradient of petroleum ether and ethyl acetate. A final crystallization from ethanol gave the pure compound.

From CTAB at 45°C: Yield: 67.9 mg (25 %).

From Tween 80 at 45°C: Yield: 76.2 mg (29 %).

Orange-red solid, m.p. 170-171 °C (Lit. 170-171°C[66]).

^{1}H NMR (600 MHz, $CDCl_3$) δ 13.20 (s, 1H), 7.93 (m, 2H), 7.58 (d, J = 9.0 Hz, 2H), 7.46 (d, J = 15.1 Hz, 2H), 7.46 (m, 1H), 7.01 (dd, J = 8.4, 0.9 Hz, 1H), 6.92 (ddd, J = 8.2, 7.2, 1.2 Hz, 1H), 6.70 (d, J = 8.9 Hz, 2H), 3.07 (s, 6H).

$^{13}C\{1H\}$ NMR (151 MHz, $CDCl_3$) δ 193.7, 163.6, 152.5, 146.7, 135.8, 131.0, 129.5, 122.5, 120.5, 118.7, 118.6, 114.5, 112.0, 40.3.

HRMS (ESI+) m/z: [M + H]+ Calcd for C17H18NO2 268.1322; Found 268.1320.

(E)-3-(4-(dimethylamino)phenyl)-1-(4-methoxyphenyl)prop-2-en-1-one (11)[64, 67]

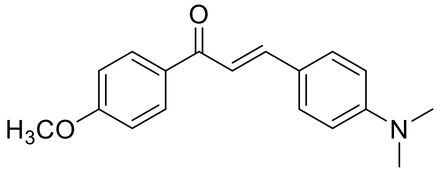

The reaction was performed following the general procedure by introducing in sequence in the vial: 4-(N.N-dimethylamino)benzaldehyde (149 mg, 1 mmol, 1 eq.), 4-methoxyacetophenone (150 mg, 1 mmol, 1 eq.), 1 mL of CTAB 2 % or Tween 80 2 %, and NaOH (100 µl of a 40 % solution, 40 mg NaOH, 1 mmol, 1 eq.). The crude was further purified by column chromatography with a gradient of petroleum ether and ethyl acetate. A final crystallization from ethanol gave the pure compound.

From CTAB at 25°C: Yield: 85.0 mg (30 %).

From CTAB at 45°C: Yield: 83.6 mg (30 %).

Dark yellow solid, m.p. 114-115 °C (Lit. 117°C[68]).

^{1}H NMR (600 MHz, $CDCl_3$) δ 8.03 (d, J = 9.0 Hz, 2H), 7.79 (d, J = 15.4 Hz, 1H), 7.55 (dd, J = 9.0, 0.5 Hz, 2H), 7.36 (d, J = 15.4 Hz, 1H), 6.97 (d, J = 8.9 Hz, 2H), 6.70 (d, J = 8.8 Hz, 2H), 3.89 (s, 3H), 3.05 (s, 6H).

$^{13}C\{1H\}$ NMR (151 MHz, $CDCl_3$) δ 189.1, 163.1, 152.0, 145.1, 132.0, 130.7, 130.4, 123.0, 116.8, 113.8, 112.0, 55.6, 40.3.

HRMS (ESI+) m/z: [M + H]+ Calcd for C18H20NO2 282.1489; Found 282.1478.

Z isomer, detected in ethanol solution due to photoisomerization: ^{1}H NMR (600 MHz, $CDCl_3$) δ 7.98 (d, J = 9.0 Hz, 1H), 7.57 (dd, J = 9.1, 0.6 Hz, 1H), 6.89 (d, J = 9.0 Hz, 1H), 6.82 (d, J = 12.8 Hz, 1H), 6.57 (d, J = 9.0 Hz, 1H), 6.45 (d, J = 12.8 Hz, 1H).

[1-Oxo-3-(2-pyridinyl)-2-propen-1-yl]ferrocene (12)[69]

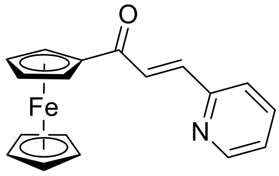

The reaction was performed following the general procedure by introducing in sequence in the vial: 2-pyridinecarboxaldehyde (107 mg, 95 µl, 1 mmol, 1 eq.), acetylferrocene (228 mg, 1 mmol, 1 eq.), 1 mL of CTAB 2 % or Tween 80 2 %, and NaOH (100 µl of a 40 % solution, 40 mg NaOH, 1 mmol, 1 eq.). The crude was further purified by column chromatography with a gradient of petroleum ether and ethyl acetate. A final crystallization from ethanol gave the pure compound.

From CTAB: Yield: 140.8 mg (44 %).

From Tween 80: Yield: 30.3 mg (14 %).

Red solid, m.p. 174-175 °C (Lit. 173-174°C [69]).

^{1}H NMR (600 MHz, $CDCl_3$) δ 8.70 (ddd, J = 4.8, 1.9, 0.8 Hz, 1H), 7.73 (m, 2H), 7.66 (d, J = 15.2 Hz, 1H), 7.46 (dt, J = 7.8, 1.1 Hz, 1H), 7.29 (ddd, J = 7.6, 4.7, 1.2 Hz, 1H), 4.98 (m, 2H), 4.61 (dd, J = 2.2, 1.7 Hz, 2H), 4.23 (s, 5H).

^{13}C{1H} NMR (151 MHz, $CDCl_3$) δ 193.4, 153.7, 150.3, 139.3, 137.0, 126.8, 125.6, 124.2, 80.7, 73.2, 70.3, 70.1.

HRMS (ESI+) m/z: [M+ H]+ Calcd for C18H16FeNO 318.0576; Found 318.0562.

[1-Oxo-3-(2-pyridinyl)-2-propen-1-yl]ferrocene (13)[70]

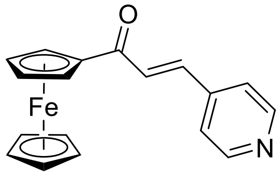

The reaction was performed following the general procedure by introducing in sequence in the vial: 2-pyridinecarboxaldehyde (107 mg, 94 µl, 1 mmol, 1 eq.), acetylferrocene (228 mg, 1 mmol, 1 eq.), 1 mL of CTAB 2 % or Tween 80 2 %, and NaOH (100 µl of a 40 % solution, 40 mg NaOH, 1 mmol, 1 eq.). The crude was further purified by column chromatography with a gradient of petroleum ether and ethyl acetate. A final crystallization from ethanol gave the pure compound.

From CTAB at 25°C: Yield: 72.0 mg (23 %).

From CTAB at 45°C: Yield: 47.3 mg (15 %).

From Tween 80 at 25°C: Yield: 44.7 mg (14 %).

From Tween 80 at 45°C: Yield: 38.4 mg (12 %).

Red solid, m.p. 193-195 °C (Lit. 195-196°C [69]).

^{1}H NMR (600 MHz, $CDCl_3$) δ 8.71 – 8.67 (m, 2H), 7.69 (d, J = 15.7 Hz, 1H), 7.48 (d, J = 6.2 Hz, 2H), 7.23 (d, J = 15.7 Hz, 1H), 4.91 (m, 2H), 4.65 (dd, J = 2.2, 1.7 Hz, 2H), 4.23 (s, 5H).

^{13}C{1H} NMR (151 MHz, $CDCl_3$) δ 192.4, 150.8, 142.7, 137.9, 127.1, 122.1, 80.3, 73.4, 70.4, 70.0.

HRMS (ESI+) m/z: [M+ H]+ Calcd for C18H16FeNO 318.0576; Found 318.0562.

(E)-3-(4-bromophenyl)-1-(thiophen-2-yl)prop-2-en-1-one (14)[71]

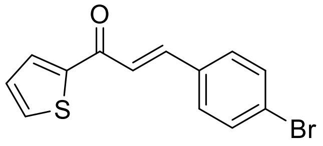

The reaction was performed following the general procedure by introducing in sequence in the vial: 4-bromobenzaldehyde (185 mg, 1 mmol, 1 eq.), acetylthiophene (126 mg, 108 µl, 1 mmol, 1 eq.), 1 mL of CTAB 2 % or Tween 80 2 %, and NaOH (100 µl of a 40 % solution, 40 mg NaOH, 1 mmol, 1

eq.). The crude was further purified by column chromatography with a gradient of petroleum ether and ethyl acetate. A final crystallization from ethanol gave the pure compound.

From CTAB: Yield: 211.4 mg (72 %).

From Tween 80: Yield: 256.8 mg (88 %).

White solid, m.p. 135-137 °C (Lit. 233-234 °C[72], 136-137 °C[73], 132-133 °C[74]).

^{1}H NMR (600 MHz, $CDCl_3$) δ 7.87 (dd, J = 3.8, 1.1 Hz, 1H), 7.78 (dt, J = 15.6, 0.5 Hz, 1H), 7.70 (dd, J = 4.9, 1.1 Hz, 1H), 7.56 (m, 2H), 7.51 (m, 2H), 7.40 (d, J = 15.6 Hz, 1H), 7.20 (dd, J = 4.9, 3.8 Hz, 1H).

^{13}C{1H} NMR (151 MHz, $CDCl_3$) δ 181.9, 145.5, 142.8, 134.3, 133.8, 132.4, 132.1, 130.0, 128.5, 125.0, 122.3.

HRMS (ESI+) m/z: [M + H]+ Calcd for C13H10BrOS 292.9630; Found 292.9619.

(E)-3-(4-bromophenyl)-1-(pyridin-2-yl)prop-2-en-1-one (**15**)[75]

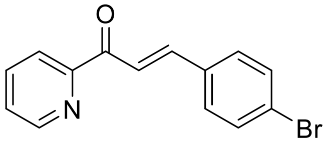

The reaction was performed following the general procedure by introducing in sequence in the vial: 4-bromobenzaldehyde (185 mg, 1 mmol, 1 eq.), 2-acetylpyridine (121 mg, 112 µl, 1 mmol, 1 eq.), 1 mL of CTAB 2 % or Tween 80 2 %, and NaOH (100 µl of a 40 % solution, 40 mg NaOH, 1 mmol, 1 eq.). The crude was further purified by column chromatography with a gradient of petroleum ether and ethyl acetate. A final crystallization from ethanol gave the pure compound.

From CTAB: Yield: 203.4 mg (71 %).

From Tween 80: Yield: 110.4 mg (38 %).

Light yellow solid, m.p. 119-120 °C (Lit. 118-119°C[76]).

^{1}H NMR (600 MHz $CDCl_3$) δ 8.74 (ddd, J = 4.7, 1.7, 0.9 Hz, 1H), 8.30 (d, J = 16.0 Hz, 1H), 8.19 (ddd, J = 7.8, 0.9 Hz, 1H), 7.87 (m, 2H), 7.59 (m, 2H), 7.55 (m, 2H), 7.50 (ddd, J = 7.5, 4.7, 1.2 Hz, 1H).

^{13}C{1H} NMR (151 MHz, $CDCl_3$) δ 189.5, 154.2, 149.0, 143.4, 137.2, 134.2, 132.3, 130.3, 127.2, 125.0, 123.1, 121.6.

HRMS (ESI+) m/z: [M + H]+ Calcd for C14H11BrNO 288.0019; Found 288.0011.

(E)-3-(3-nitrophenyl)-1-(2,4,6-trimethoxyphenyl)prop-2-en-1-one (16)[77]

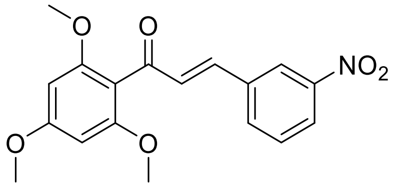

The reaction was performed following the general procedure by introducing in sequence in the vial: 3-nitrobenzaldehyde (151 mg, 1 mmol, 1 eq.), 2',4',6'-trimethoxyacetophenone (210 mg, 1 mmol, 1 eq.), 1 mL of CTAB 2 % or Tween 80 2 %, and NaOH (100 µl of a 40 % solution, 40 mg NaOH, 1 mmol, 1 eq.). The crude was further purified by column chromatography with a gradient of petroleum ether and ethyl acetate. A final crystallization from ethanol gave the pure compound.

From CTAB: Yield: 176.5 mg (51 %).

From Tween 80: Yield: 117.8 mg (34 %).

Yellow solid, m.p. 145-146 °C (Lit. 144-146°C[78]).

^{1}H NMR (600 MHz, $CDCl_3$) δ 8.34 (s, 1H), 8.19 (m, 1H), 7.83 (m, 1H), 7.55 (t, J = 8.0 Hz, 1H), 7.42 (d, J = 16.0 Hz, 1H), 7.05 (d, J = 15.9 Hz, 1H), 6.16 (s, 2H), 3.86 (s, 3H), 3.78 (6H).

^{13}C{1H} NMR (151 MHz, $CDCl_3$) δ 193.1, 163.0, 159.2, 148.7, 140.3, 137.6, 133.9, 131.5, 130.0, 124.4, 122.8, 111.5, 90.9, 56.1, 55.6.

HRMS (ESI+) m/z: [M + H]+ Calcd for C18H18NO6 344.1129; Found 344.1115.

(E)-1-(3,4-dimethoxyphenyl)-3-(3,4,5-trimethoxyphenyl)prop-2-en-1-one (**17**)[79]

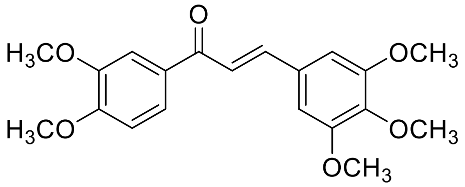

The reaction was performed following the general procedure by introducing in sequence in the vial: 3,4,5-trimethoxybenzaldehyde (196 mg, 1 mmol, 1 eq.), 3',4'-imethoxyacetophenone (180 mg, 1 mmol, 1 eq.), 1 mL of CTAB 2 % or Tween 80 2 %, and NaOH (100 µl of a 40 % solution, 40 mg NaOH, 1 mmol, 1 eq.). The crude was further purified by column chromatography with a gradient of petroleum ether and ethyl acetate. A final crystallization from ethanol gave the pure compound.

From CTAB: Yield: 246.9 mg (69 %).

From Tween 80: Yield: 327.6 mg (91 %).

White solid, m.p. 124-126 °C (Lit. 124-126°C[79]).

^{1}H NMR (600 MHz, $CDCl_3$) δ 7.72 (d, J = 15.5 Hz, 1H), 7.68 (dd, J = 8.4, 2.0 Hz, 1H), 7.62 (d, J = 2.0 Hz, 1H), 7.43 (d, J = 15.5 Hz, 1H), 6.94 (d, J = 8.5 Hz, 1H), 6.87 (d, J = 0.5 Hz, 2H), 3.98 (s, 3H), 3.97 (s, 3H), 3.93 (s, 6H), 3.90 (s, 3H).

^{13}C{1H} NMR (151 MHz, $CDCl_3$) δ 188.8, 153.6, 153.4, 149.5, 144.3, 140.5, 131.5, 130.7, 123.1, 121.2, 111.0, 110.1, 105.8, 61.2, 56.4, 56.26, 56.24.

HRMS (ESI+) m/z: [M + h]+ Calcd for C20H23O6 359.1489; Found 359.1475.

NMR Spectra (at 600 MHz for 1H and 151 MHz for 13C{1H})

^{1}H and ^{13}C{1H} spectra of series 3

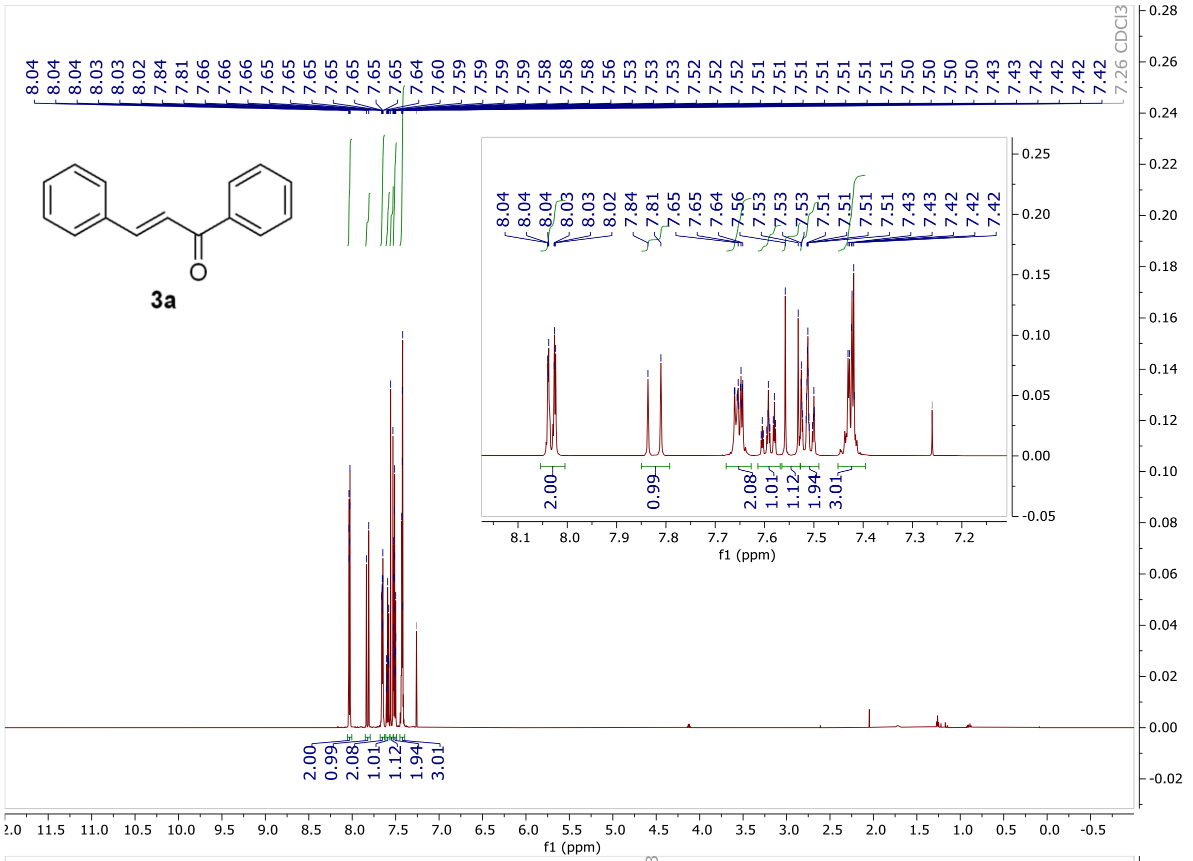

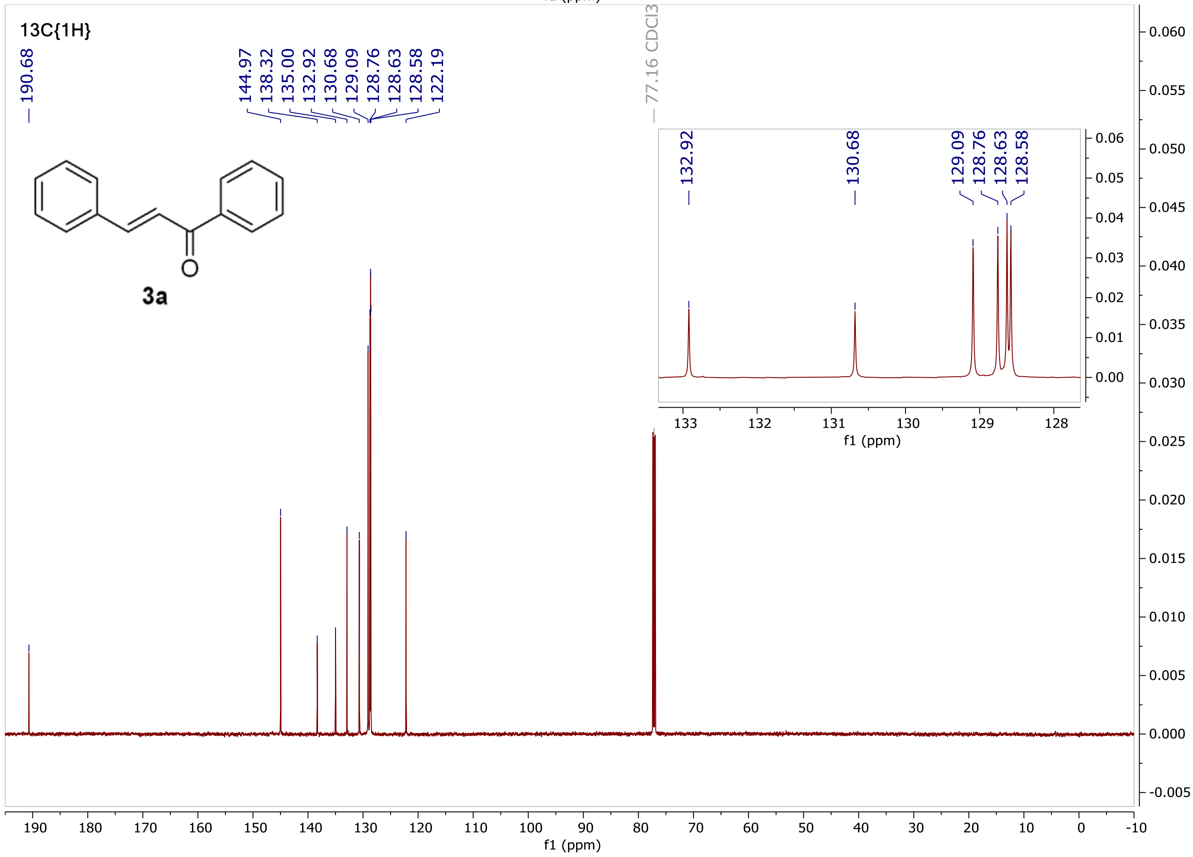

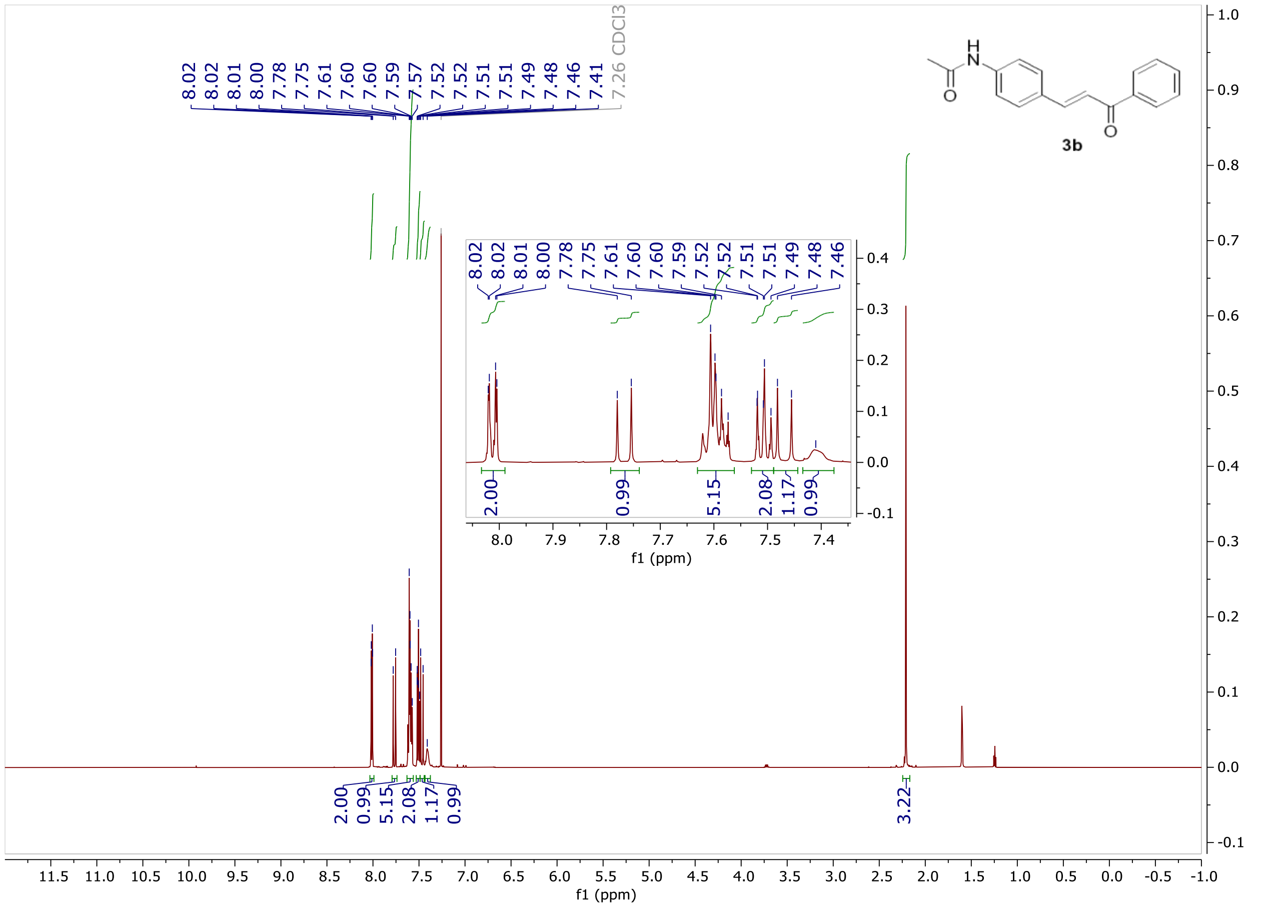
8.02
8.02
8.01
8.00
7.78
7.75
7.61
7.60
7.60
7.59
7.57
7.52
7.52
7.51
7.51
7.49
7.48
7.46
7.41
7.26 CDCl3
3b
2.00
0.99
5.15
2.08
1.17
0.99
3.22
f1 (ppm)

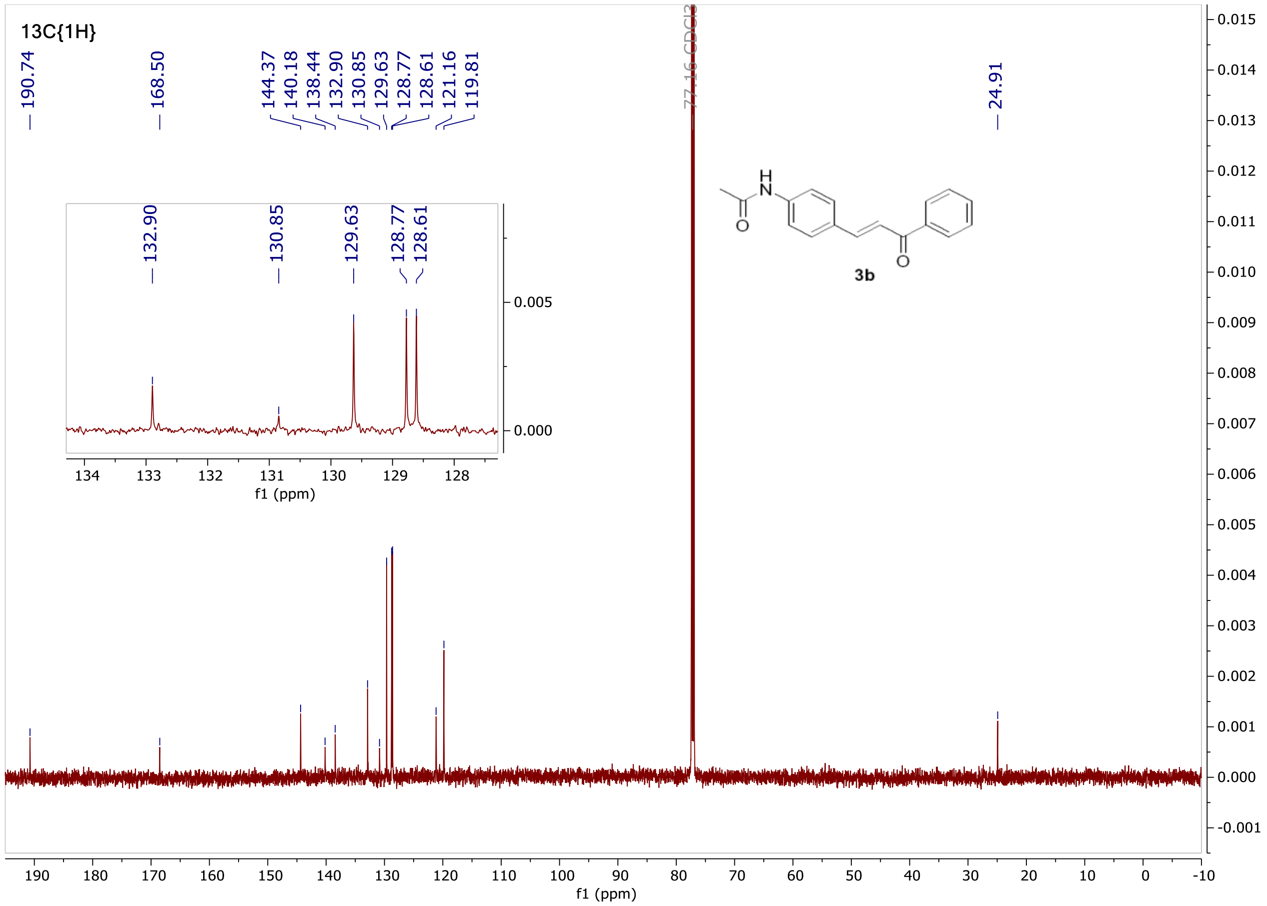
13C{1H}
190.74
168.50
144.37
140.18
138.44
132.90
130.85
129.63
128.77
128.61
121.16
119.81
77.16 CDCl3
24.91
3b
f1 (ppm)

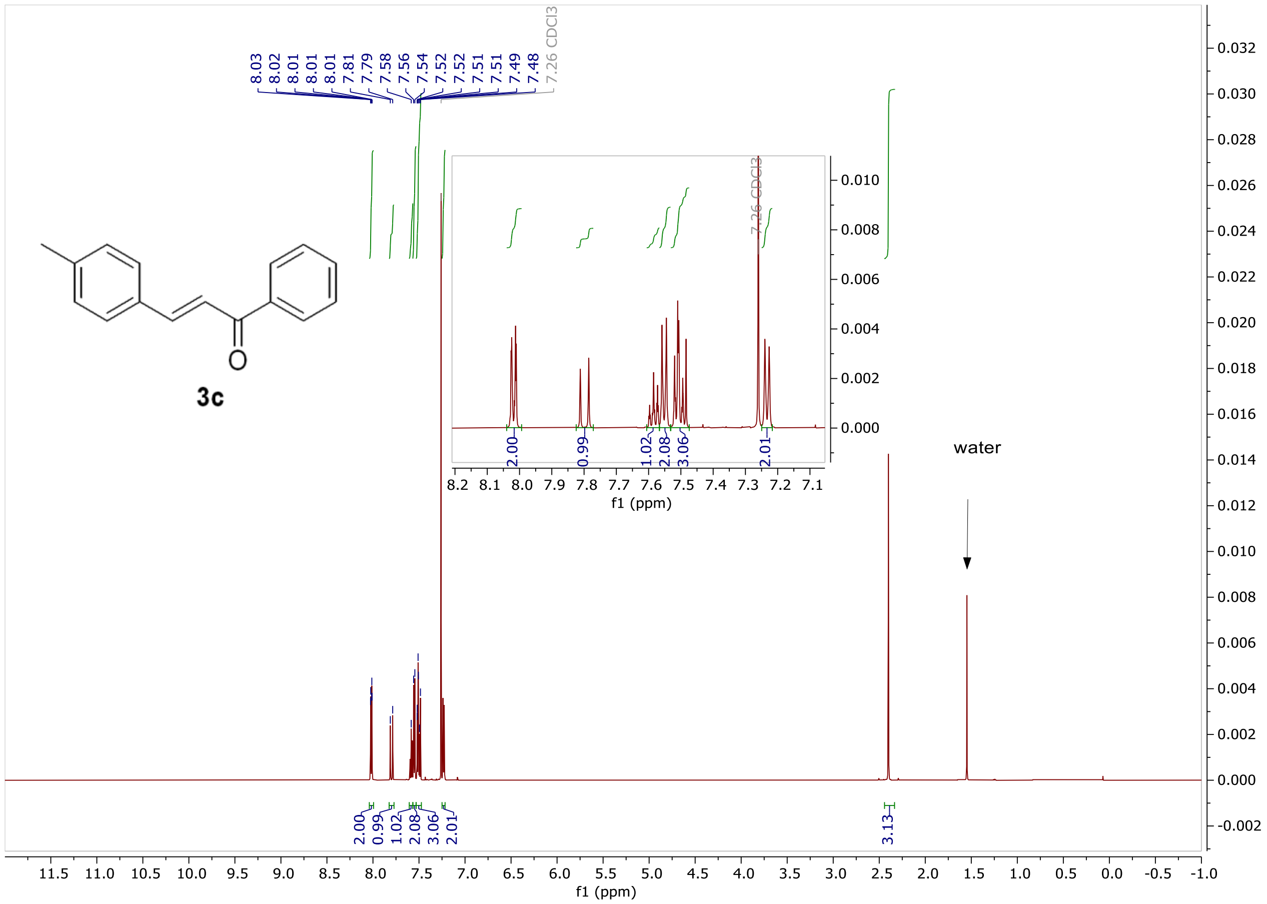
3c
water
f1 (ppm)

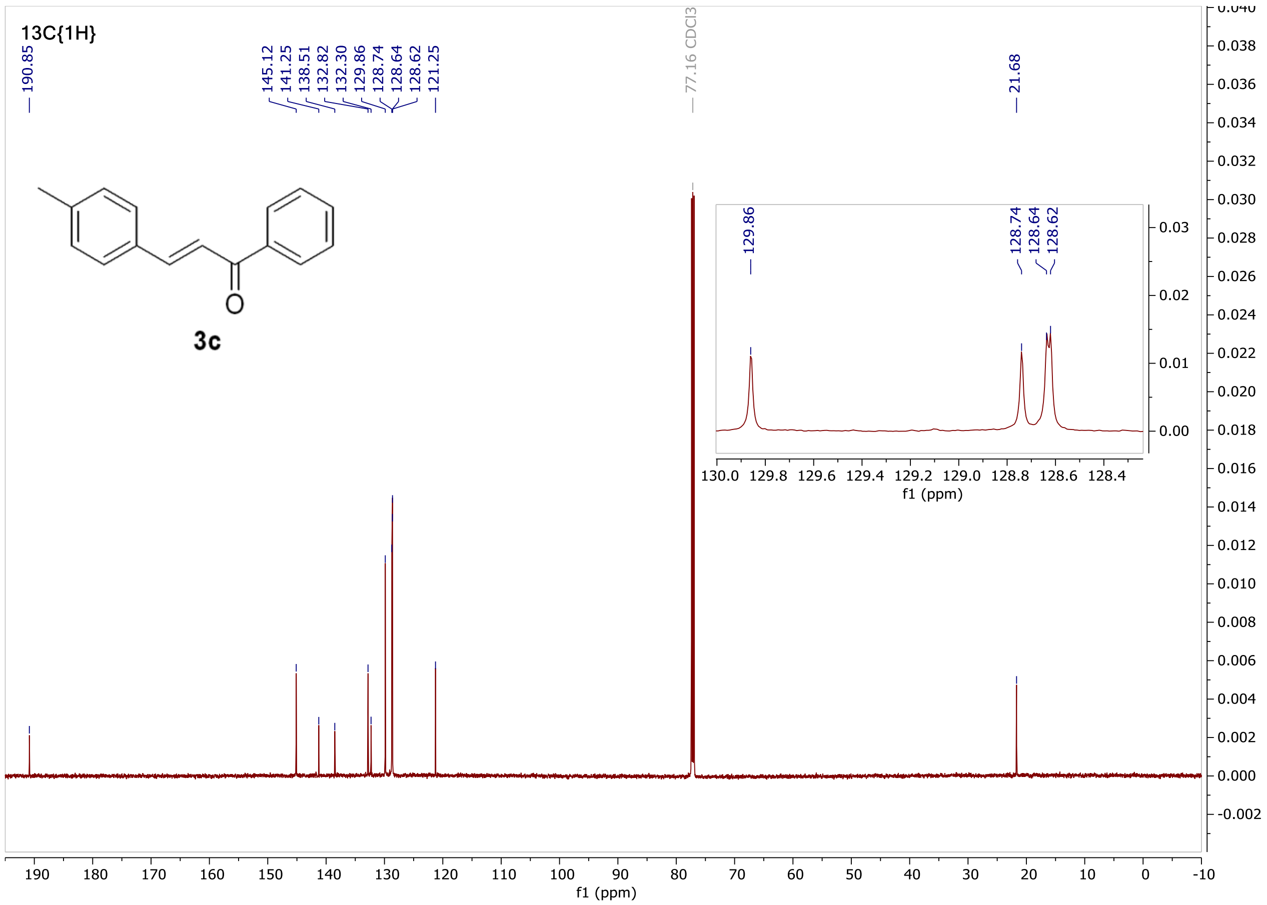
13C{1H}
3c
f1 (ppm)

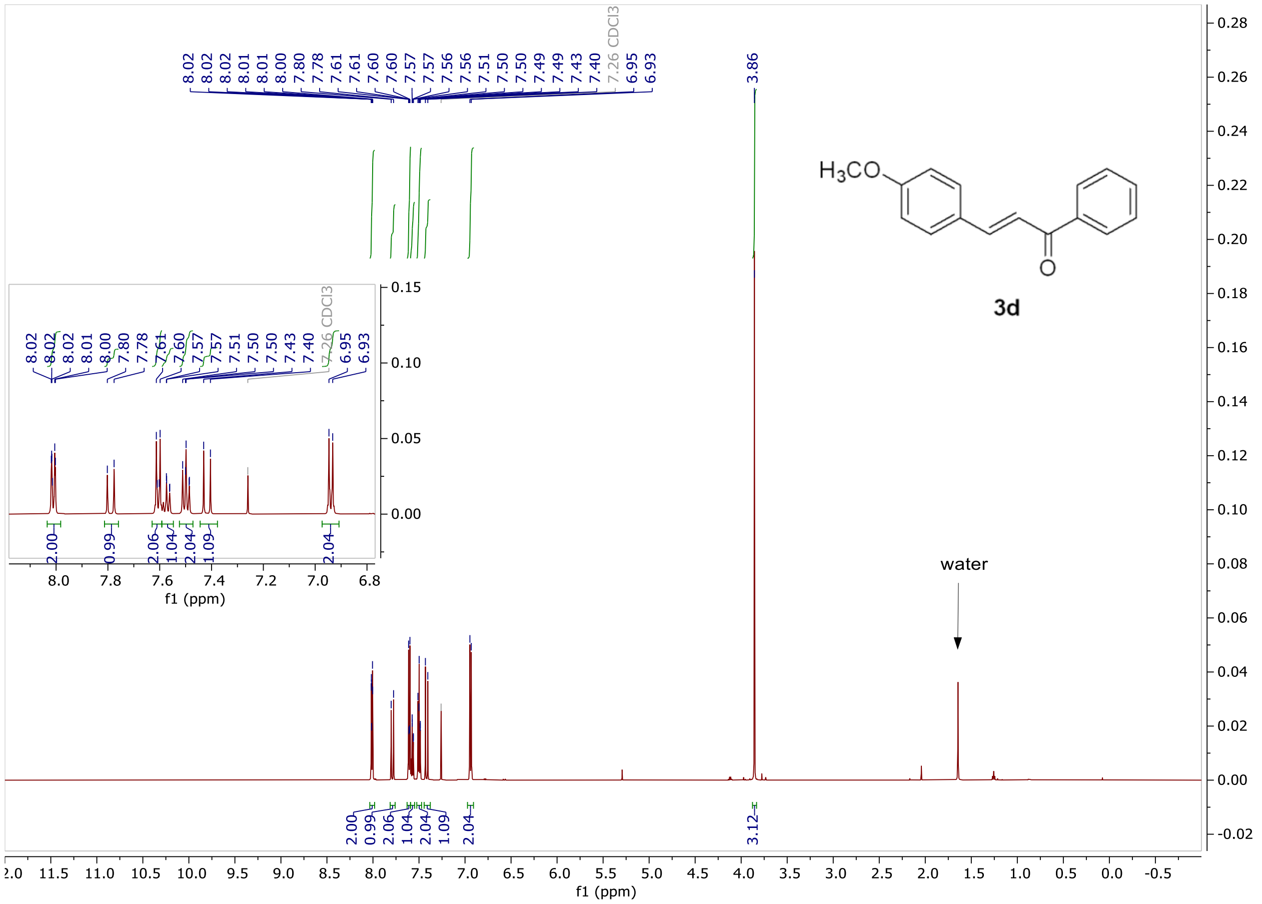
8.02 8.02 8.02 8.01 8.01 8.00 7.80 7.78 7.61 7.61 7.60 7.60 7.57 7.57 7.56 7.56 7.51 7.50 7.50 7.49 7.49 7.43 7.40 7.26 CDCl3 6.95 6.93
3.86
H3CO
3d
water
2.00 0.99 2.06 1.04 2.04 1.09 2.04
3.12
f1 (ppm)

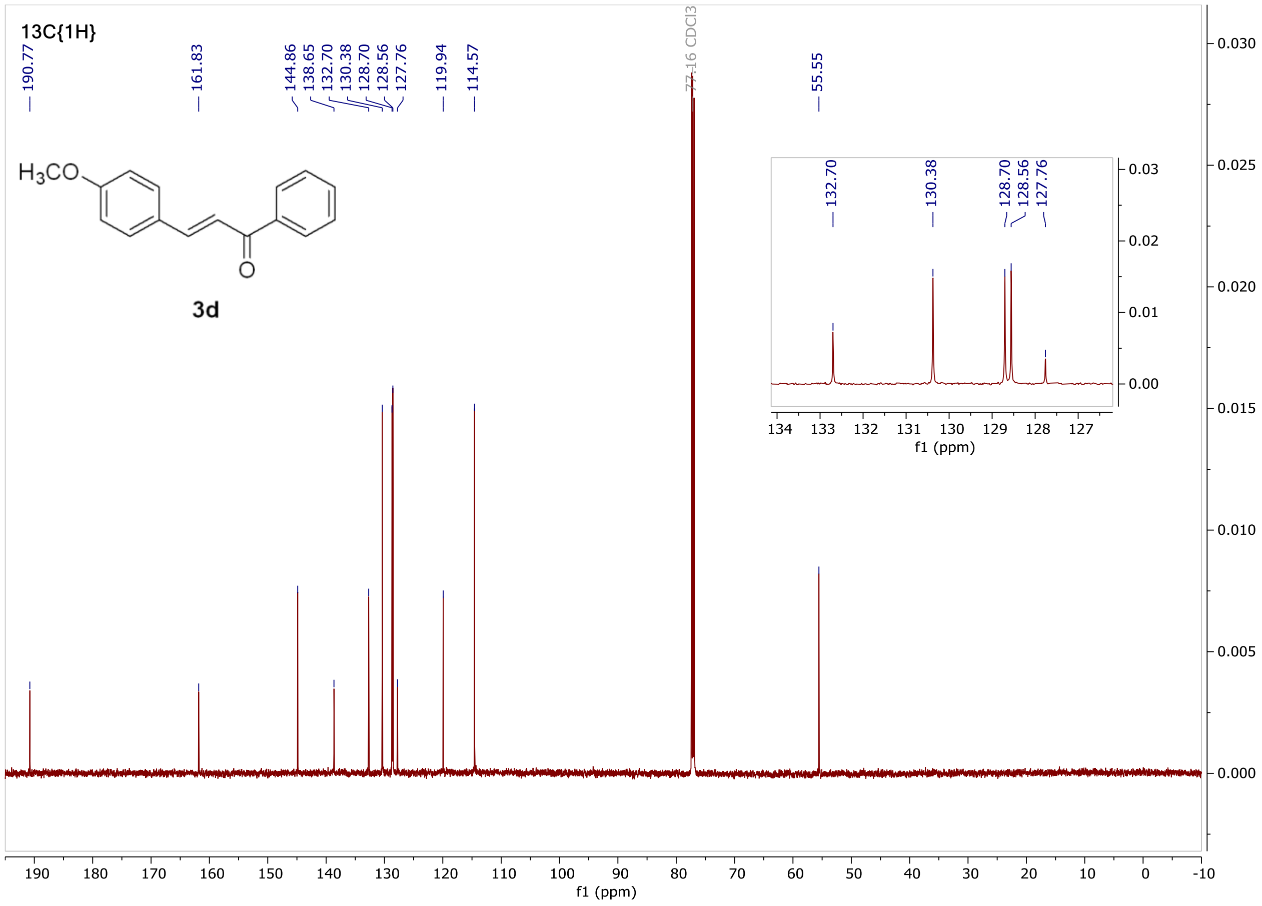
13C{1H}
190.77
161.83
144.86 138.65 132.70 130.38 128.70 128.56 127.76
119.94
114.57
77.16 CDCl3
55.55
H3CO
3d
f1 (ppm)

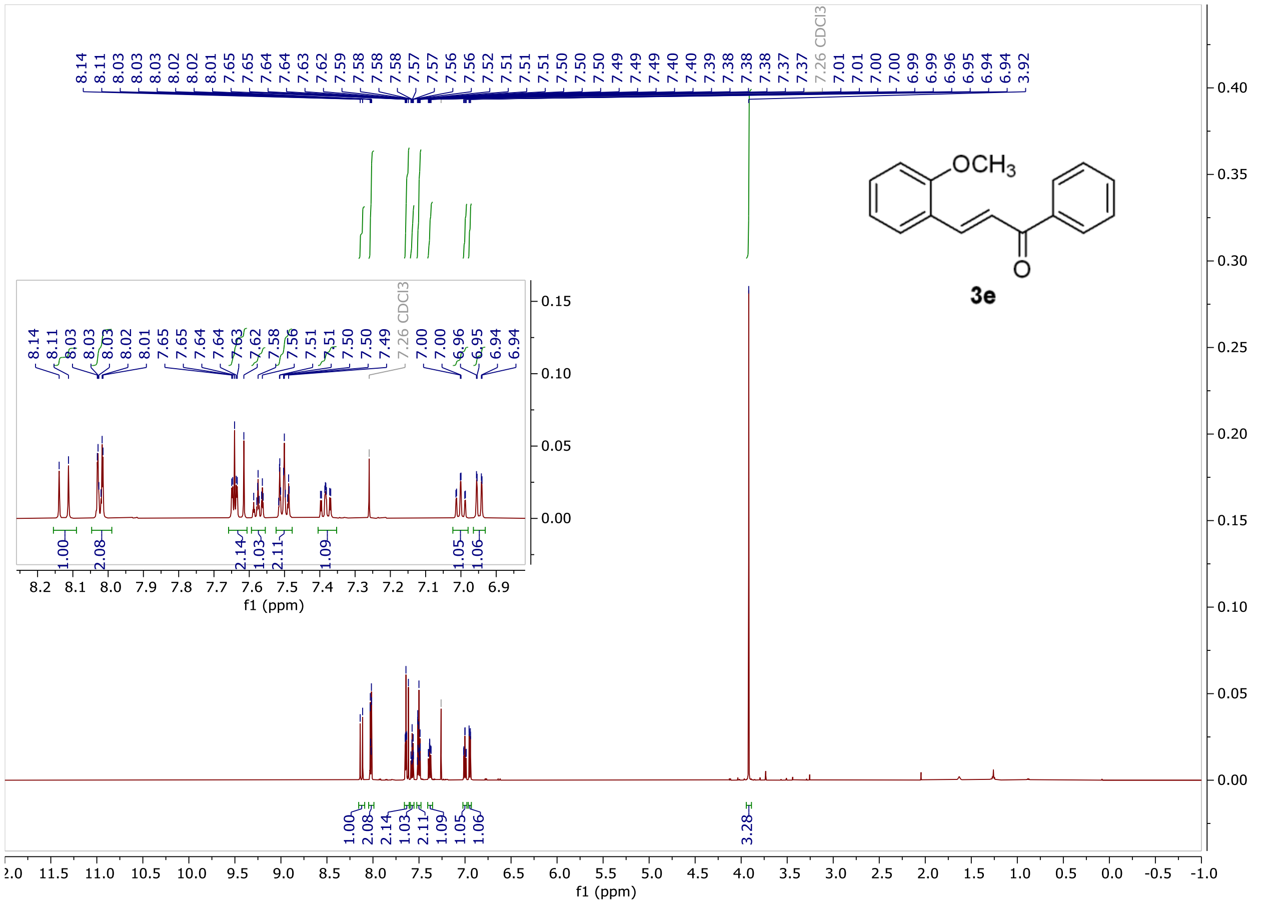
8.14 8.11 8.03 8.03 8.03 8.02 8.02 8.01 7.65 7.65 7.64 7.64 7.63 7.62 7.59 7.58 7.58 7.58 7.57 7.57 7.56 7.56 7.52 7.51 7.51 7.51 7.50 7.50 7.50 7.49 7.49 7.49 7.40 7.40 7.39 7.38 7.38 7.38 7.37 7.37 7.26 CDCl3 7.01 7.01 7.00 7.00 6.99 6.96 6.95 6.95 6.94 6.94 3.92
OCH3
3e
1.00 2.08 2.14 1.03 2.11 1.09 1.05 1.06 3.28
f1 (ppm)

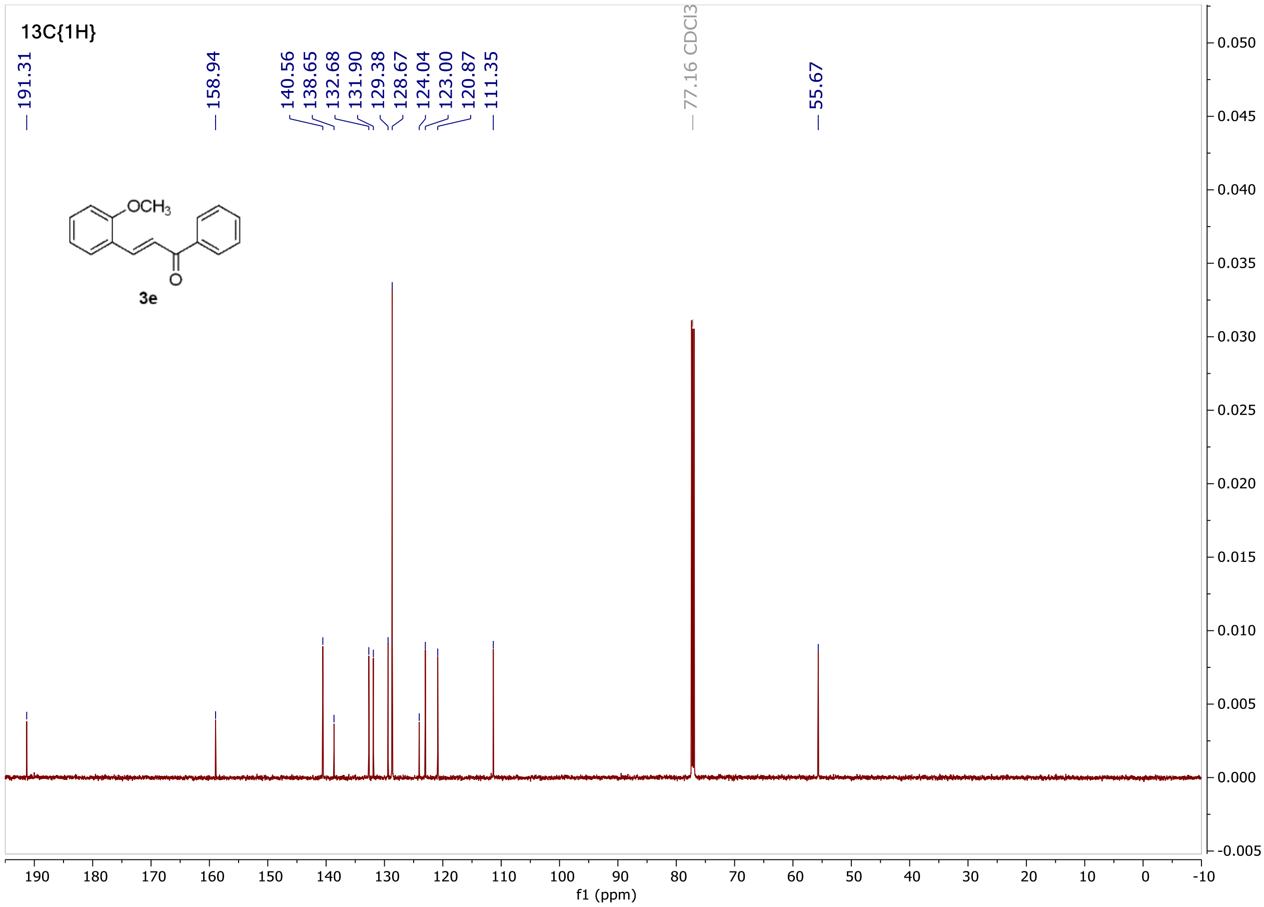
13C{1H}
191.31 158.94 140.56 138.65 132.68 131.90 129.38 128.67 124.04 123.00 120.87 111.35 77.16 CDCl3 55.67
OCH3
3e
f1 (ppm)

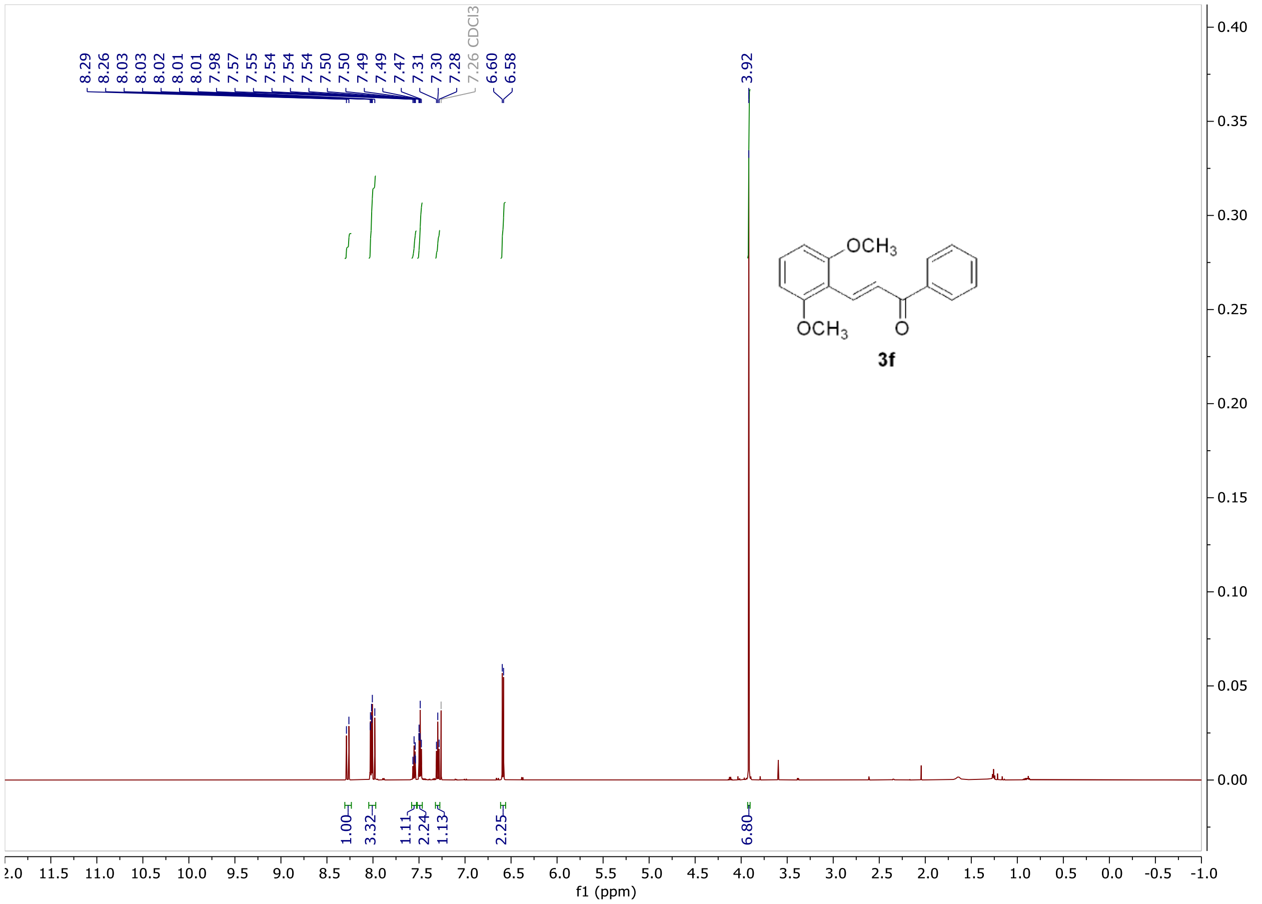

8.29
8.26
8.03
8.02
8.01
7.98
7.57
7.55
7.54
7.54
7.50
7.49
7.49
7.47
7.31
7.30
7.28
7.26 CDCl3
6.60
6.58
3.92
OCH3
OCH3
O
3f
1.00
3.32
1.11
2.24
1.13
2.25
6.80
f1 (ppm)

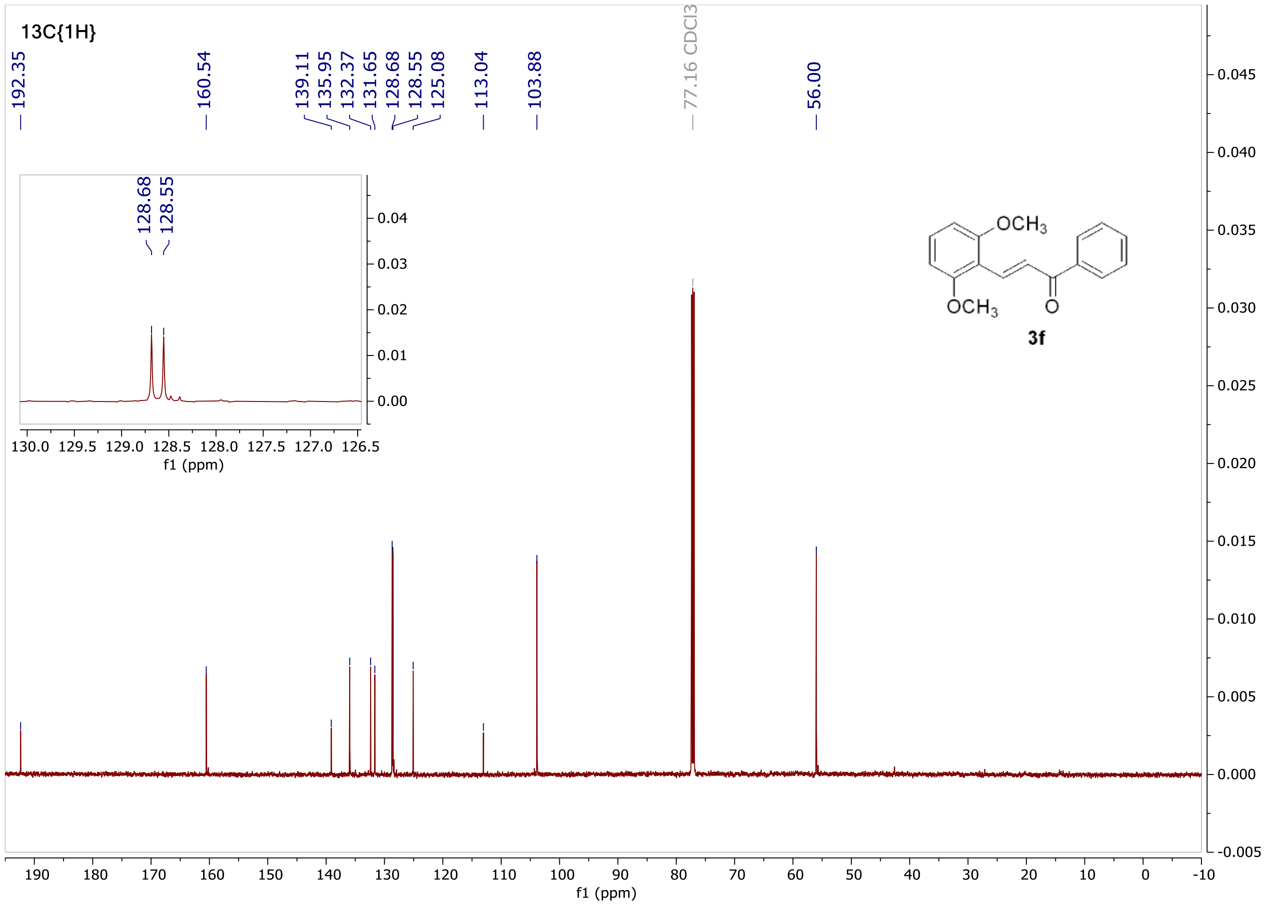

13C{1H}
192.35
160.54
139.11
135.95
132.37
131.65
128.68
128.55
125.08
113.04
103.88
77.16 CDCl3
56.00
OCH3
OCH3
O
3f
f1 (ppm)

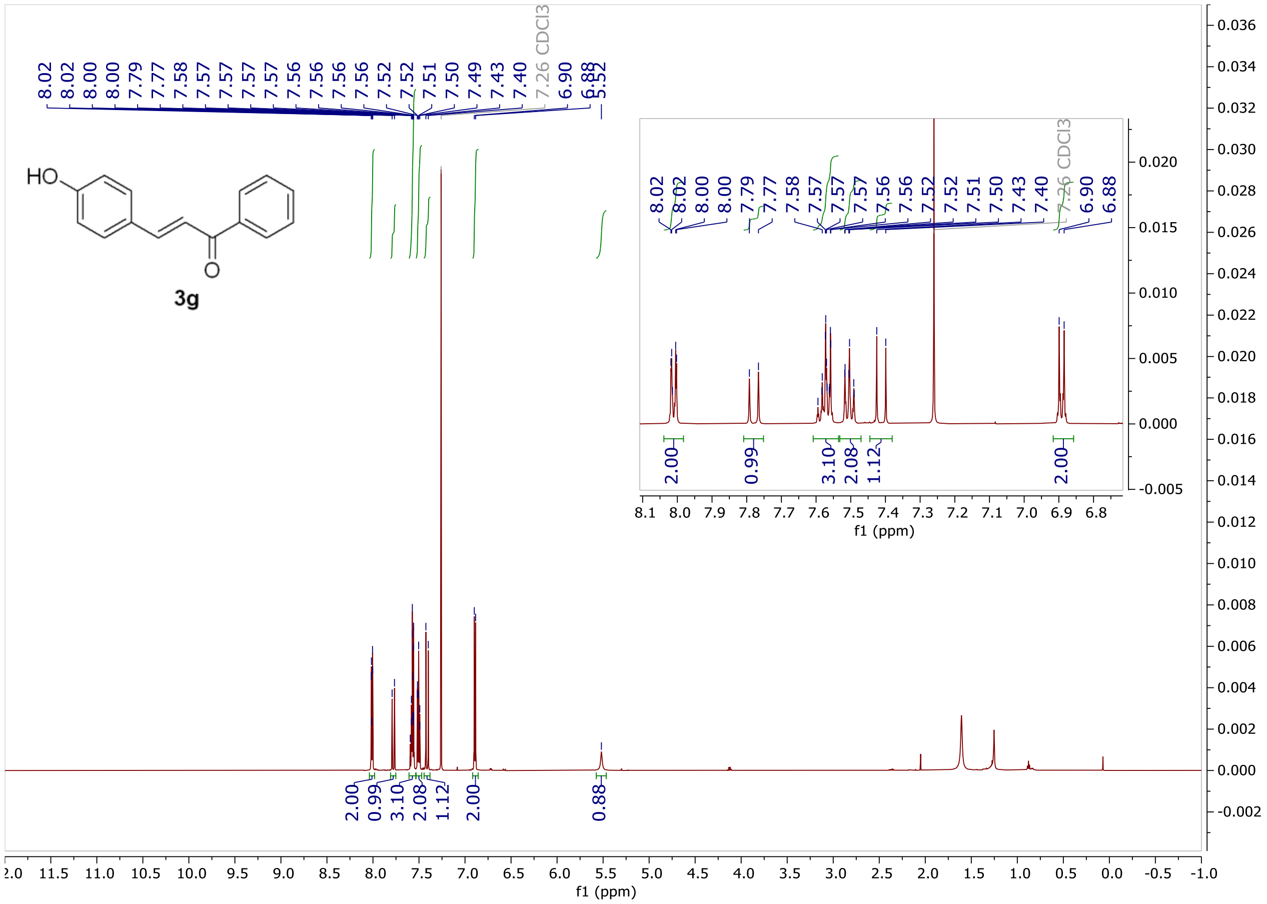
8.02
8.02
8.00
8.00
7.79
7.77
7.58
7.57
7.57
7.57
7.57
7.56
7.56
7.56
7.52
7.52
7.51
7.50
7.49
7.43
7.40
7.26 CDCl3
6.90
6.88
5.52
HO
O
3g
2.00
0.99
3.10
2.08
1.12
2.00
0.88
f1 (ppm)

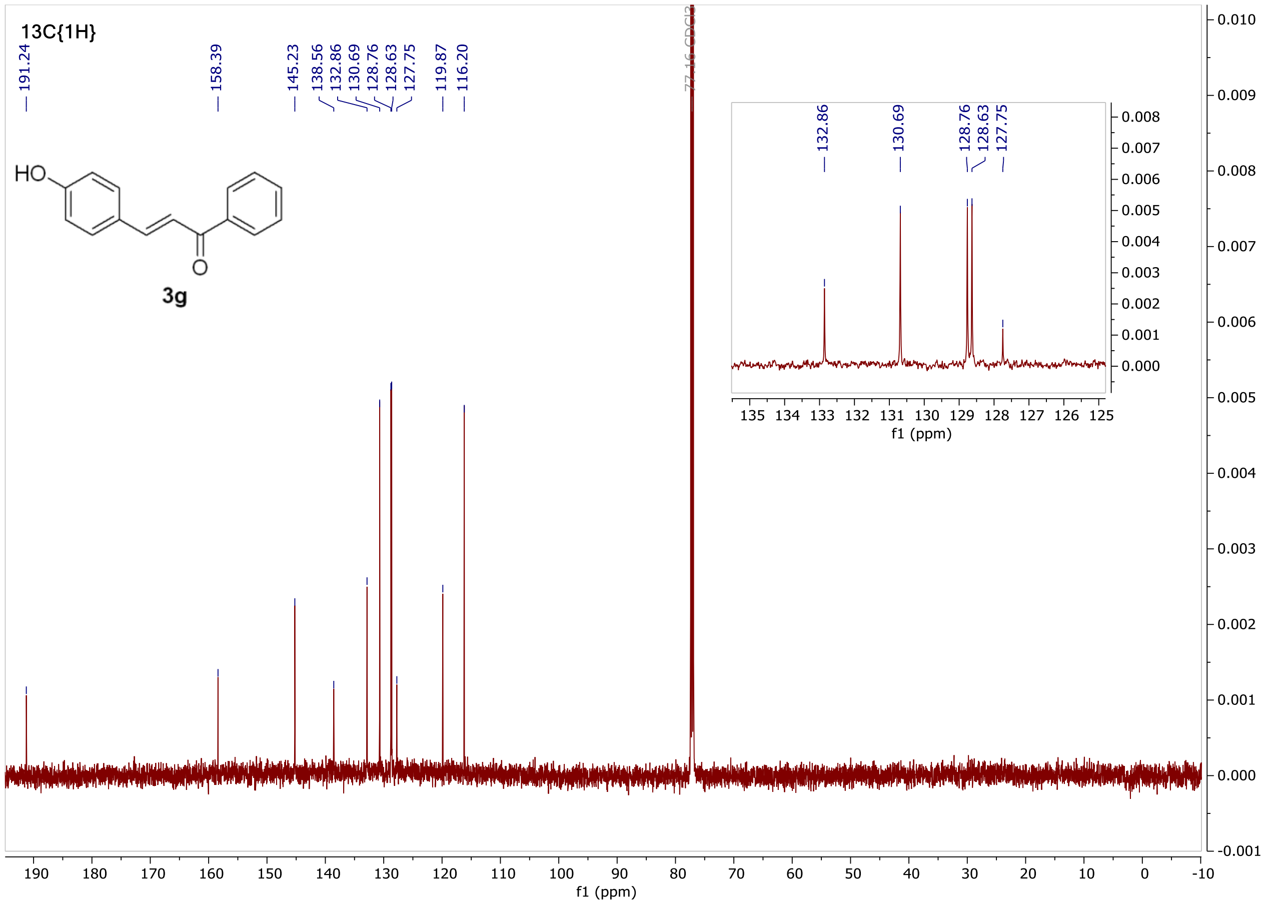
13C{1H}
191.24
158.39
145.23
138.56
132.86
130.69
128.76
128.63
127.75
119.87
116.20
77.16 CDCl3
HO
O
3g
f1 (ppm)

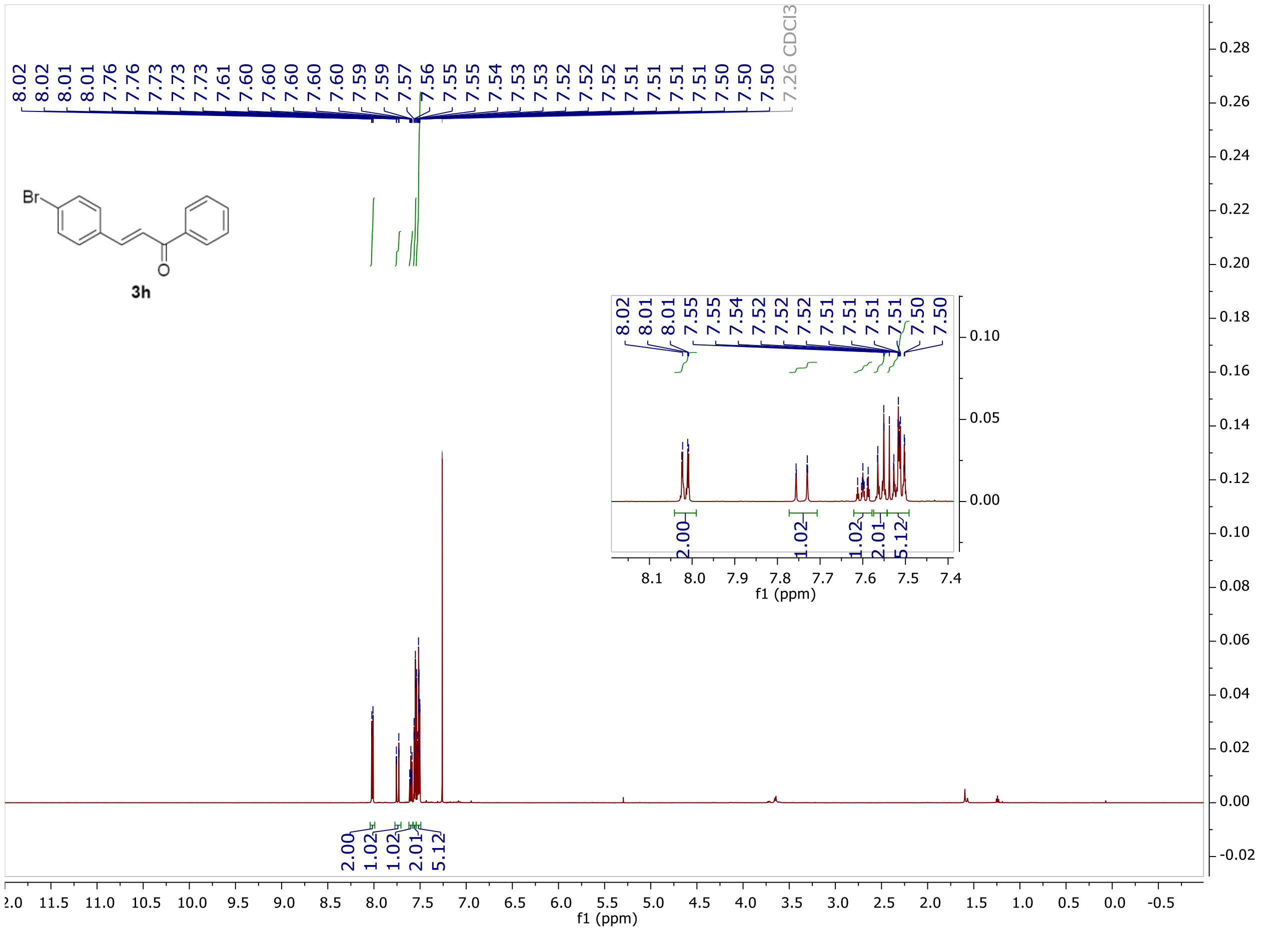
8.02 8.02 8.01 8.01 7.76 7.76 7.73 7.73 7.73 7.61 7.60 7.60 7.60 7.60 7.59 7.59 7.57 7.56 7.55 7.55 7.54 7.53 7.53 7.52 7.52 7.52 7.51 7.51 7.51 7.51 7.50 7.50 7.50
7.26 CDCl3
Br
O
3h
2.00
1.02
1.02
2.01
5.12
f1 (ppm)

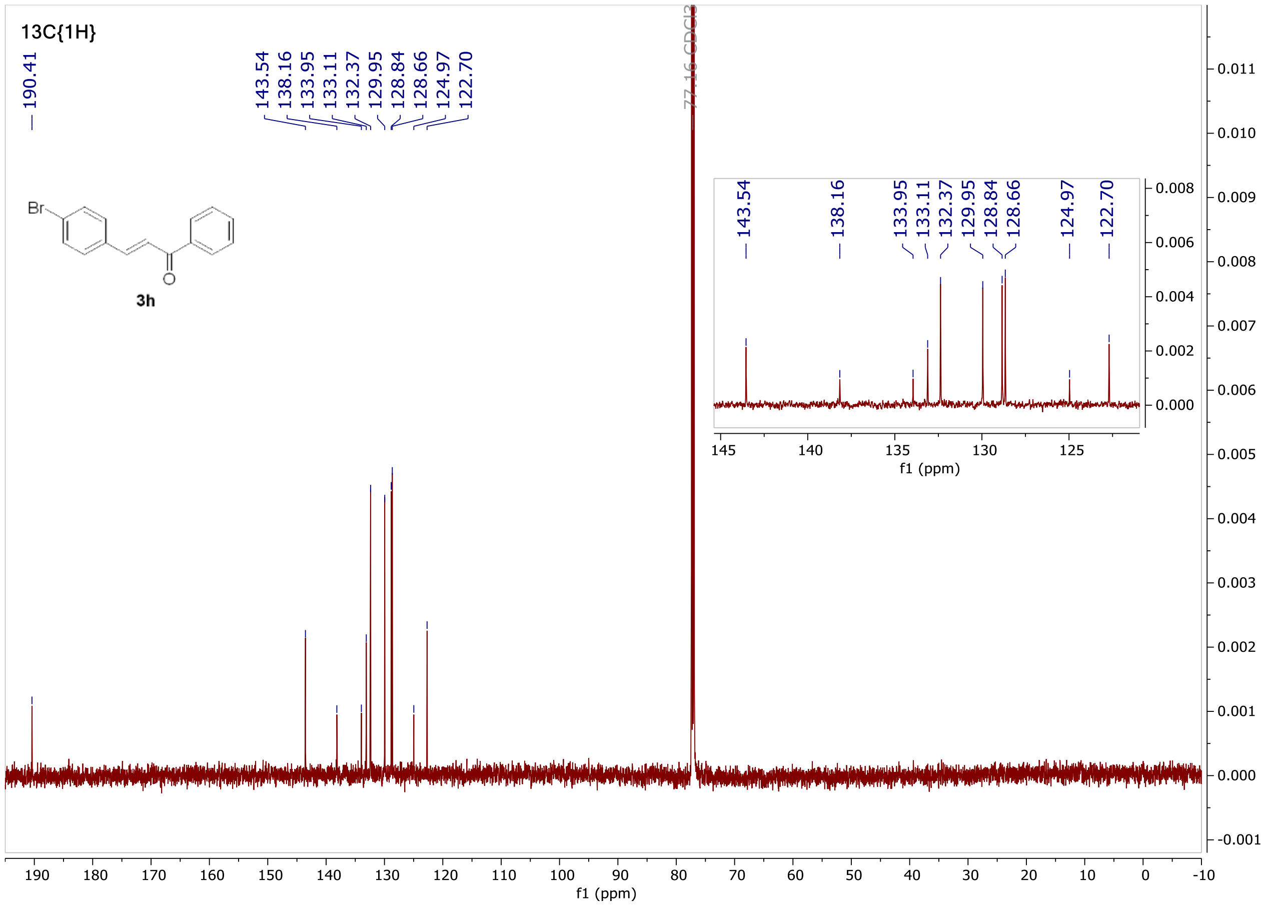
13C{1H}
190.41
143.54 138.16 133.95 133.11 132.37 129.95 128.84 128.66 124.97 122.70
77.16 CDCl3
Br
O
3h
f1 (ppm)

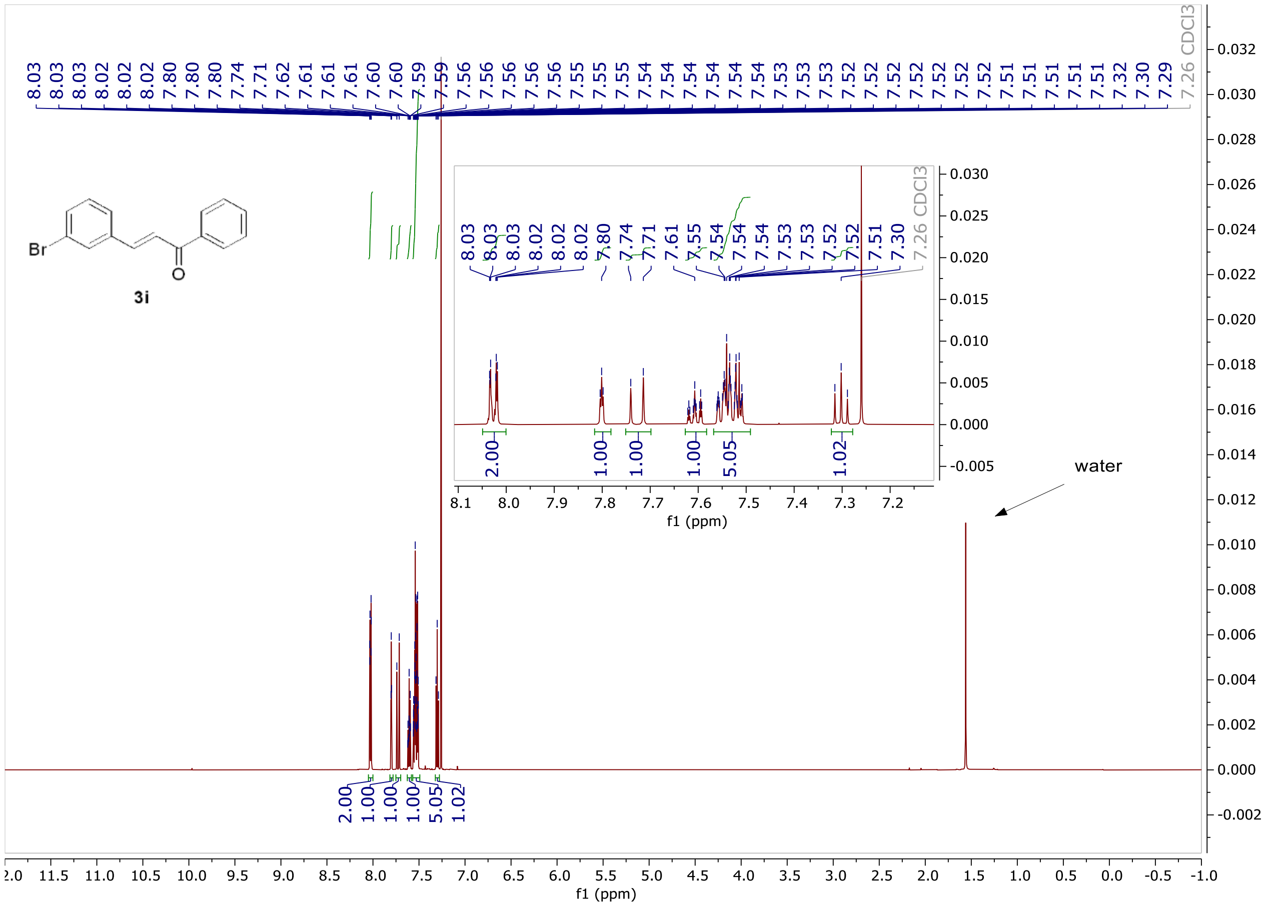
3i
water
f1 (ppm)
7.26 CDCl3

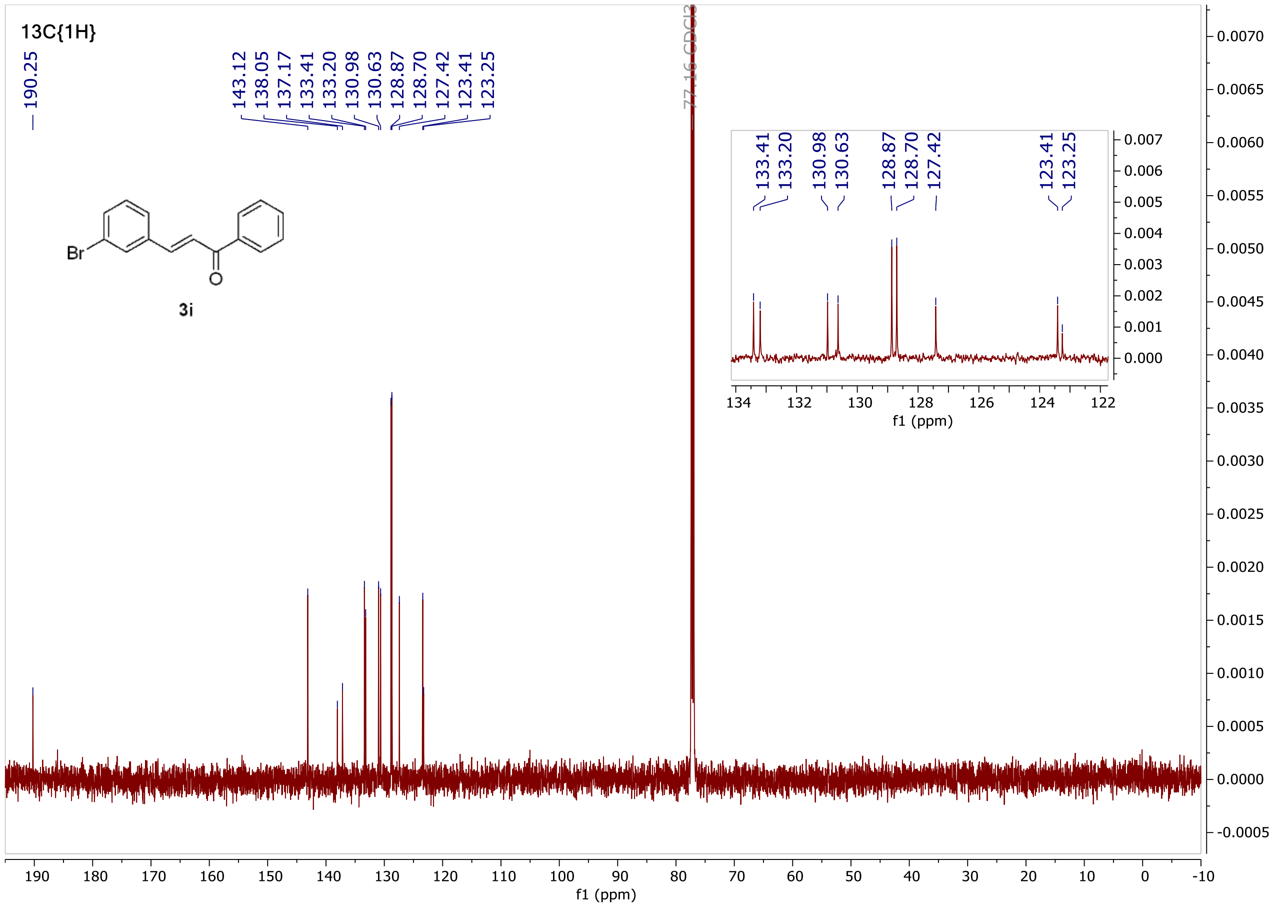
13C{1H}
190.25
143.12
138.05
137.17
133.41
133.20
130.98
130.63
128.87
128.70
127.42
123.41
123.25
77.16 CDCl3
3i
f1 (ppm)

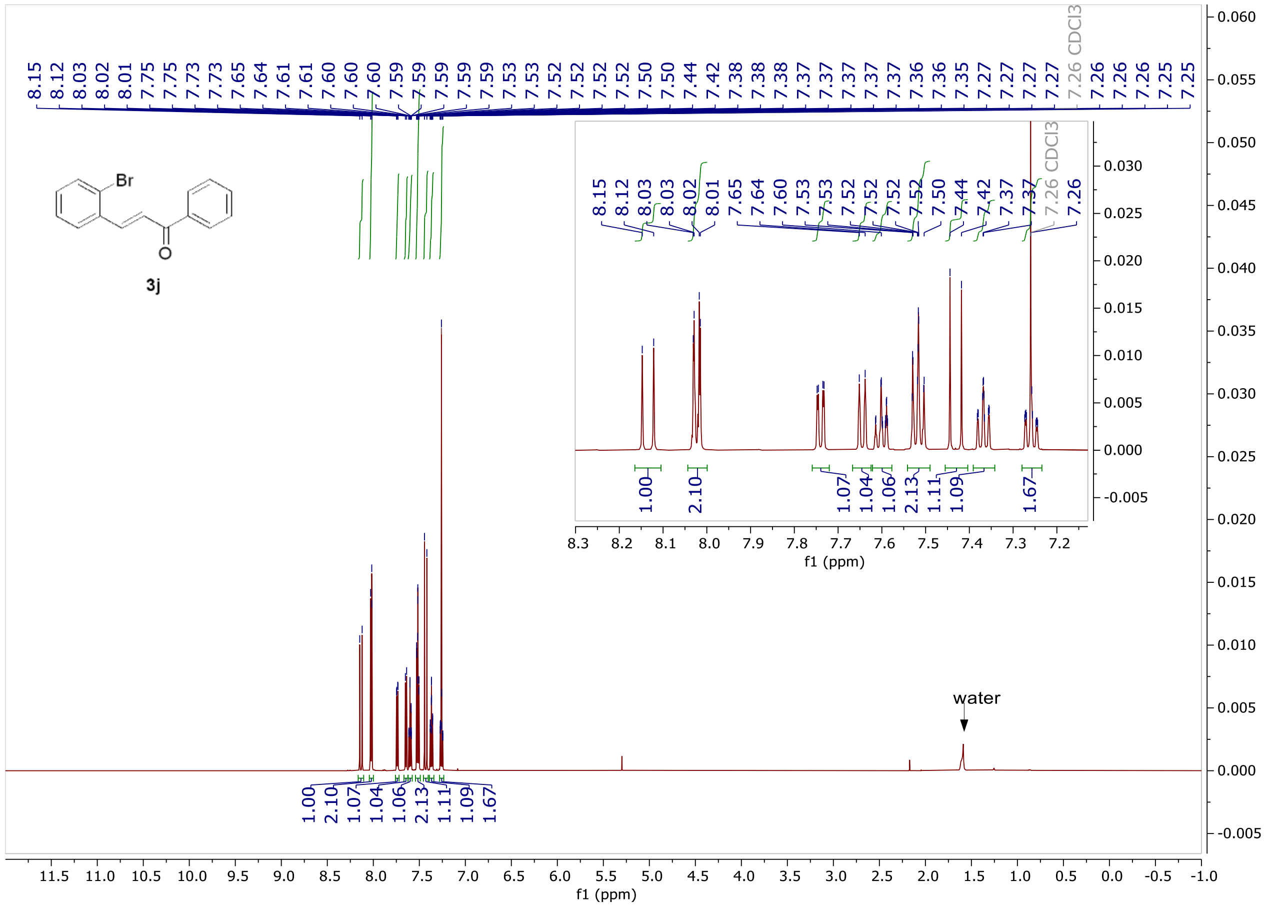

8.15
8.12
8.03
8.02
8.01
7.75
7.75
7.73
7.73
7.65
7.64
7.61
7.60
7.60
7.60
7.59
7.59
7.59
7.59
7.53
7.52
7.52
7.52
7.52
7.50
7.50
7.44
7.42
7.38
7.38
7.38
7.37
7.37
7.37
7.37
7.37
7.36
7.36
7.35
7.27
7.27
7.27
7.27
7.26 CDCl3
7.26
7.26
7.26
7.25
7.25
Br
O
3j
water
1.00
2.10
1.07
1.04
1.06
2.13
1.11
1.09
1.67
f1 (ppm)

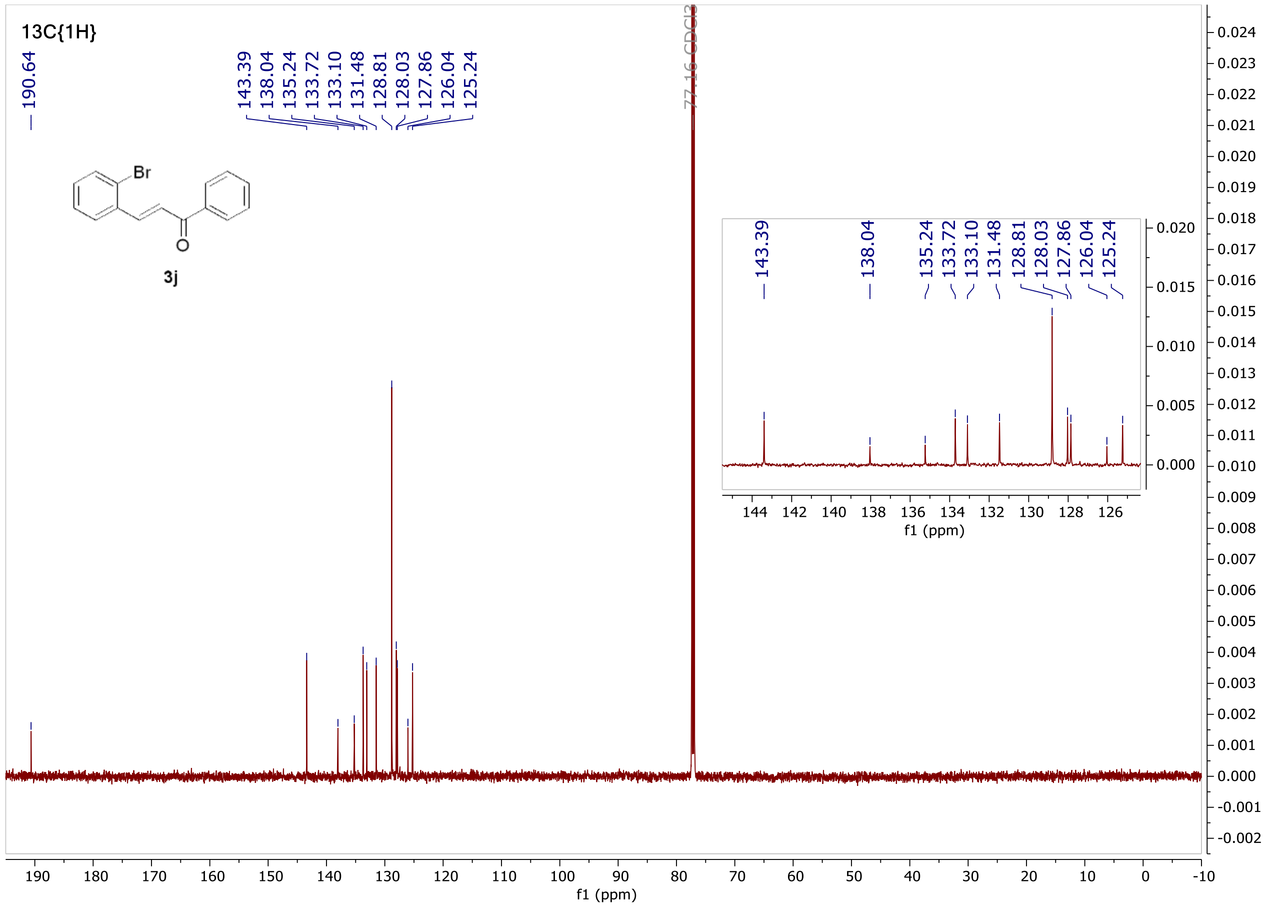

13C{1H}
190.64
143.39
138.04
135.24
133.72
133.10
131.48
128.81
128.03
127.86
126.04
125.24
77.16 CDCl3
Br
O
3j
f1 (ppm)

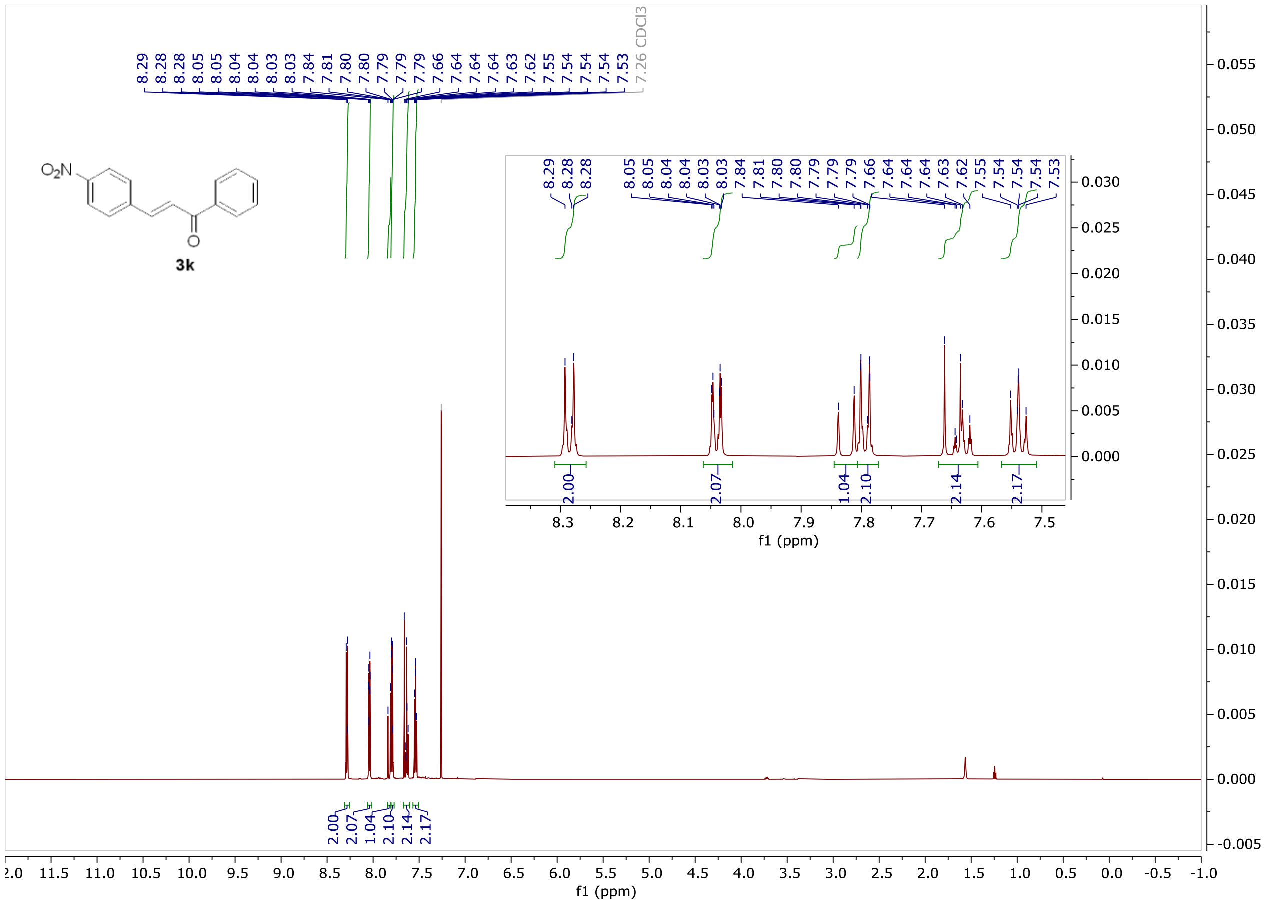
8.29 8.28 8.28 8.05 8.05 8.04 8.04 8.03 8.03 7.84 7.81 7.80 7.80 7.79 7.79 7.79 7.66 7.64 7.64 7.64 7.63 7.62 7.55 7.54 7.54 7.54 7.54 7.53
7.26 CDCl3
O2N
3k
2.00 2.07 1.04 2.10 2.14 2.17
f1 (ppm)

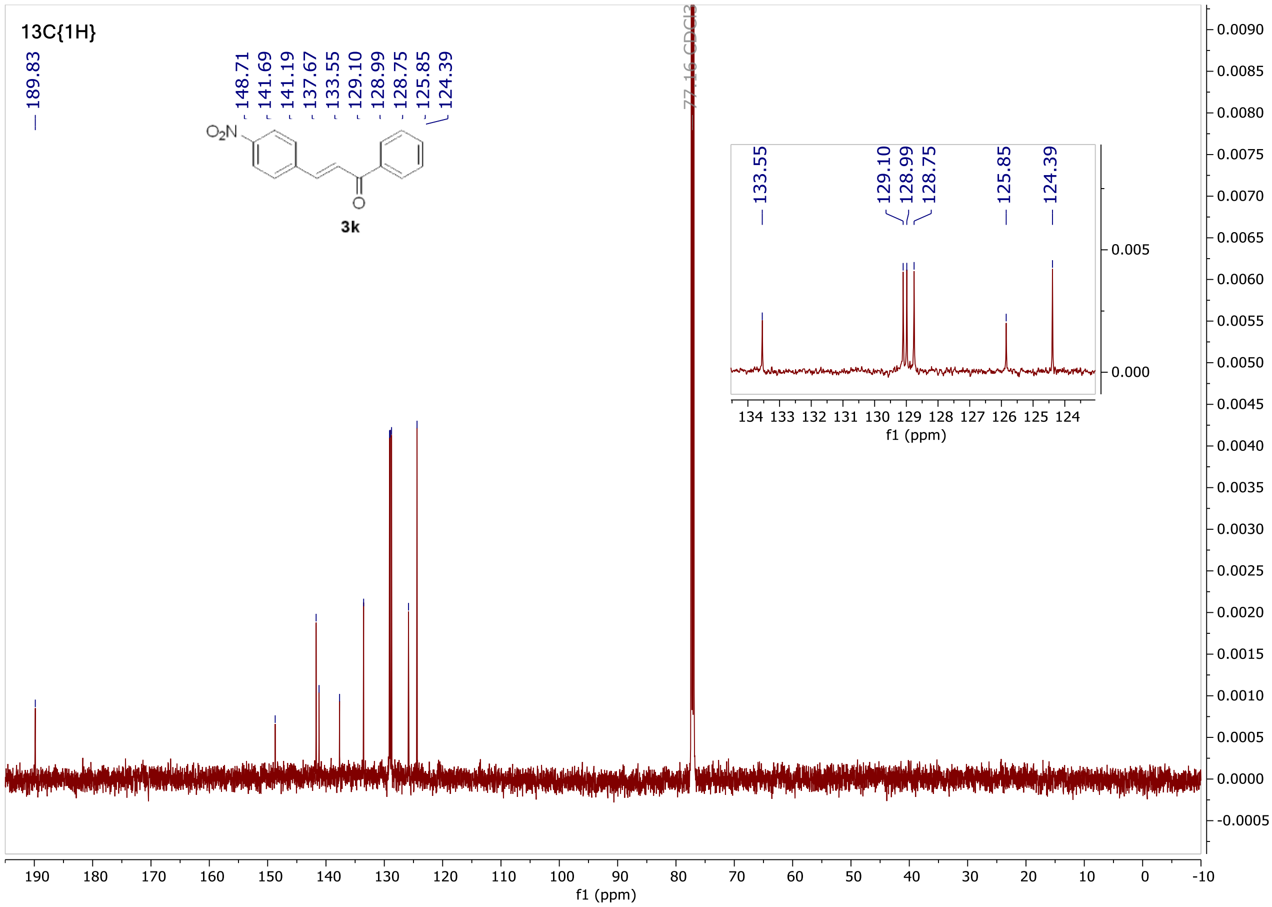
13C{1H}
189.83
148.71 141.69 141.19 137.67 133.55 129.10 128.99 128.75 125.85 124.39
77.16 CDCl3
O2N
3k
f1 (ppm)

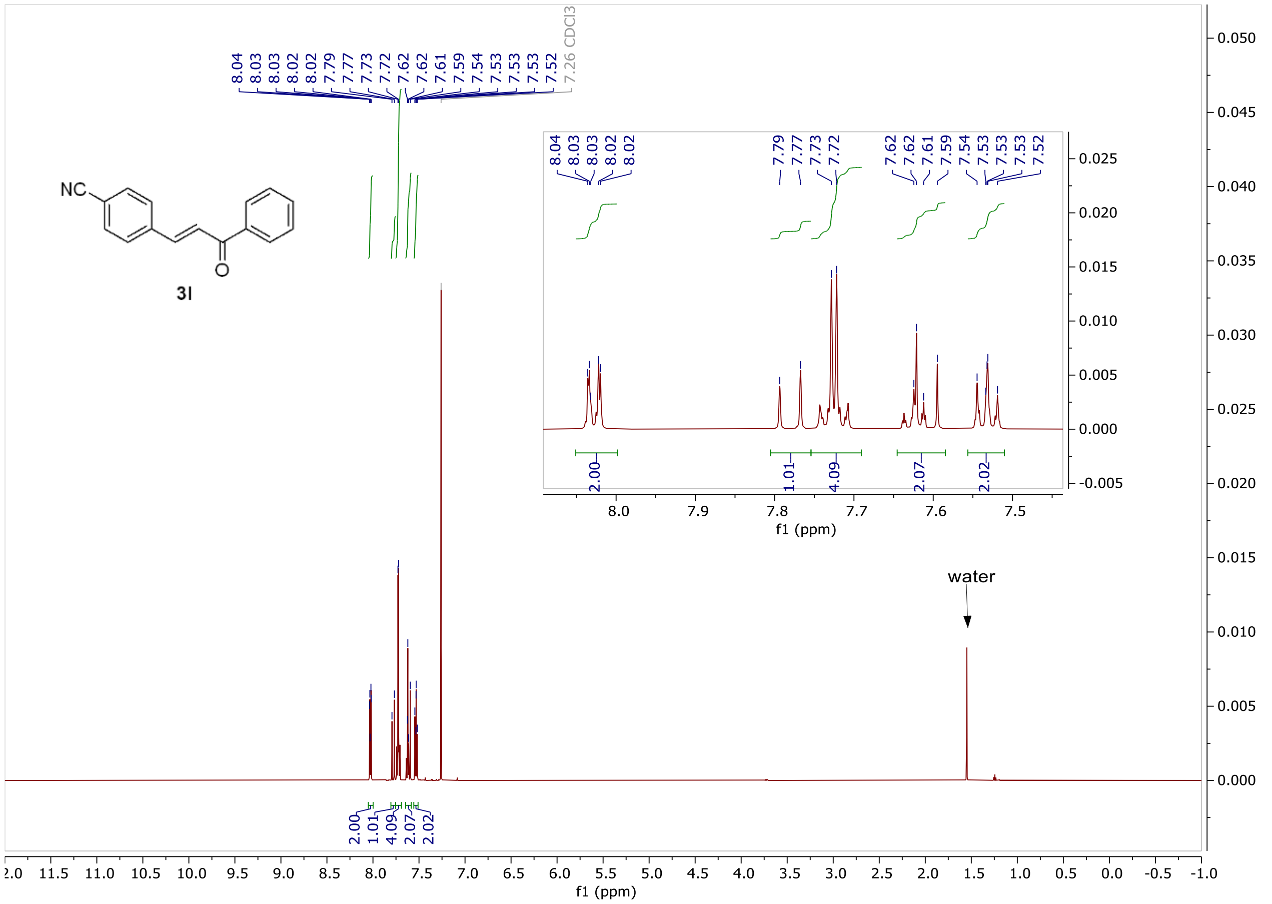
8.04
8.03
8.03
8.02
8.02
7.79
7.77
7.73
7.72
7.62
7.62
7.61
7.59
7.54
7.53
7.53
7.53
7.52
7.26 CDCl3
NC
O
3l
water
2.00
1.01
4.09
2.07
2.02
f1 (ppm)

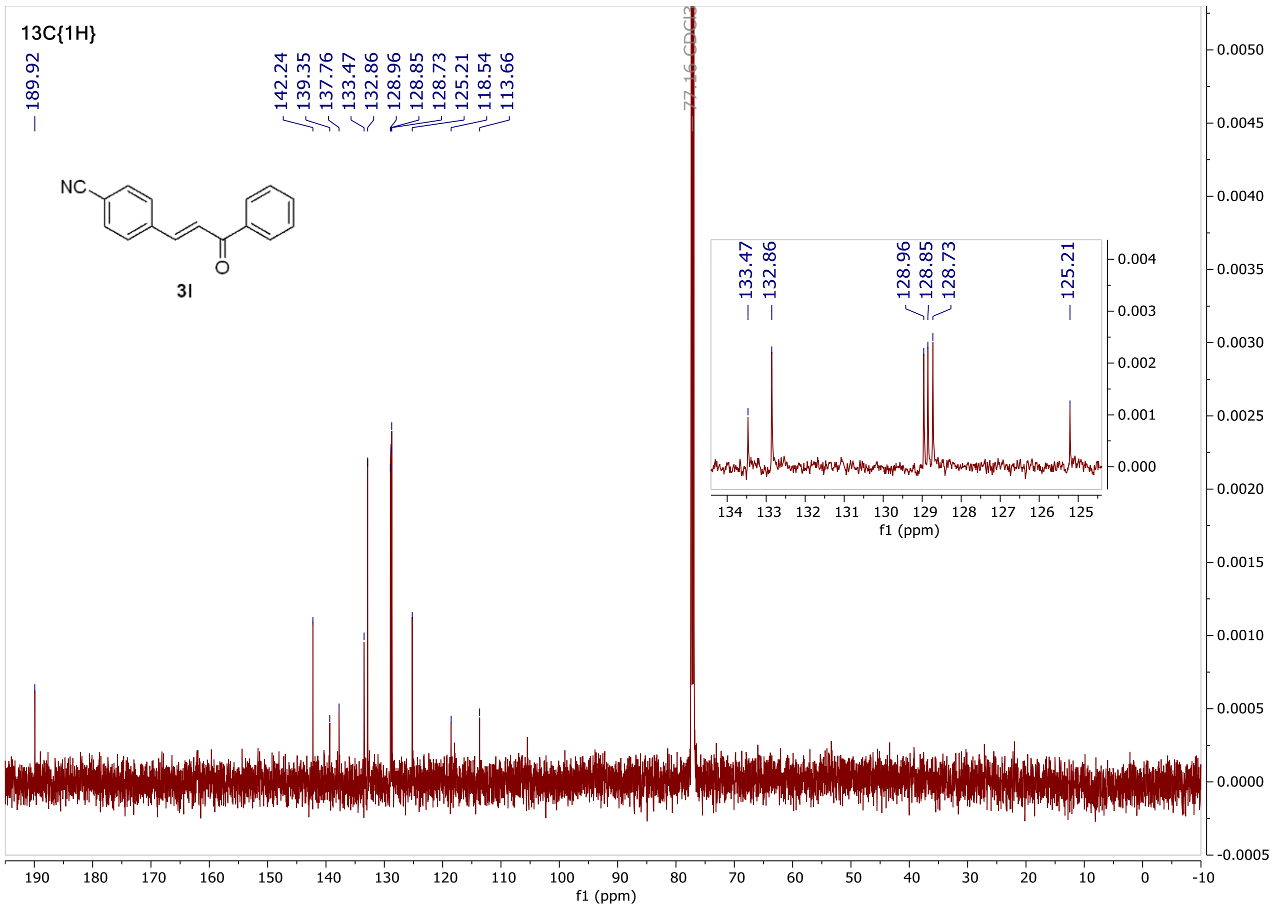
13C{1H}
189.92
142.24
139.35
137.76
133.47
132.86
128.96
128.85
128.73
125.21
118.54
113.66
77.16 CDCl3
NC
O
3l
f1 (ppm)

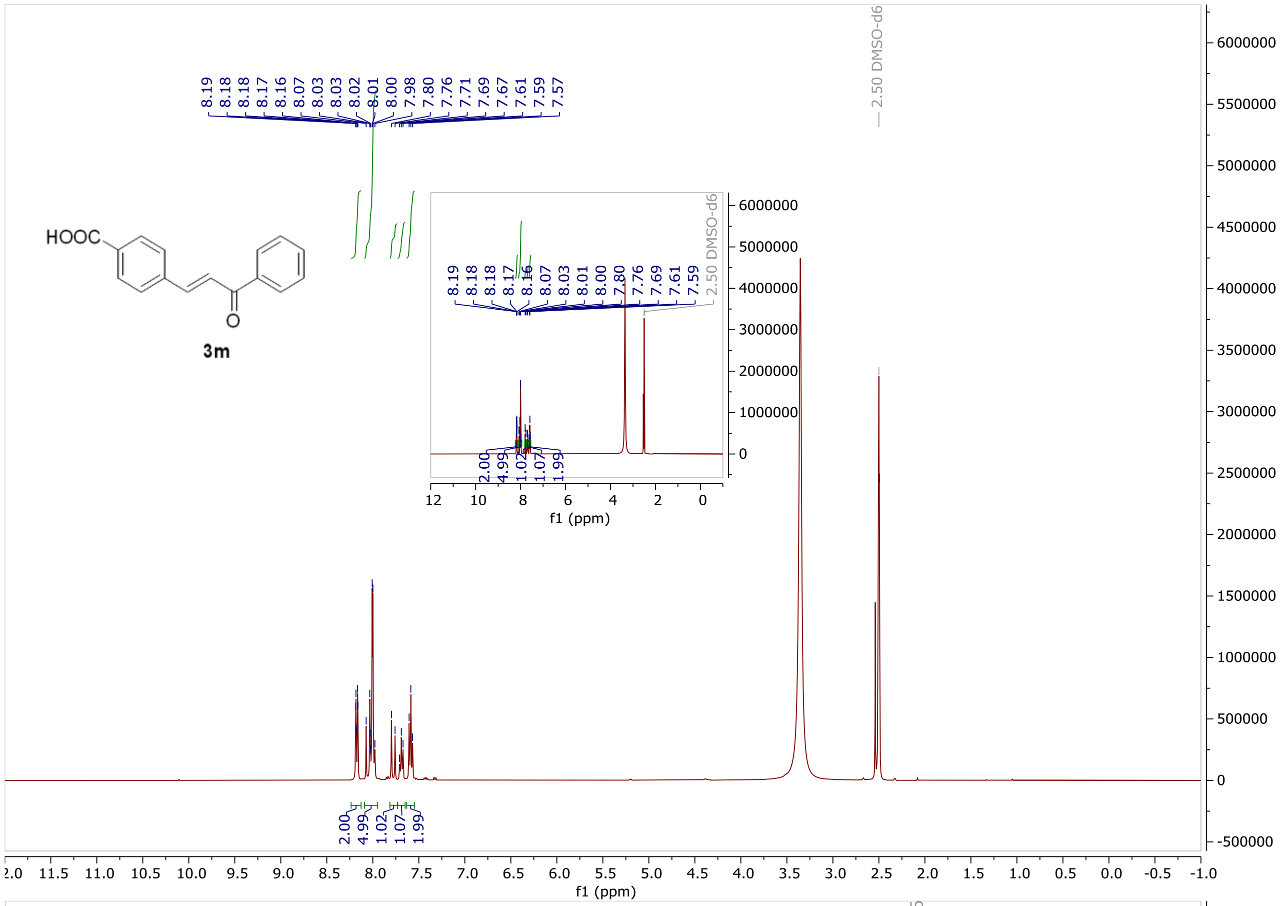
HOOC
3m
8.19 8.18 8.18 8.17 8.16 8.07 8.03 8.03 8.02 8.01 8.00 7.98 7.80 7.76 7.71 7.69 7.67 7.61 7.59 7.57
2.50 DMSO-d6
2.00 4.99 1.02 1.07 1.99
f1 (ppm)

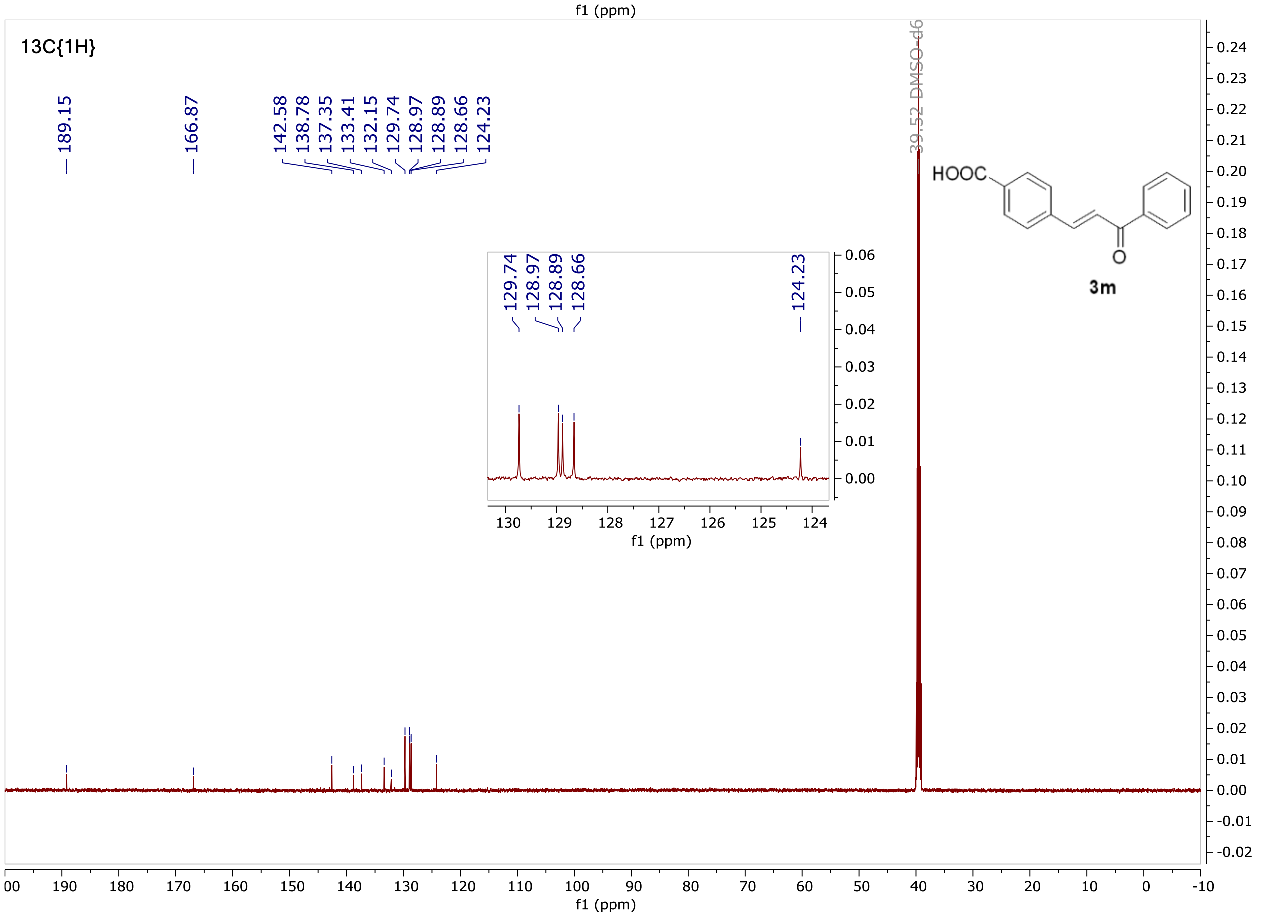
13C{1H}
189.15
166.87
142.58 138.78 137.35 133.41 132.15 129.74 128.97 128.89 128.66 124.23
39.52 DMSO-d6
HOOC
3m
f1 (ppm)

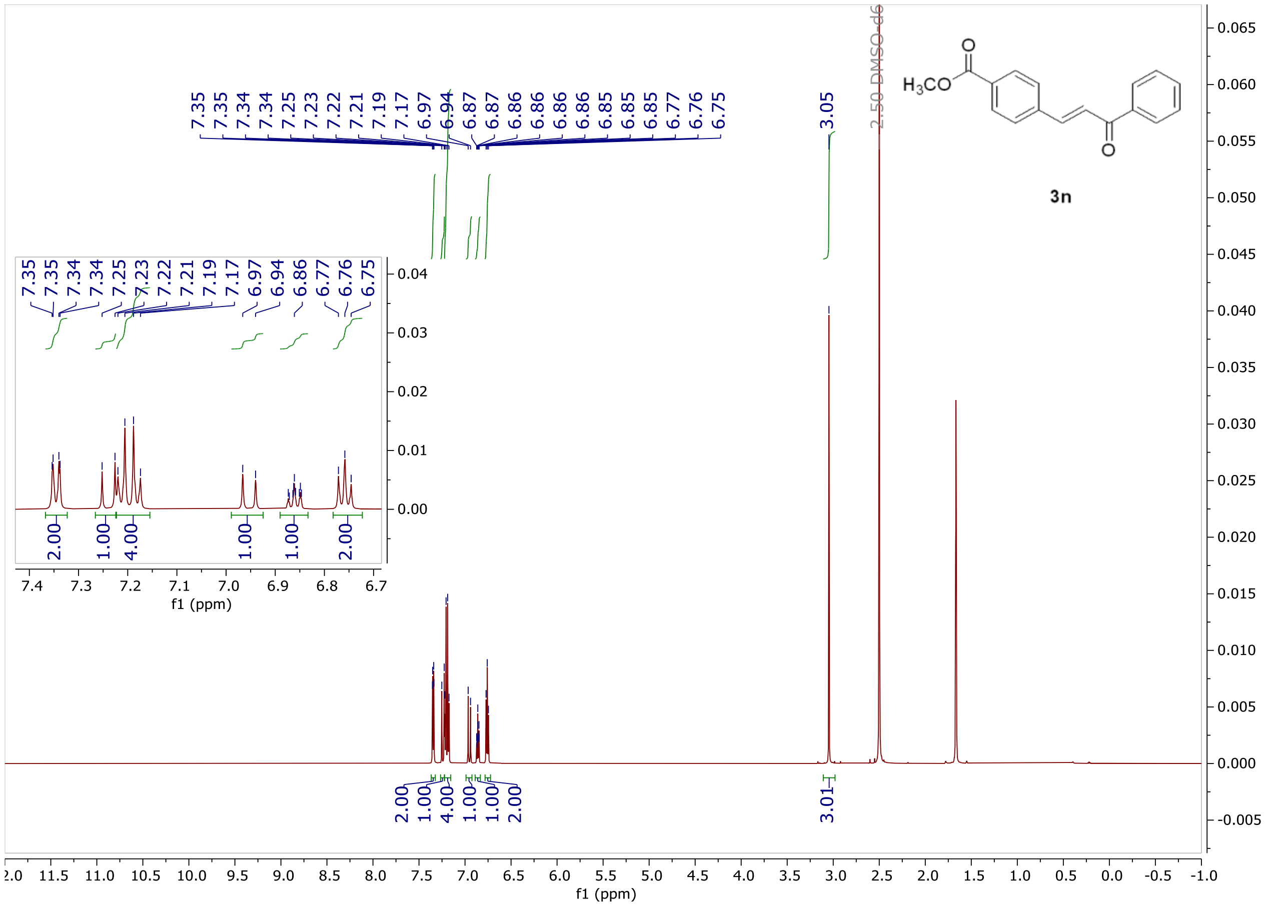
7.35
7.35
7.34
7.34
7.25
7.23
7.22
7.21
7.19
7.17
6.97
6.94
6.87
6.87
6.86
6.86
6.86
6.85
6.85
6.85
6.77
6.76
6.75
3.05
2.50 DMSO-d6
H3CO
3n
2.00
1.00
4.00
1.00
1.00
2.00
3.01
f1 (ppm)

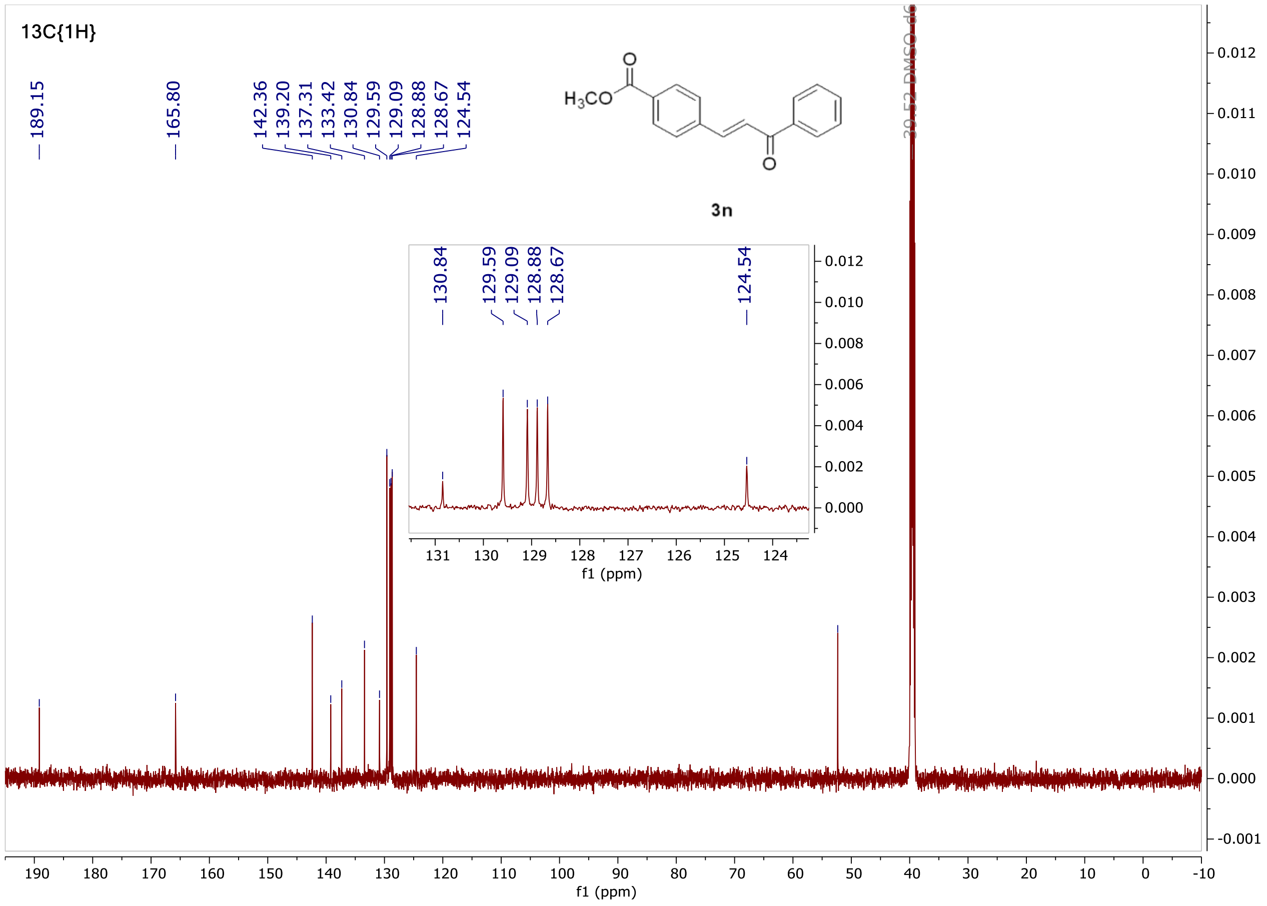
13C{1H}
189.15
165.80
142.36
139.20
137.31
133.42
130.84
129.59
129.09
128.88
128.67
124.54
39.52 DMSO-d6
H3CO
3n
f1 (ppm)

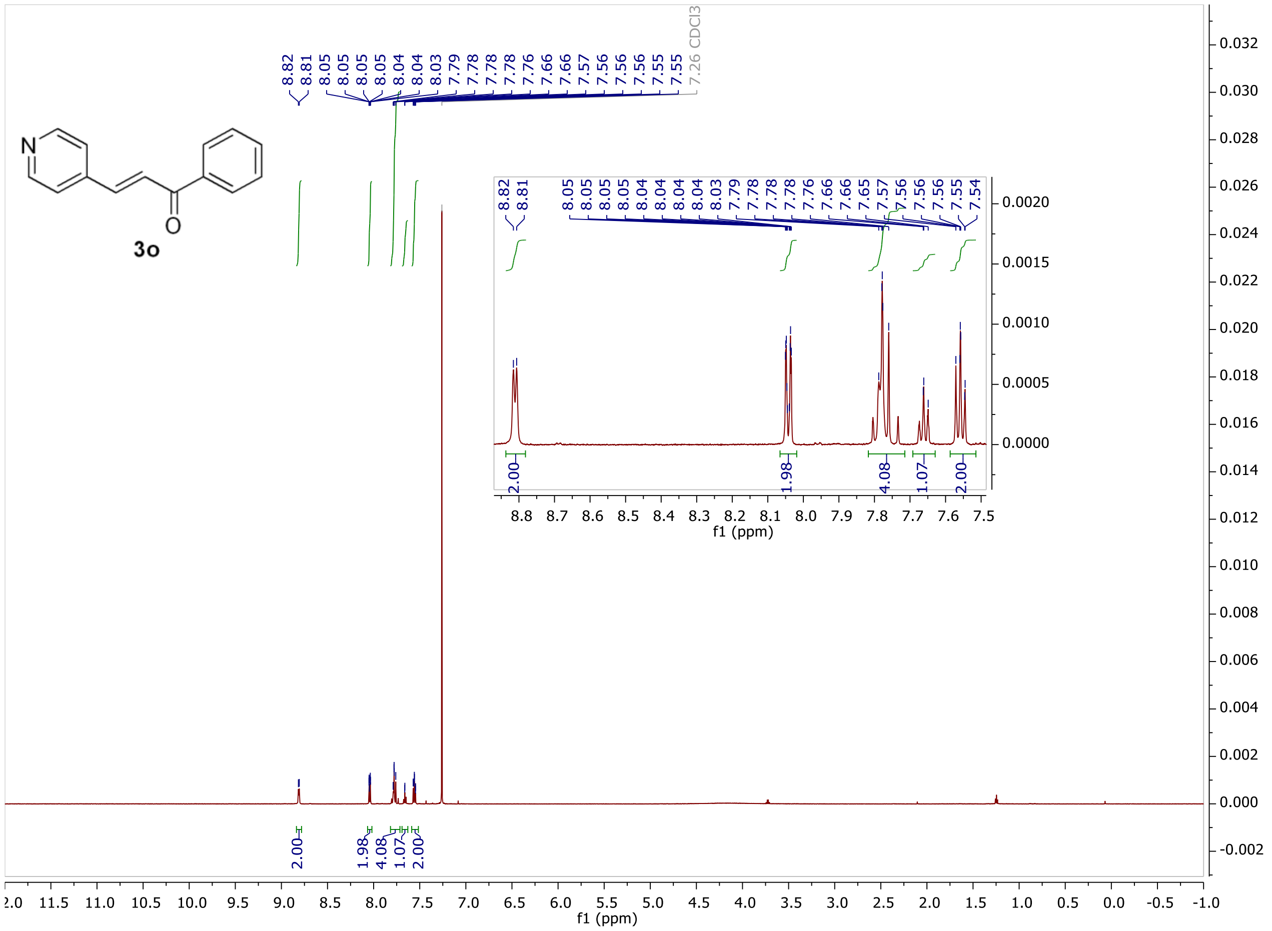
3o
8.82 8.81 8.05 8.05 8.05 8.05 8.04 8.04 8.03 7.79 7.78 7.78 7.78 7.76 7.66 7.66 7.57 7.56 7.56 7.56 7.55 7.55
7.26 CDCl3
2.00 1.98 4.08 1.07 2.00
f1 (ppm)

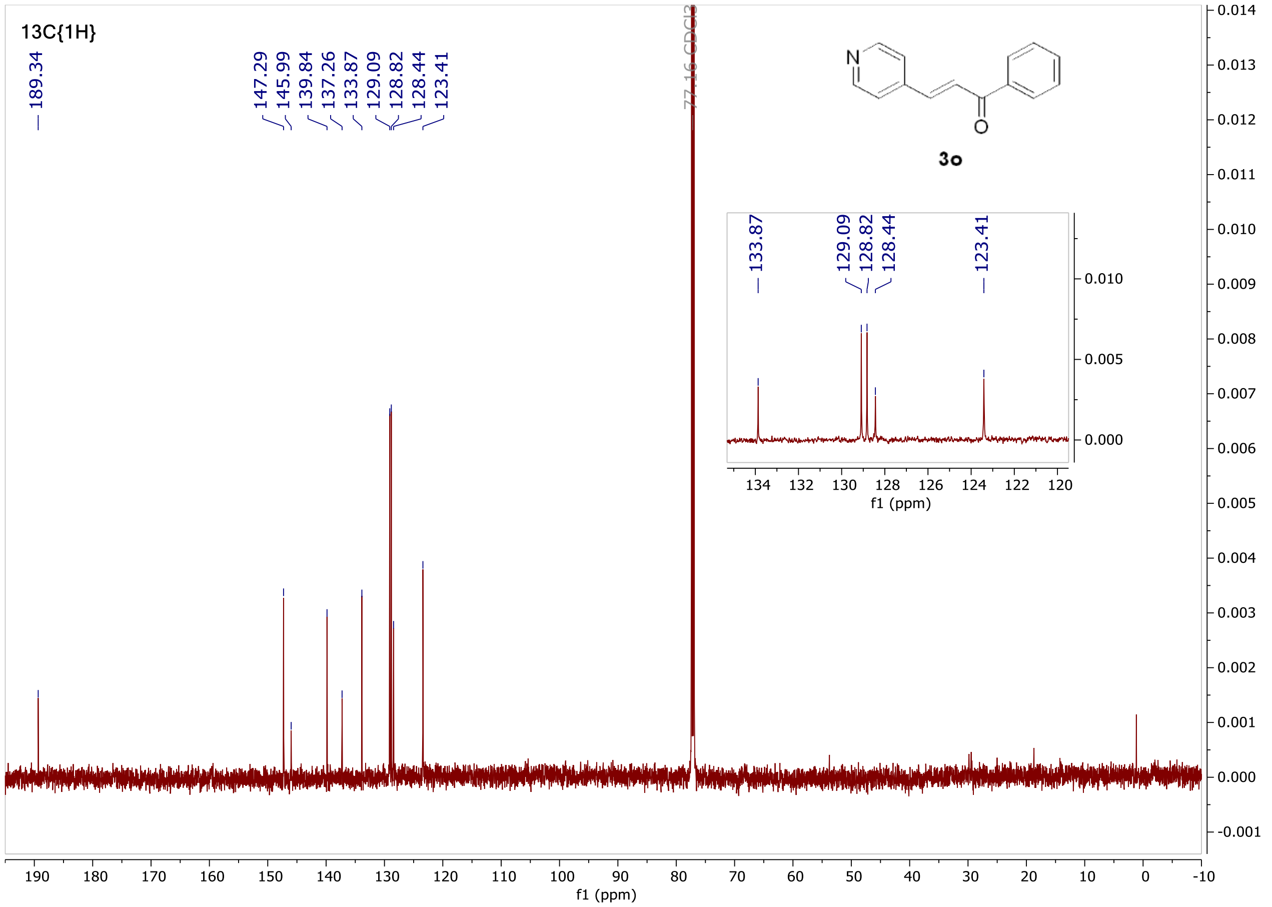
13C{1H}
189.34
147.29 145.99 139.84 137.26 133.87 129.09 128.82 128.44 123.41
77.16 CDCl3
3o
f1 (ppm)

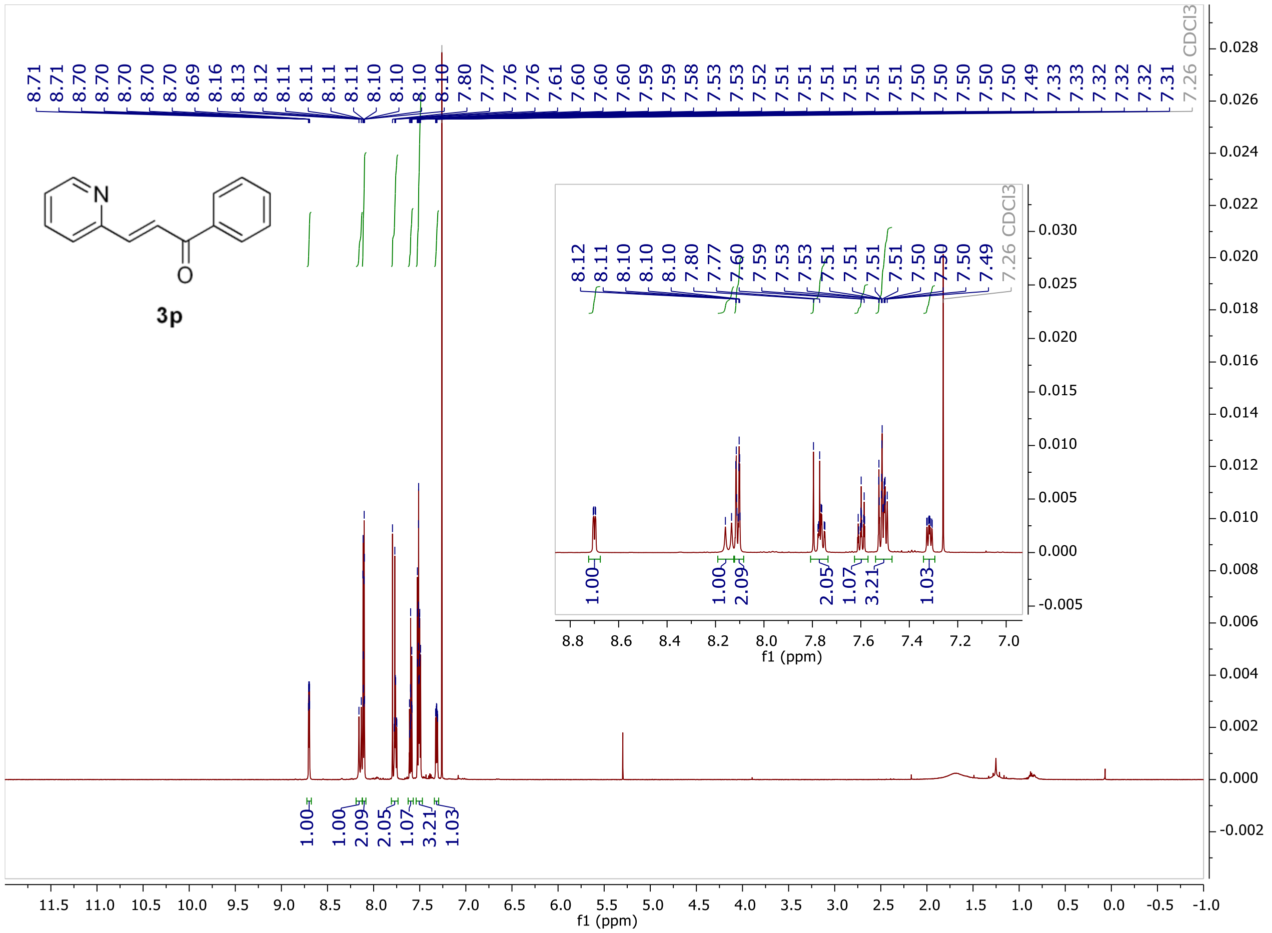

3p
8.71 8.71 8.70 8.70 8.70 8.70 8.70 8.69 8.16 8.13 8.12 8.11 8.11 8.11 8.11 8.10 8.10 8.10 8.10 7.80 7.77 7.76 7.61 7.60 7.60 7.60 7.59 7.59 7.58 7.53 7.53 7.52 7.51 7.51 7.51 7.51 7.51 7.51 7.50 7.50 7.50 7.50 7.50 7.49 7.33 7.33 7.32 7.32 7.32 7.31
7.26 CDCl3
1.00 1.00 2.09 2.05 1.07 3.21 1.03
f1 (ppm)

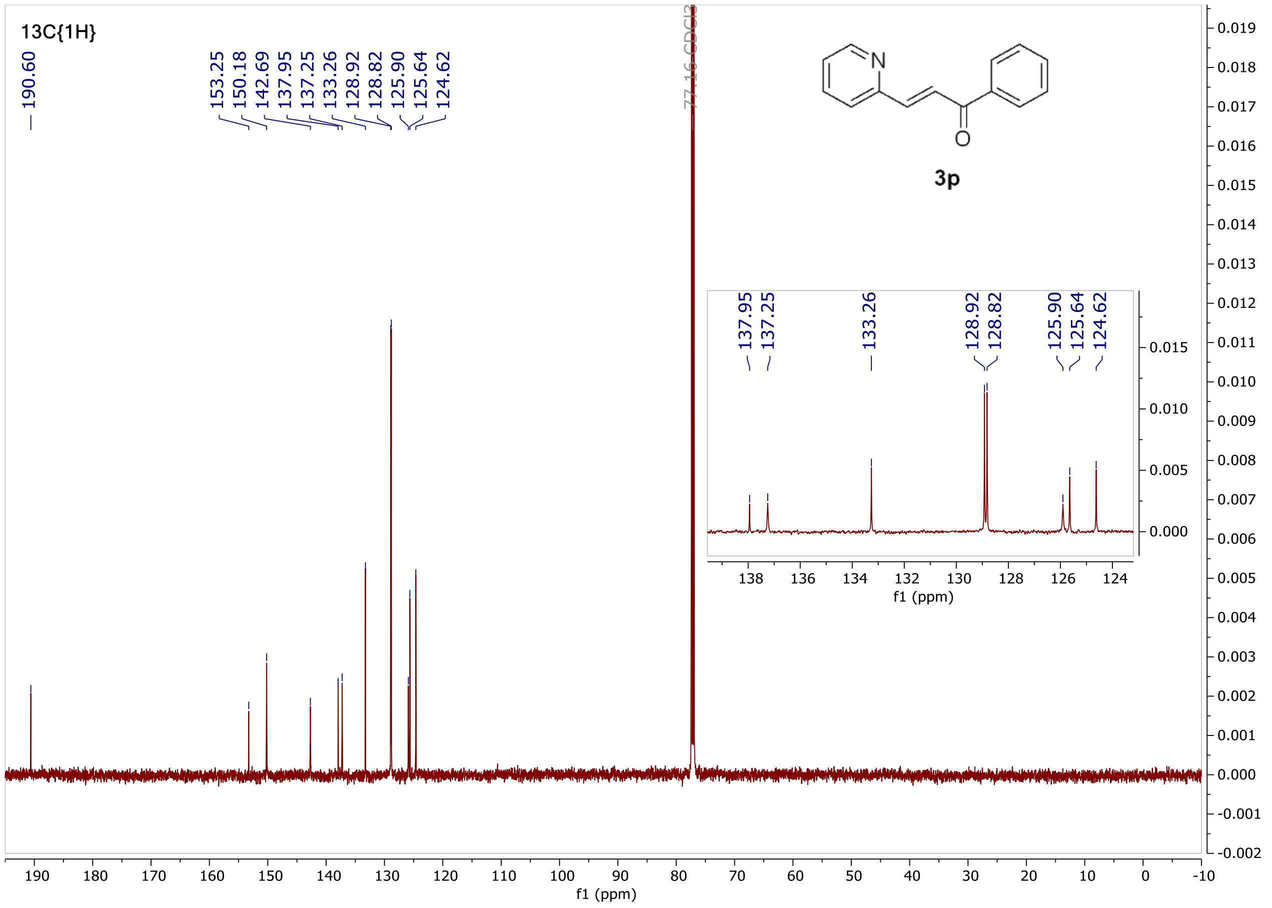

13C{1H}
190.60
153.25 150.18 142.69 137.95 137.25 133.26 128.92 128.82 125.90 125.64 124.62
77.16 CDCl3
3p
f1 (ppm)

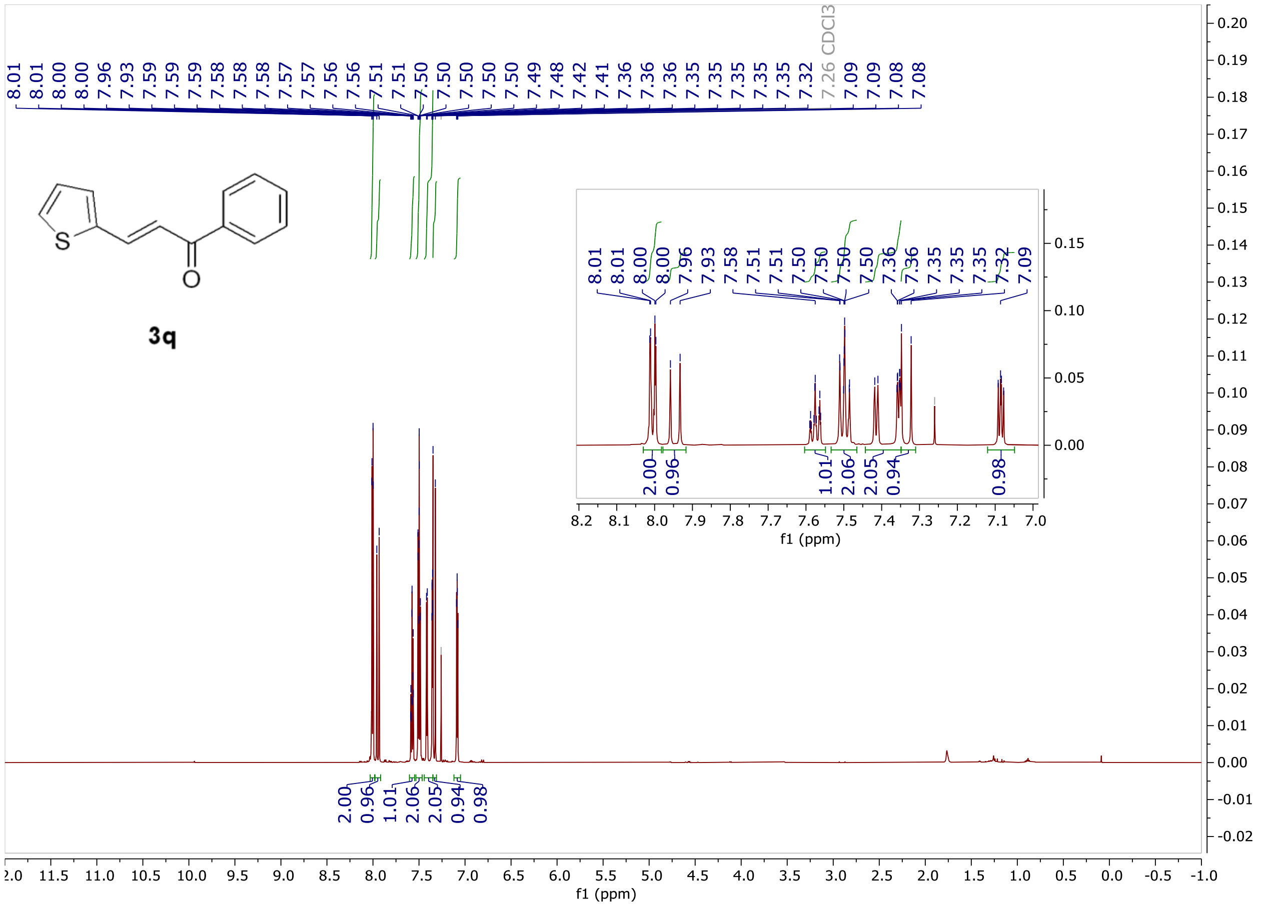
8.01 8.01 8.00 8.00 7.96 7.93 7.59 7.59 7.59 7.58 7.58 7.58 7.57 7.56 7.56 7.51 7.51 7.50 7.50 7.50 7.50 7.50 7.49 7.48 7.42 7.41 7.36 7.36 7.36 7.35 7.35 7.35 7.35 7.35 7.32 7.26 CDCl3 7.09 7.09 7.09 7.08 7.08
S
O
3q
2.00 0.96 1.01 2.06 2.05 0.94 0.98
f1 (ppm)

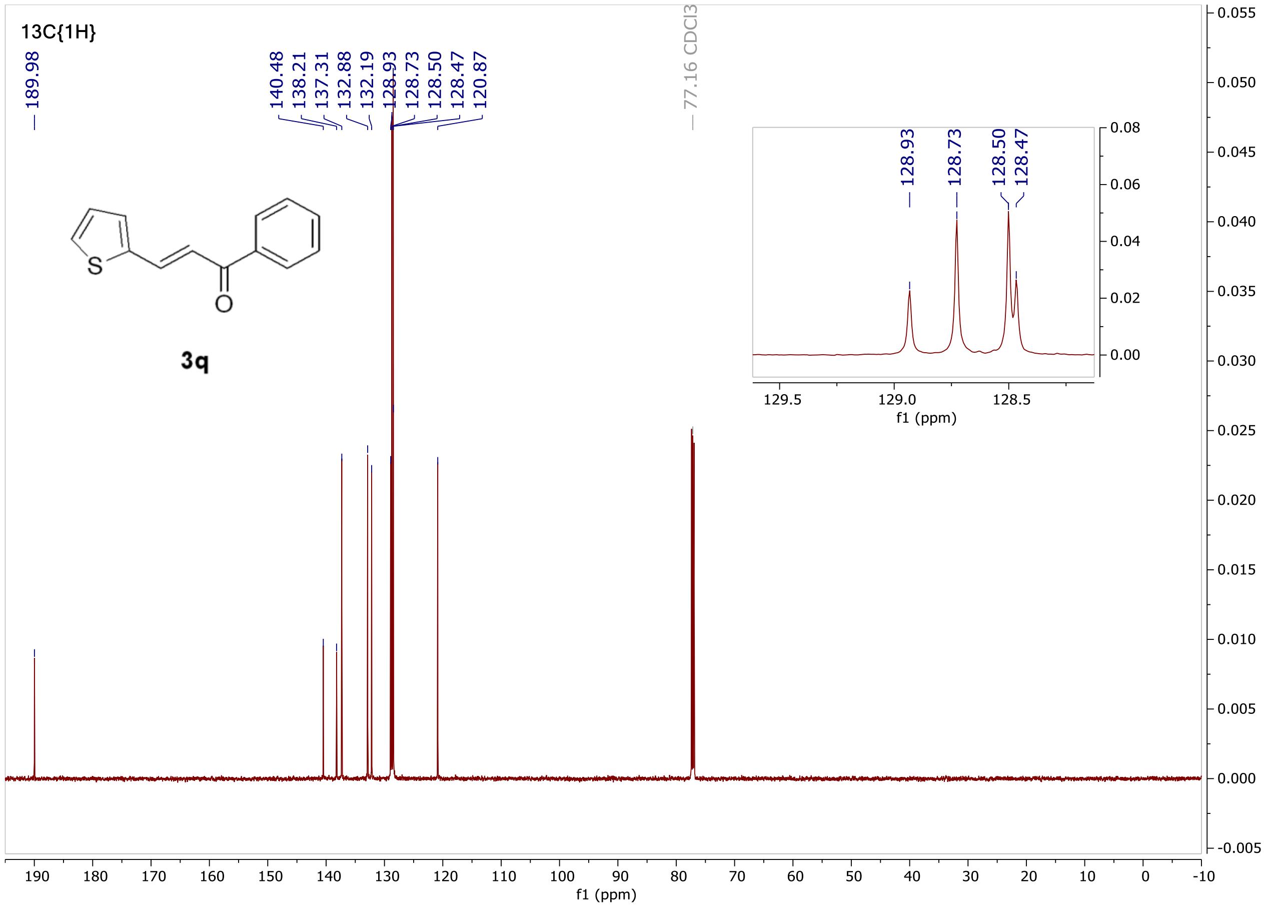
13C{1H}
189.98
140.48 138.21 137.31 132.88 132.19 128.93 128.73 128.50 128.47 120.87
77.16 CDCl3
S
O
3q
f1 (ppm)

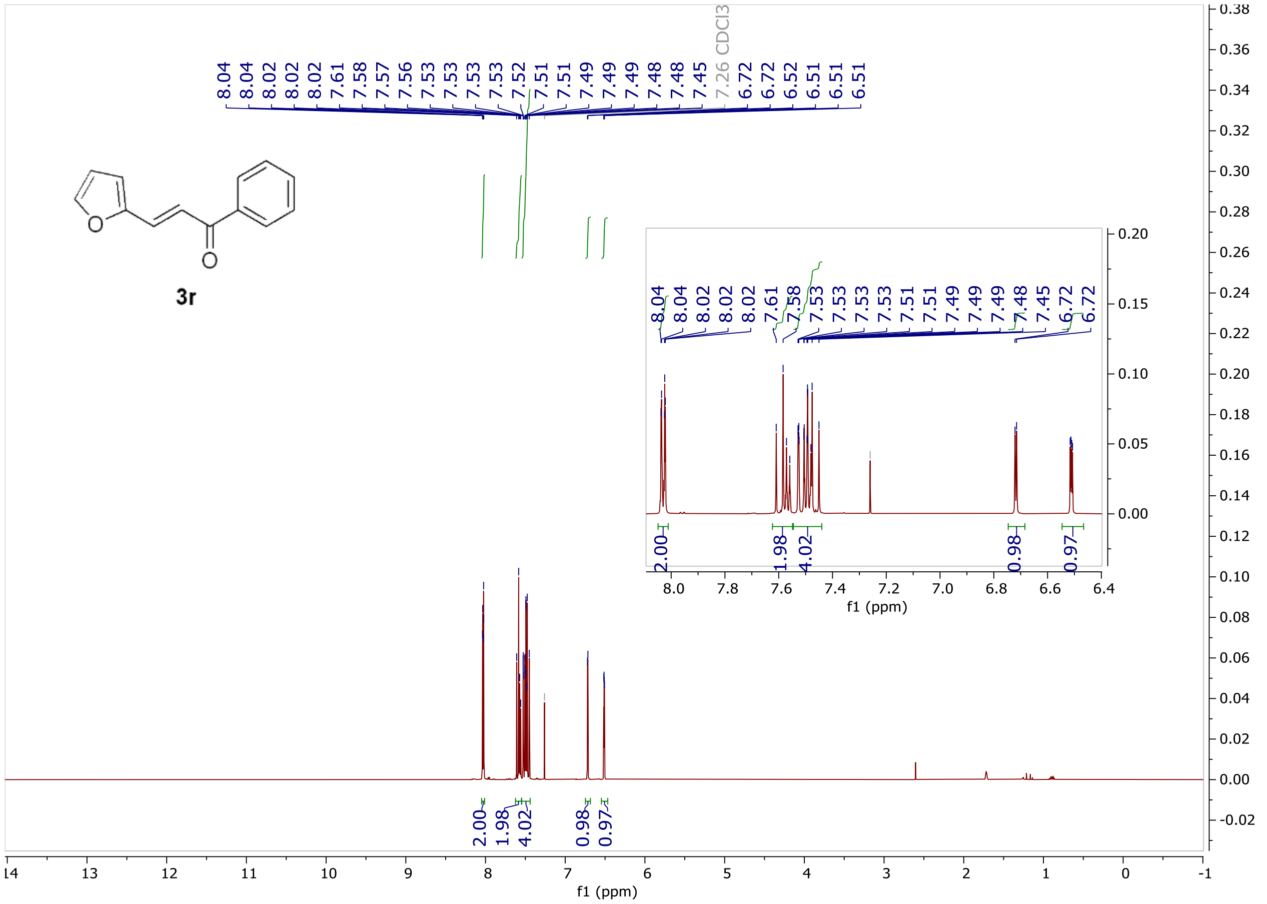
8.04 8.04 8.02 8.02 8.02 7.61 7.58 7.57 7.56 7.53 7.53 7.53 7.53 7.52 7.51 7.51 7.49 7.49 7.49 7.48 7.48 7.45 7.26 CDCl3 6.72 6.72 6.52 6.51 6.51 6.51
3r
2.00 1.98 4.02 0.98 0.97
f1 (ppm)

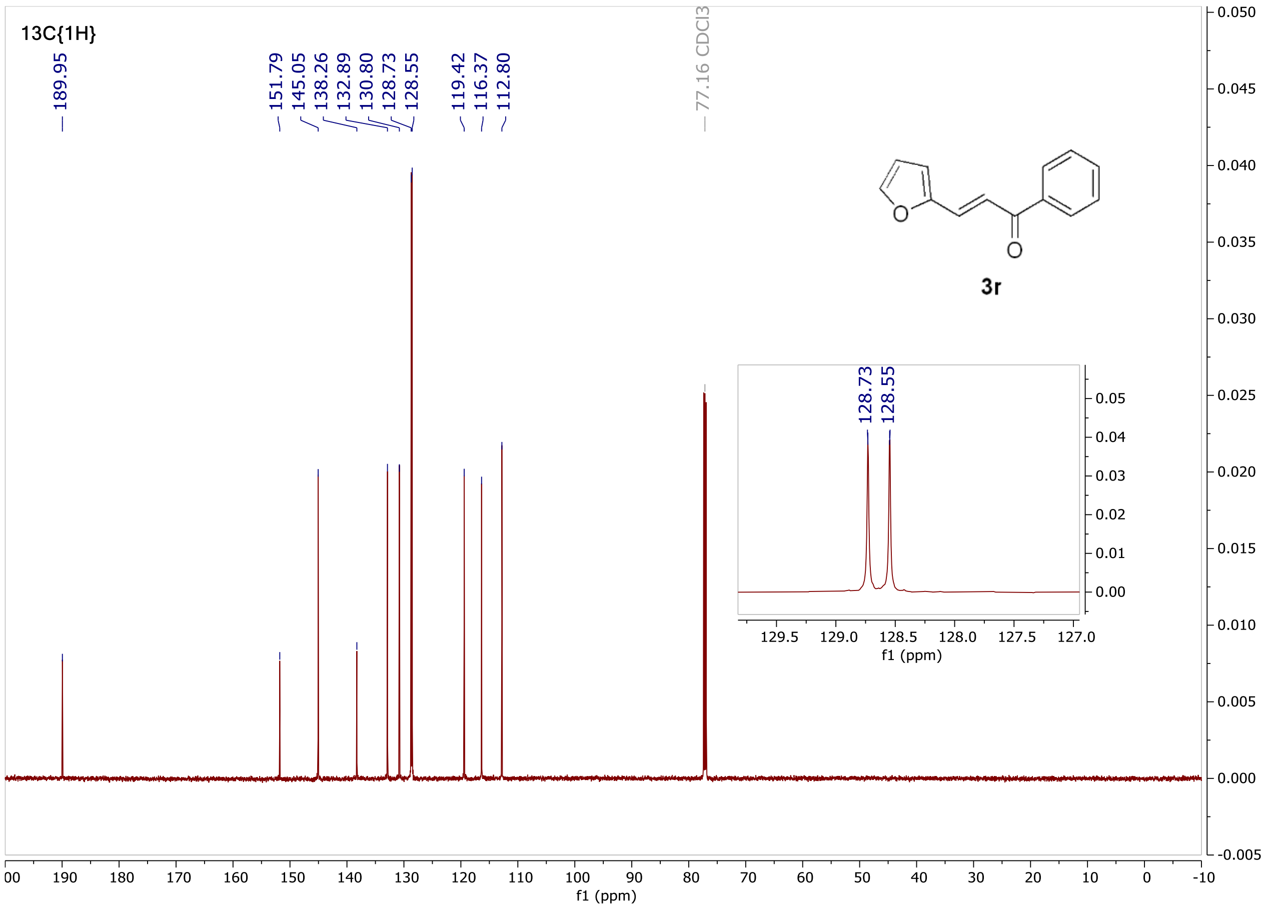
13C{1H}
189.95
151.79 145.05 138.26 132.89 130.80 128.73 128.55
119.42 116.37 112.80
77.16 CDCl3
3r
128.73 128.55
f1 (ppm)

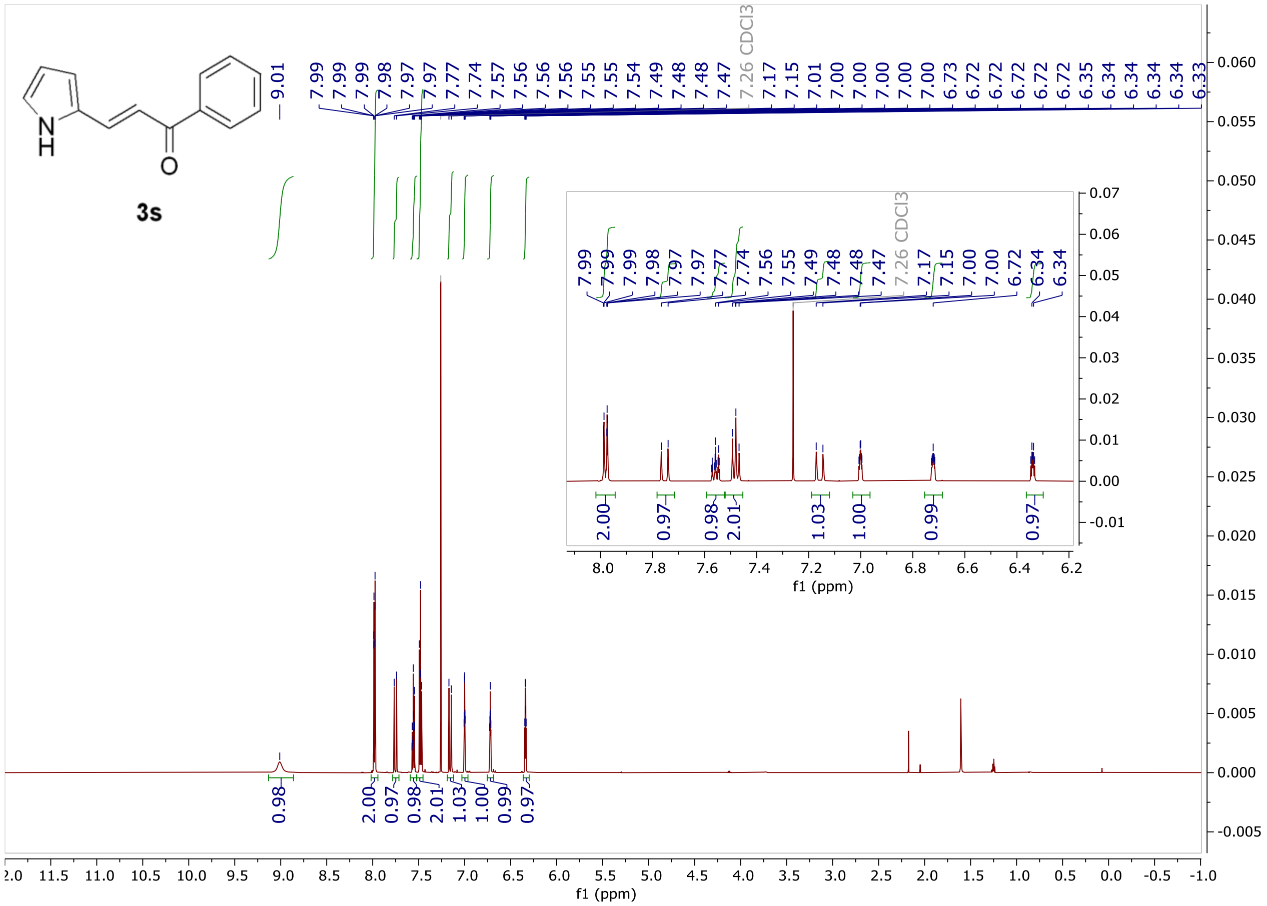

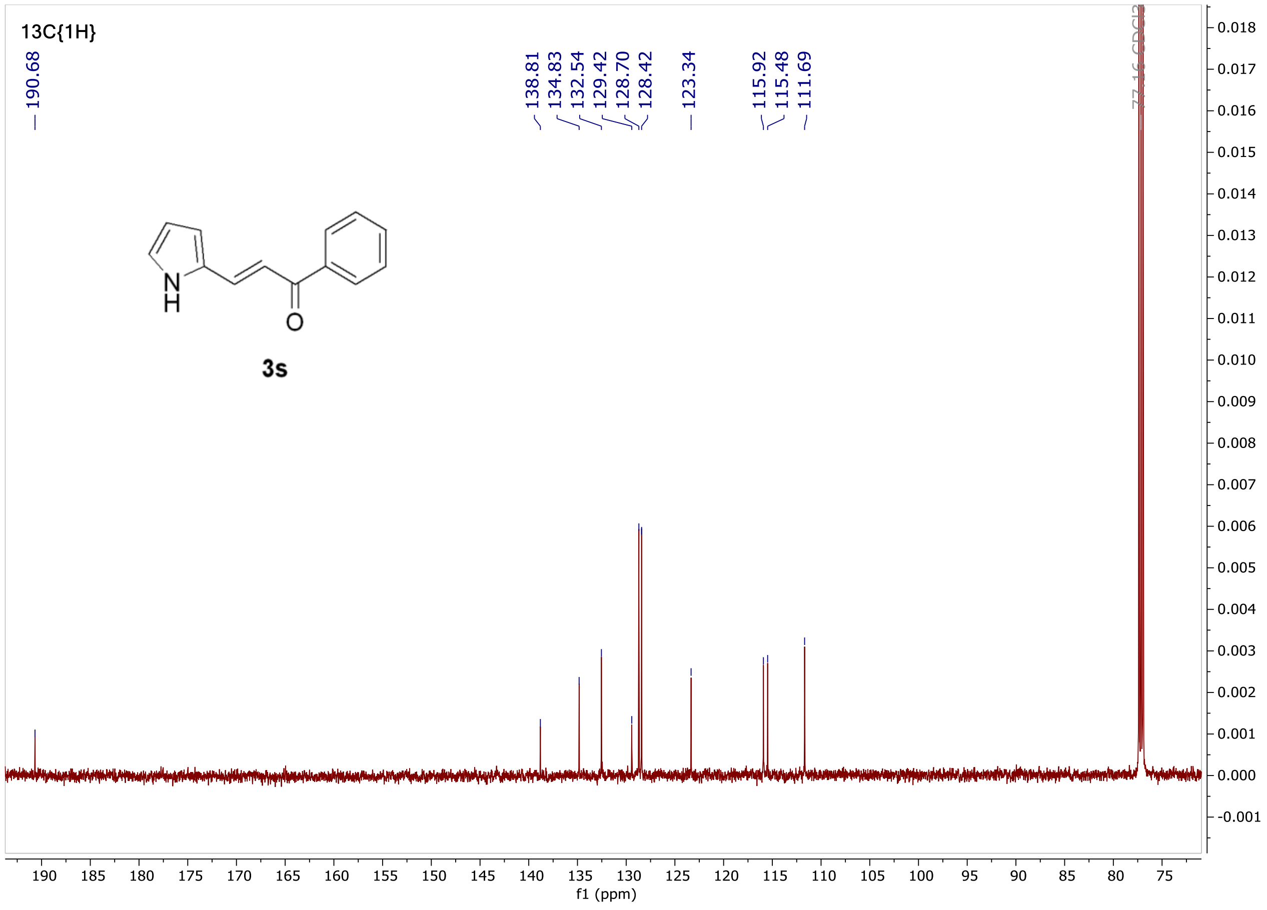

^{1}H and ^{13}C{1H} spectra of series 4

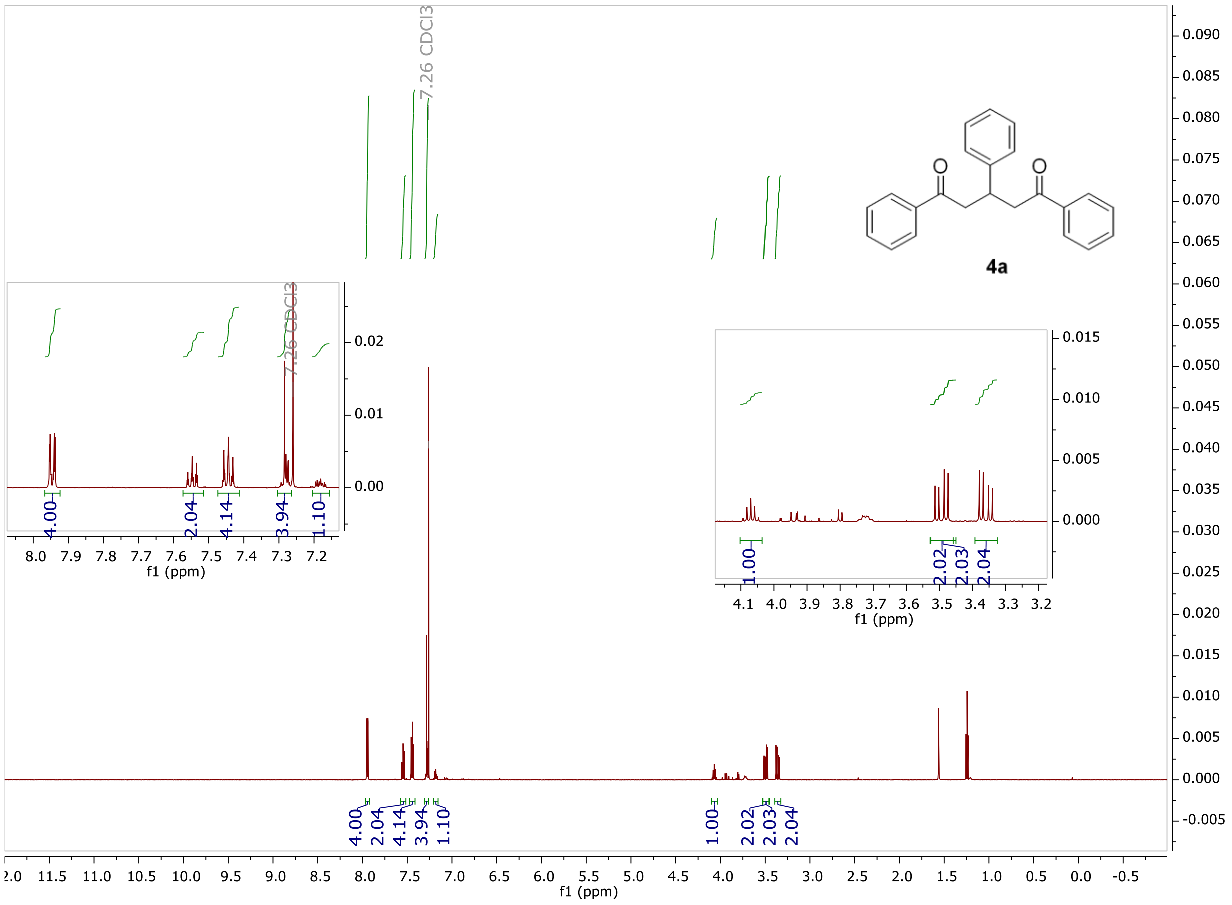

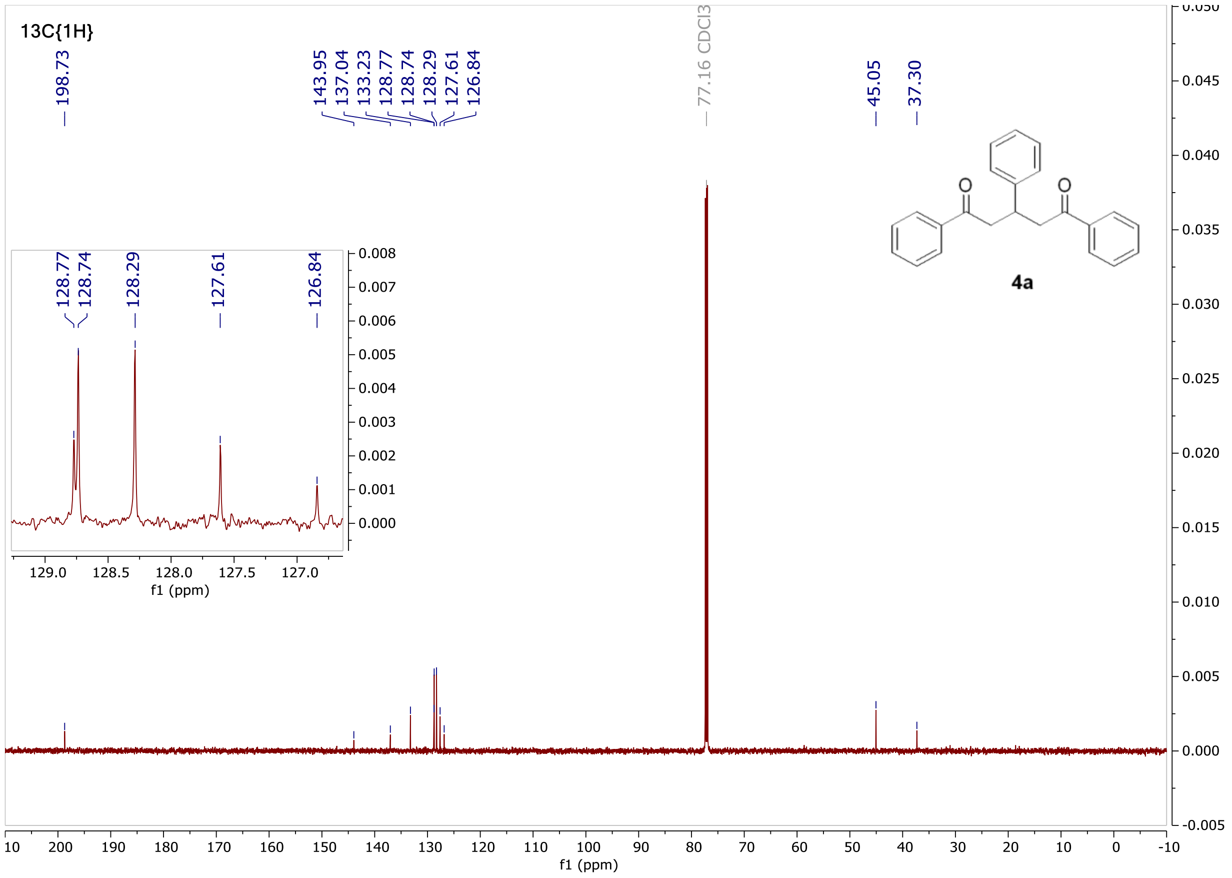

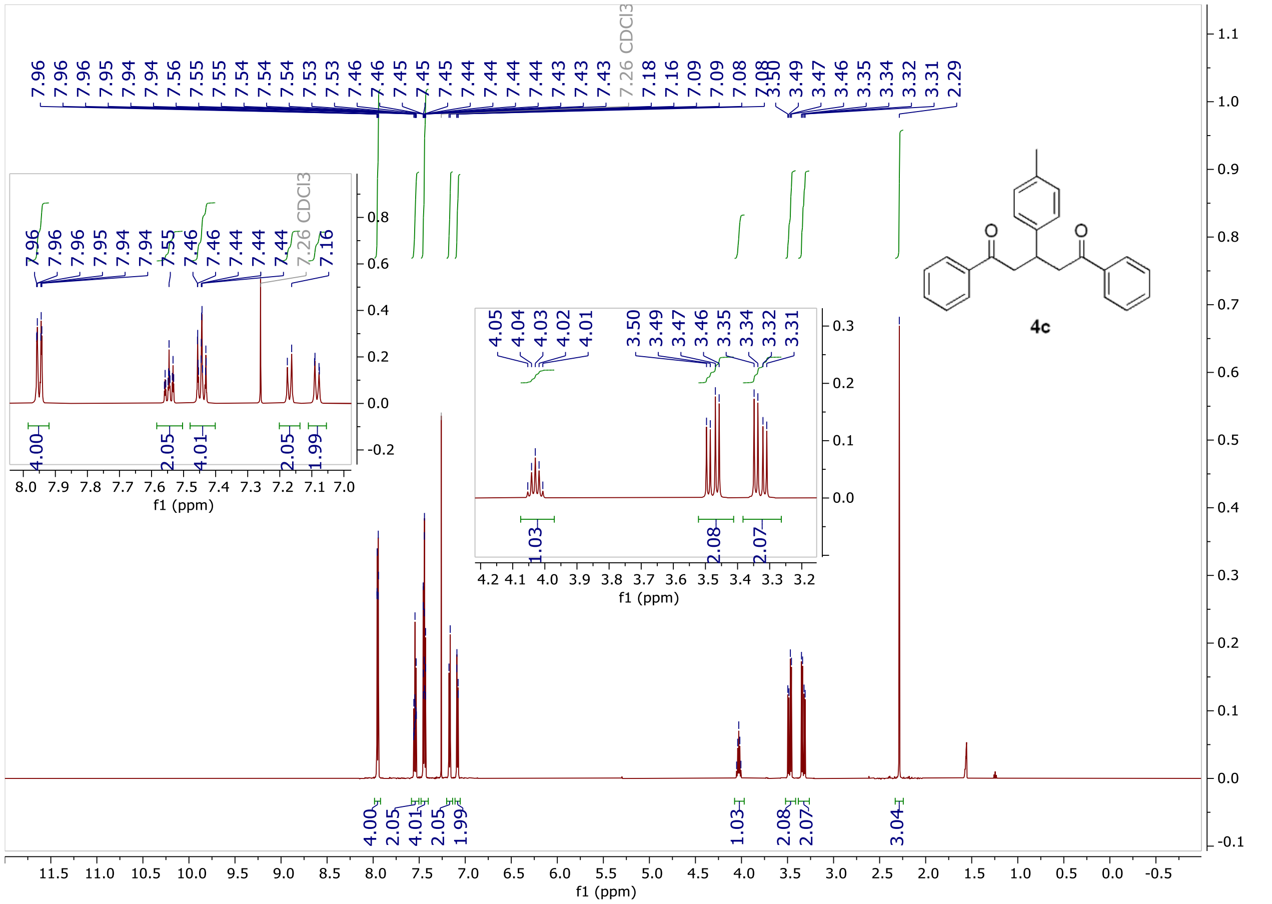

4c
f1 (ppm)

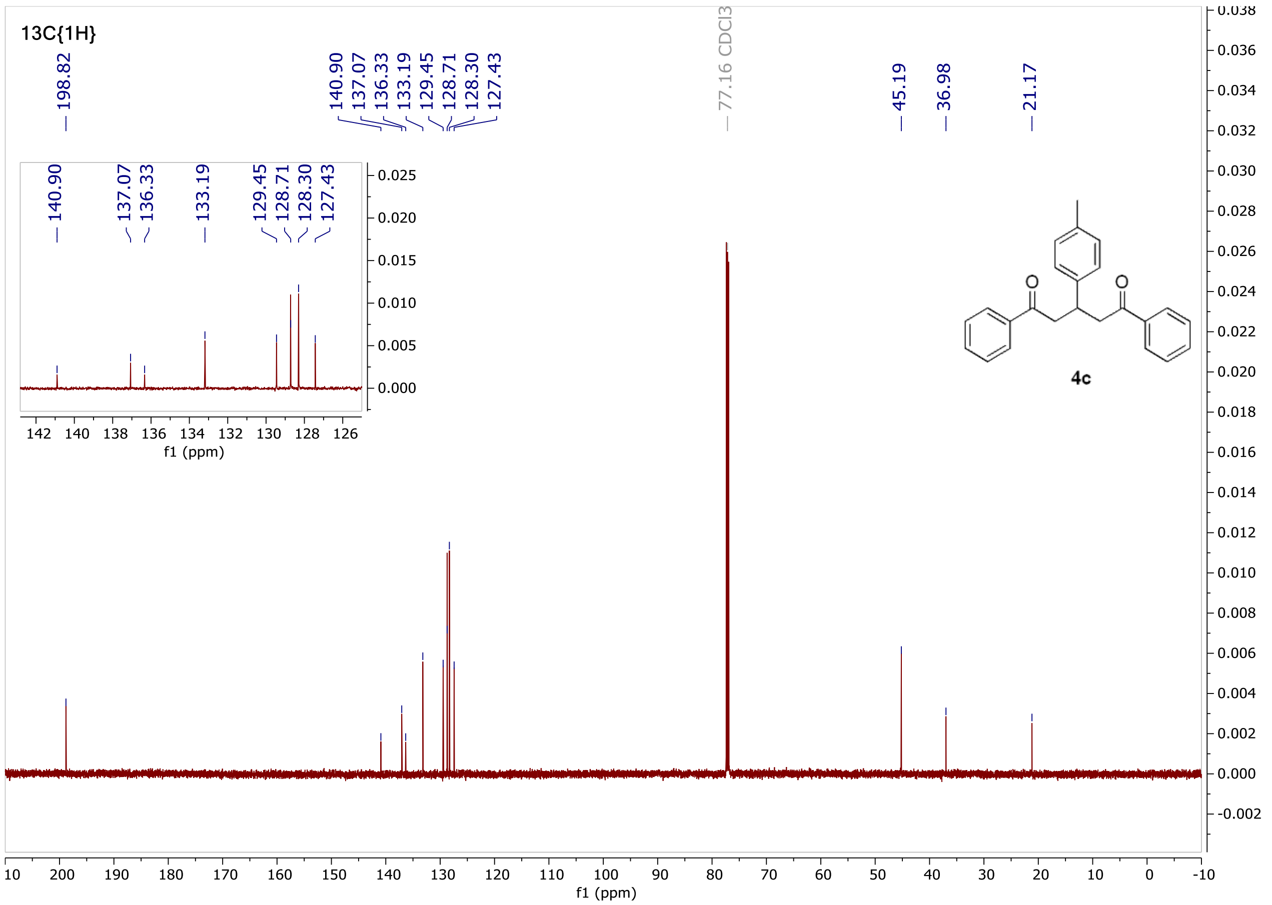

13C{1H}
4c
f1 (ppm)

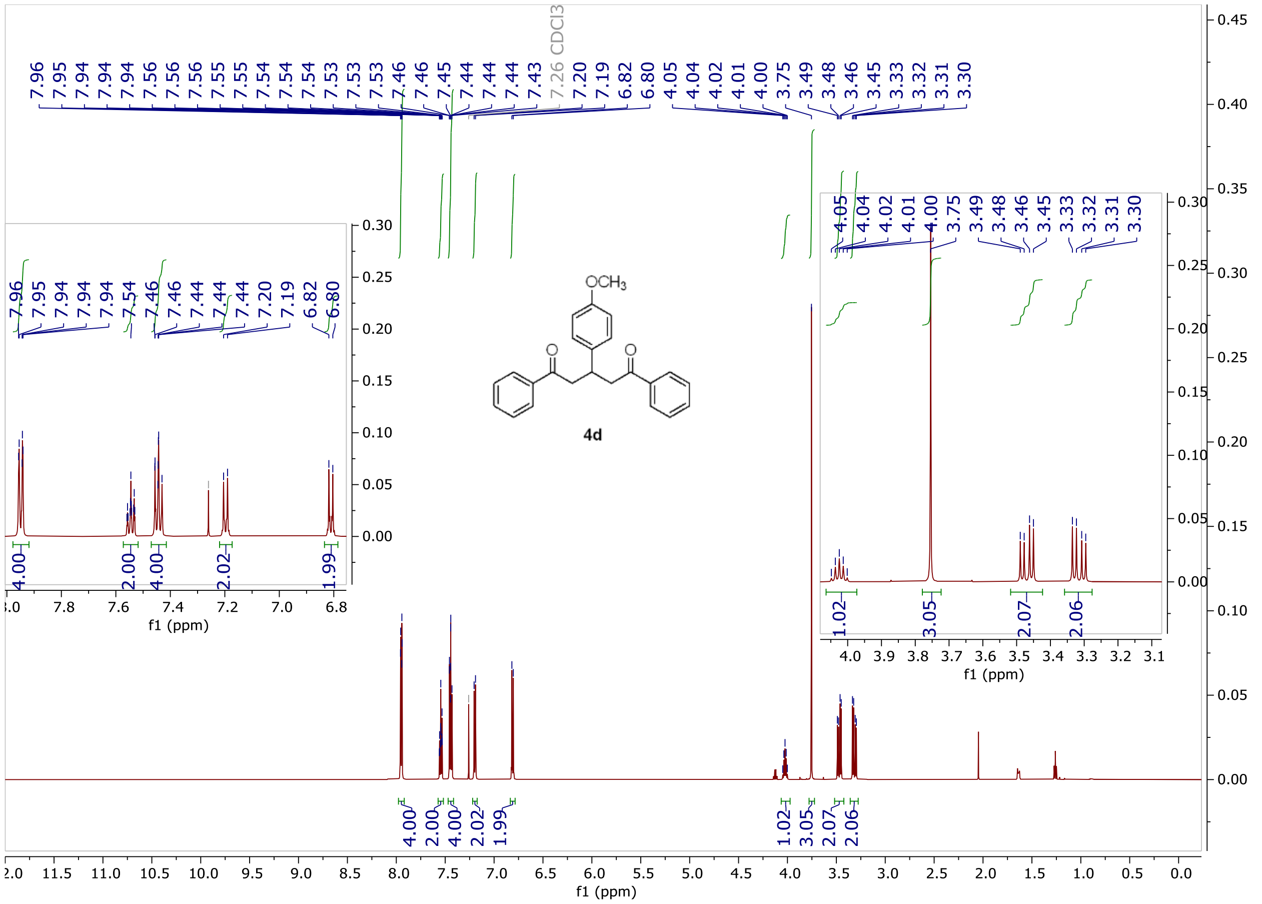
7.96
7.95
7.94
7.94
7.94
7.56
7.56
7.56
7.55
7.55
7.54
7.54
7.54
7.53
7.53
7.53
7.46
7.45
7.44
7.44
7.44
7.43
7.26 CDCl3
7.20
7.19
6.82
6.80
4.05
4.04
4.02
4.01
4.00
3.75
3.49
3.48
3.46
3.45
3.33
3.32
3.31
3.30
OCH3
4d
4.00
2.00
4.00
2.02
1.99
1.02
3.05
2.07
2.06
f1 (ppm)

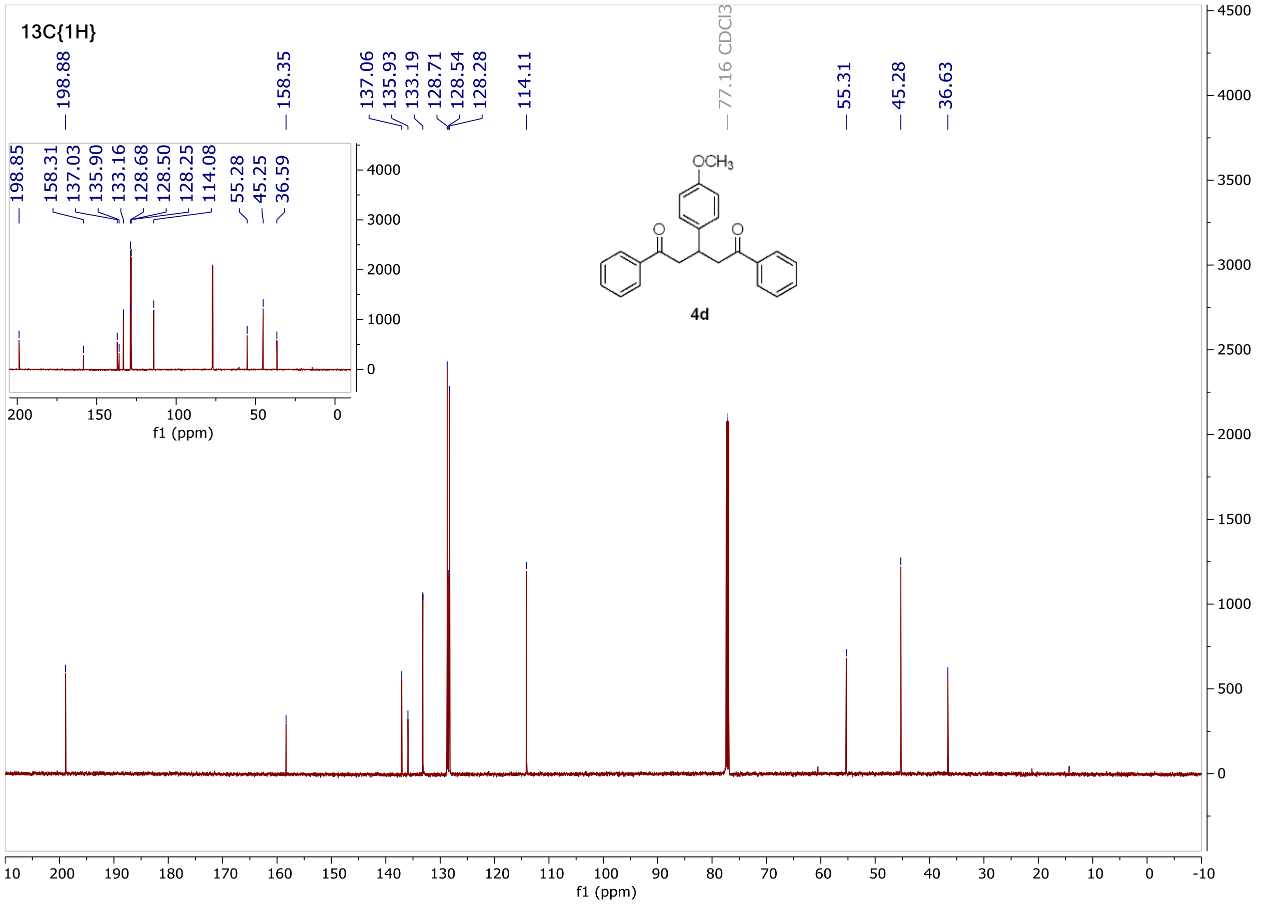
13C{1H}
198.88
158.35
137.06
135.93
133.19
128.71
128.54
128.28
114.11
77.16 CDCl3
55.31
45.28
36.63
198.85
158.31
137.03
135.90
133.16
128.68
128.50
128.25
114.08
55.28
45.25
36.59
OCH3
4d
f1 (ppm)

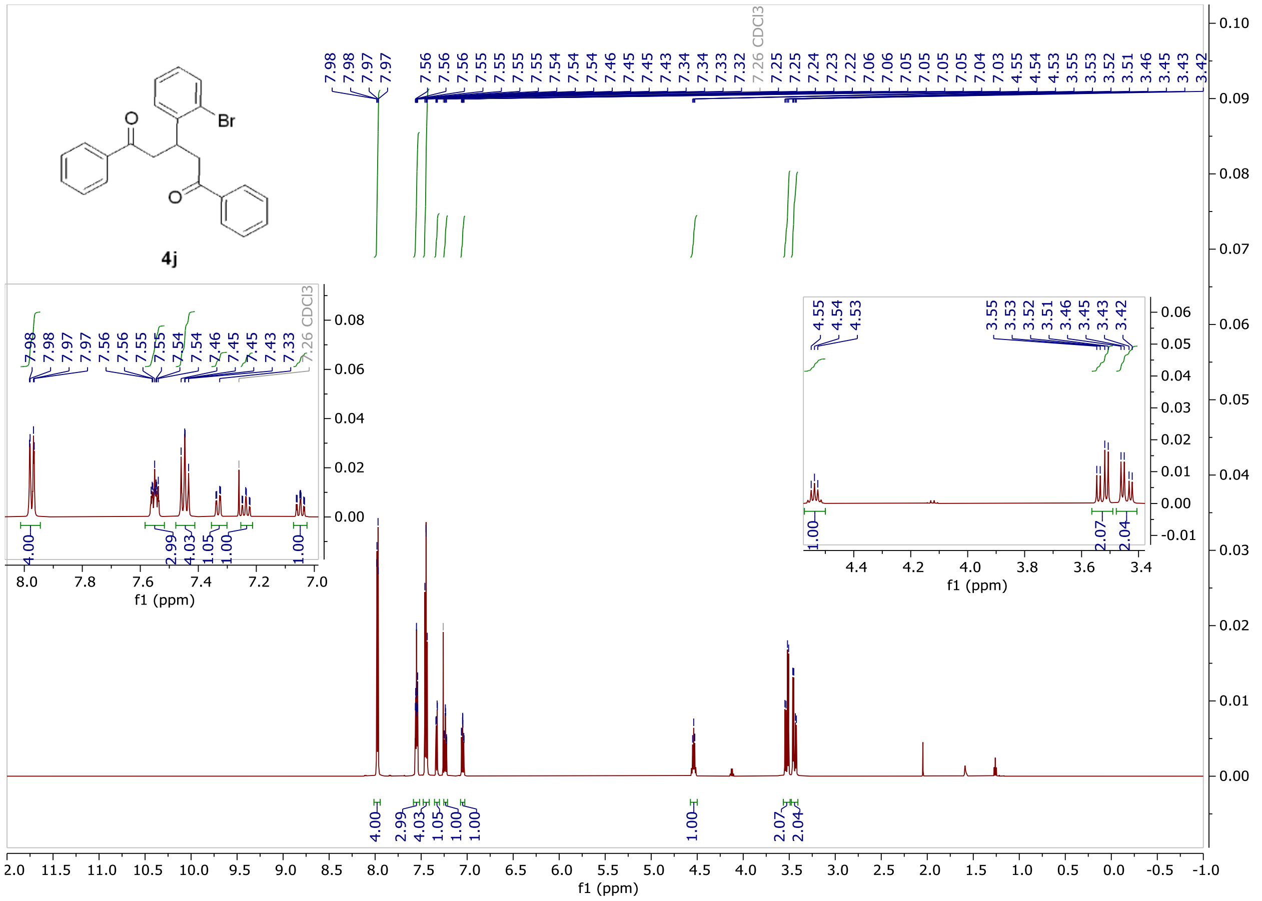

4j
7.98, 7.98, 7.97, 7.97, 7.56, 7.56, 7.56, 7.55, 7.55, 7.55, 7.54, 7.54, 7.54, 7.46, 7.45, 7.45, 7.43, 7.34, 7.34, 7.33, 7.32, 7.26 CDCl3, 7.25, 7.25, 7.24, 7.23, 7.22, 7.06, 7.06, 7.05, 7.05, 7.05, 7.05, 7.04, 7.03, 4.55, 4.54, 4.53, 3.55, 3.53, 3.52, 3.51, 3.46, 3.45, 3.43, 3.42
4.00, 2.99, 4.03, 1.05, 1.00, 1.00, 1.00, 2.07, 2.04
f1 (ppm)

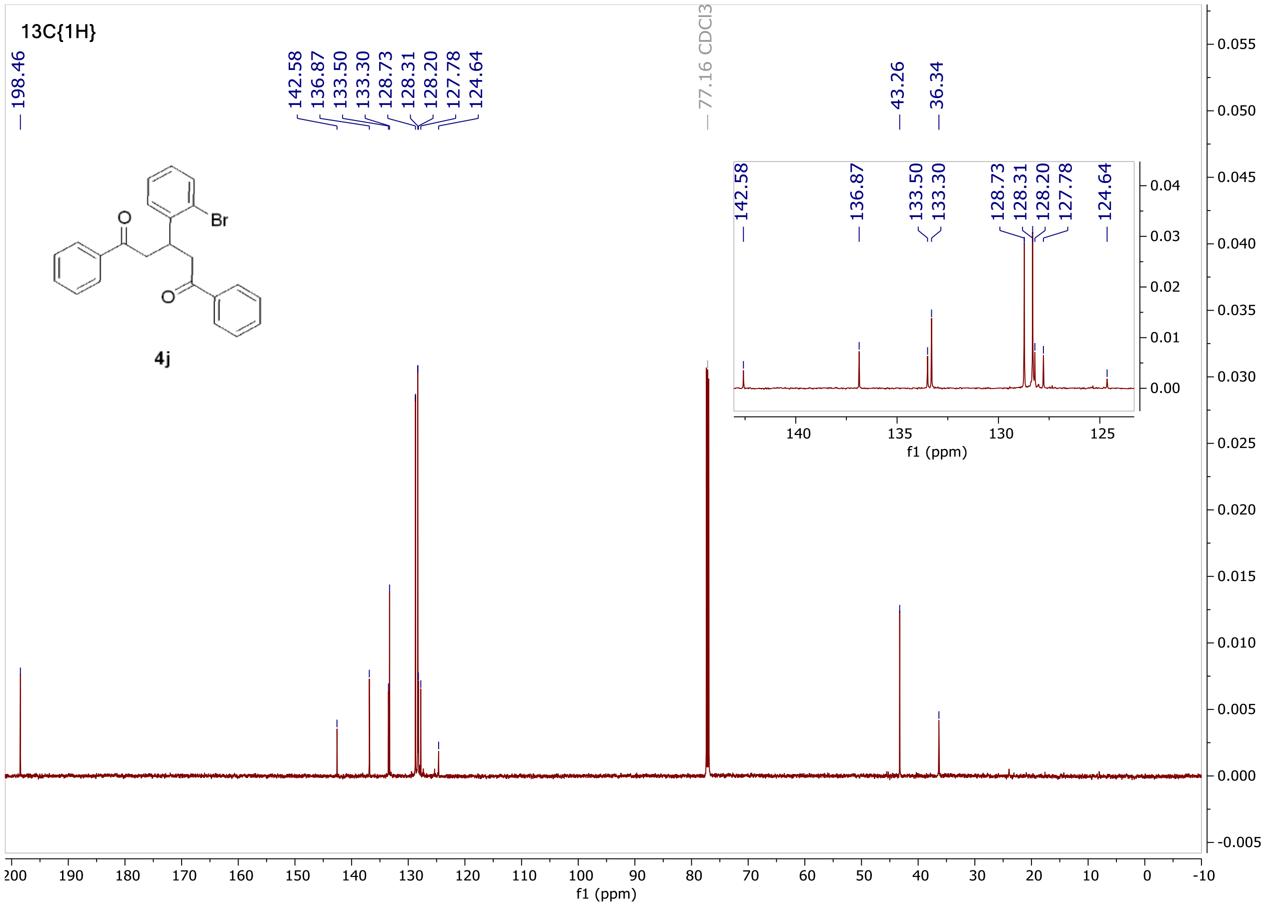

13C{1H}
4j
198.46
142.58, 136.87, 133.50, 133.30, 128.73, 128.31, 128.20, 127.78, 124.64
77.16 CDCl3
43.26, 36.34
f1 (ppm)

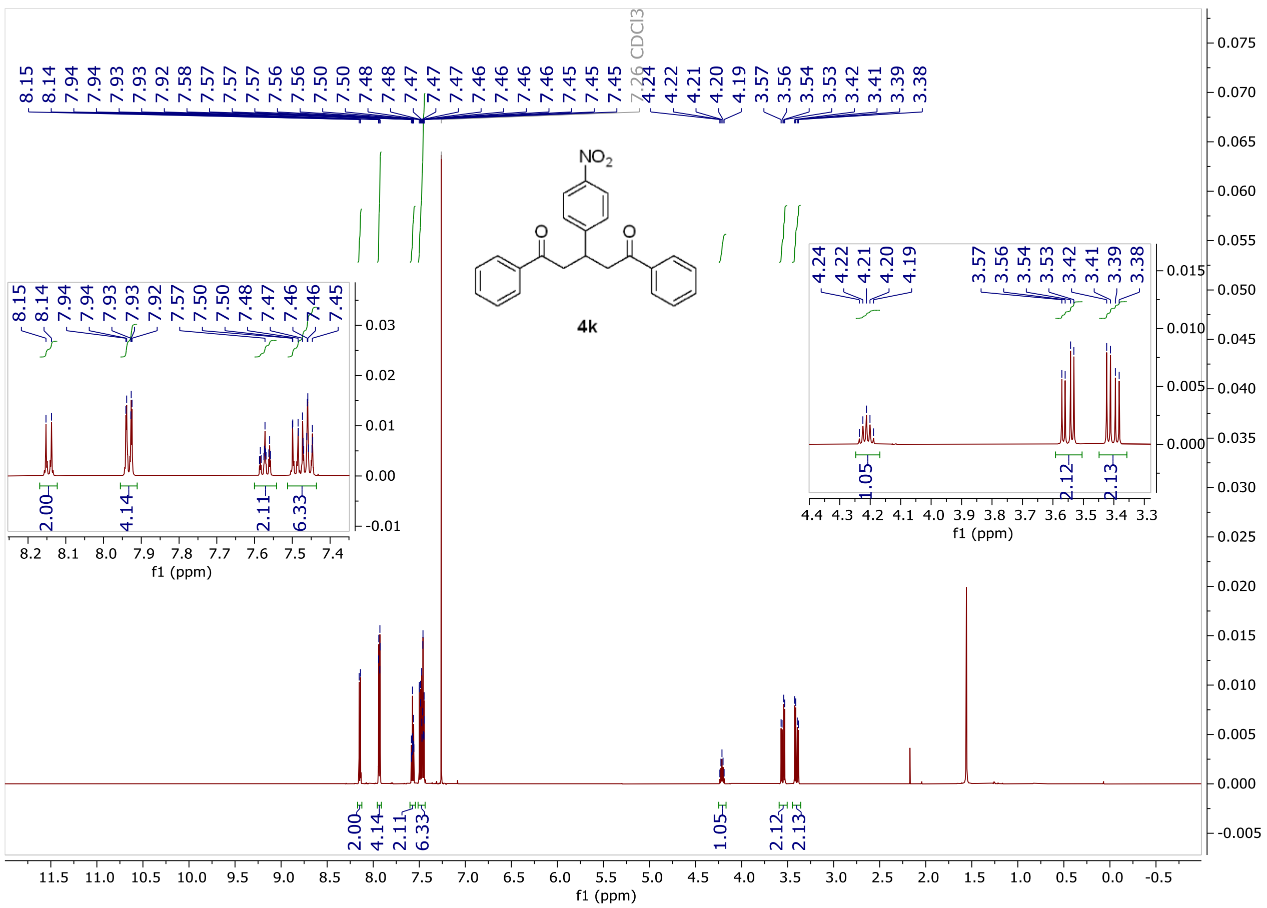
8.15 8.14 7.94 7.94 7.93 7.93 7.92 7.58 7.57 7.57 7.57 7.56 7.56 7.50 7.50 7.48 7.48 7.47 7.47 7.47 7.46 7.46 7.46 7.45 7.45 7.45
7.26 CDCl3
4.24 4.22 4.21 4.20 4.19 3.57 3.56 3.54 3.53 3.42 3.41 3.39 3.38
NO2
4k
2.00 4.14 2.11 6.33 1.05 2.12 2.13
f1 (ppm)

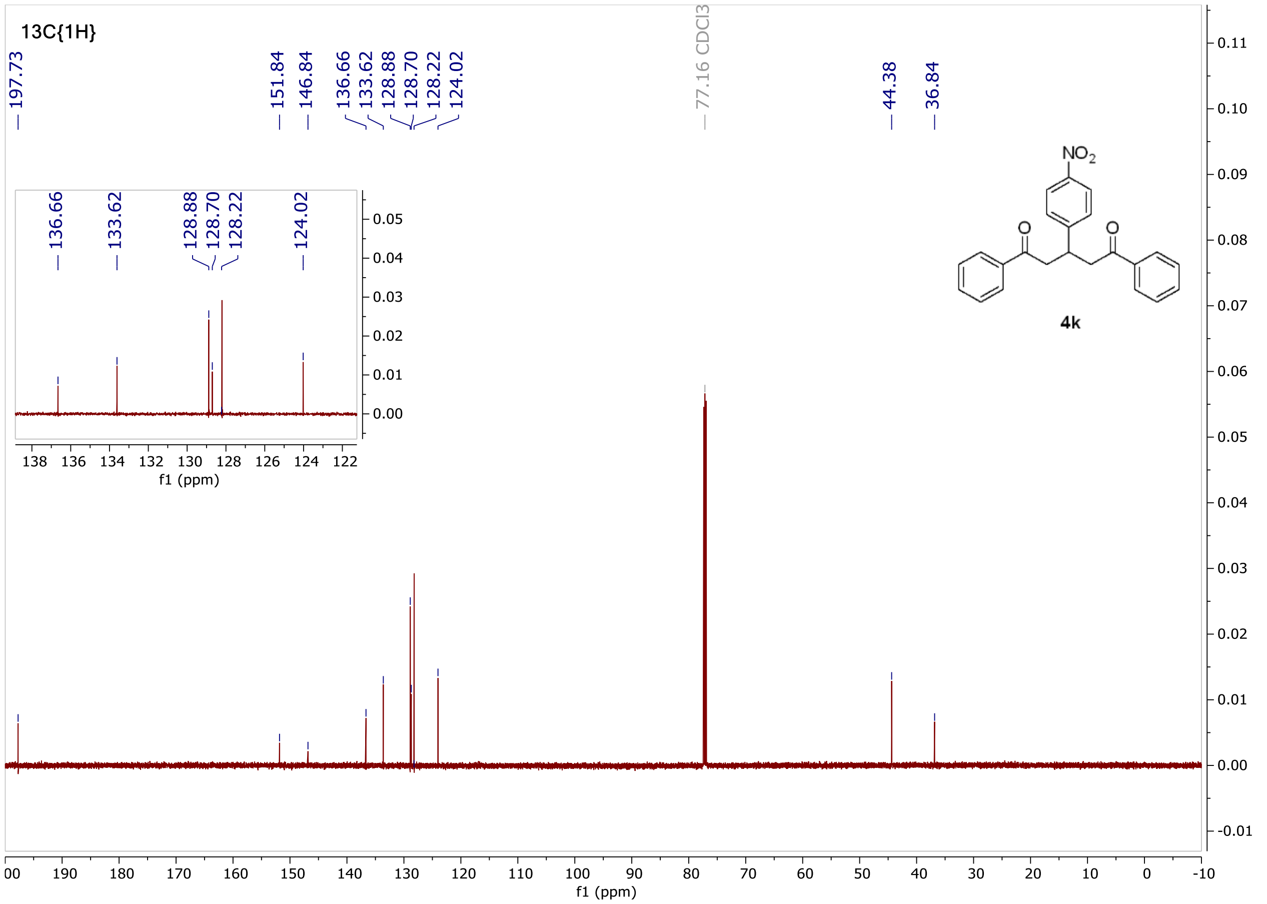
13C{1H}
197.73
151.84 146.84 136.66 133.62 128.88 128.70 128.22 124.02
77.16 CDCl3
44.38 36.84
NO2
4k
f1 (ppm)

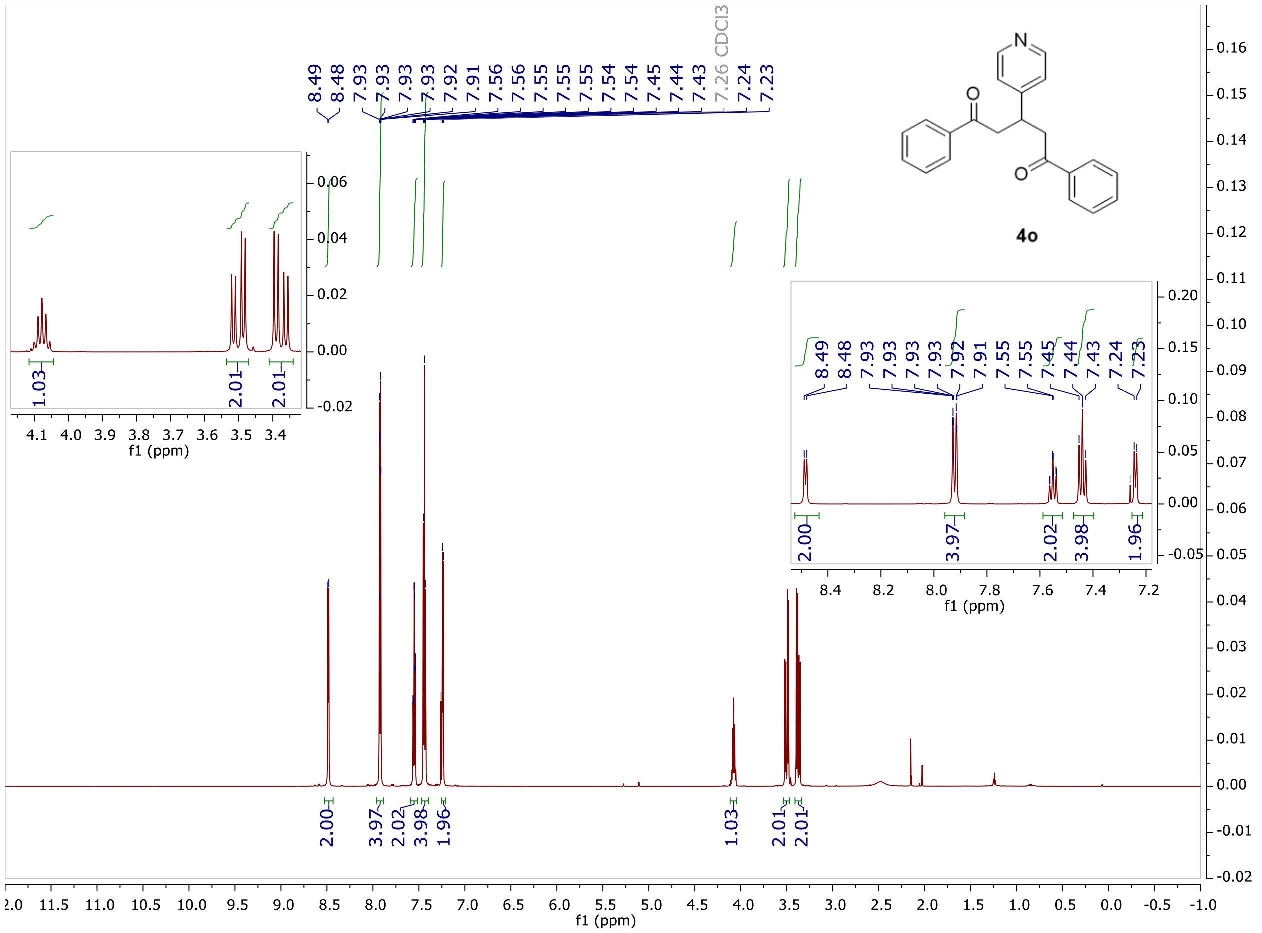
8.49
8.48
7.93
7.93
7.93
7.93
7.92
7.91
7.56
7.55
7.55
7.55
7.54
7.54
7.45
7.44
7.43
7.26 CDCl3
7.24
7.23
4o
2.00
3.97
2.02
3.98
1.96
1.03
2.01
2.01
f1 (ppm)

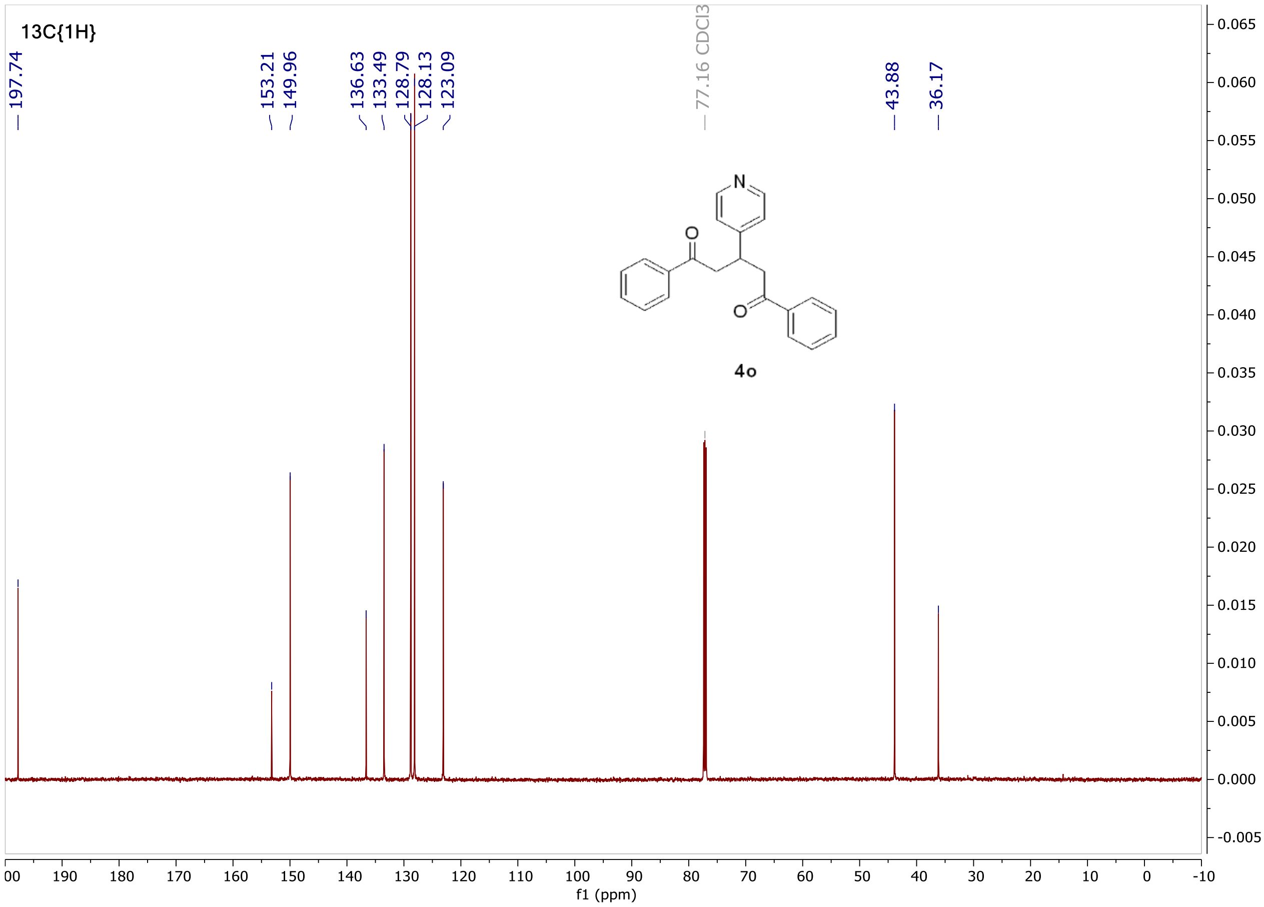
13C{1H}
197.74
153.21
149.96
136.63
133.49
128.79
128.13
123.09
77.16 CDCl3
43.88
36.17
4o
f1 (ppm)

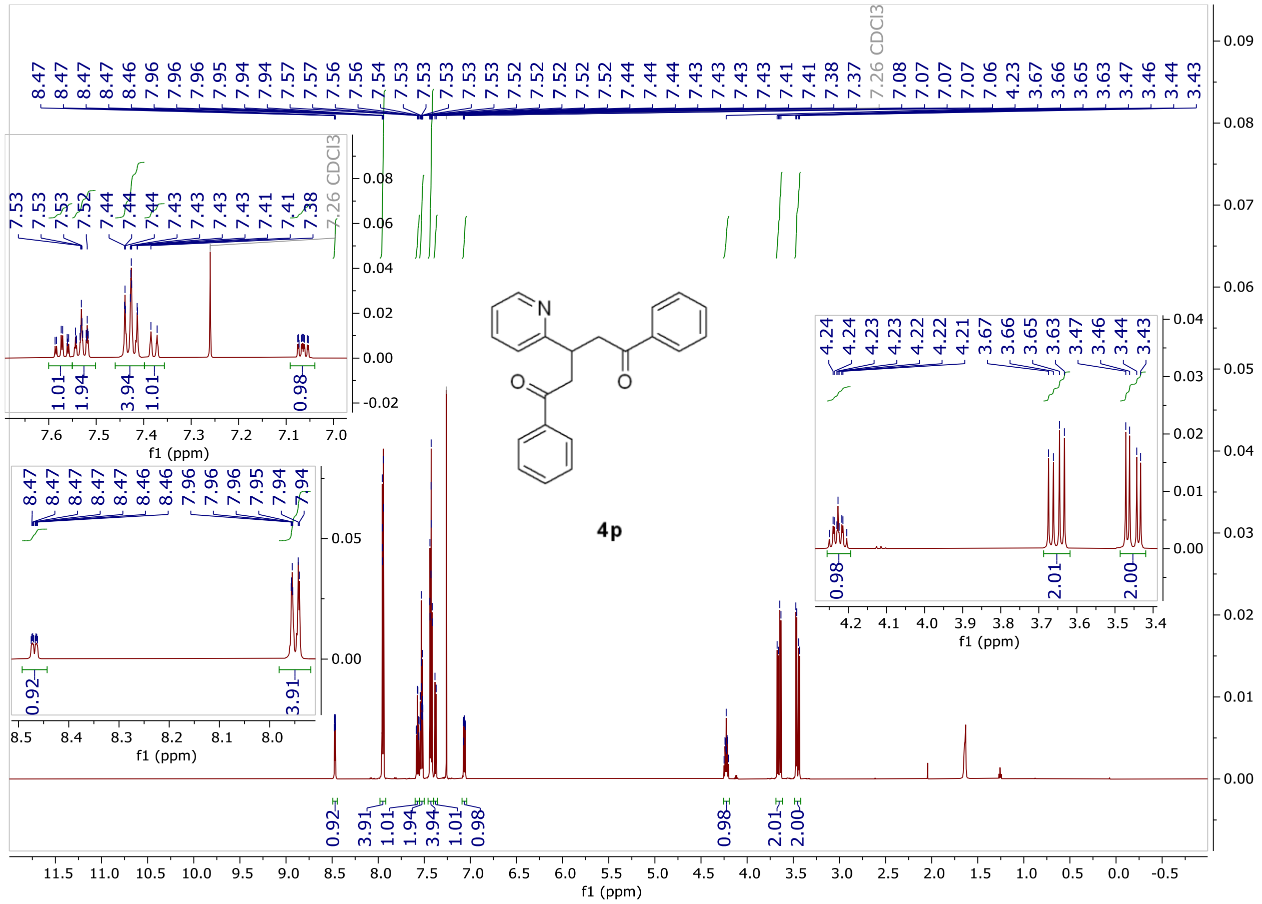
4p
f1 (ppm)

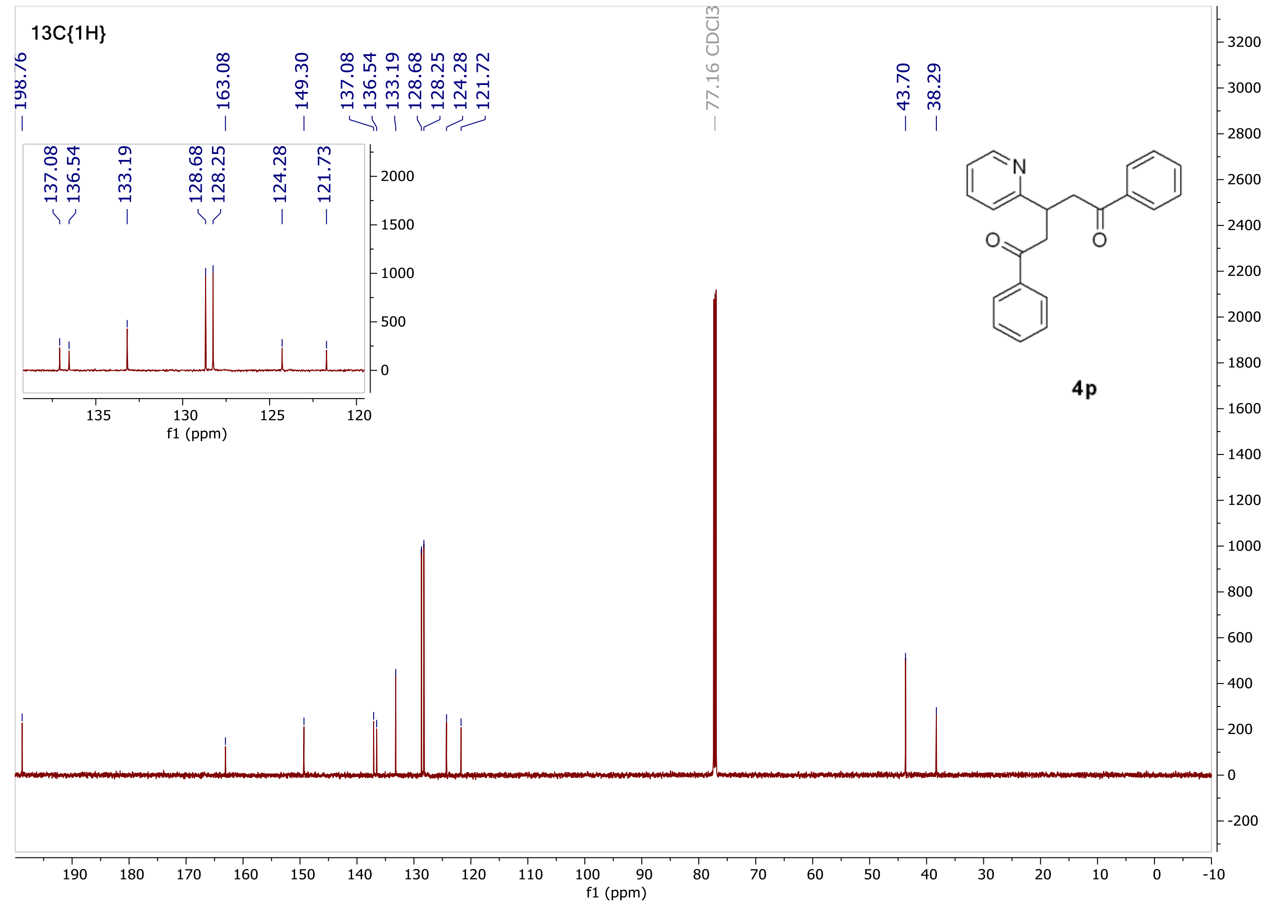
13C{1H}
198.76
163.08
149.30
137.08
136.54
133.19
128.68
128.25
124.28
121.72
77.16 CDCl3
43.70
38.29
4p
f1 (ppm)

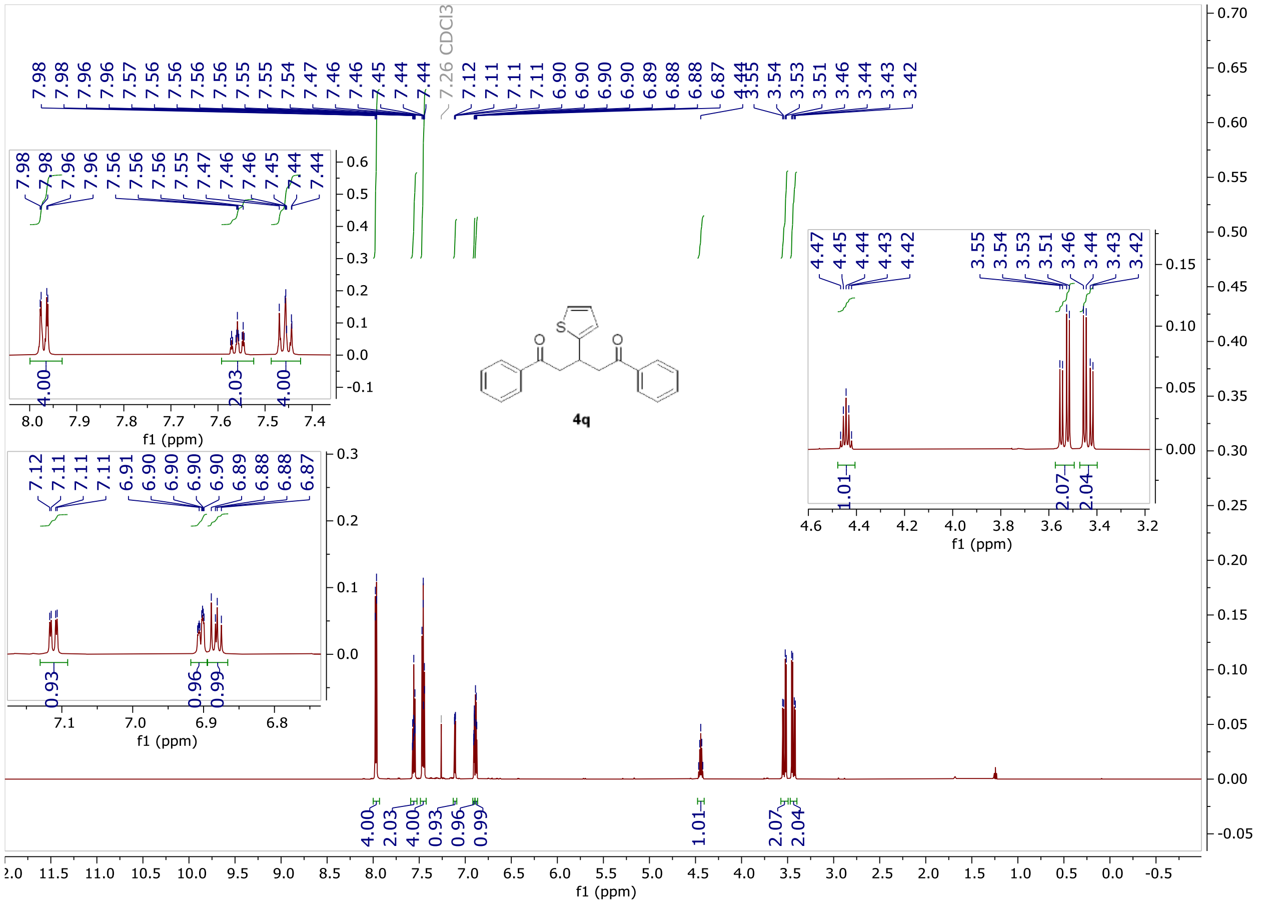

7.98 7.98 7.96 7.96 7.57 7.56 7.56 7.56 7.56 7.55 7.55 7.54 7.47 7.46 7.46 7.45 7.44 7.44
7.26 CDCl3
7.12 7.11 7.11 7.11 6.90 6.90 6.90 6.90 6.89 6.88 6.88 6.87
4.44 3.55 3.54 3.53 3.51 3.46 3.44 3.43 3.42
4.00 2.03 4.00 0.93 0.96 0.99 1.01 2.07 2.04
4.47 4.45 4.44 4.43 4.42
6.91
4q
f1 (ppm)

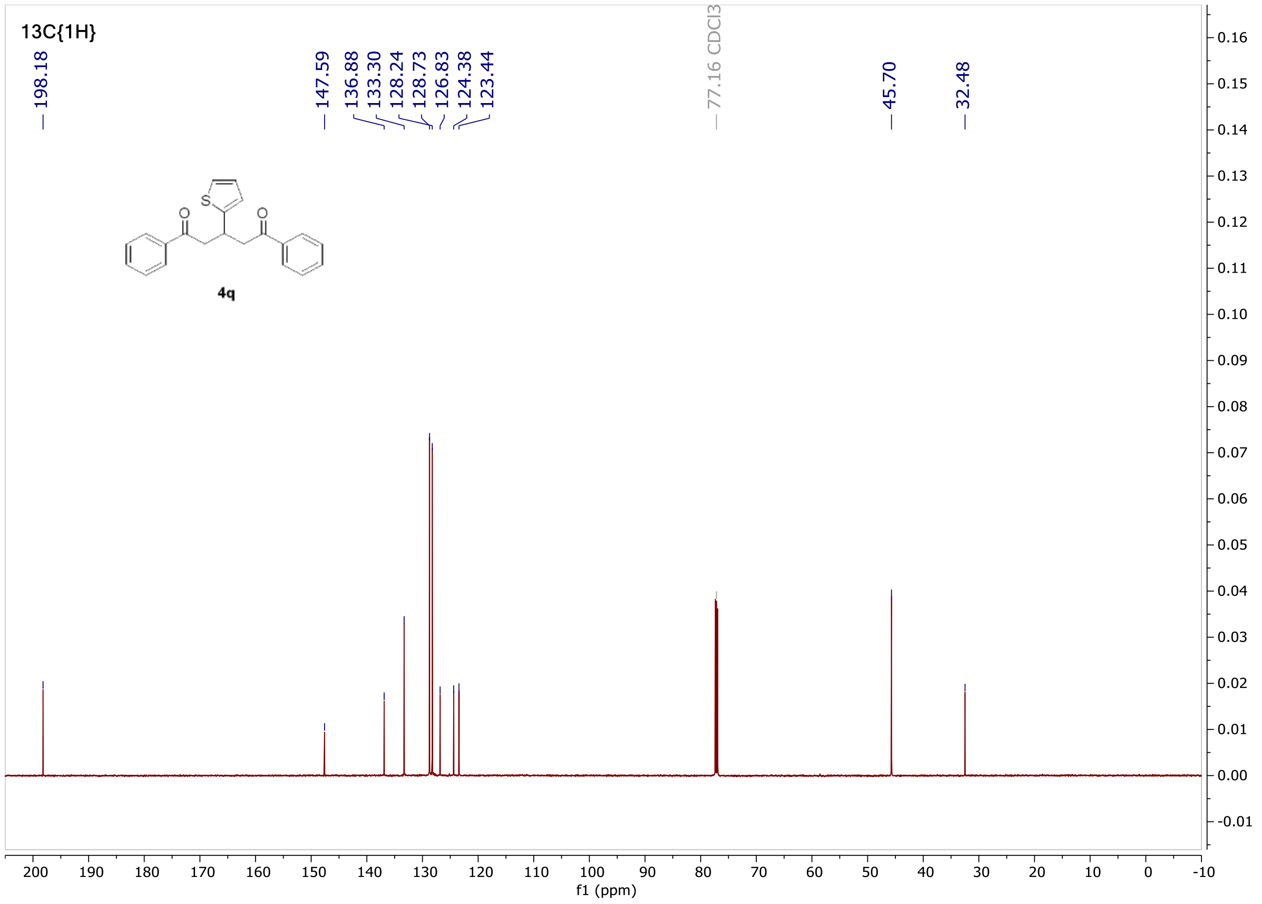

13C{1H}
198.18
147.59 136.88 133.30 128.24 128.73 126.83 124.38 123.44
77.16 CDCl3
45.70
32.48
4q
f1 (ppm)

^{1}H and $^{13}C\{1H\}$ spectra of series 6

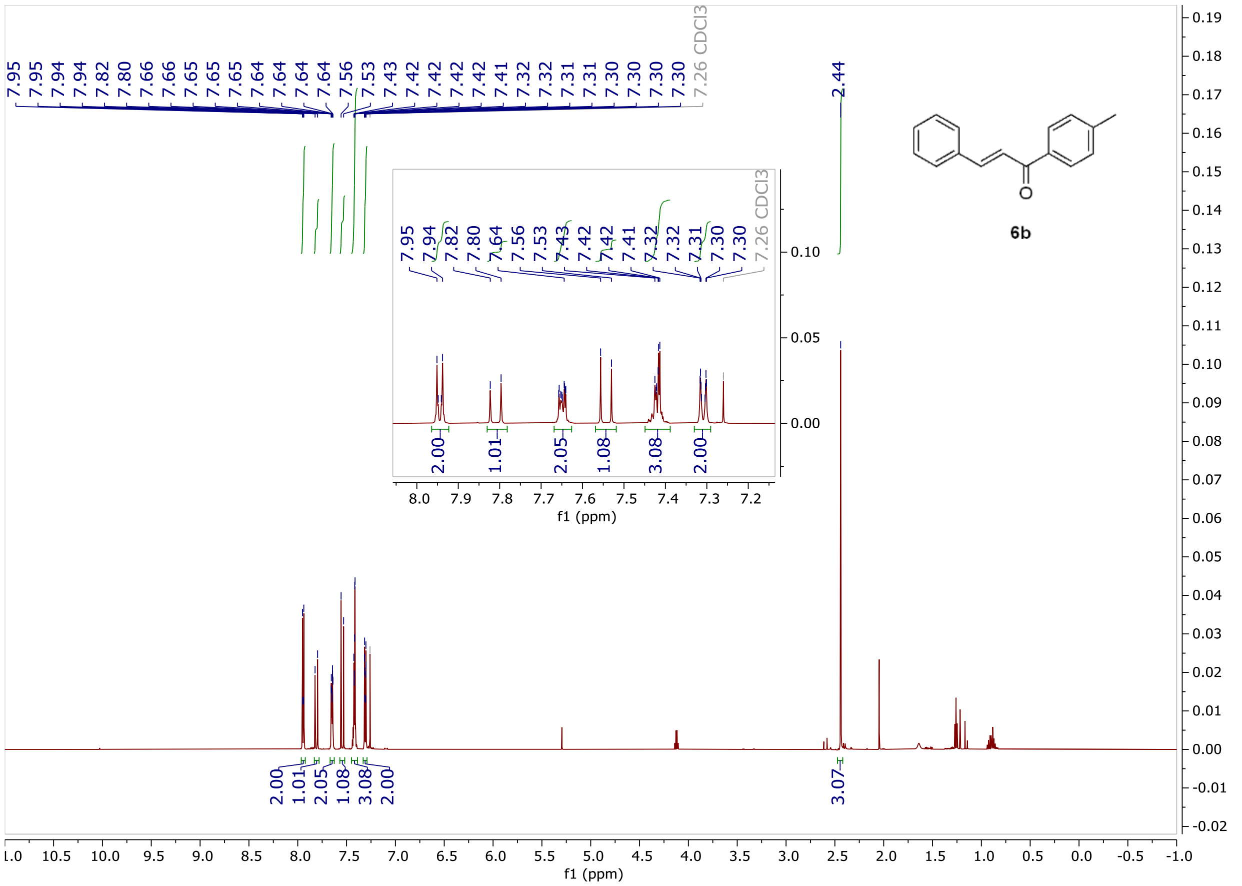

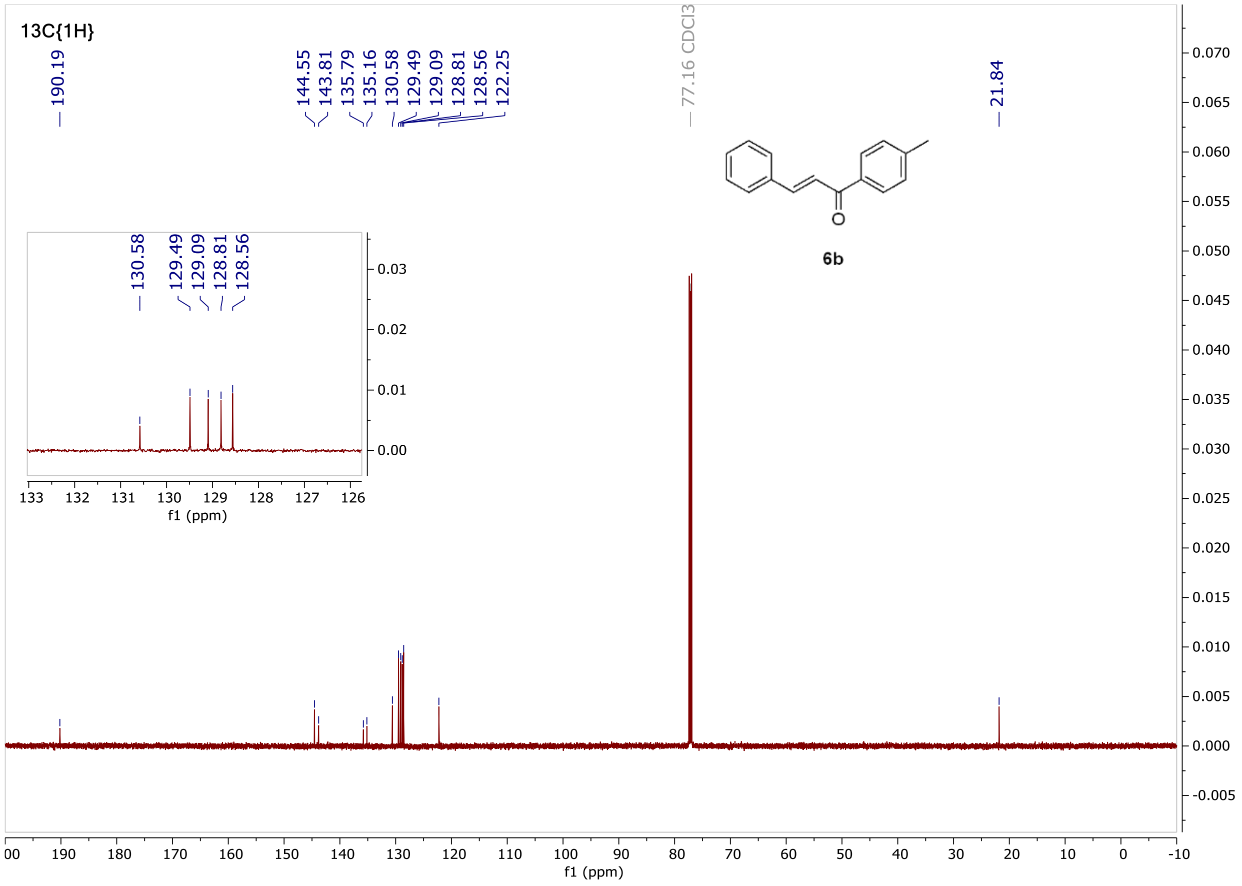

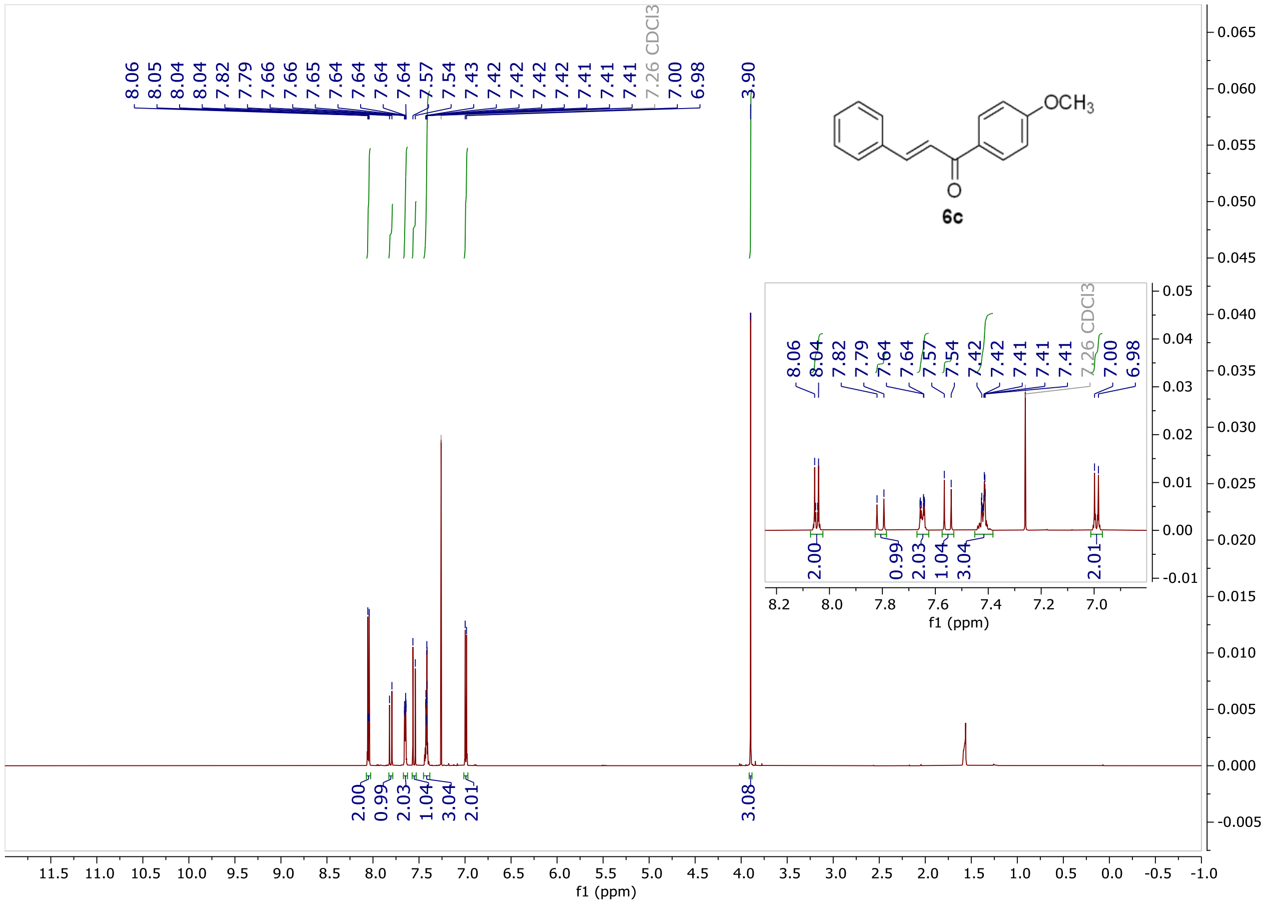
8.06
8.05
8.04
8.04
7.82
7.79
7.66
7.65
7.64
7.64
7.64
7.64
7.57
7.54
7.43
7.42
7.42
7.42
7.41
7.41
7.41
7.26 CDCl3
7.00
6.98
3.90
OCH3
6c
2.00
0.99
2.03
1.04
3.04
2.01
3.08
f1 (ppm)

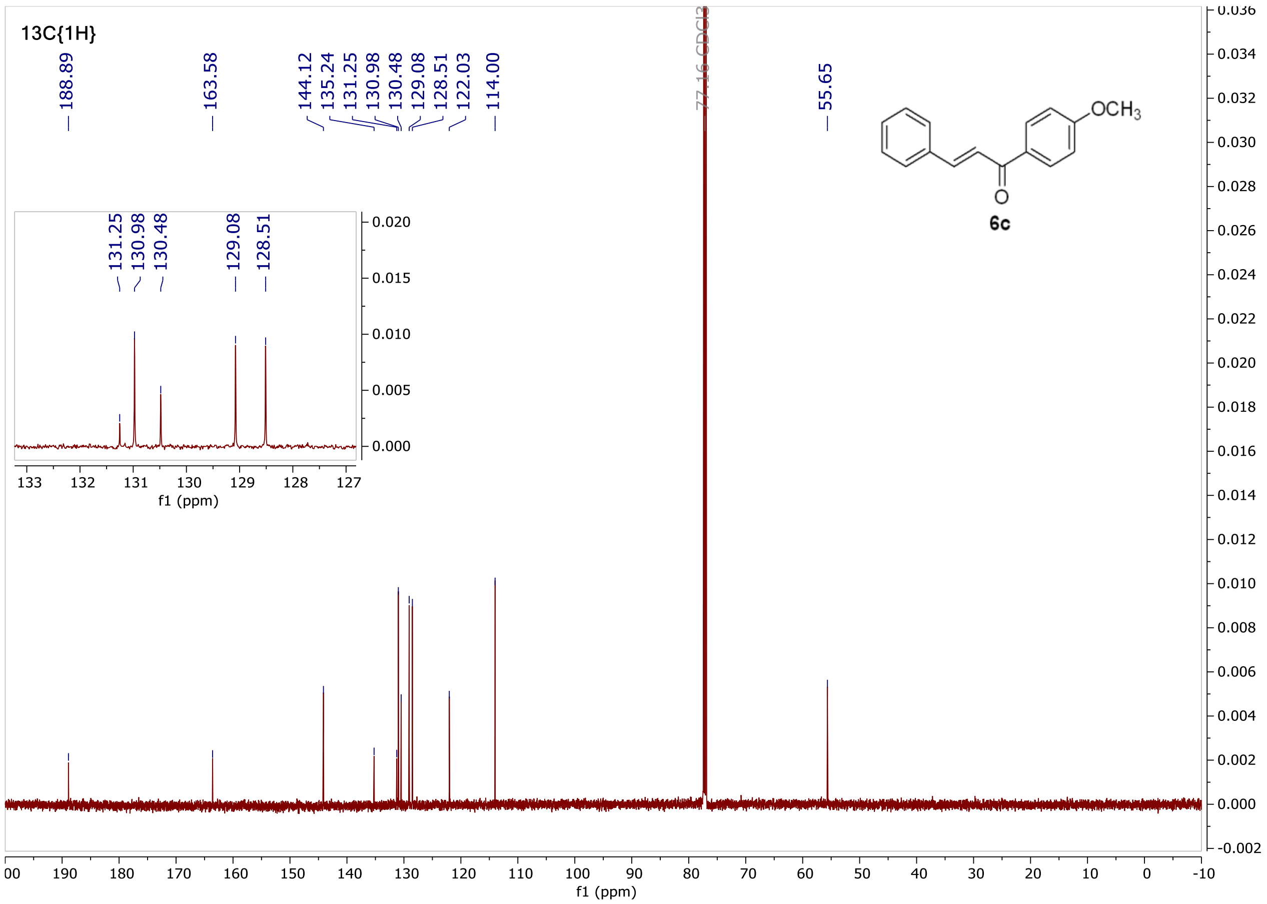
13C{1H}
188.89
163.58
144.12
135.24
131.25
130.98
130.48
129.08
128.51
122.03
114.00
77.16 CDCl3
55.65
OCH3
6c
f1 (ppm)

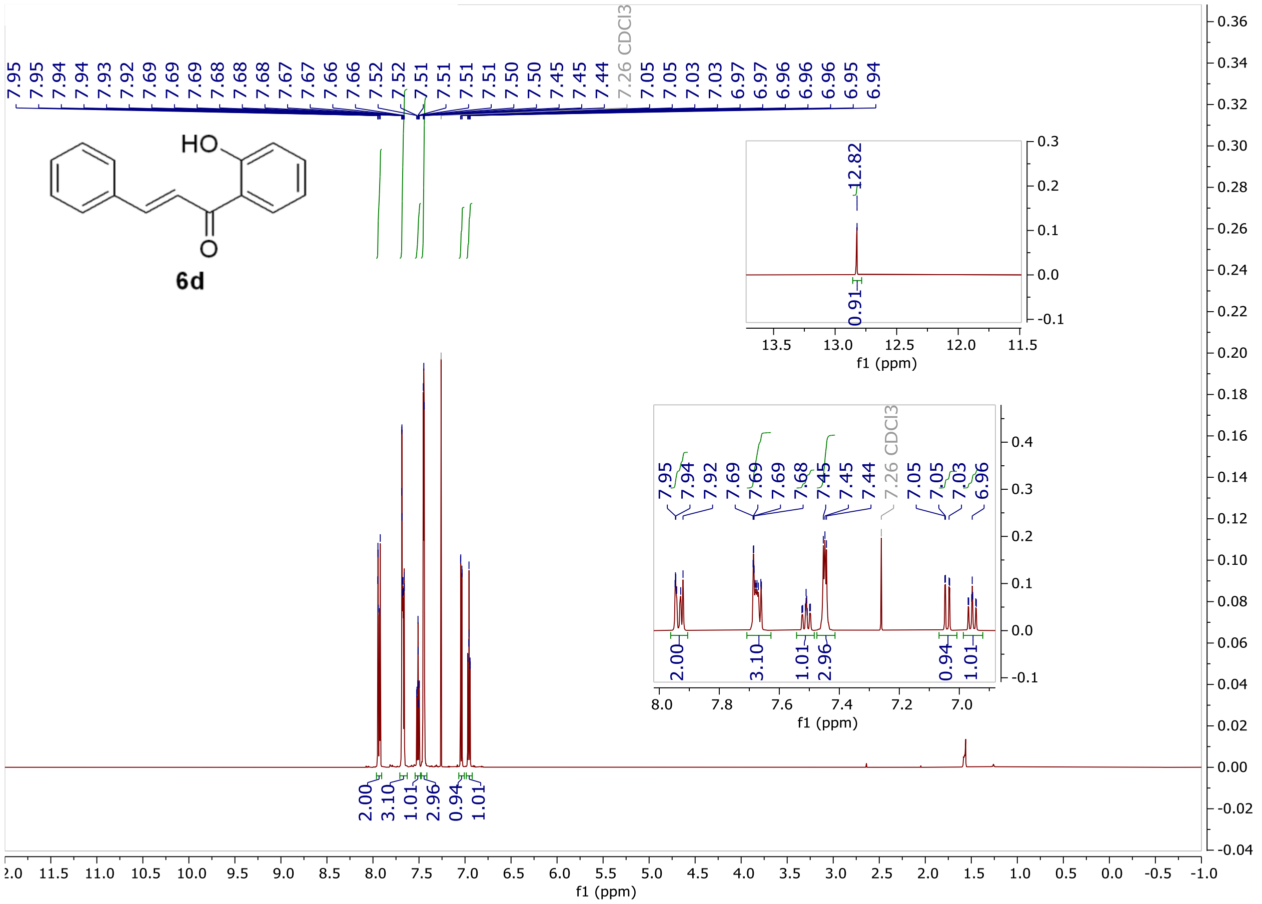
7.95
7.95
7.94
7.94
7.93
7.92
7.69
7.69
7.69
7.68
7.68
7.68
7.67
7.67
7.66
7.66
7.52
7.52
7.51
7.51
7.51
7.51
7.50
7.45
7.45
7.44
7.26 CDCl3
7.05
7.05
7.03
7.03
6.97
6.97
6.96
6.96
6.95
6.94
HO
O
6d
12.82
0.91
2.00
3.10
1.01
2.96
0.94
1.01
f1 (ppm)

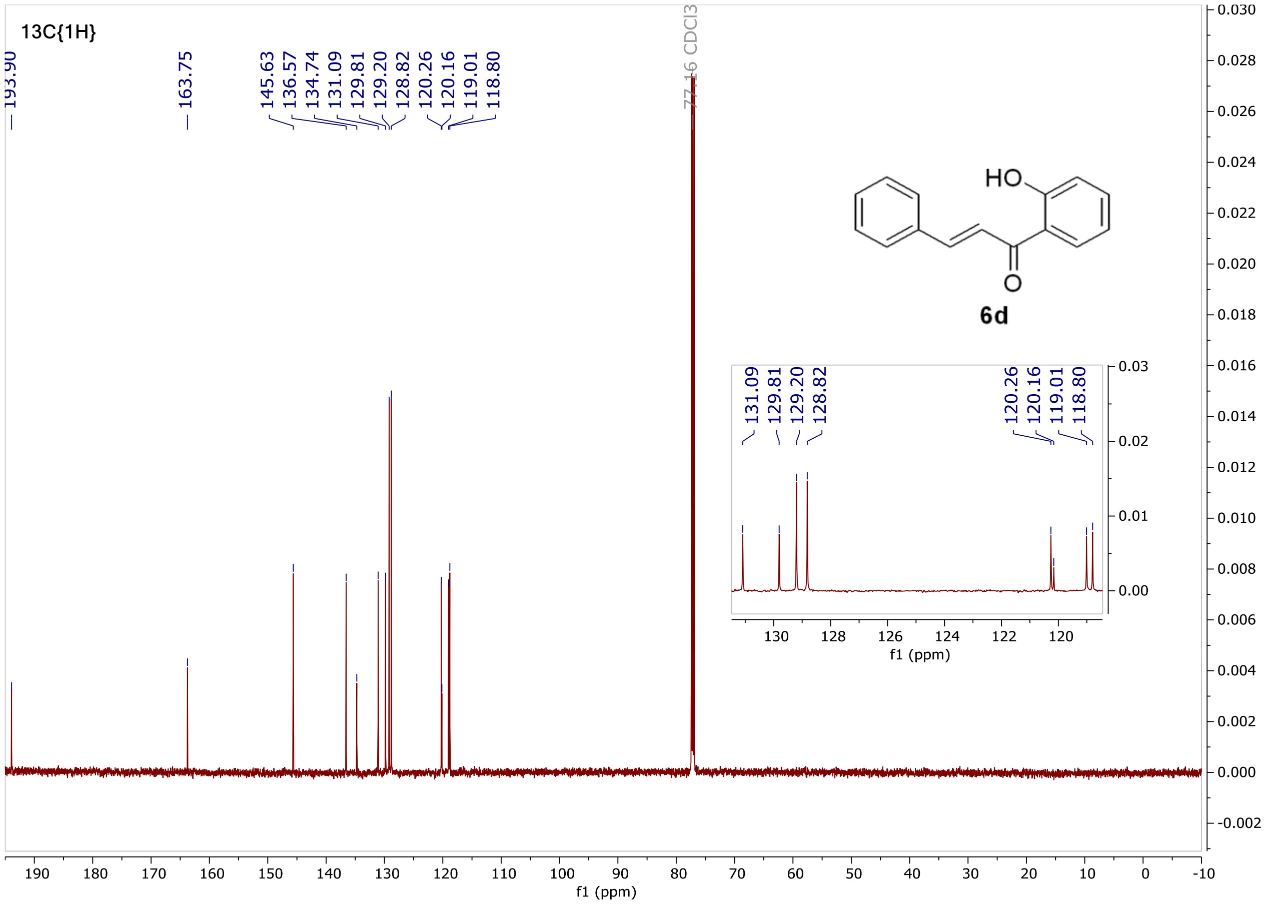
13C{1H}
193.90
163.75
145.63
136.57
134.74
131.09
129.81
129.20
128.82
120.26
120.16
119.01
118.80
77.16 CDCl3
HO
O
6d
f1 (ppm)

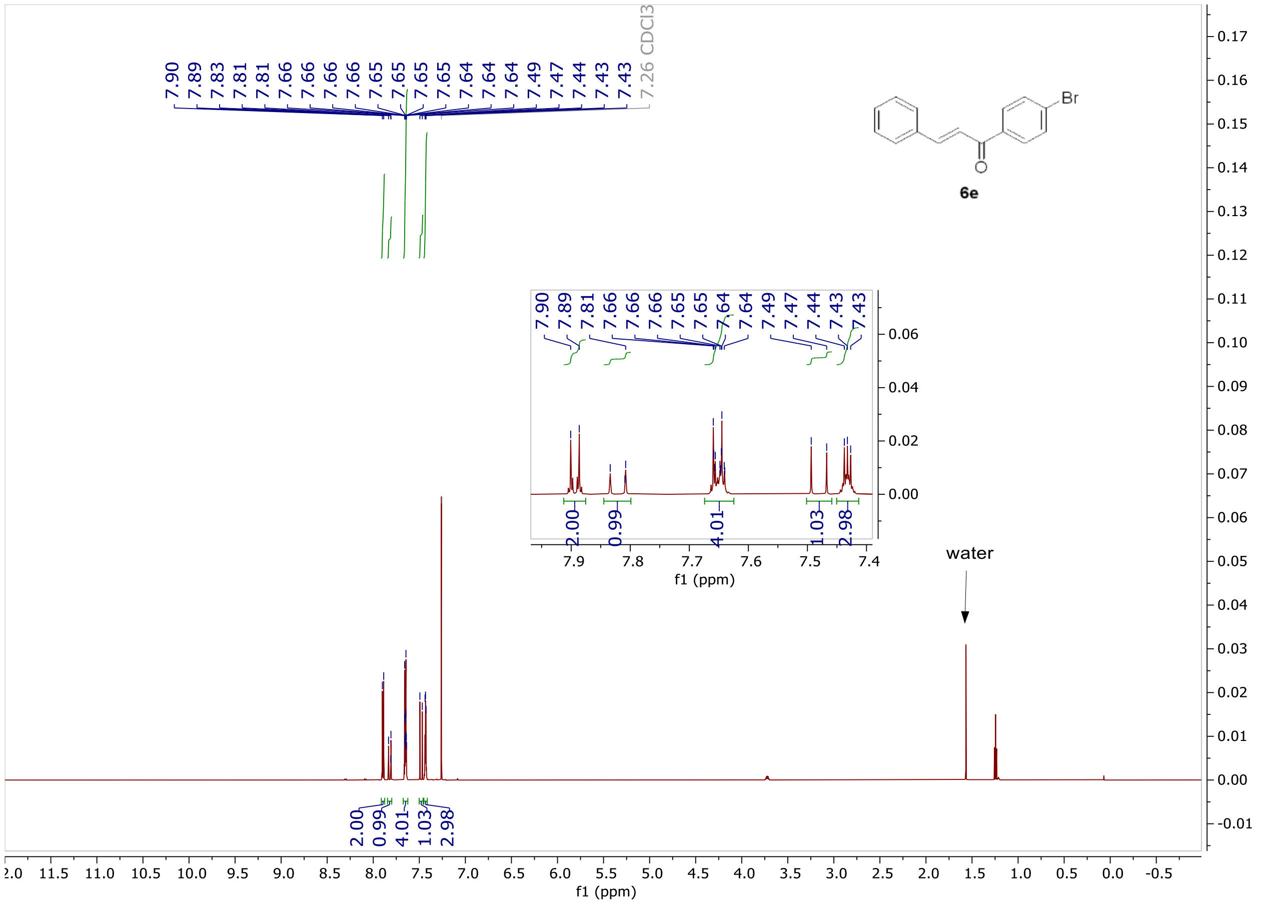
7.90
7.89
7.83
7.81
7.81
7.66
7.66
7.66
7.66
7.65
7.65
7.65
7.65
7.64
7.64
7.64
7.49
7.47
7.44
7.43
7.43
7.26 CDCl3
Br
O
6e
water
2.00
0.99
4.01
1.03
2.98
f1 (ppm)

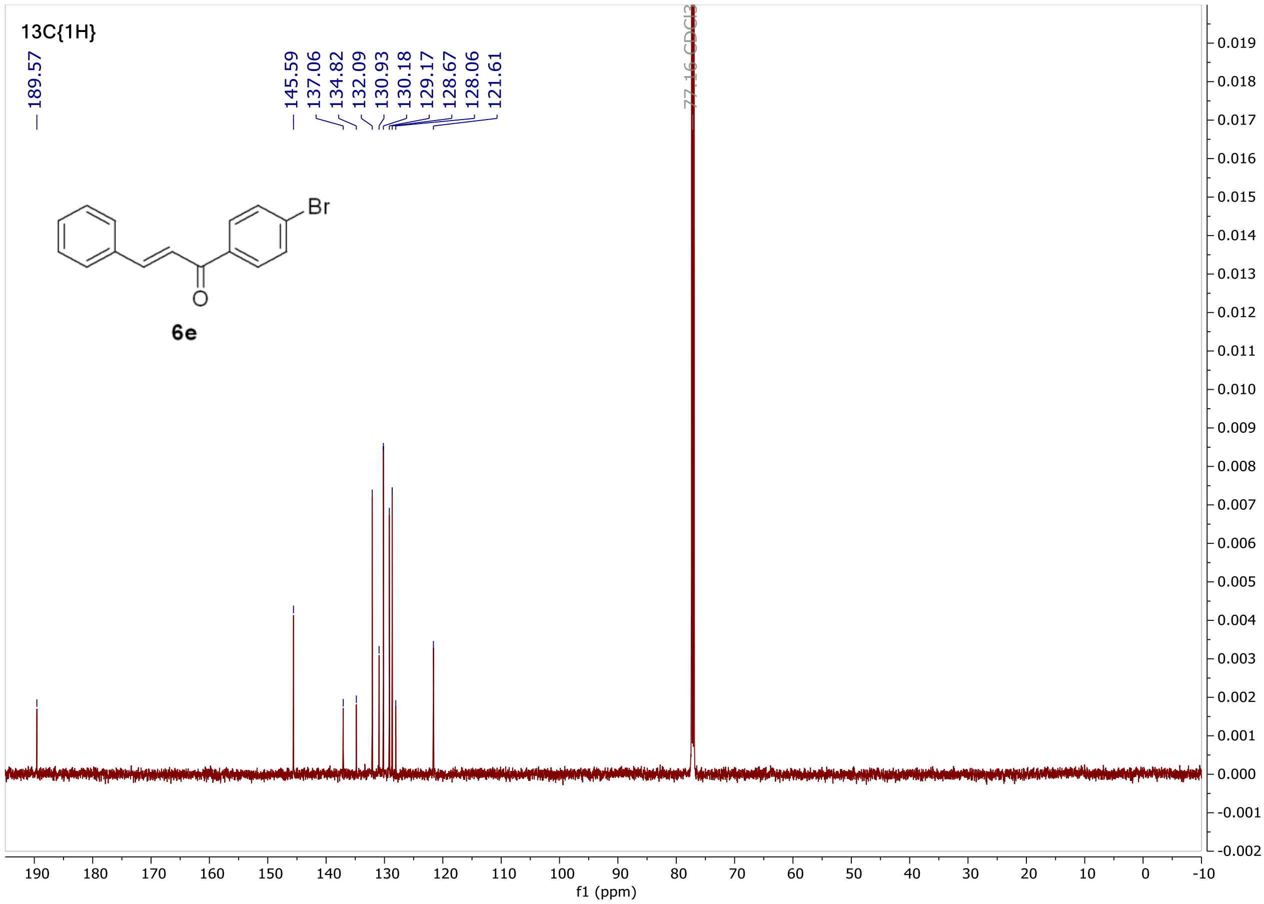
13C{1H}
189.57
145.59
137.06
134.82
132.09
130.93
130.18
129.17
128.67
128.06
121.61
77.16 CDCl3
Br
O
6e
f1 (ppm)

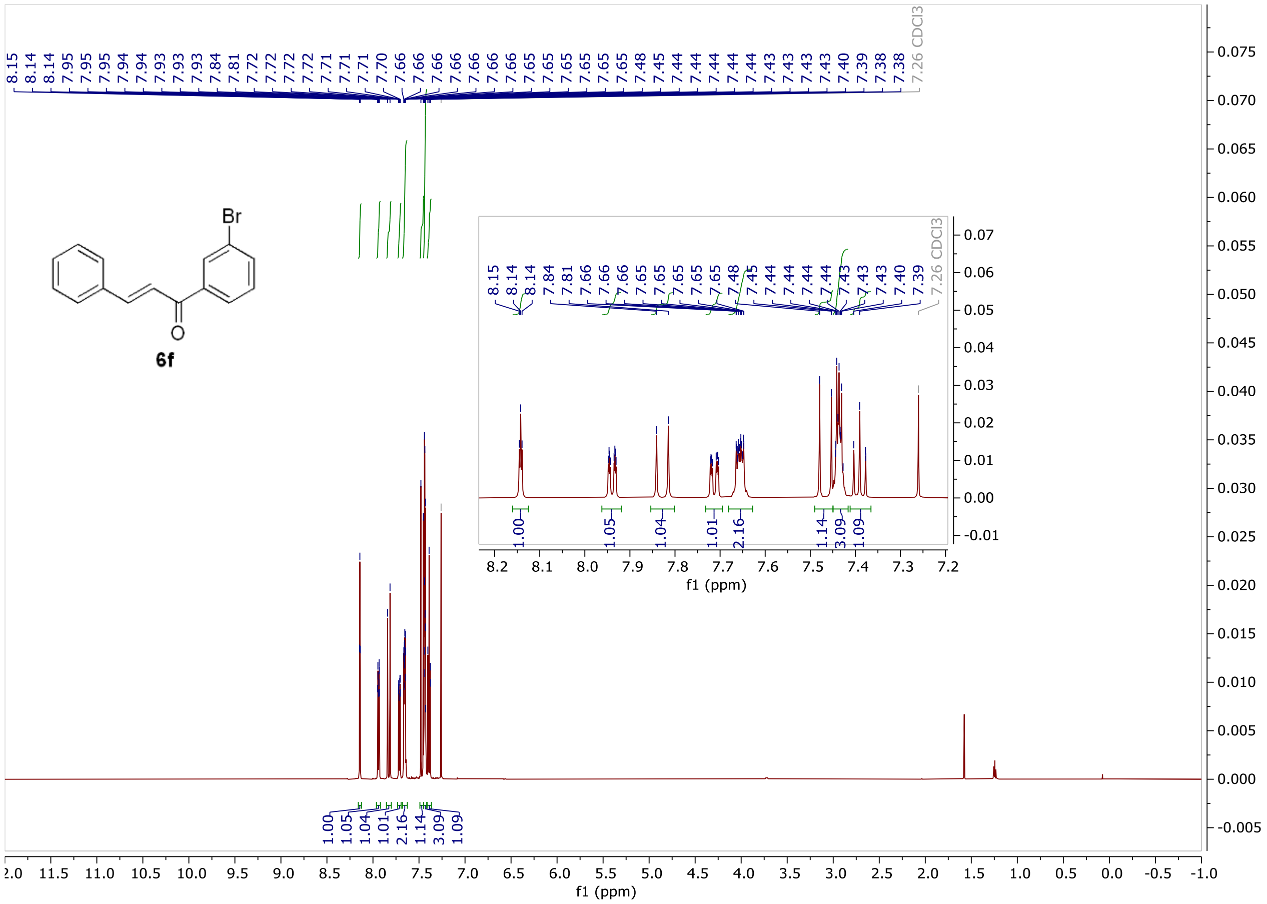
8.15 8.14 8.14 7.95 7.95 7.95 7.94 7.93 7.93 7.93 7.84 7.81 7.72 7.72 7.72 7.72 7.71 7.71 7.71 7.70 7.66 7.66 7.66 7.66 7.66 7.65 7.65 7.65 7.65 7.65 7.48 7.45 7.44 7.44 7.44 7.44 7.44 7.43 7.43 7.43 7.43 7.40 7.39 7.38 7.38
7.26 CDCl3
Br
O
6f
1.00 1.05 1.04 1.01 2.16 1.14 3.09 1.09
f1 (ppm)

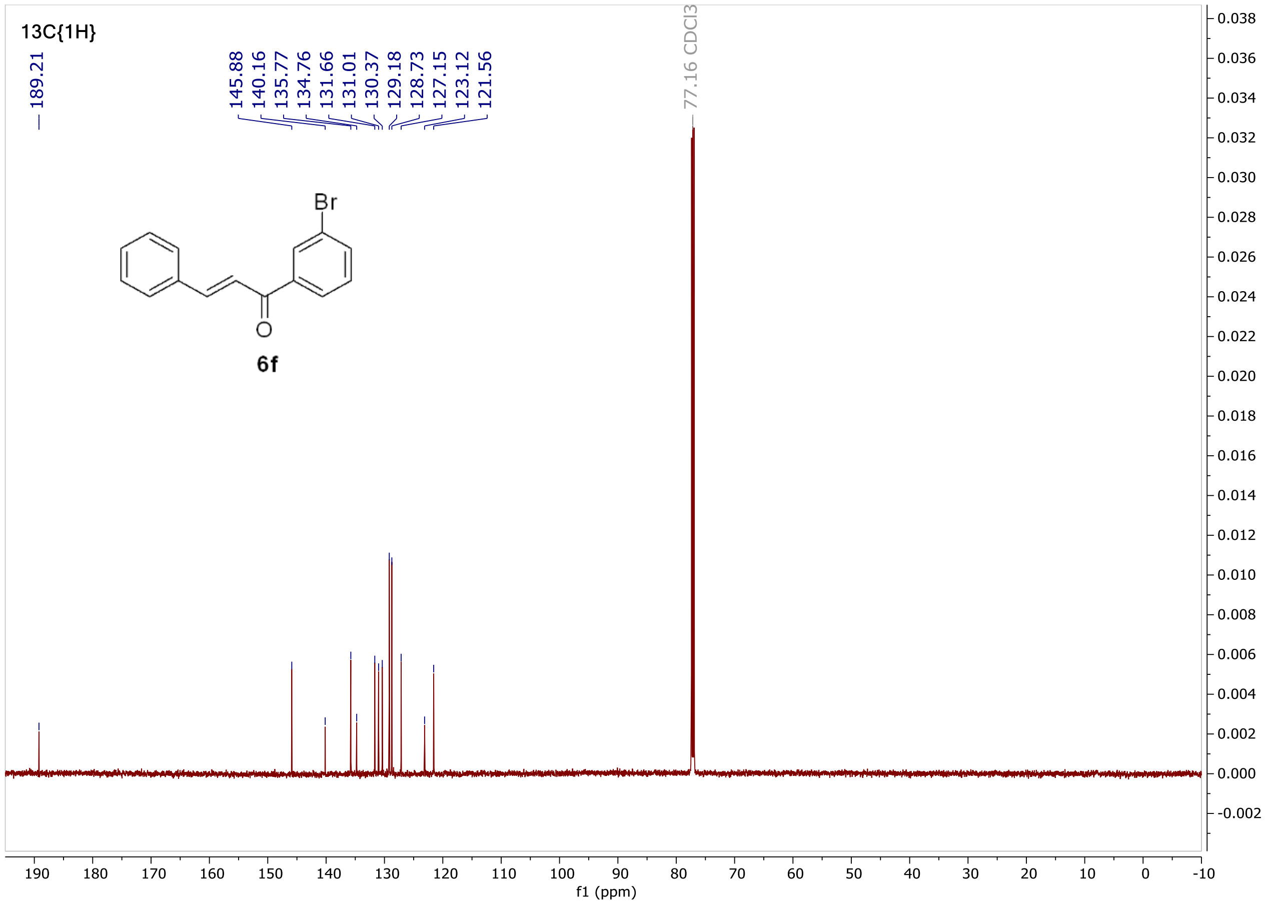
13C{1H}
189.21
145.88 140.16 135.77 134.76 131.66 131.01 130.37 129.18 128.73 127.15 123.12 121.56
77.16 CDCl3
Br
O
6f
f1 (ppm)

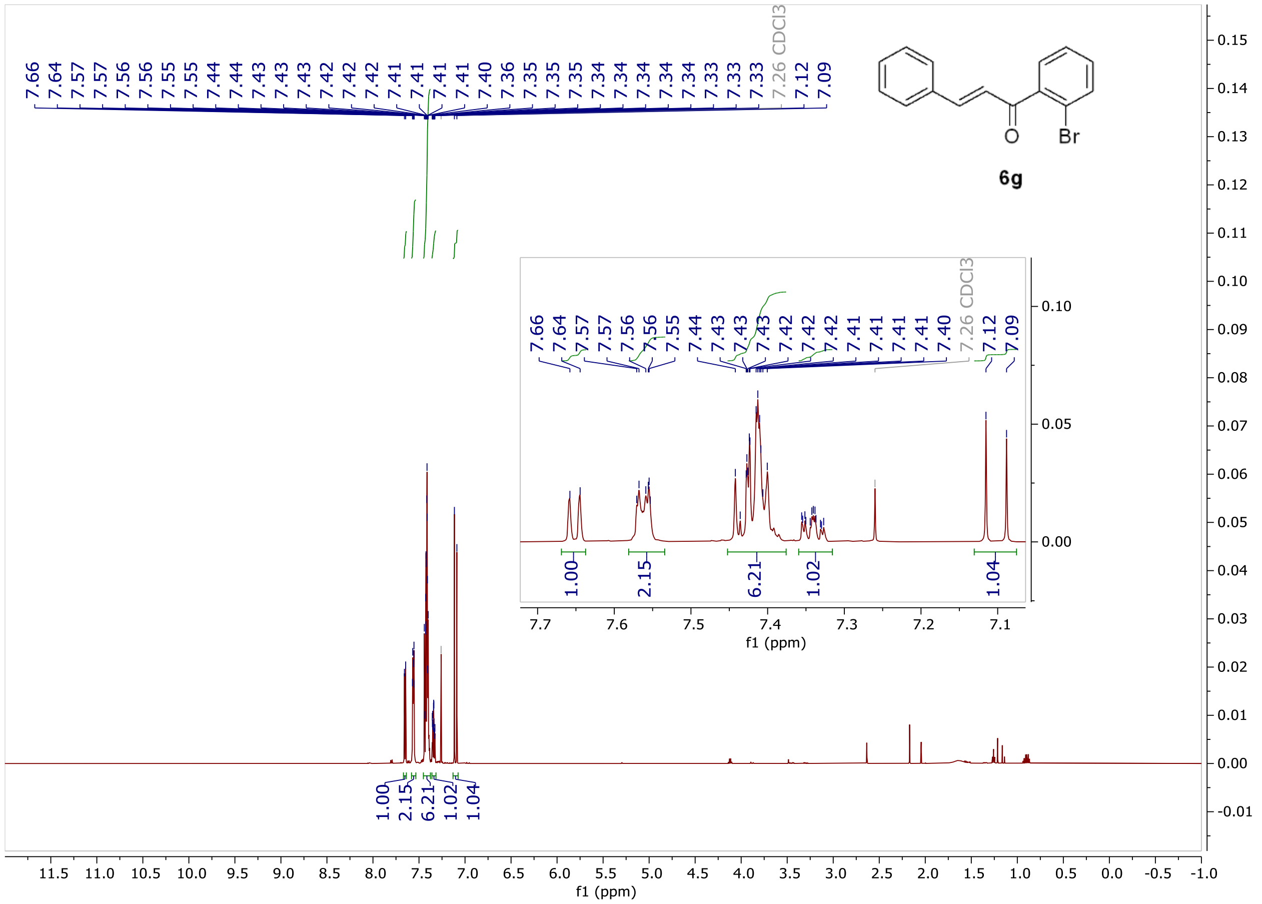
7.66 7.64 7.57 7.57 7.56 7.56 7.55 7.55 7.44 7.44 7.43 7.43 7.43 7.42 7.42 7.42 7.41 7.41 7.41 7.41 7.40 7.36 7.35 7.35 7.35 7.34 7.34 7.34 7.34 7.34 7.33 7.33 7.33 7.26 CDCl3 7.12 7.09
6g
O
Br
1.00 2.15 6.21 1.02 1.04
f1 (ppm)

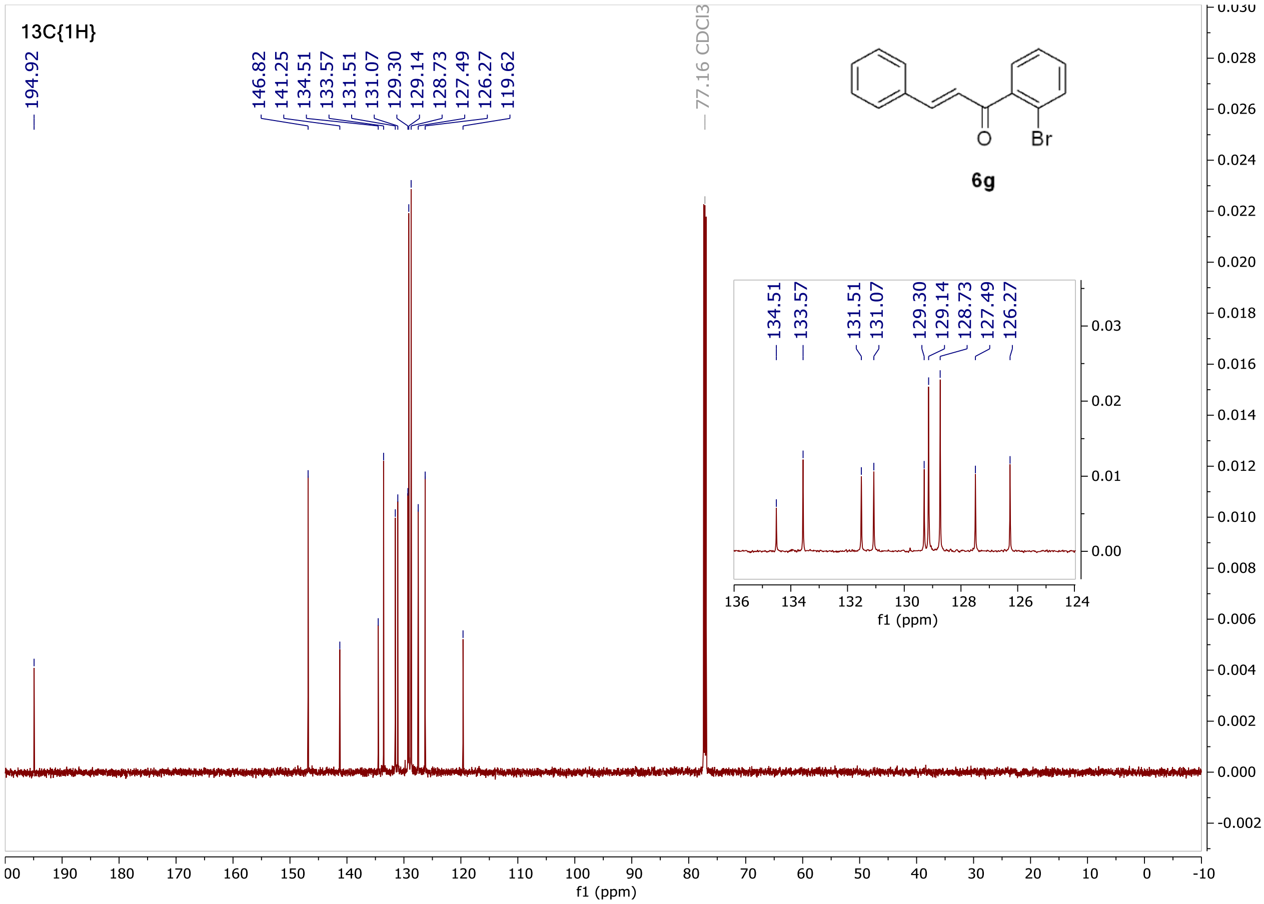
13C{1H}
194.92
146.82 141.25 134.51 133.57 131.51 131.07 129.30 129.14 128.73 127.49 126.27 119.62
77.16 CDCl3
6g
O
Br
f1 (ppm)

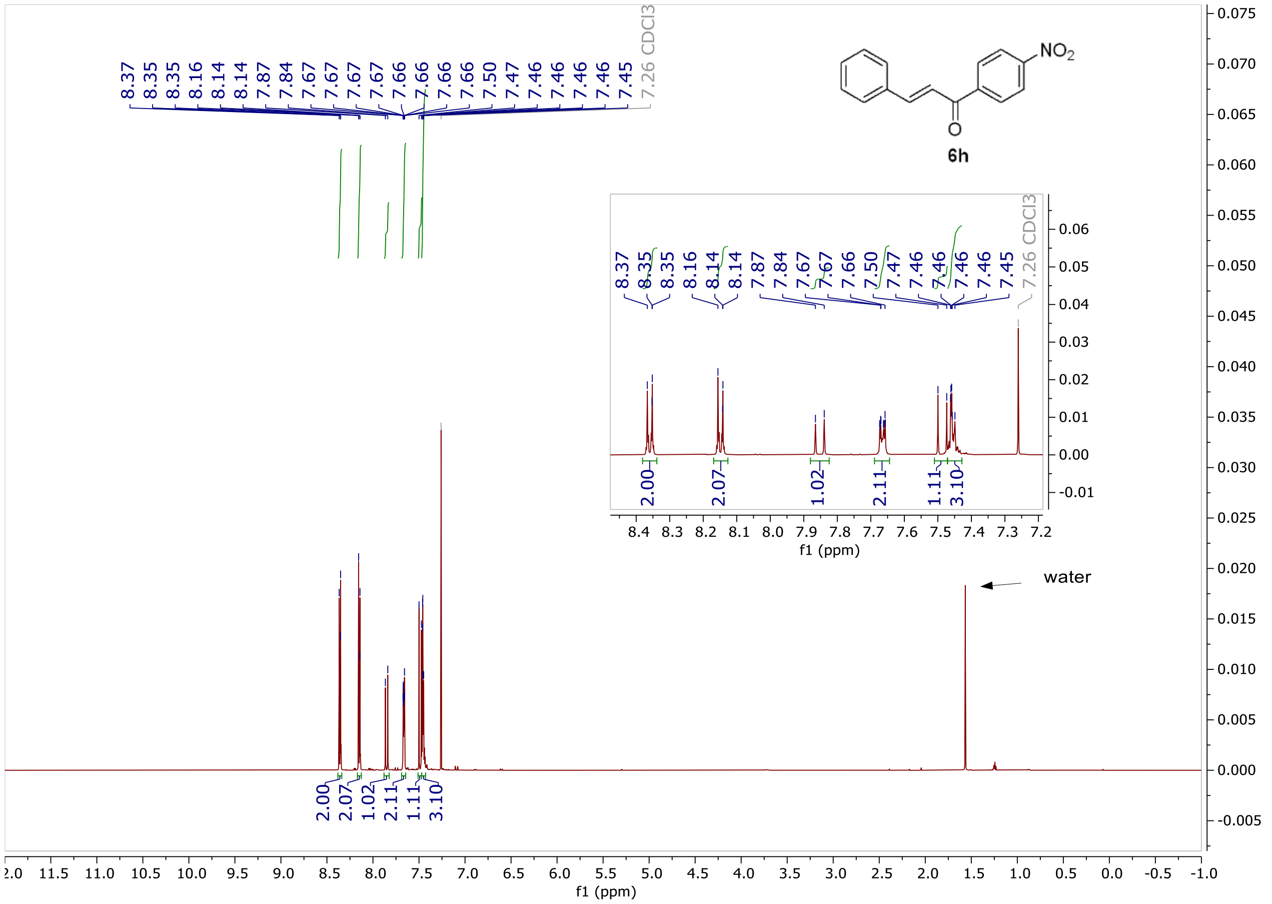
8.37 8.35 8.35 8.16 8.14 8.14 7.87 7.84 7.67 7.67 7.67 7.67 7.66 7.66 7.66 7.50 7.47 7.46 7.46 7.46 7.46 7.45
7.26 CDCl3
NO2
6h
water
2.00 2.07 1.02 2.11 1.11 3.10
f1 (ppm)

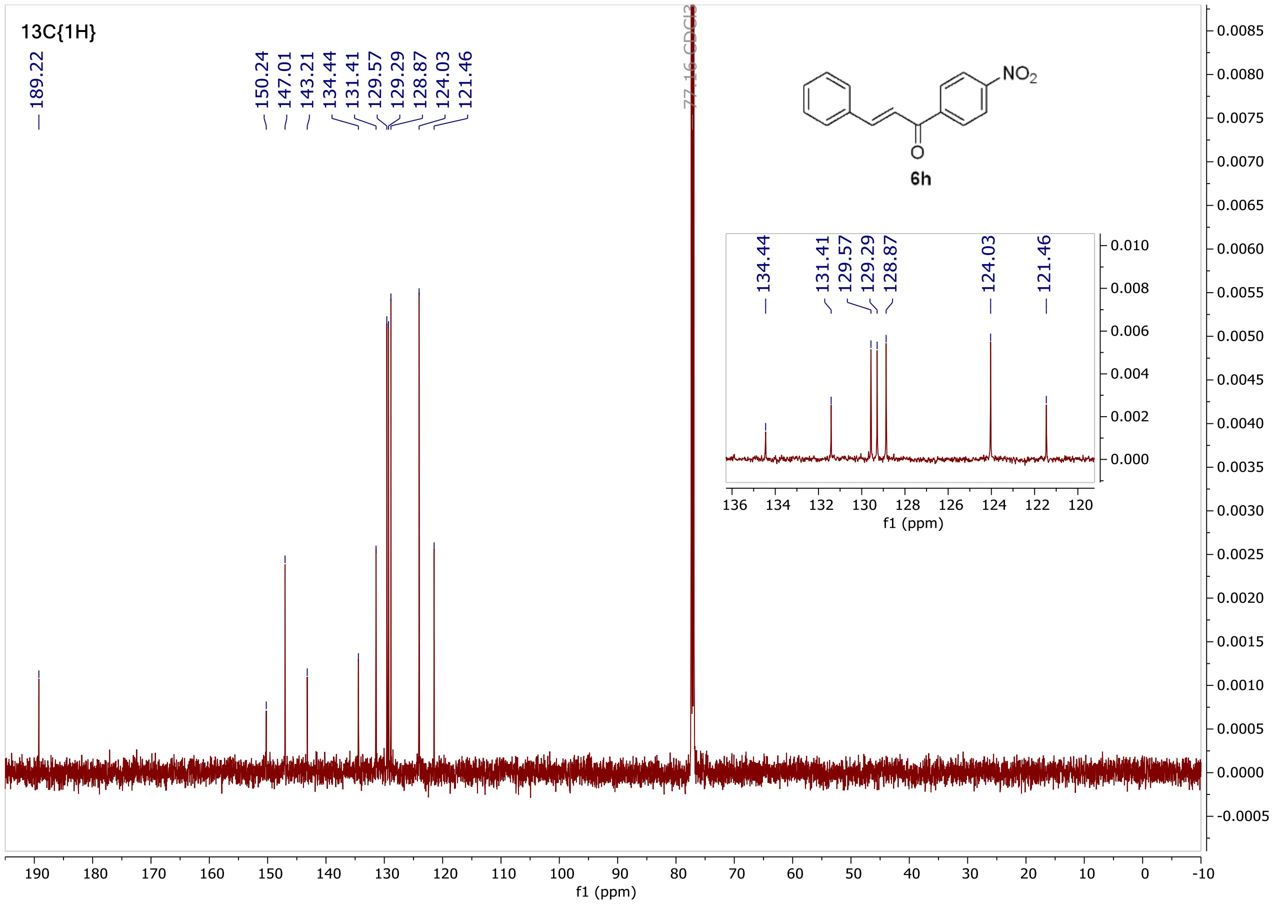
13C{1H}
189.22
150.24 147.01 143.21 134.44 131.41 129.57 129.29 128.87 124.03 121.46
77.16 CDCl3
NO2
6h
f1 (ppm)

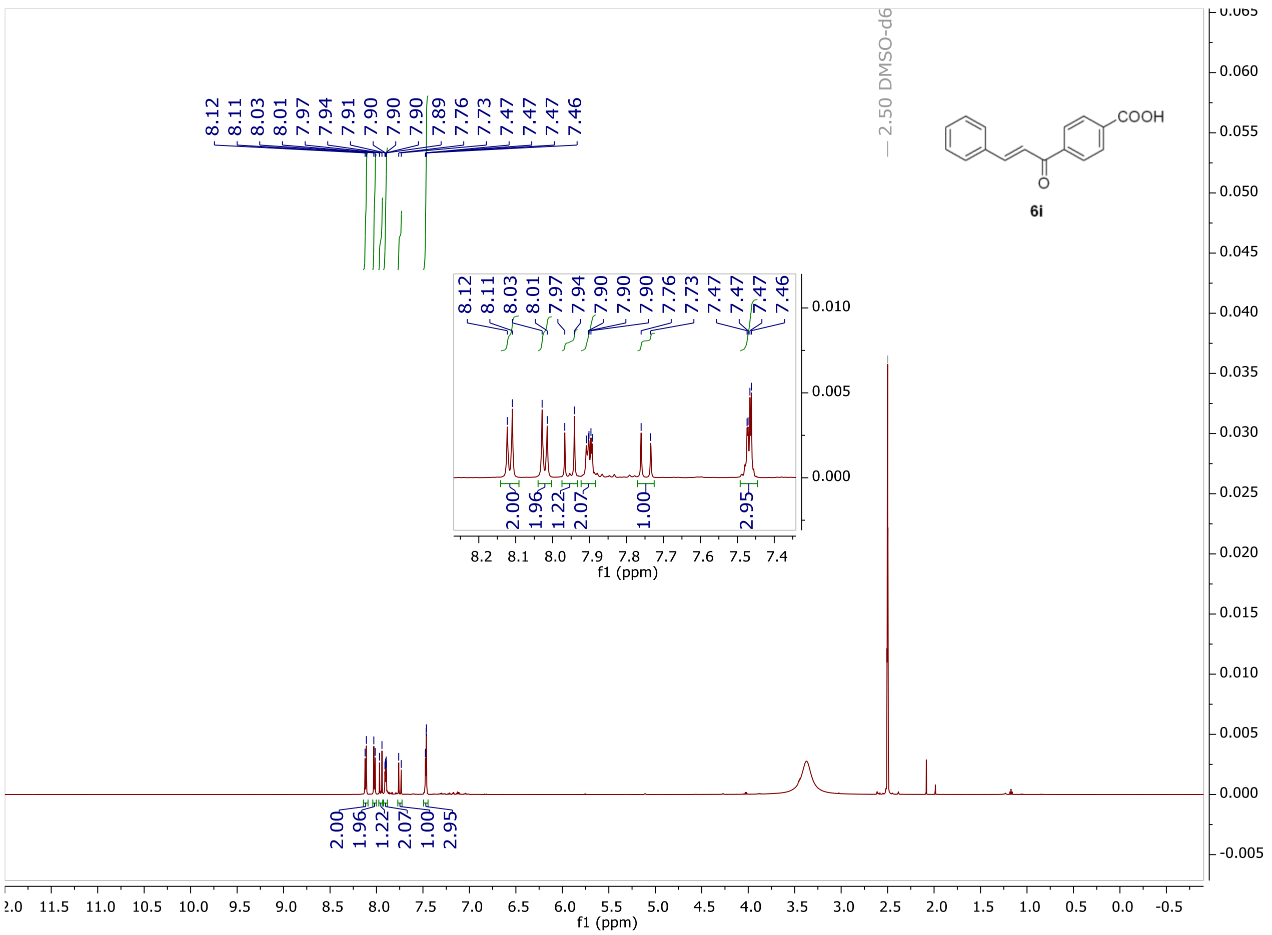

8.12 8.11 8.03 8.01 7.97 7.94 7.91 7.90 7.90 7.90 7.89 7.76 7.73 7.47 7.47 7.47 7.46
2.50 DMSO-d6
COOH
6i
2.00 1.96 1.22 2.07 1.00 2.95
f1 (ppm)

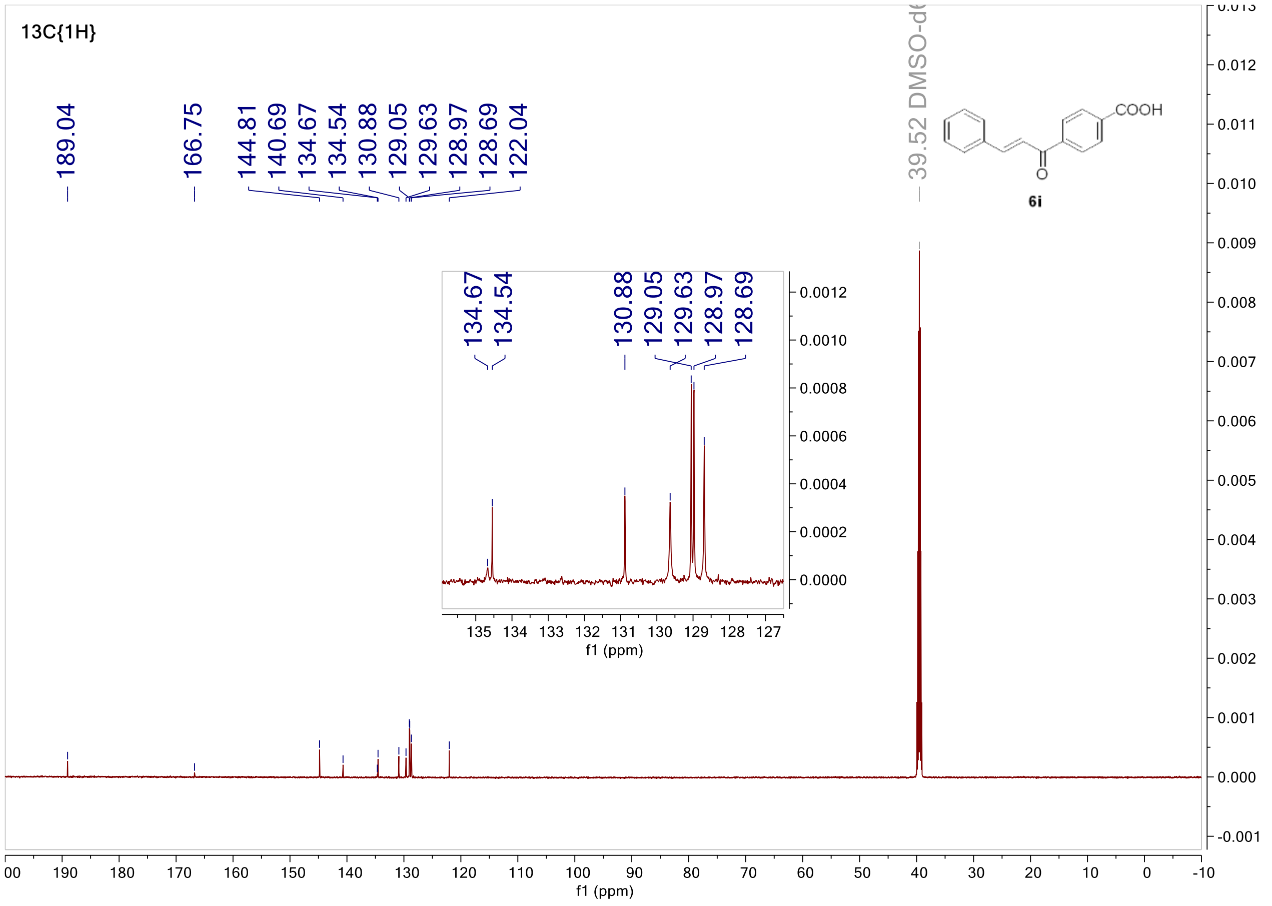

13C{1H}
189.04
166.75
144.81 140.69 134.67 134.54 130.88 129.05 129.63 128.97 128.69 122.04
39.52 DMSO-d6
COOH
6i
f1 (ppm)

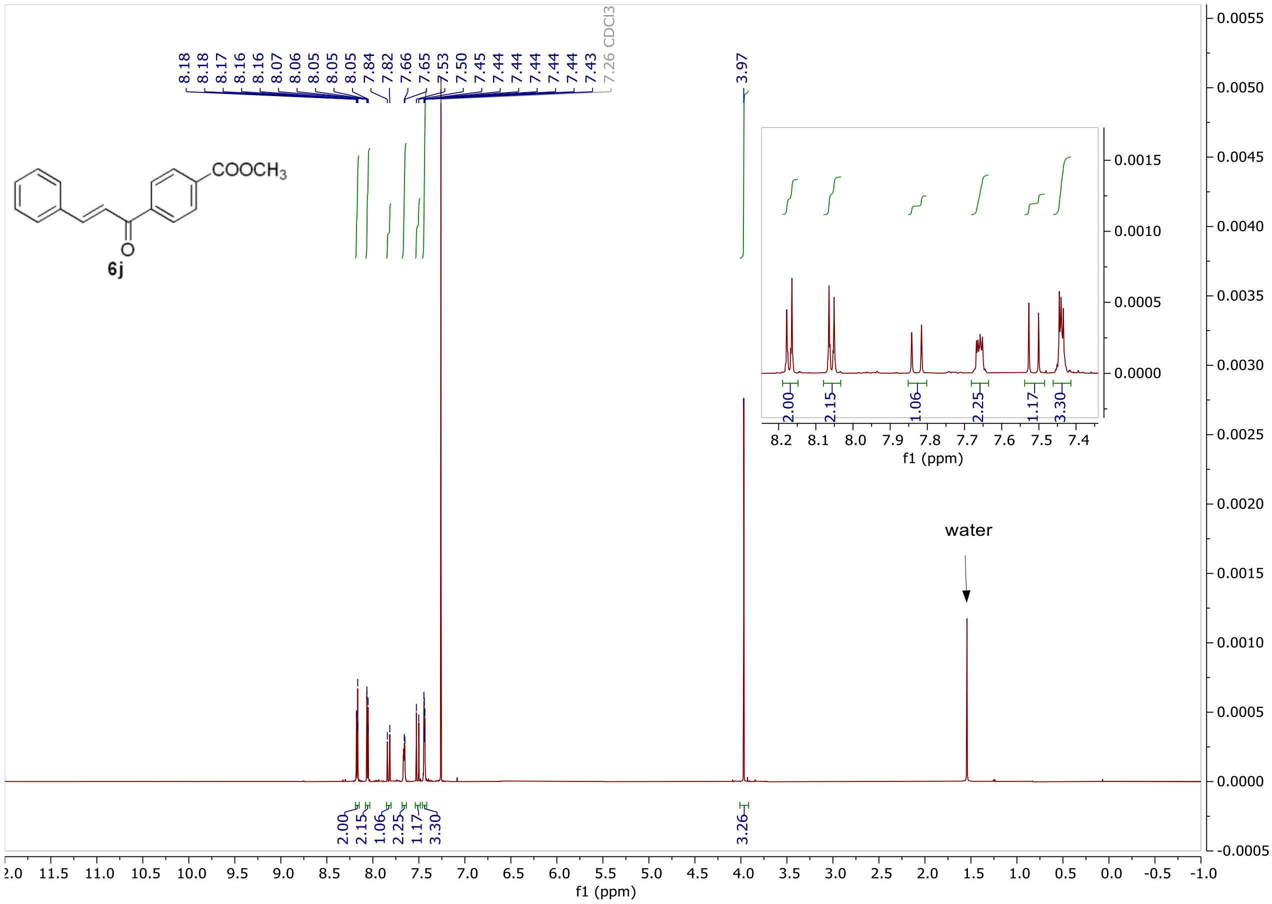

8.18
8.18
8.17
8.16
8.16
8.07
8.06
8.05
8.05
8.05
7.84
7.82
7.66
7.65
7.53
7.50
7.45
7.44
7.44
7.44
7.44
7.44
7.43
7.26 CDCl3
3.97
COOCH3
O
6j
water
2.00
2.15
1.06
2.25
1.17
3.30
3.26
f1 (ppm)

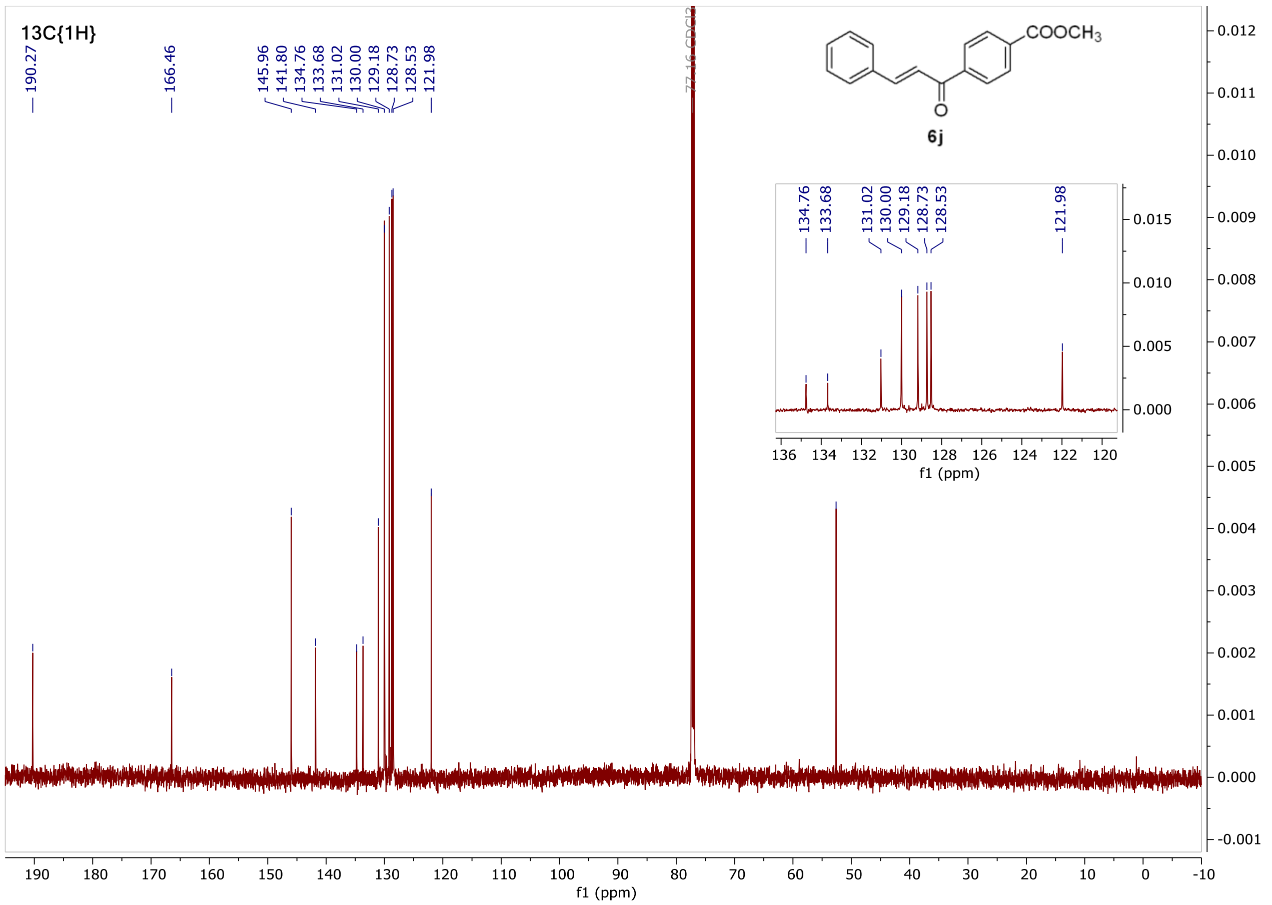

13C{1H}
190.27
166.46
145.96
141.80
134.76
133.68
131.02
130.00
129.18
128.73
128.53
121.98
77.16 CDCl3
COOCH3
O
6j
f1 (ppm)

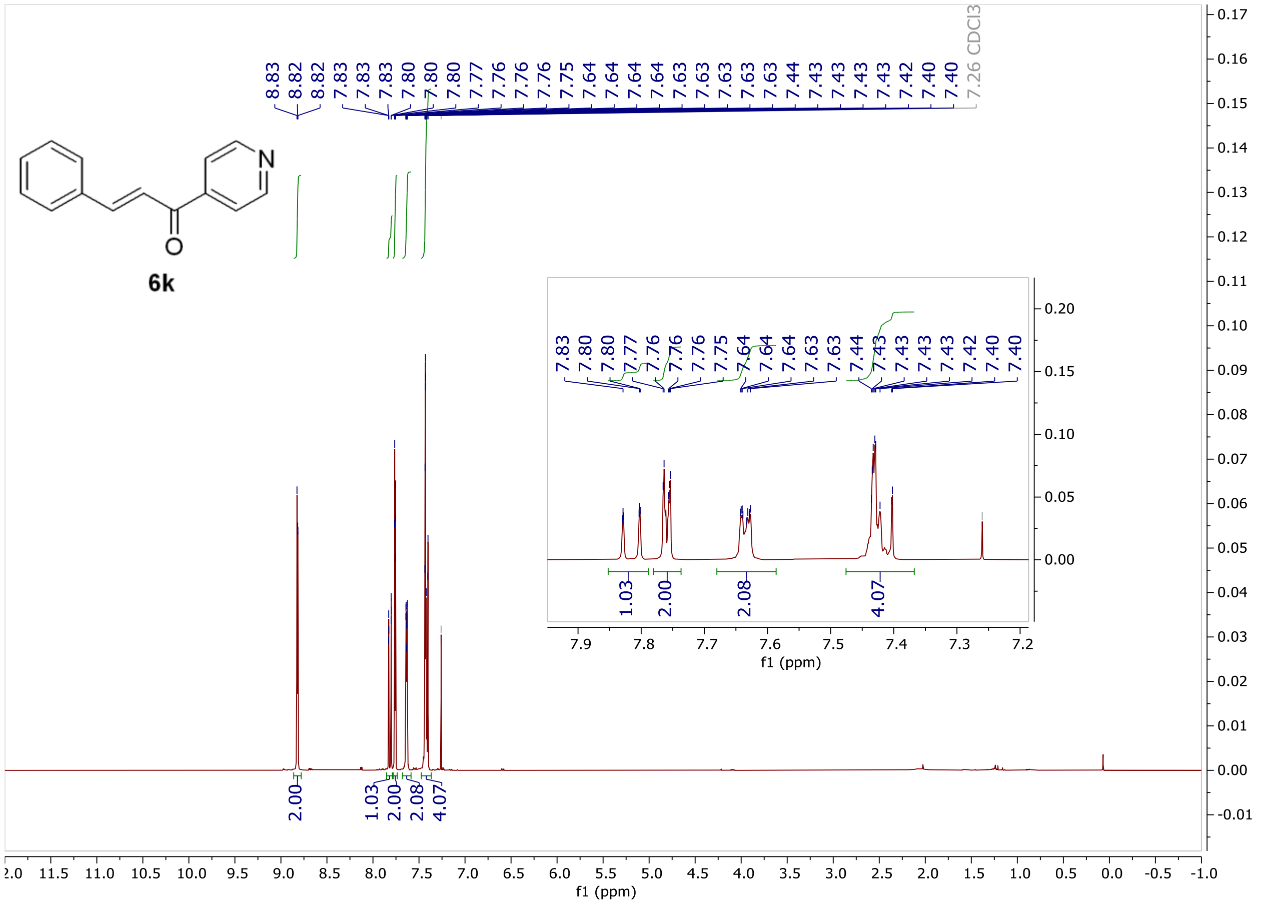
6k
8.83 8.82 8.82 7.83 7.83 7.83 7.80 7.80 7.80 7.77 7.76 7.76 7.76 7.75 7.64 7.64 7.64 7.63 7.63 7.63 7.63 7.44 7.43 7.43 7.43 7.42 7.40 7.40
7.26 CDCl3
2.00 1.03 2.00 2.08 4.07
f1 (ppm)

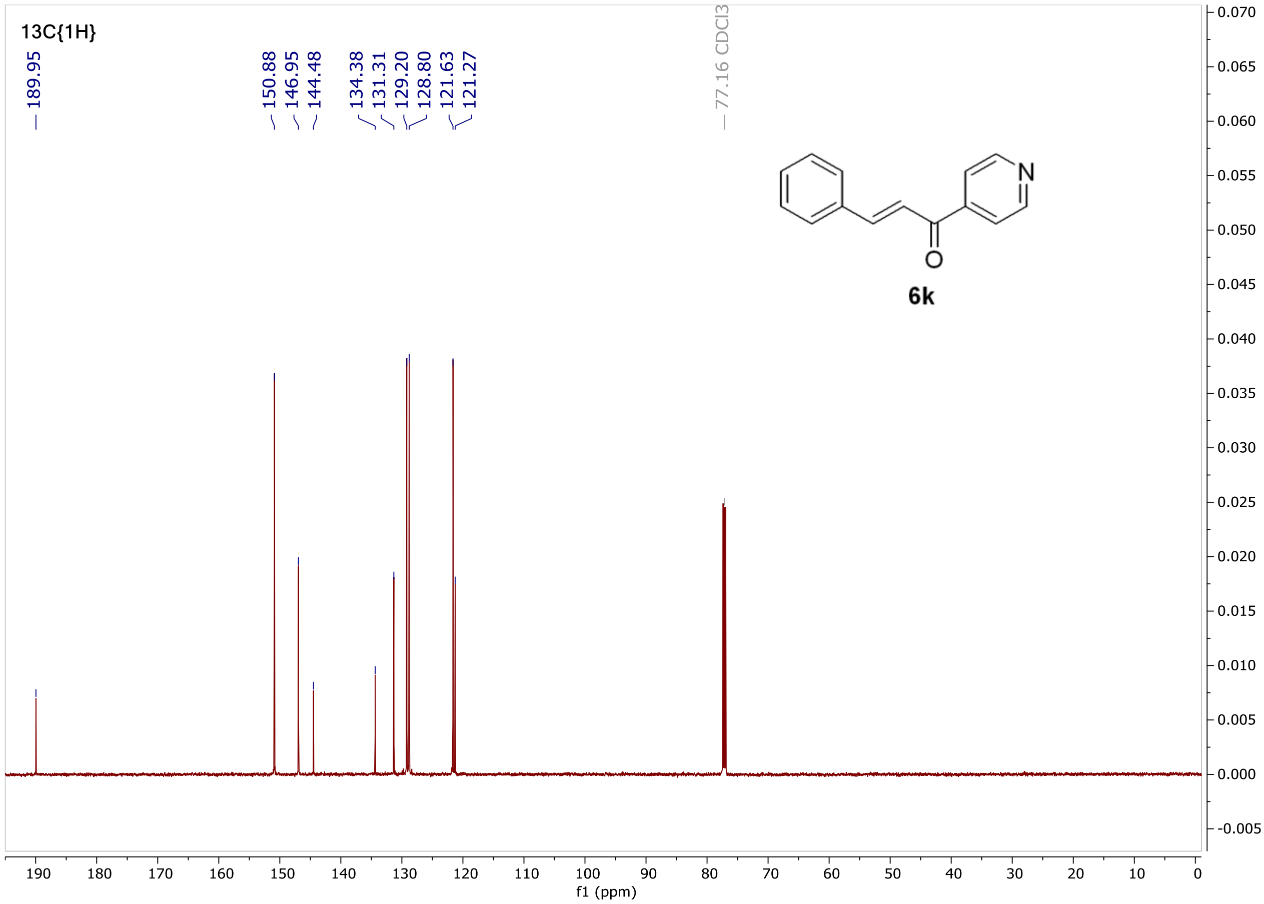
13C{1H}
189.95
150.88 146.95 144.48
134.38 131.31 129.20 128.80 121.63 121.27
77.16 CDCl3
6k
f1 (ppm)

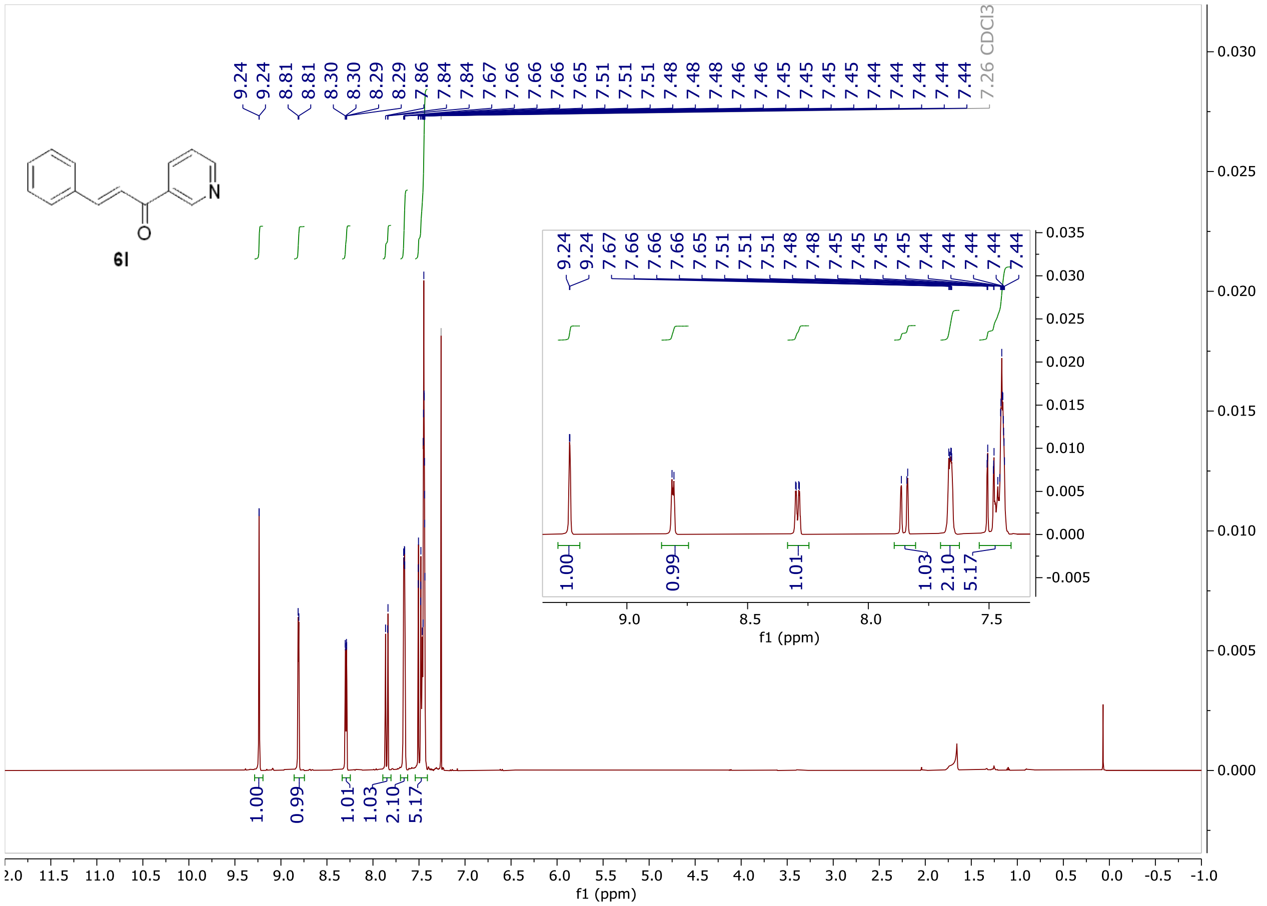
9.24
9.24
8.81
8.81
8.30
8.30
8.29
8.29
7.86
7.84
7.84
7.67
7.66
7.66
7.65
7.51
7.51
7.51
7.48
7.48
7.48
7.46
7.46
7.45
7.45
7.45
7.44
7.44
7.44
7.44
7.44
7.26 CDCl3
6l
1.00
0.99
1.01
1.03
2.10
5.17
f1 (ppm)

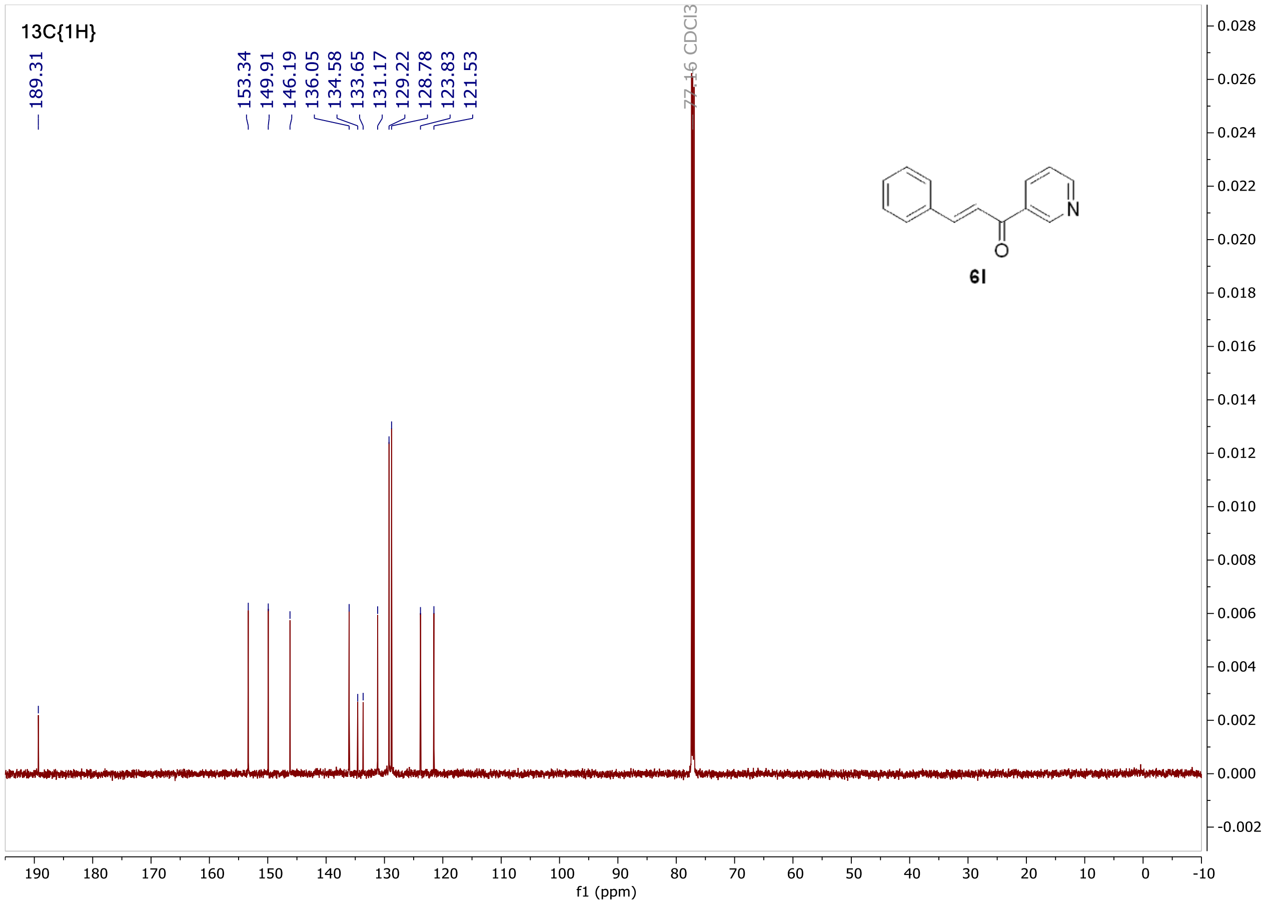
13C{1H}
189.31
153.34
149.91
146.19
136.05
134.58
133.65
131.17
129.22
128.78
123.83
121.53
77.16 CDCl3
6l
f1 (ppm)

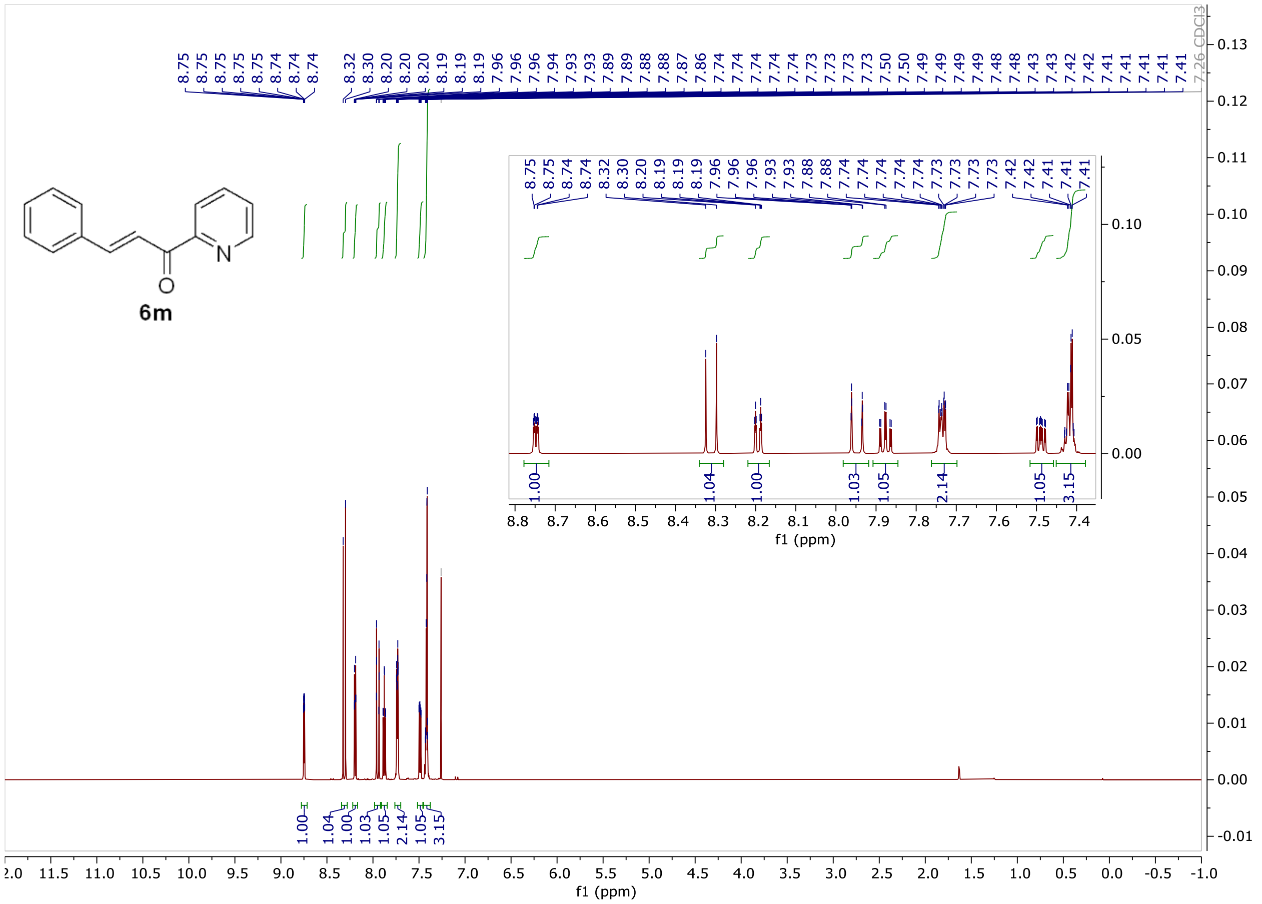

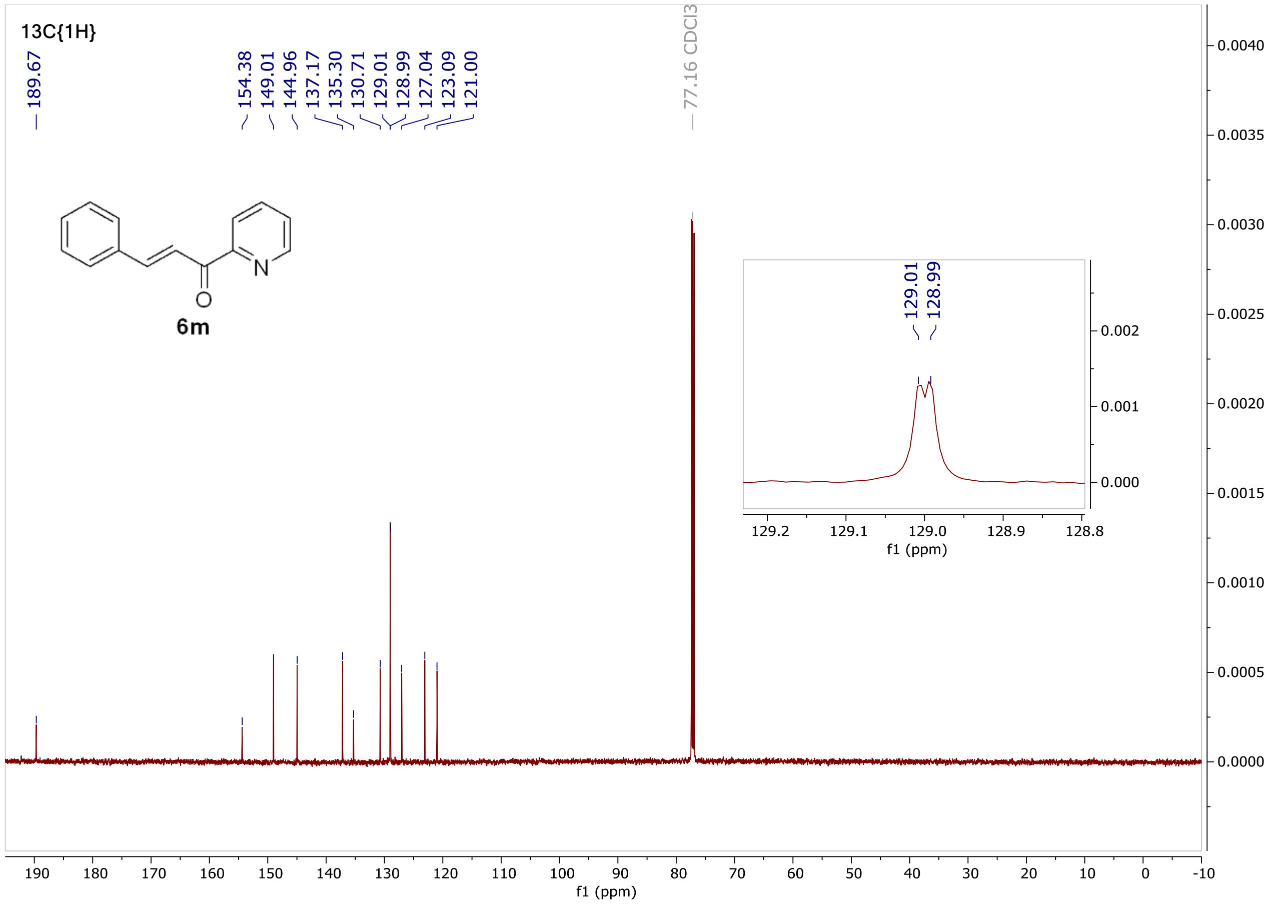

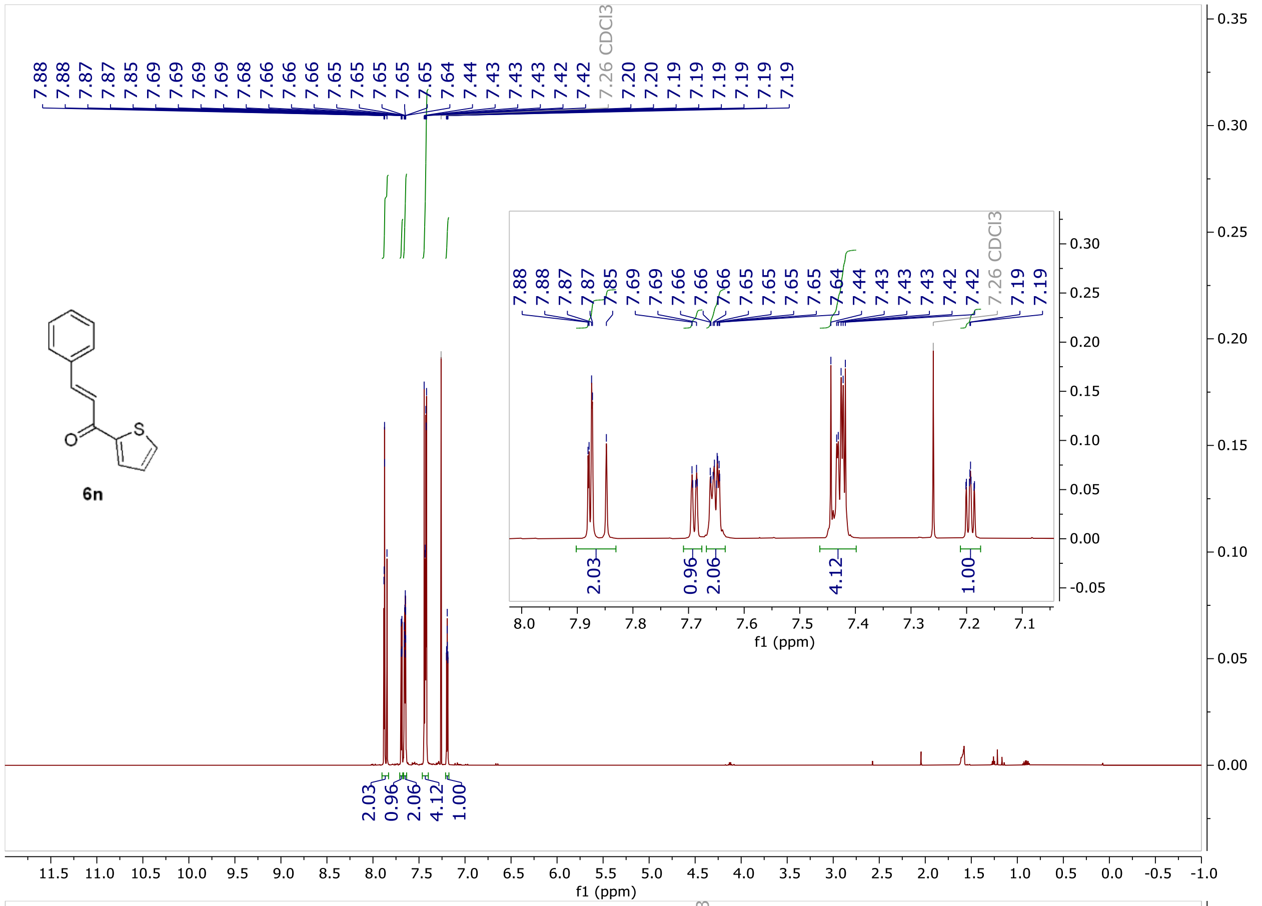
7.88 7.88 7.87 7.87 7.85 7.69 7.69 7.69 7.69 7.68 7.66 7.66 7.65 7.65 7.65 7.65 7.64 7.44 7.43 7.43 7.43 7.42 7.42 7.26 CDCl3 7.20 7.20 7.19 7.19 7.19 7.19 7.19 7.19
6n
2.03 0.96 2.06 4.12 1.00
f1 (ppm)

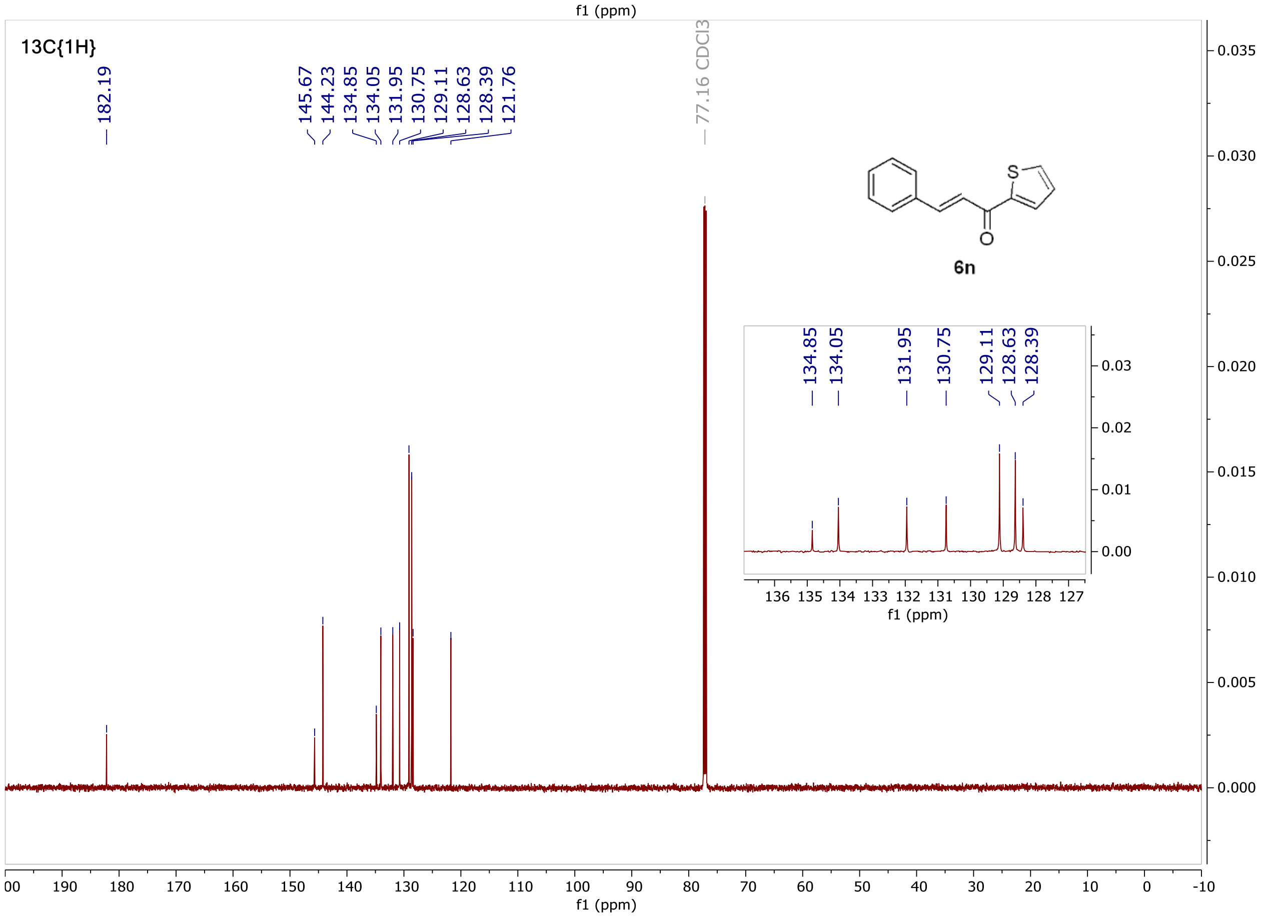
13C{1H}
182.19
145.67 144.23 134.85 134.05 131.95 130.75 129.11 128.63 128.39 121.76
77.16 CDCl3
6n
f1 (ppm)

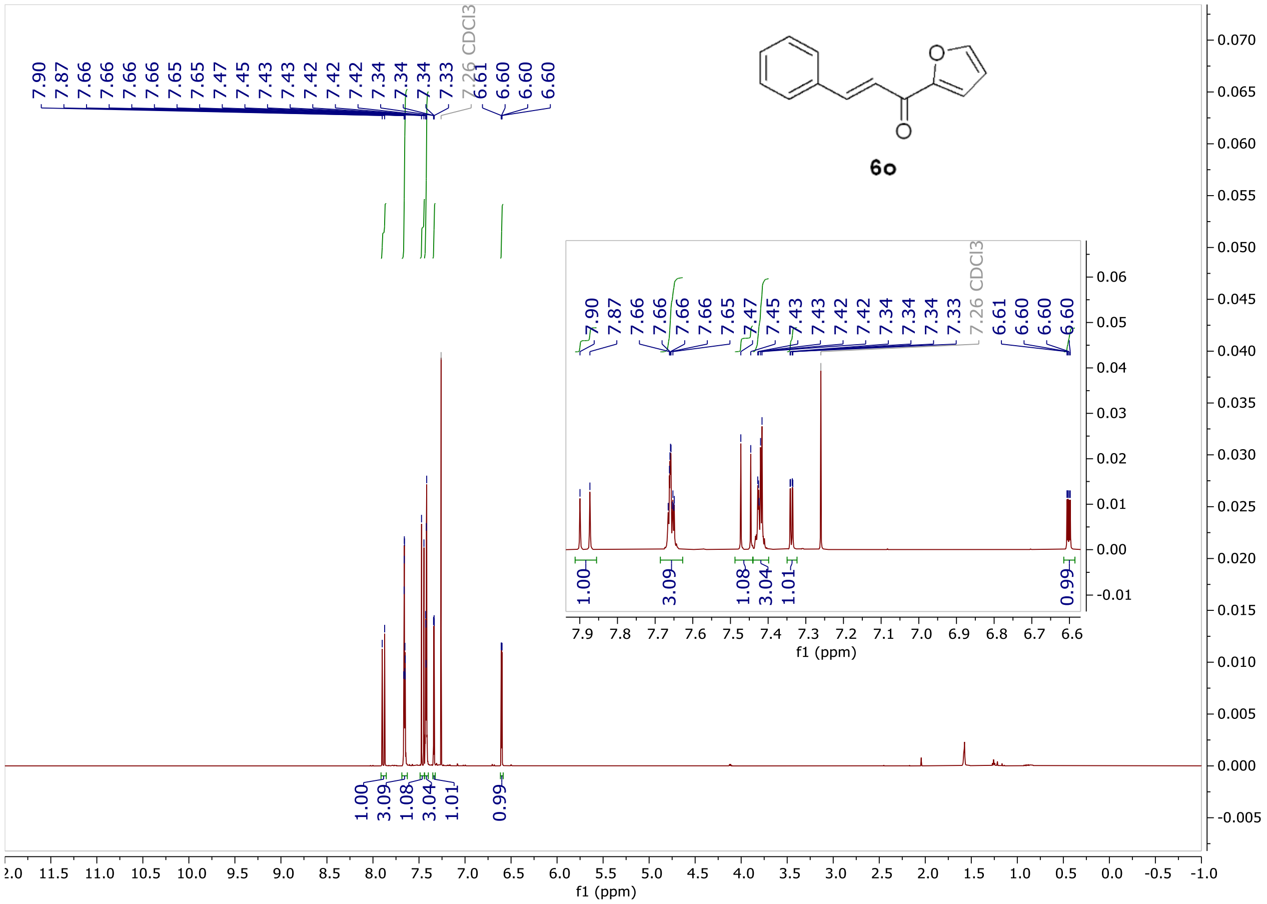
7.90 7.87 7.66 7.66 7.66 7.66 7.65 7.65 7.47 7.45 7.43 7.43 7.42 7.42 7.42 7.34 7.34 7.34 7.33
7.26 CDCl3
6.61 6.60 6.60 6.60
6o
1.00 3.09 1.08 3.04 1.01 0.99
f1 (ppm)

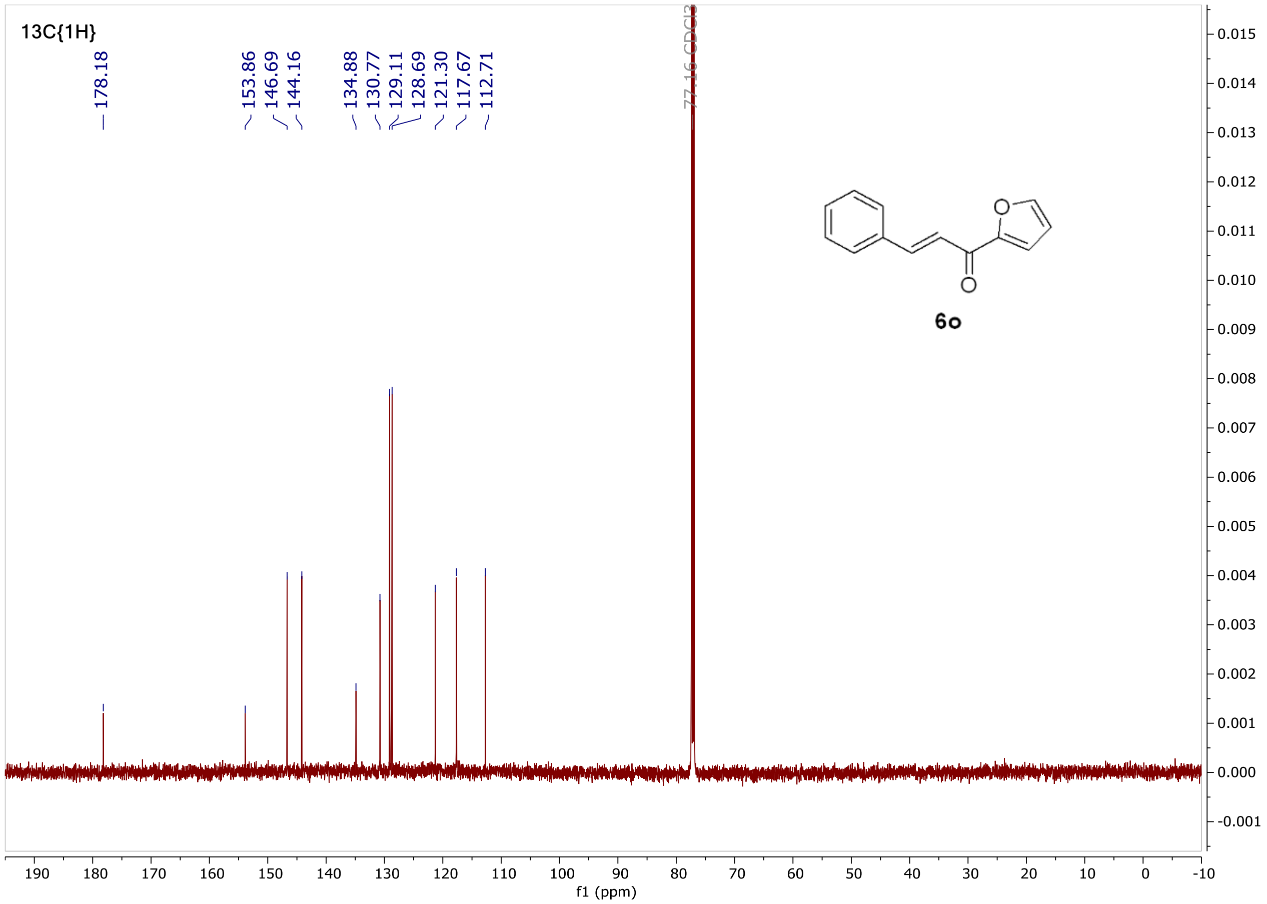
13C{1H}
178.18
153.86 146.69 144.16
134.88 130.77 129.11 128.69 121.30 117.67 112.71
77.16 CDCl3
6o
f1 (ppm)

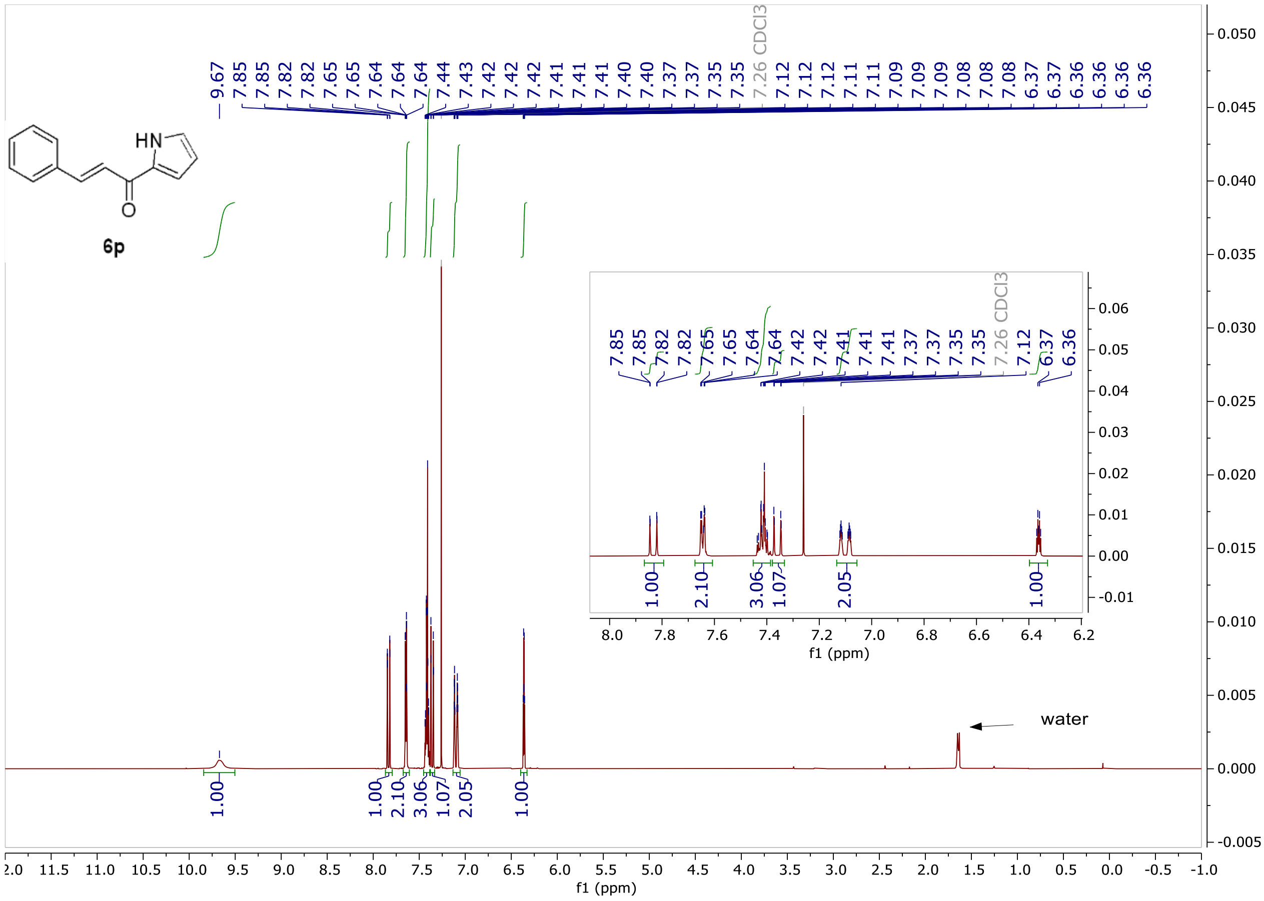
6p
water
f1 (ppm)

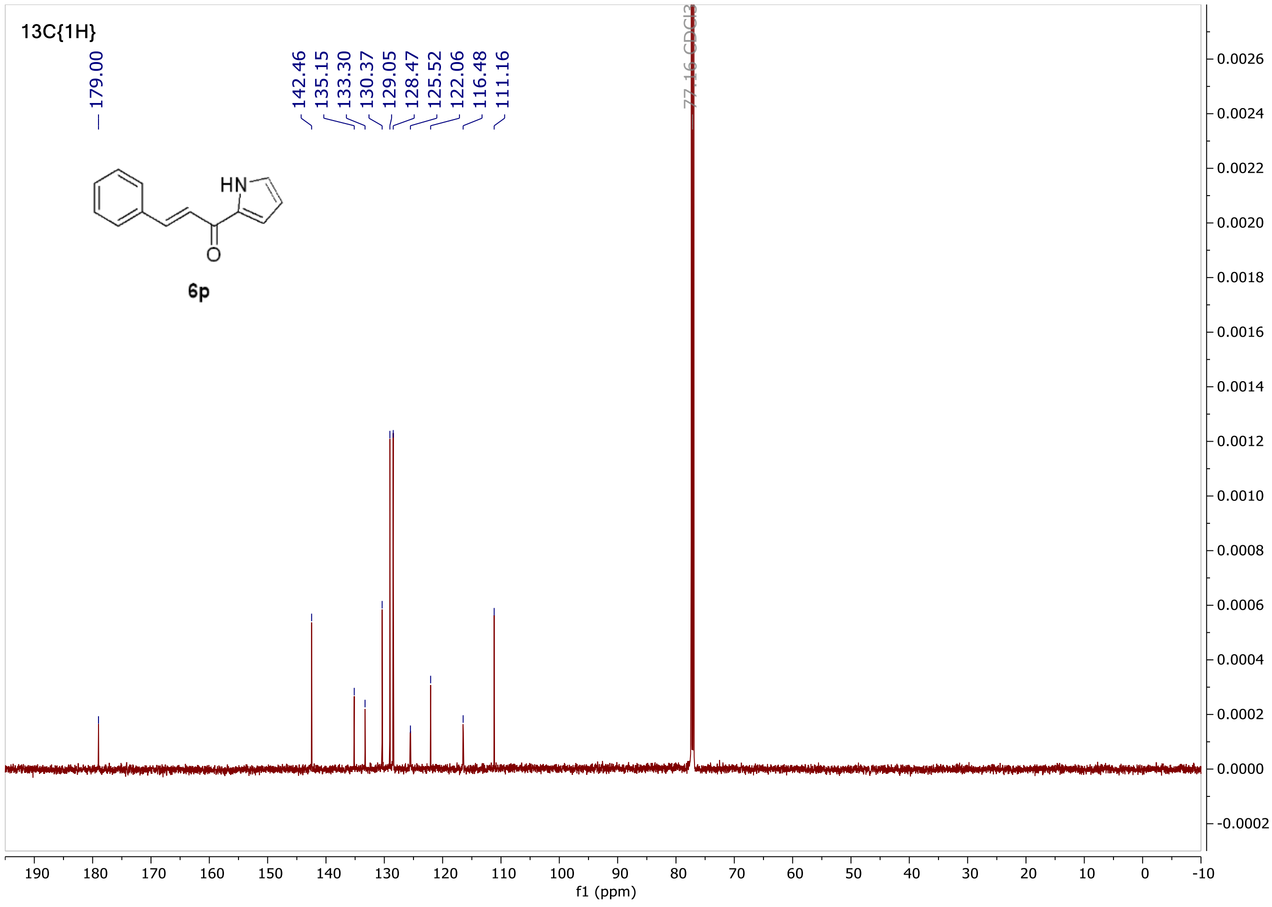
13C{1H}
179.00
142.46
135.15
133.30
130.37
129.05
128.47
125.52
122.06
116.48
111.16
77.16 CDCl3
6p
f1 (ppm)

^{1}H and ^{13}C{1H} spectra of series 7

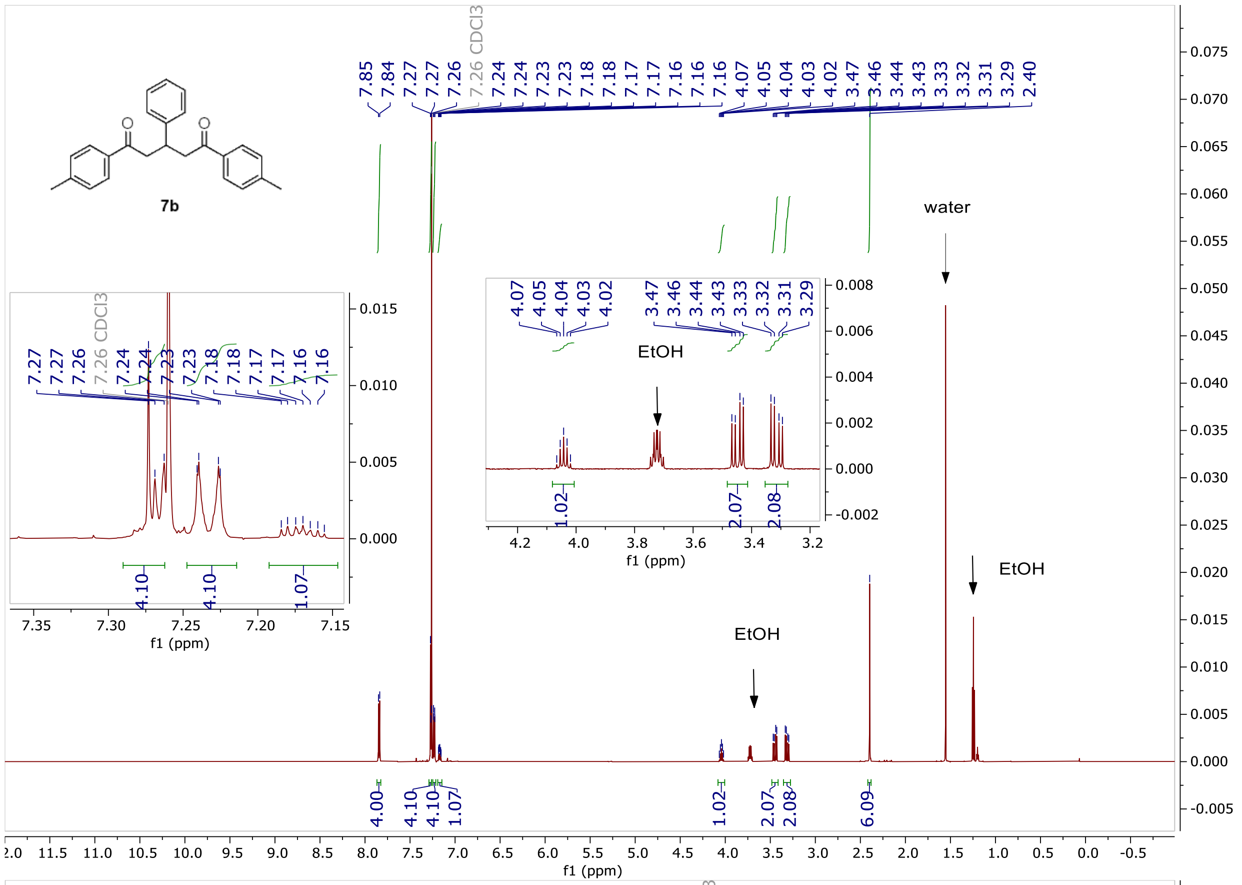

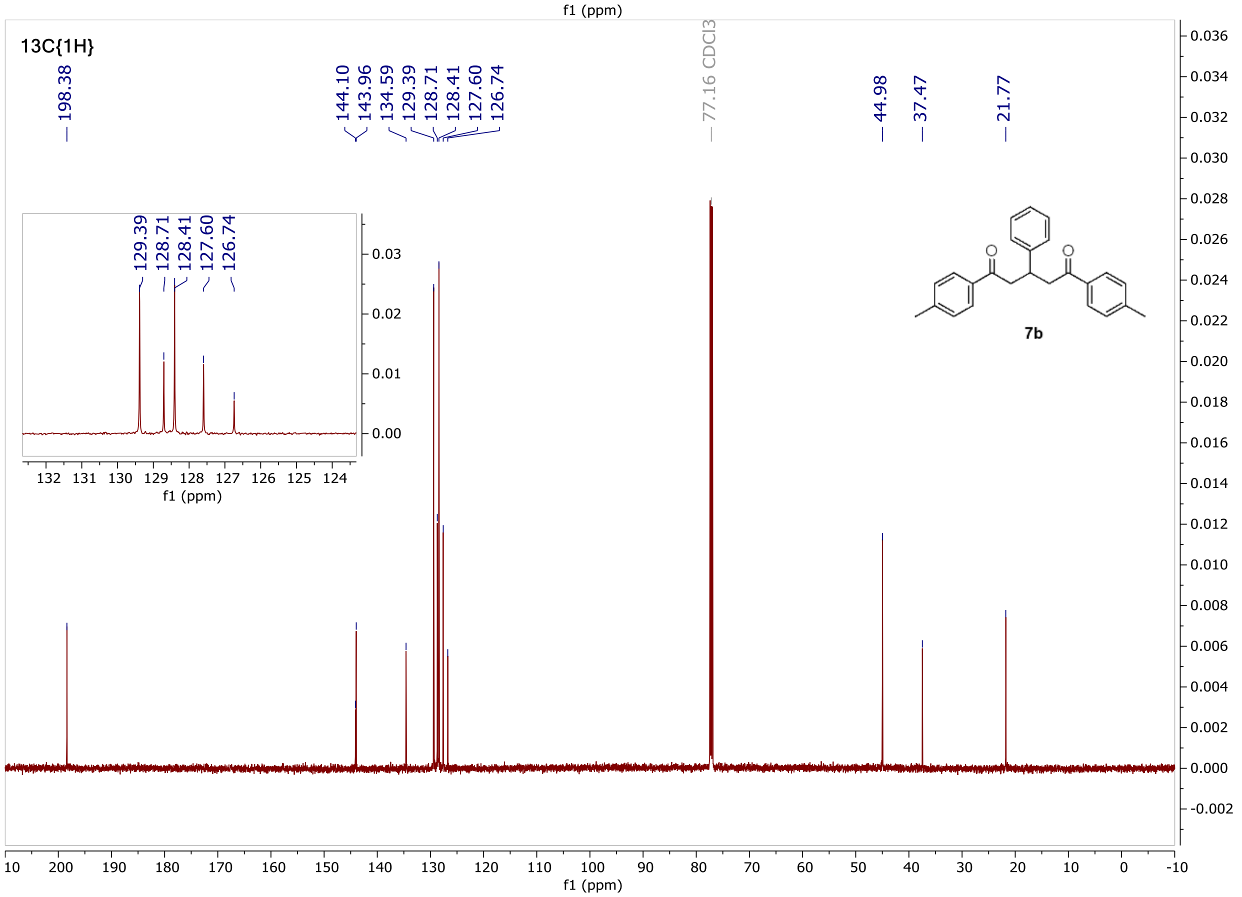

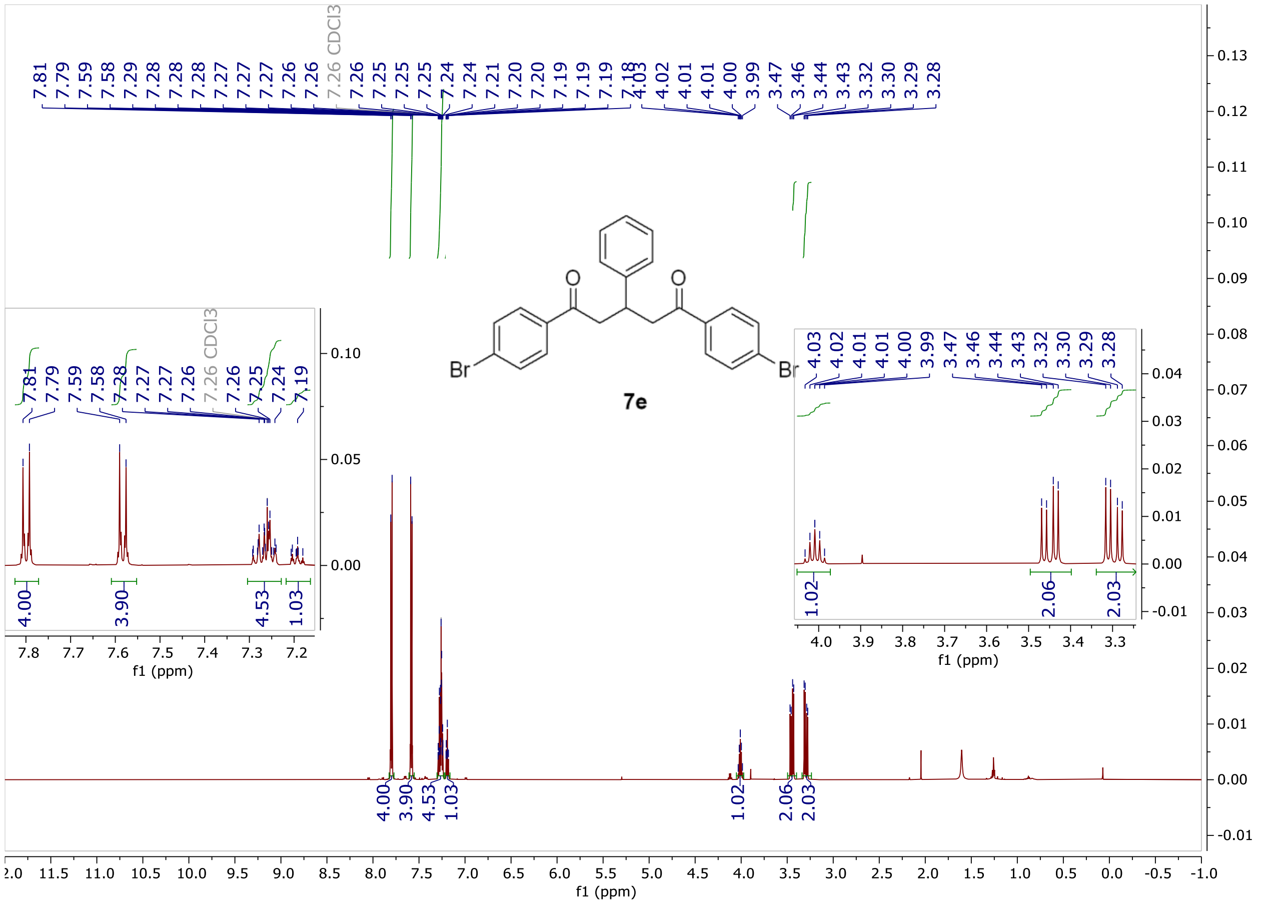

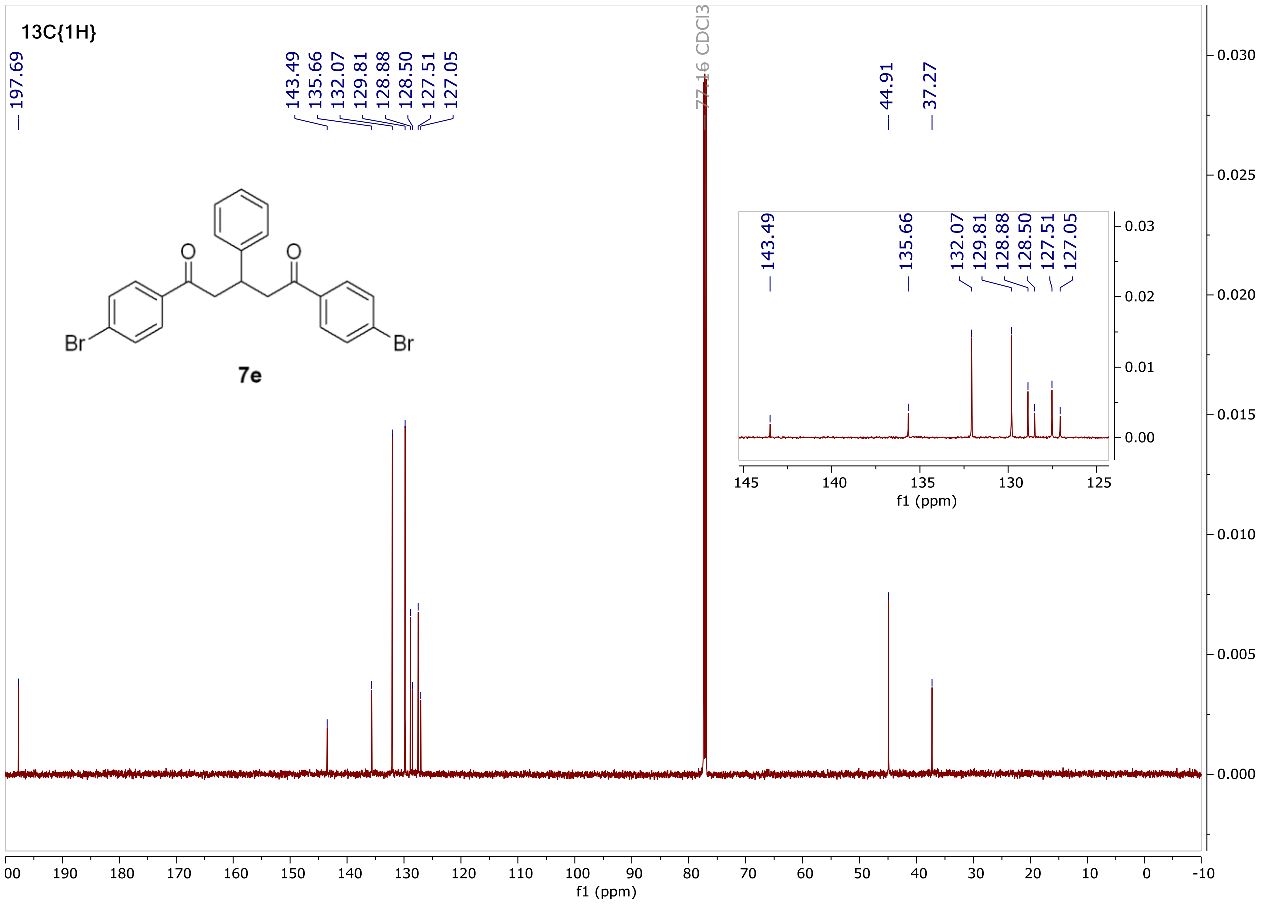

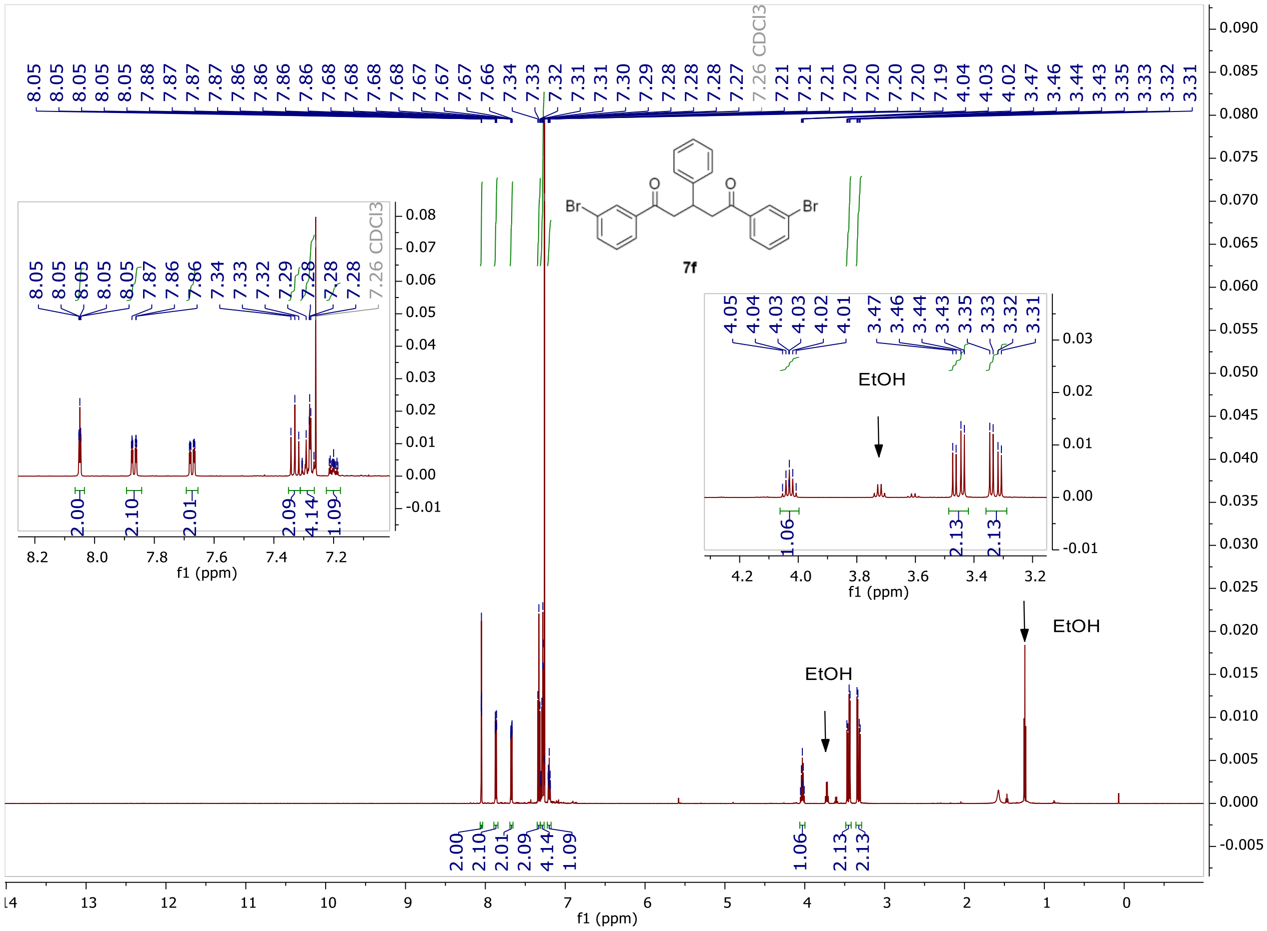
8.05 8.05 8.05 8.05 8.05 7.88 7.87 7.87 7.87 7.86 7.86 7.86 7.68 7.68 7.68 7.67 7.67 7.67 7.66 7.34 7.33 7.32 7.31 7.31 7.30 7.29 7.28 7.28 7.28 7.27
7.26 CDCl3
7.21 7.21 7.21 7.20 7.20 7.20 7.20 7.19 4.04 4.03 4.02 3.47 3.46 3.44 3.43 3.35 3.33 3.32 3.31
7f
EtOH
EtOH
2.00 2.10 2.01 2.09 4.14 1.09 1.06 2.13 2.13
f1 (ppm)

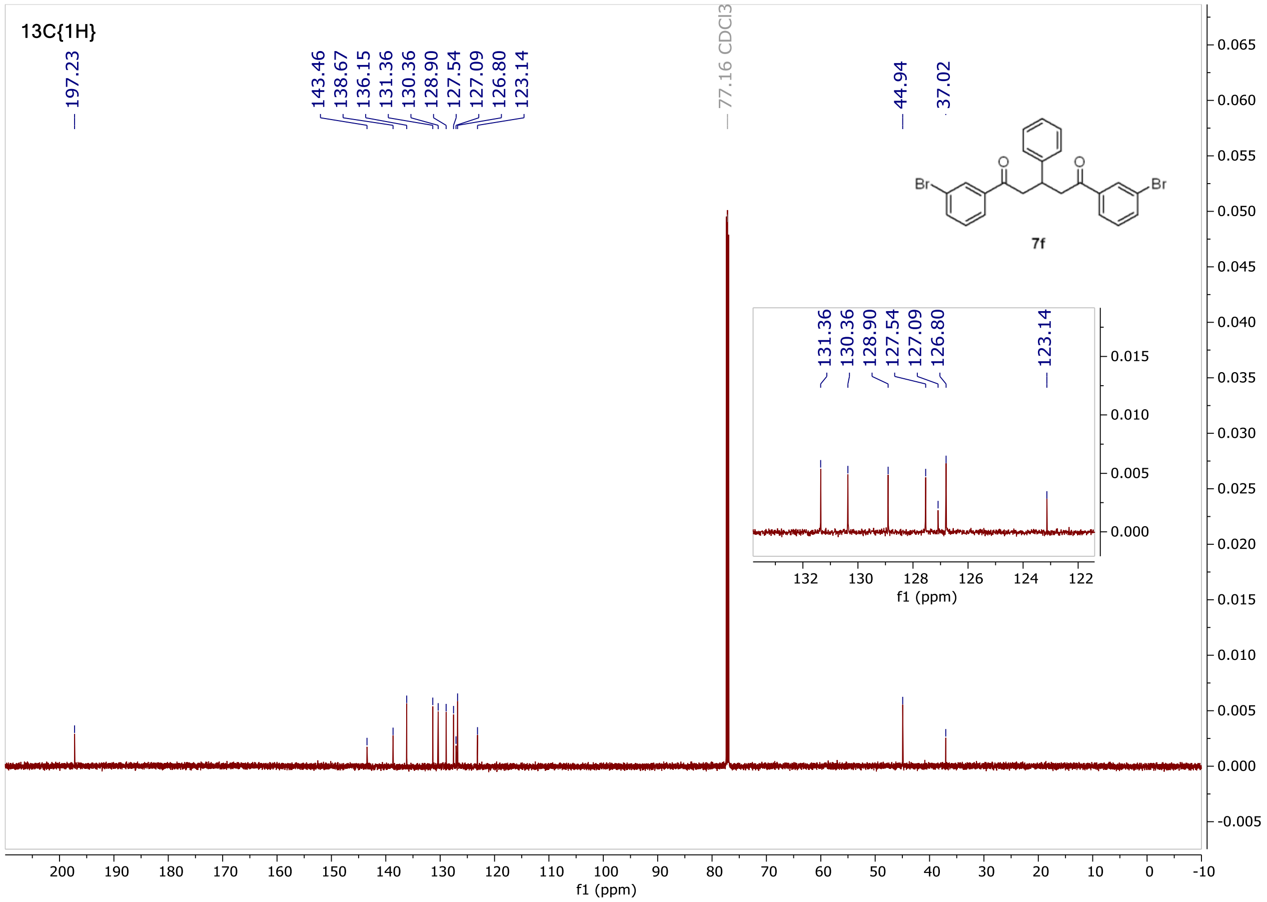
13C{1H}
197.23
143.46 138.67 136.15 131.36 130.36 128.90 127.54 127.09 126.80 123.14
77.16 CDCl3
44.94 37.02
7f
f1 (ppm)

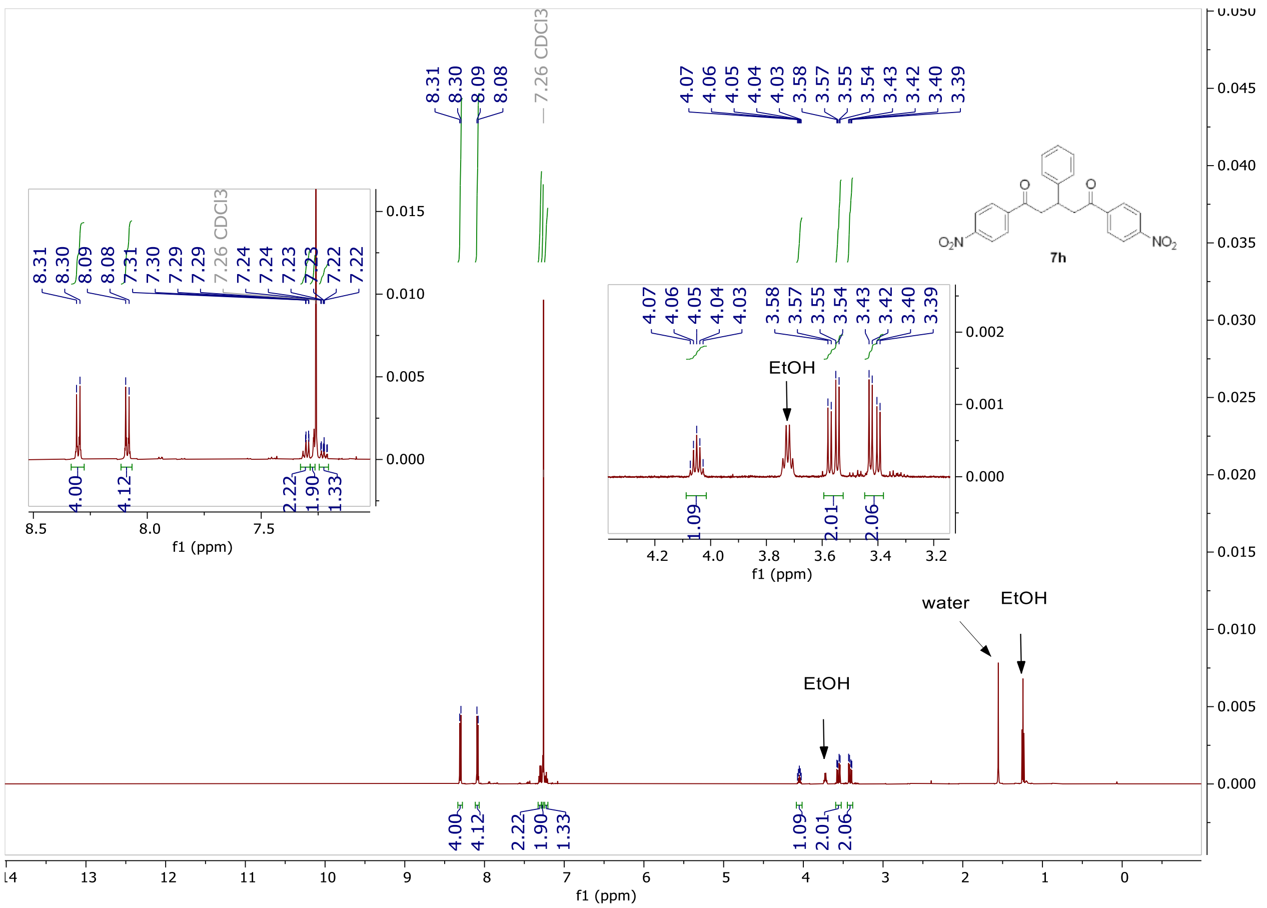
8.31
8.30
8.09
8.08
7.26 CDCl3
4.07
4.06
4.05
4.04
4.03
3.58
3.57
3.55
3.54
3.43
3.42
3.40
3.39
7.31
7.30
7.29
7.29
7.24
7.24
7.23
7.23
7.22
7.22
EtOH
water
7h
4.00
4.12
2.22
1.90
1.33
1.09
2.01
2.06
f1 (ppm)

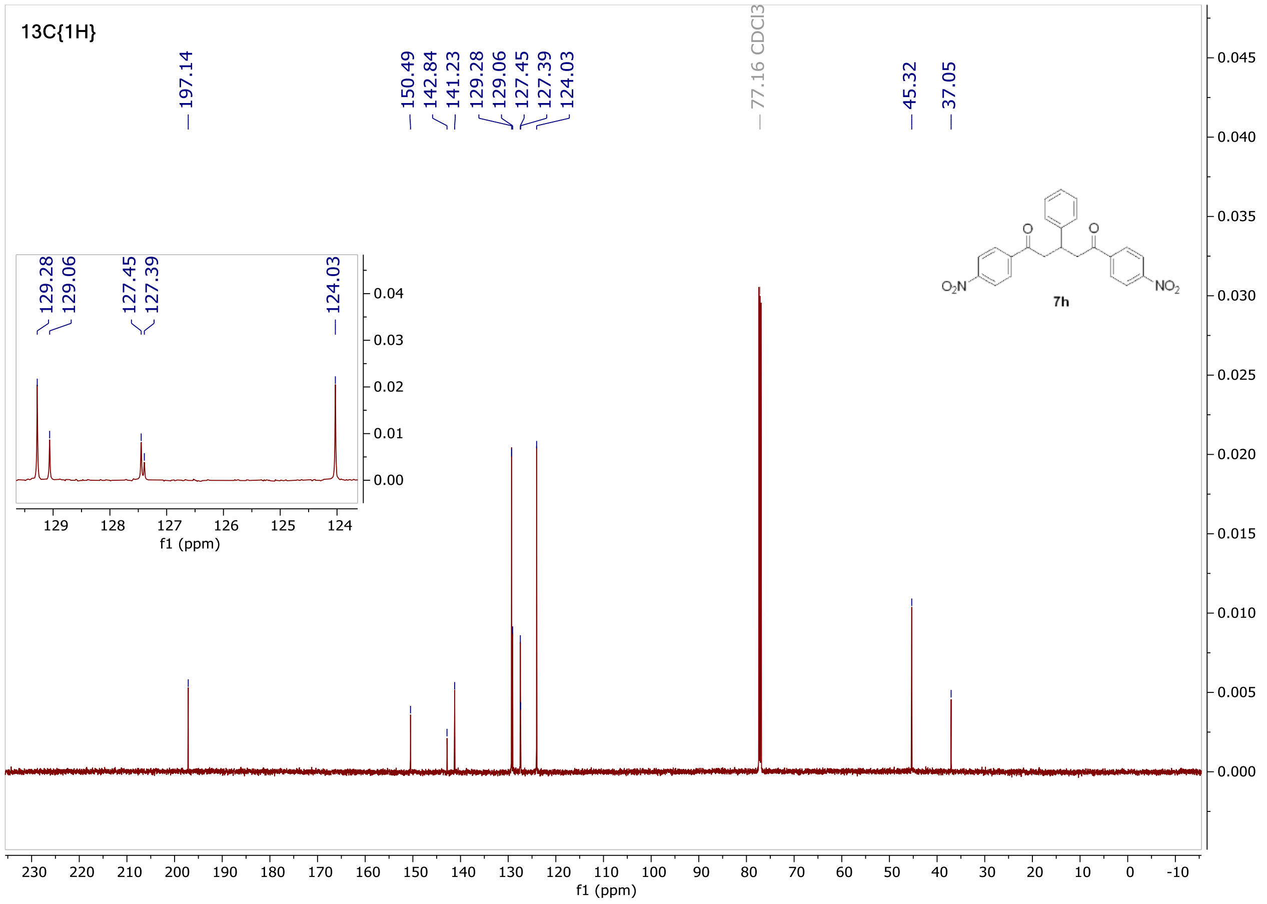
13C{1H}
197.14
150.49
142.84
141.23
129.28
129.06
127.45
127.39
124.03
77.16 CDCl3
45.32
37.05
7h
f1 (ppm)

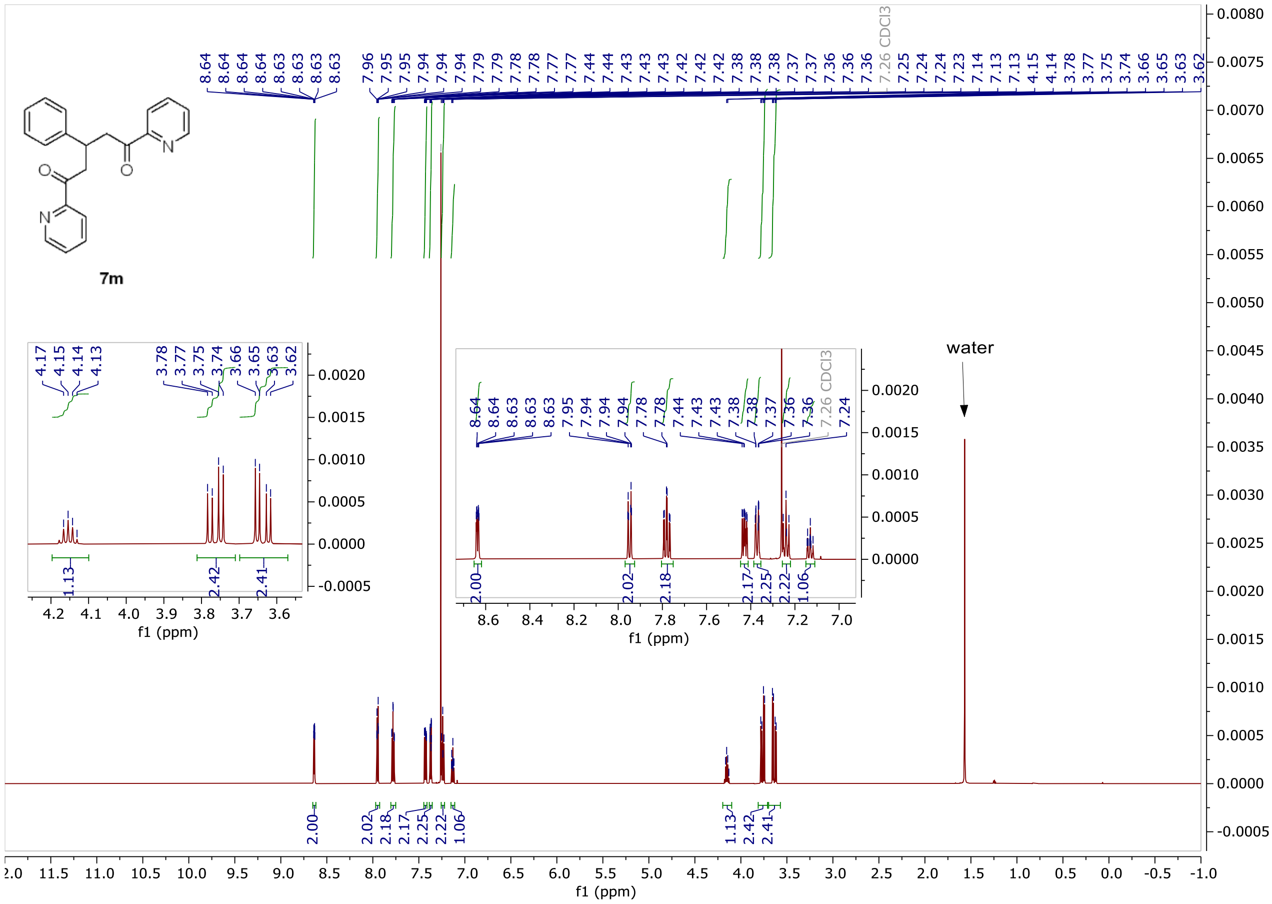

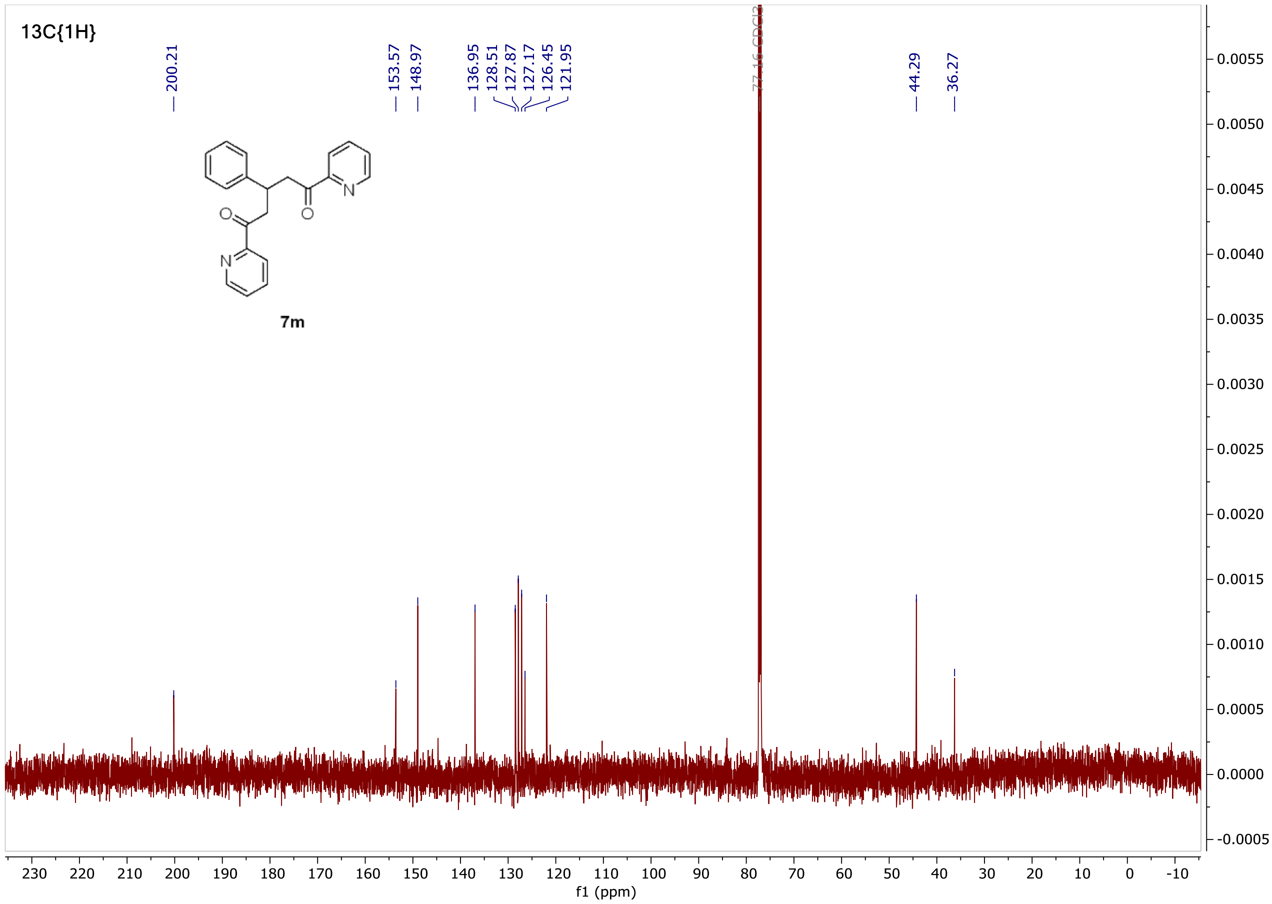

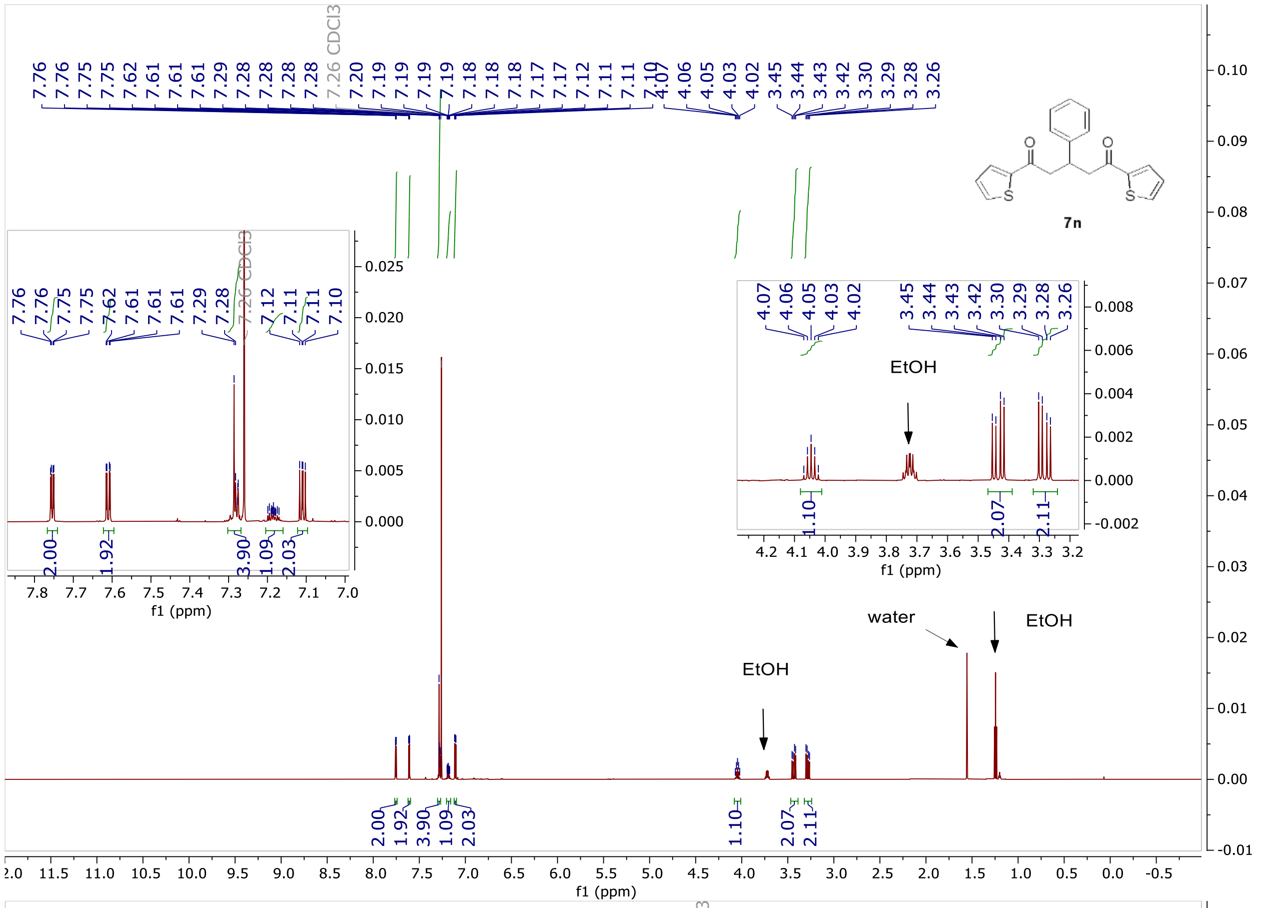
7.76
7.76
7.75
7.75
7.62
7.61
7.61
7.61
7.29
7.28
7.28
7.28
7.28
7.26 CDCl3
7.20
7.19
7.19
7.19
7.19
7.18
7.18
7.18
7.17
7.12
7.11
7.11
7.10
4.07
4.06
4.05
4.03
4.02
3.45
3.44
3.43
3.42
3.30
3.29
3.28
3.26
7n
EtOH
water
EtOH
EtOH
2.00
1.92
3.90
1.09
2.03
1.10
2.07
2.11
f1 (ppm)

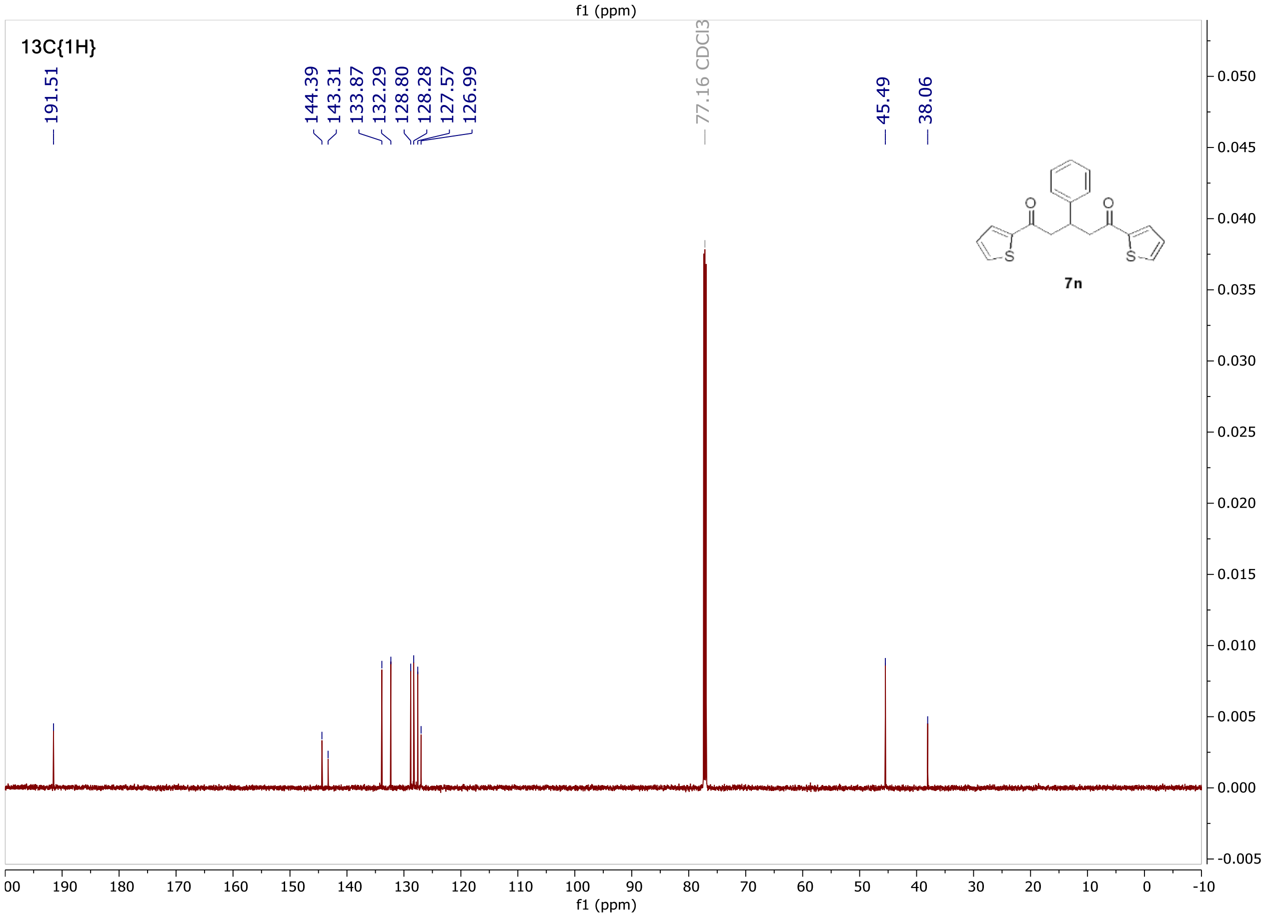
13C{1H}
191.51
144.39
143.31
133.87
132.29
128.80
128.28
127.57
126.99
77.16 CDCl3
45.49
38.06
7n
f1 (ppm)

^{1}H and ^{13}C{1H} spectra of compounds 8-17

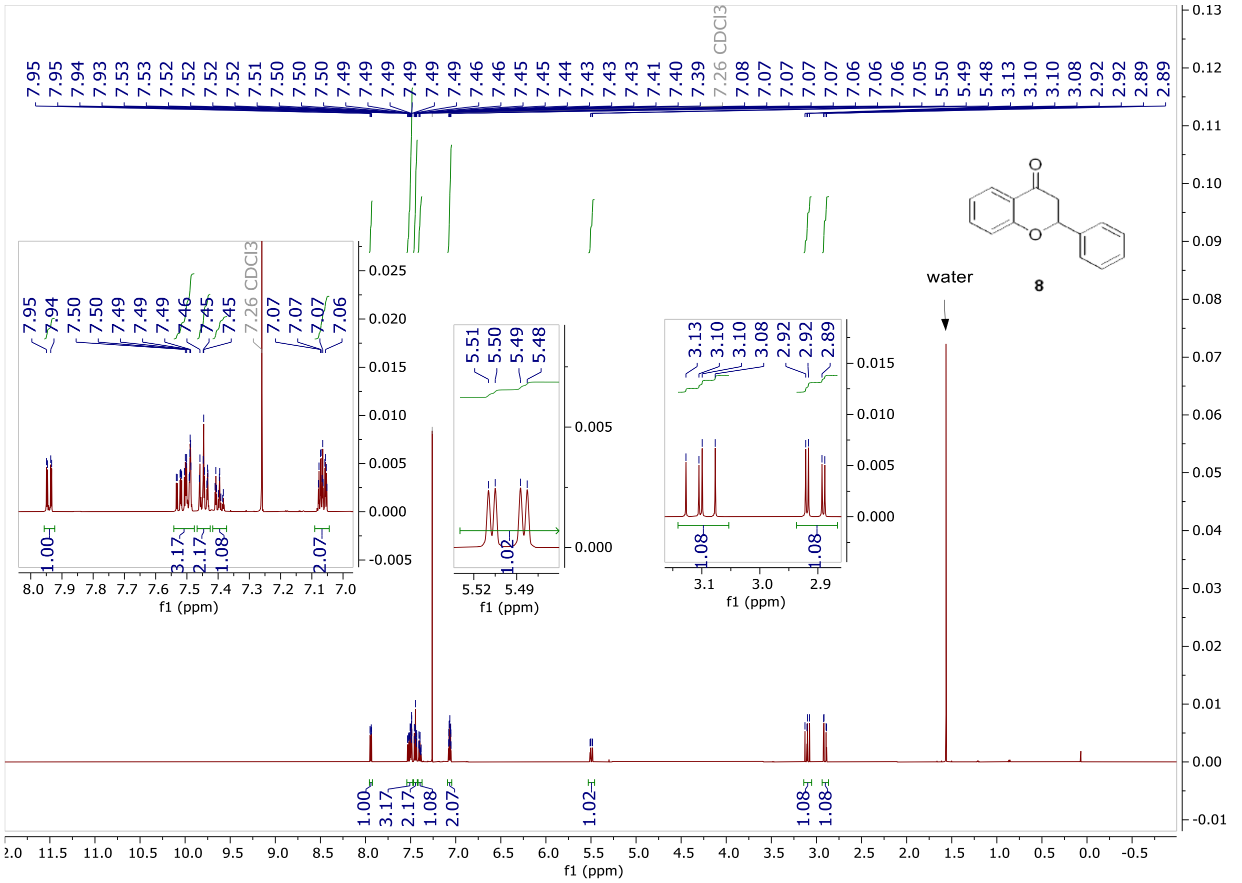

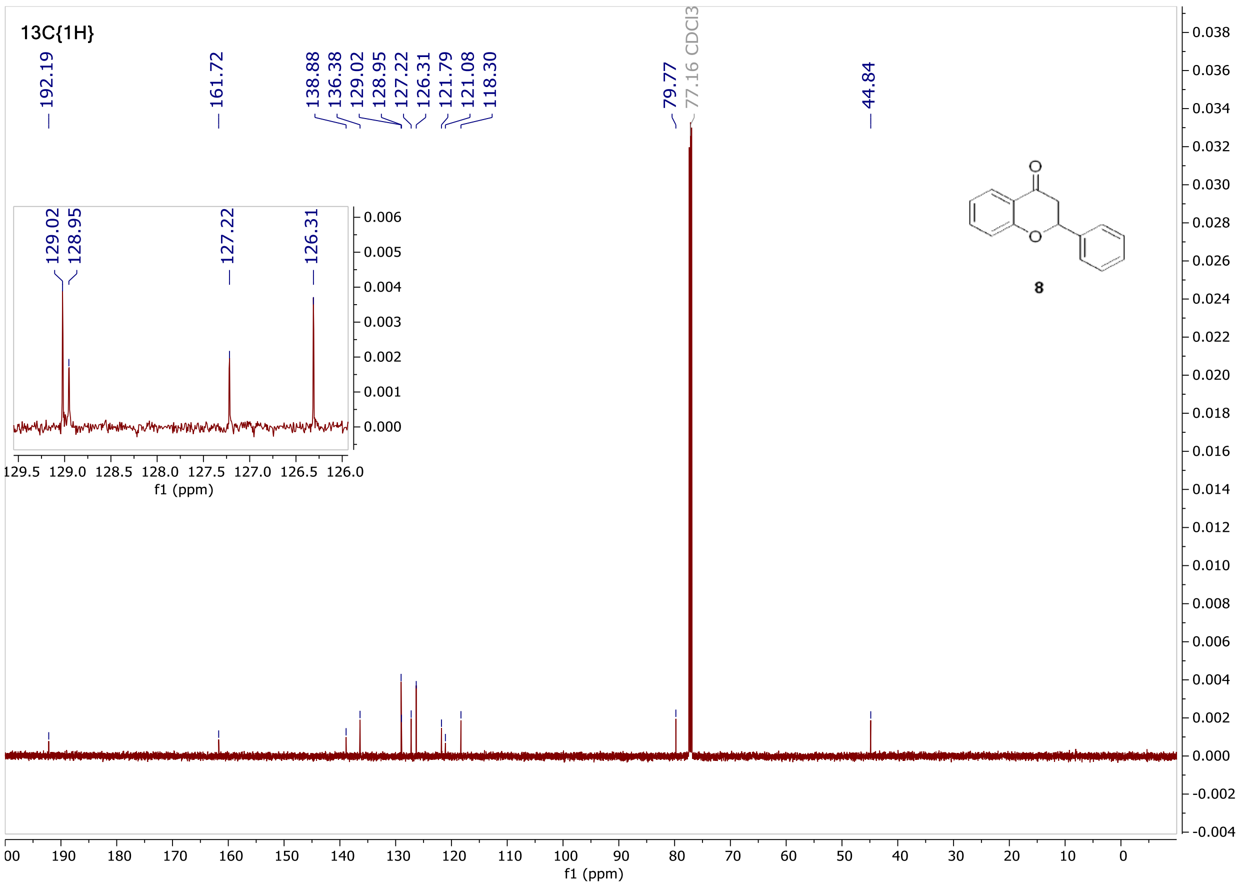

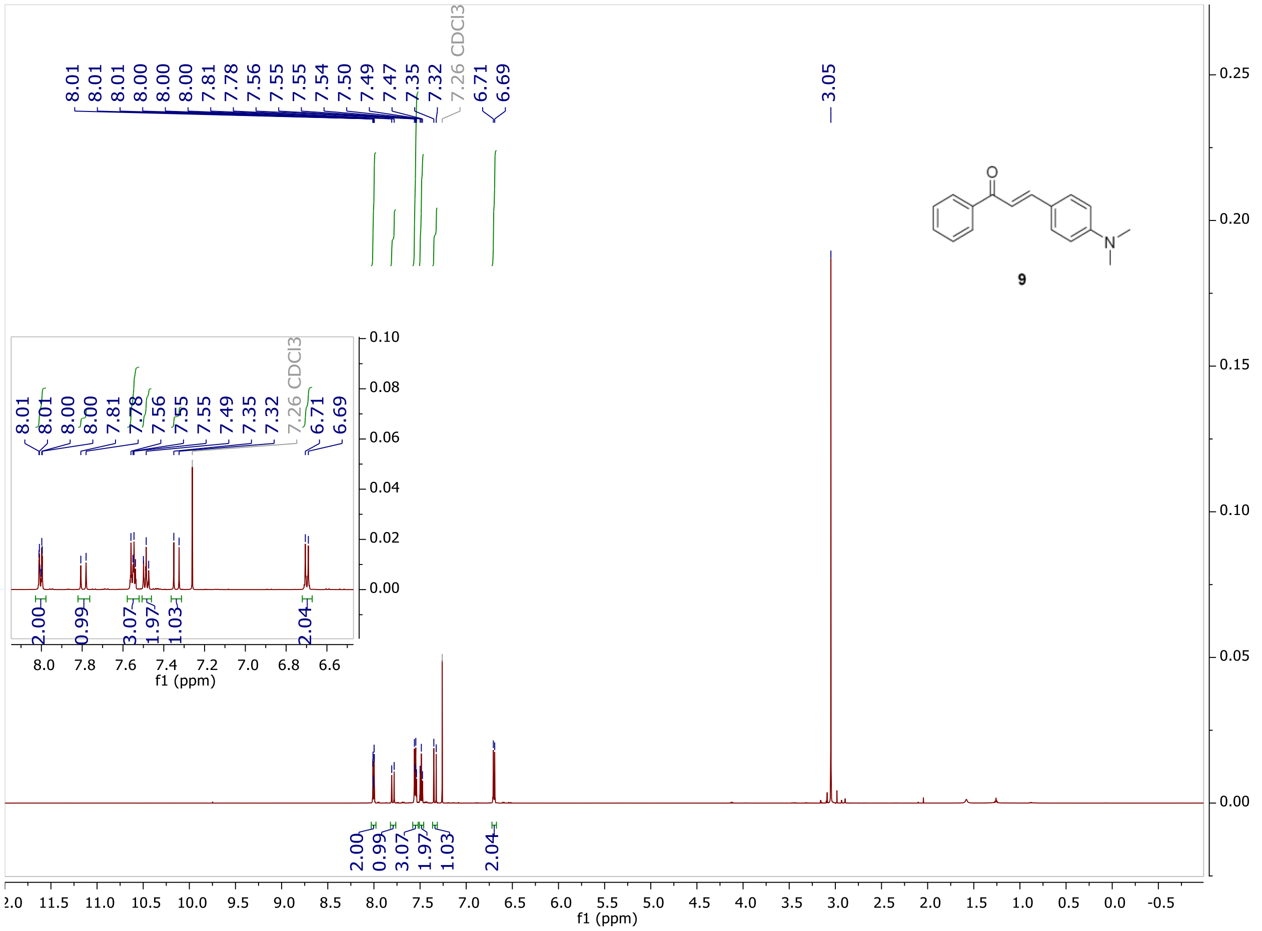

8.01
8.01
8.01
8.00
8.00
8.00
7.81
7.78
7.56
7.55
7.55
7.54
7.50
7.49
7.47
7.35
7.32
7.26 CDCl3
6.71
6.69
3.05
9
2.00
0.99
3.07
1.97
1.03
2.04
f1 (ppm)

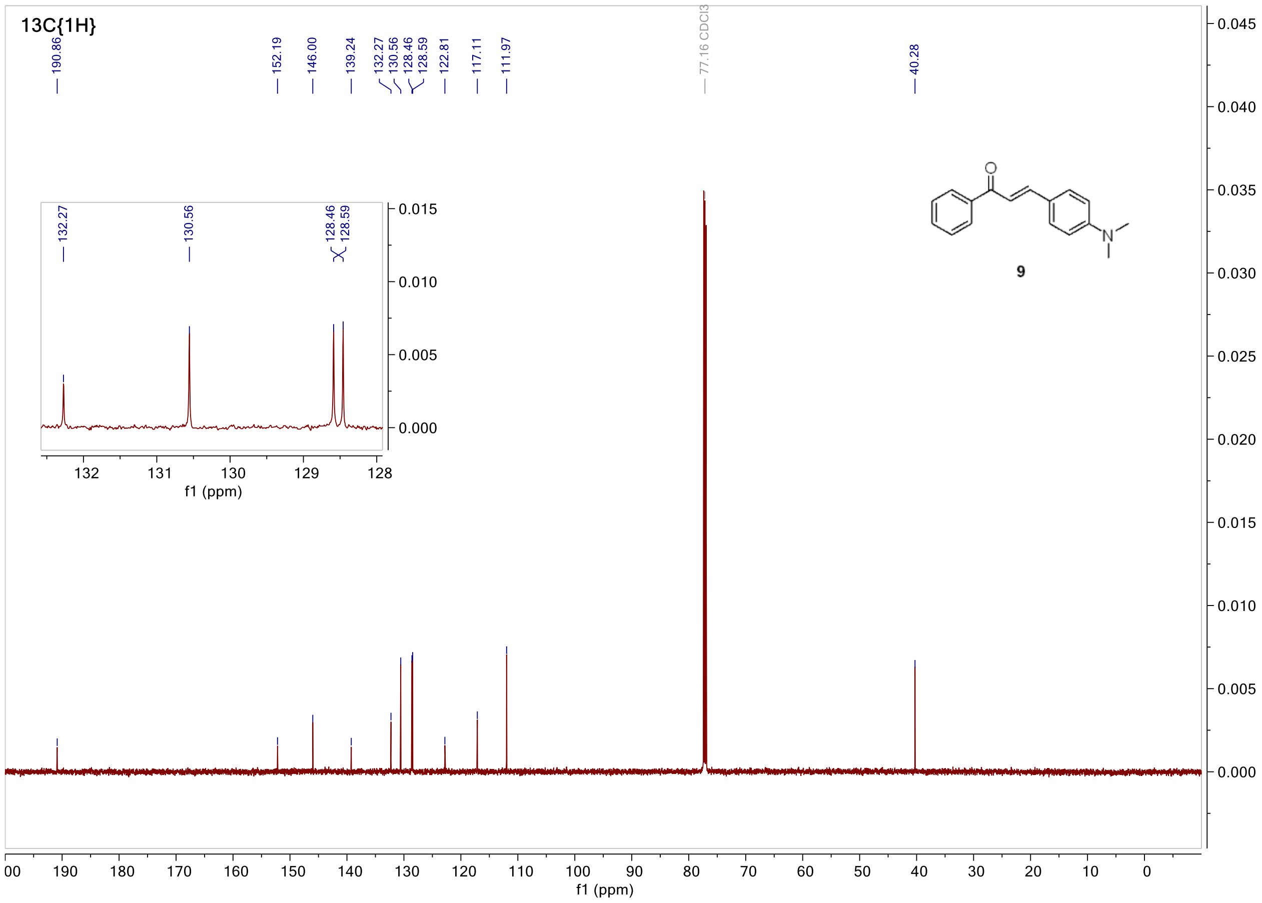

13C{1H}
190.86
152.19
146.00
139.24
132.27
130.56
128.46
128.59
122.81
117.11
111.97
77.16 CDCl3
40.28
9
f1 (ppm)

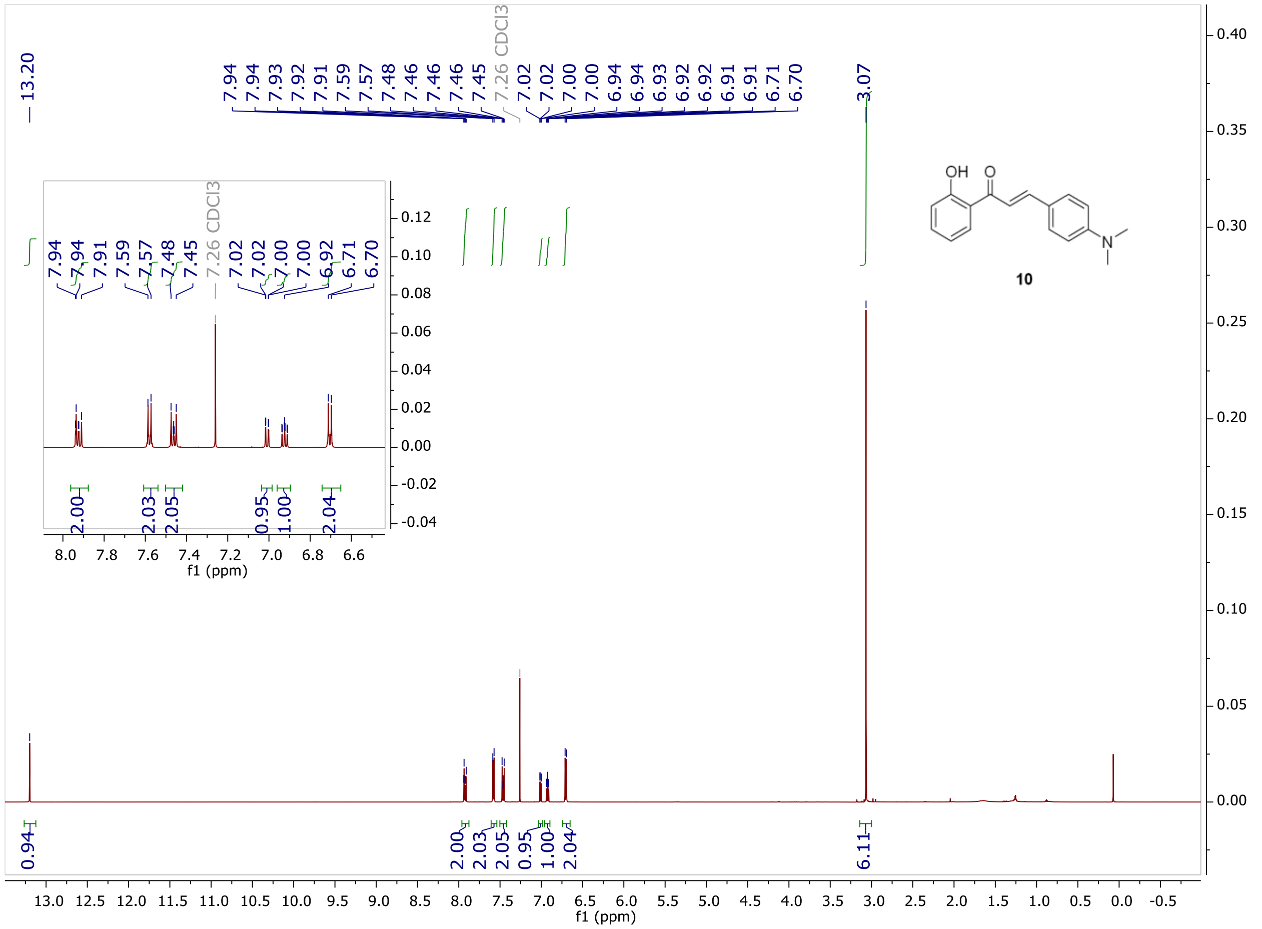
13.20
7.94 7.94 7.93 7.92 7.91 7.59 7.57 7.48 7.46 7.46 7.46 7.45 7.26 CDCl3 7.02 7.02 7.00 7.00 6.94 6.94 6.93 6.92 6.92 6.92 6.91 6.91 6.71 6.70
3.07
OH
O
N
10
0.94
2.00 2.03 2.05 0.95 1.00 2.04
6.11
f1 (ppm)

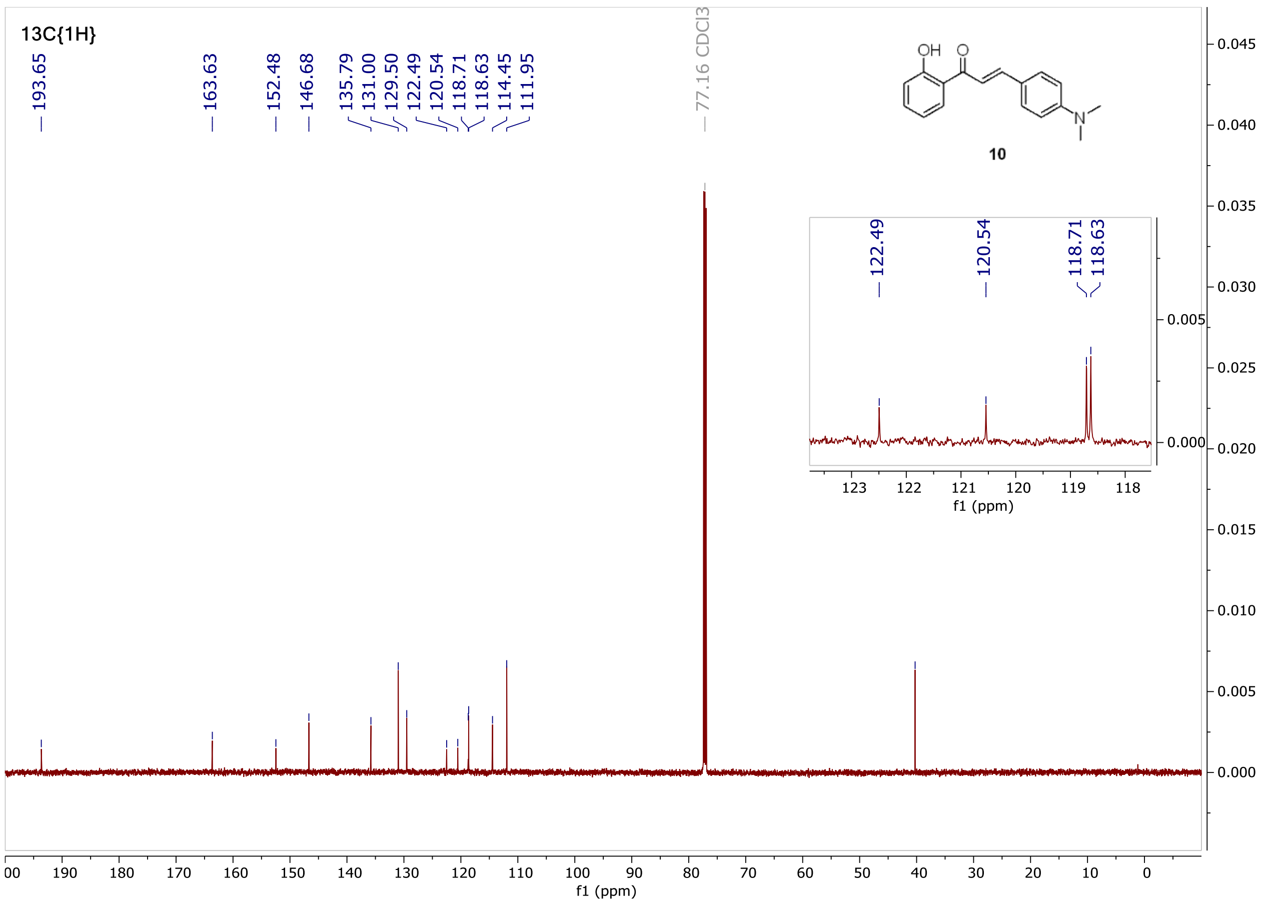
13C{1H}
193.65
163.63
152.48
146.68
135.79 131.00 129.50 122.49 120.54 118.71 118.63 114.45 111.95
77.16 CDCl3
OH
O
N
10
122.49
120.54
118.71 118.63
f1 (ppm)

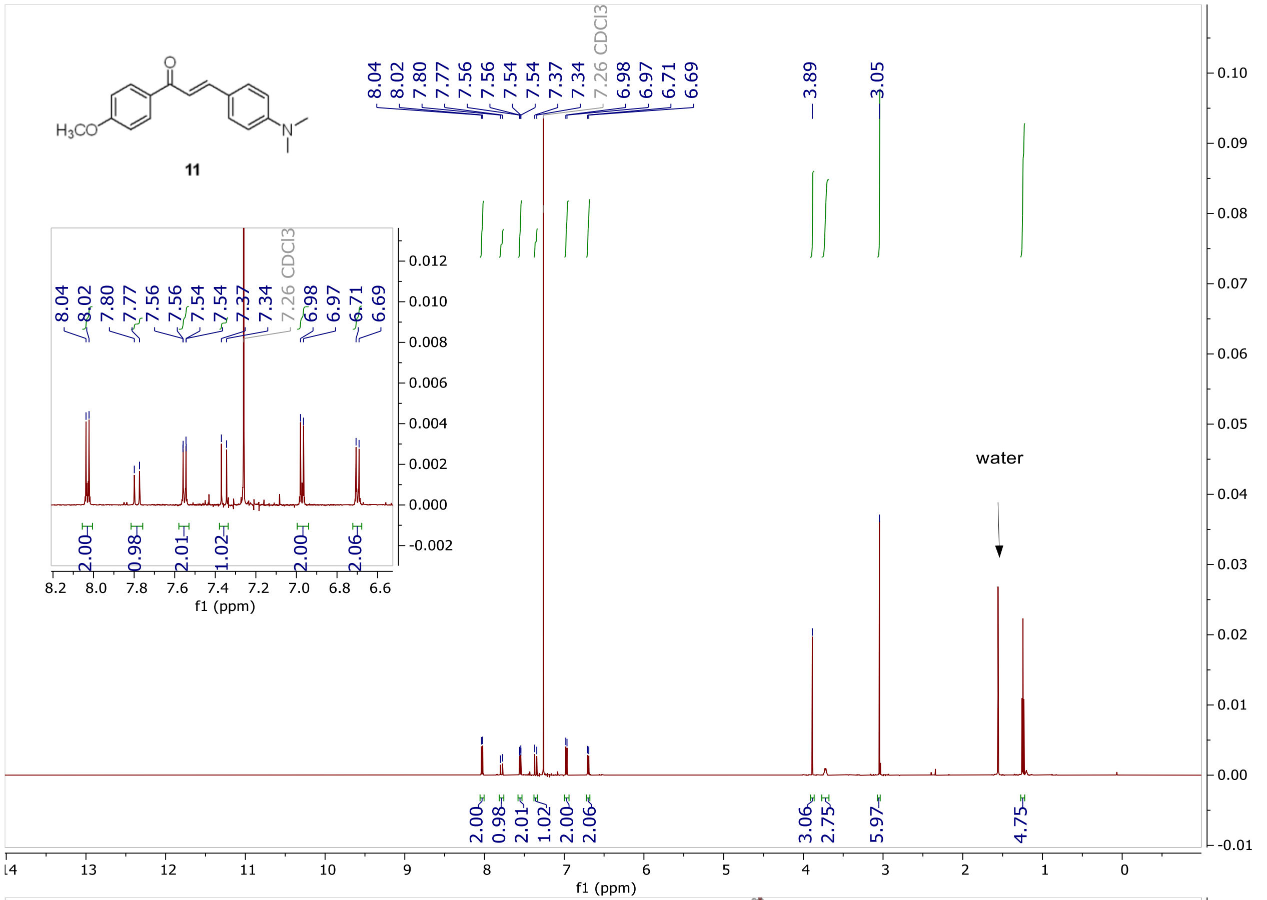

H3CO
11
8.04 8.02 7.80 7.77 7.56 7.56 7.54 7.54 7.37 7.34 7.26 CDCl3 6.98 6.97 6.71 6.69
3.89
3.05
water
2.00 0.98 2.01 1.02 2.00 2.06
3.06 2.75 5.97 4.75
f1 (ppm)

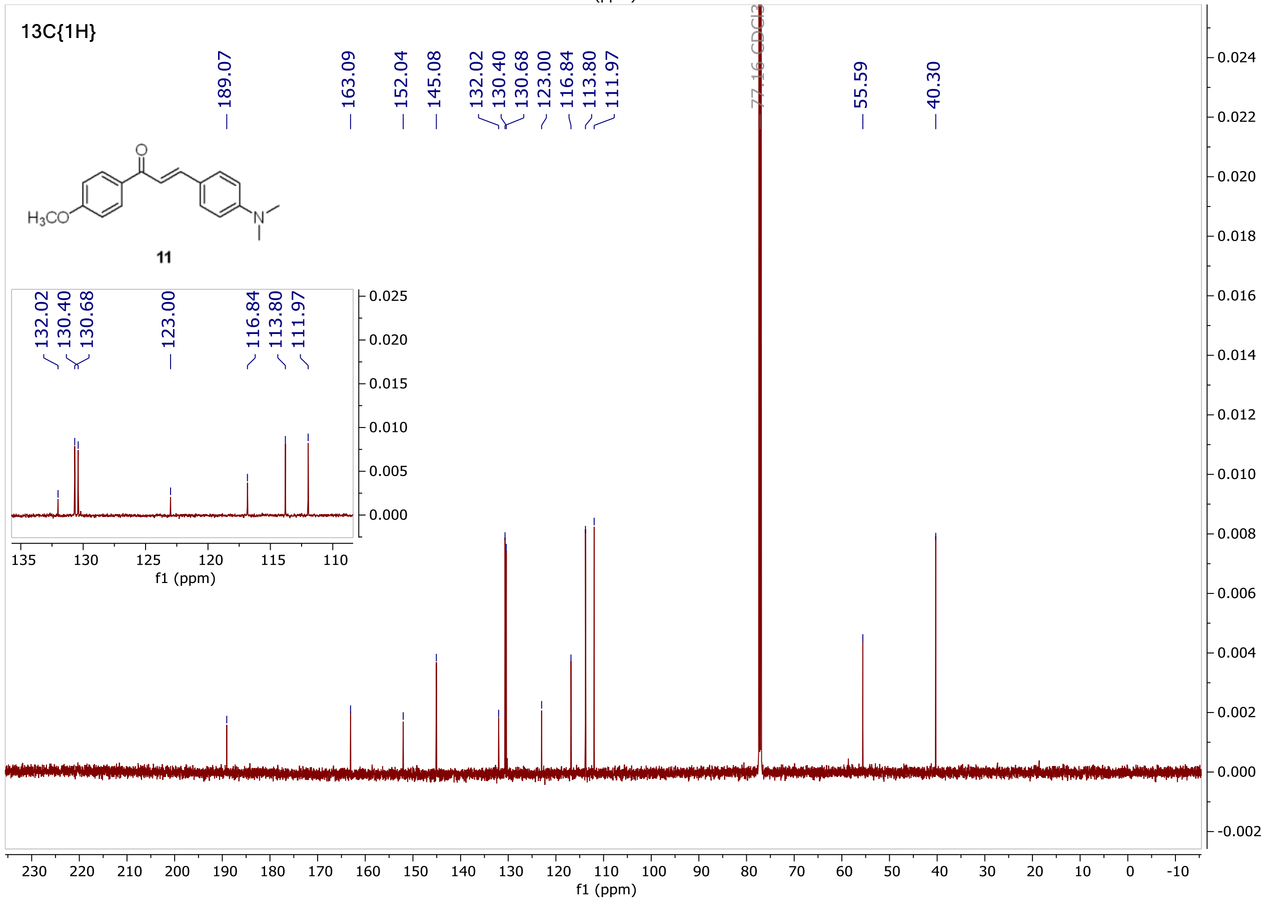

13C{1H}
189.07
163.09
152.04
145.08
132.02 130.40 130.68 123.00 116.84 113.80 111.97
77.16 CDCl3
55.59
40.30
H3CO
11
f1 (ppm)

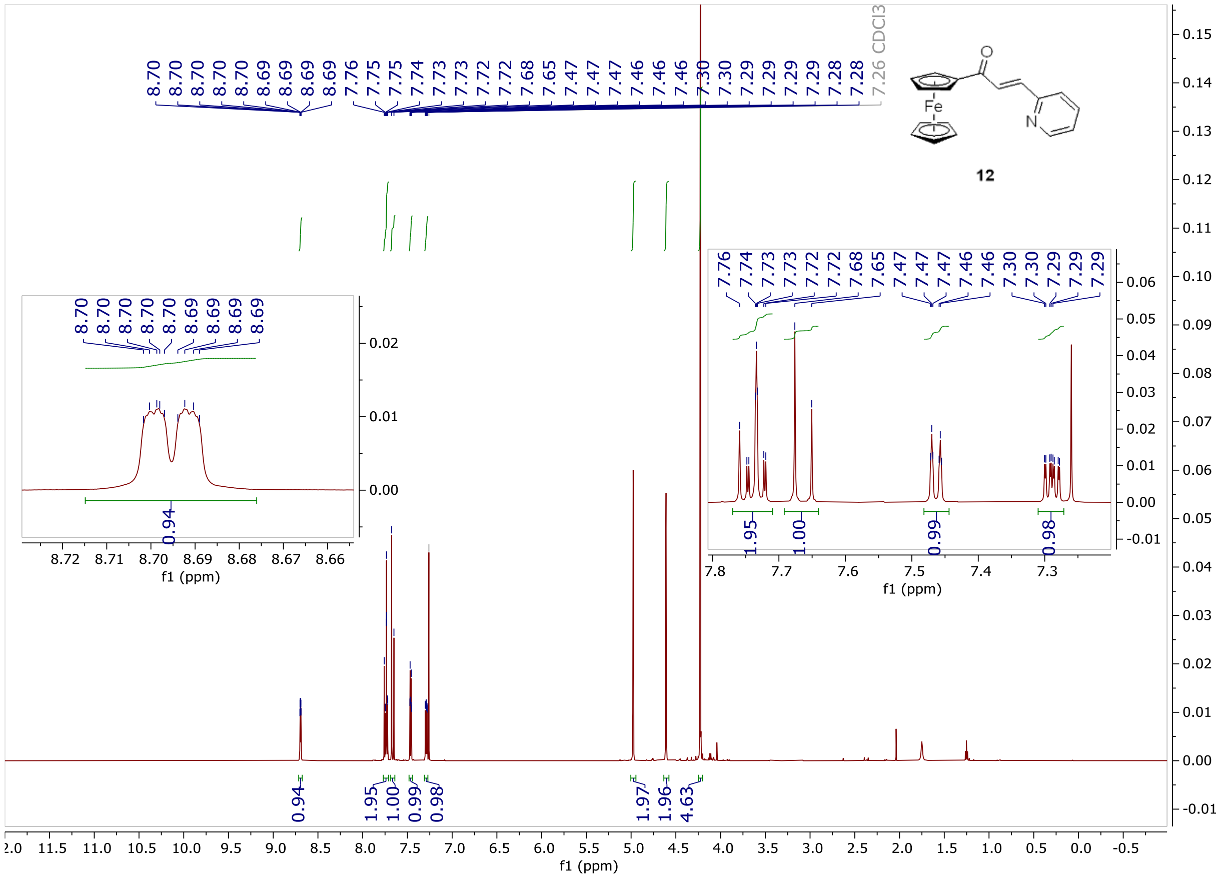
8.70 8.70 8.70 8.70 8.70 8.69 8.69 8.69 8.69 7.76 7.75 7.75 7.74 7.73 7.73 7.72 7.72 7.68 7.65 7.47 7.47 7.47 7.46 7.46 7.46 7.30 7.30 7.29 7.29 7.29 7.29 7.28 7.28
7.26 CDCl3
Fe
O
N
12
0.94 1.95 1.00 0.99 0.98 1.97 1.96 4.63
f1 (ppm)

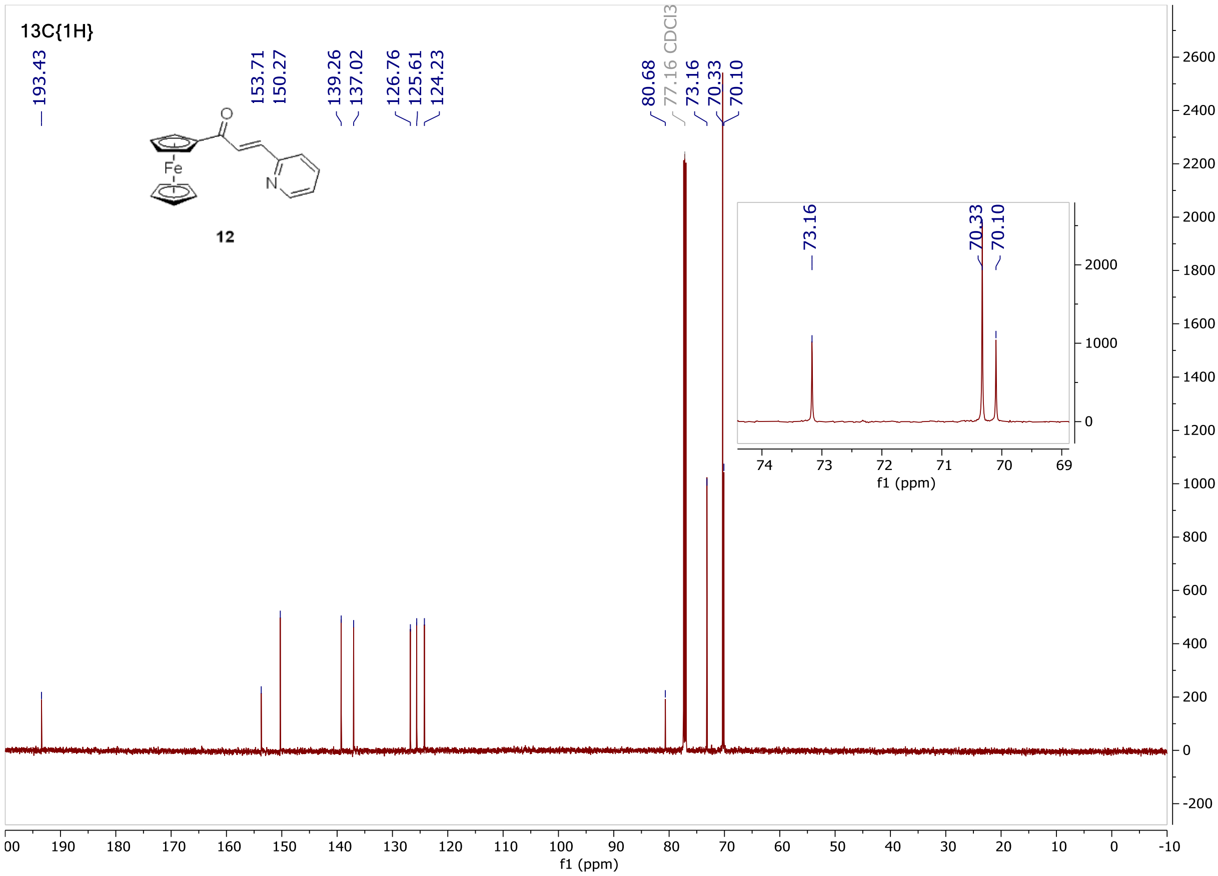
13C{1H}
193.43
153.71 150.27
139.26 137.02
126.76 125.61 124.23
80.68
77.16 CDCl3
73.16 70.33 70.10
Fe
O
N
12
f1 (ppm)

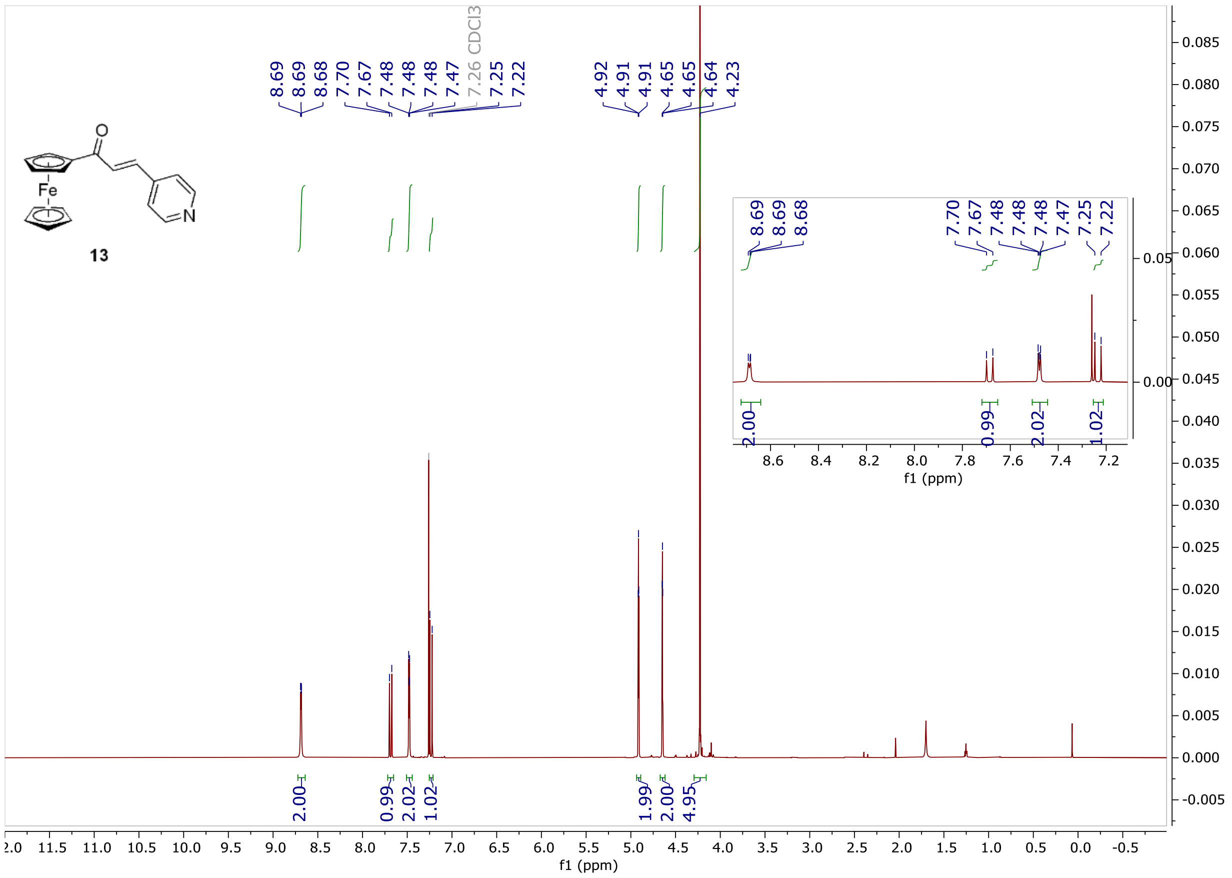
8.69 8.69 8.68 7.70 7.67 7.48 7.48 7.48 7.47 7.26 CDCl3 7.25 7.22
4.92 4.91 4.91 4.65 4.65 4.64 4.23
Fe
N
13
2.00
0.99
2.02
1.02
1.99
2.00
4.95
f1 (ppm)

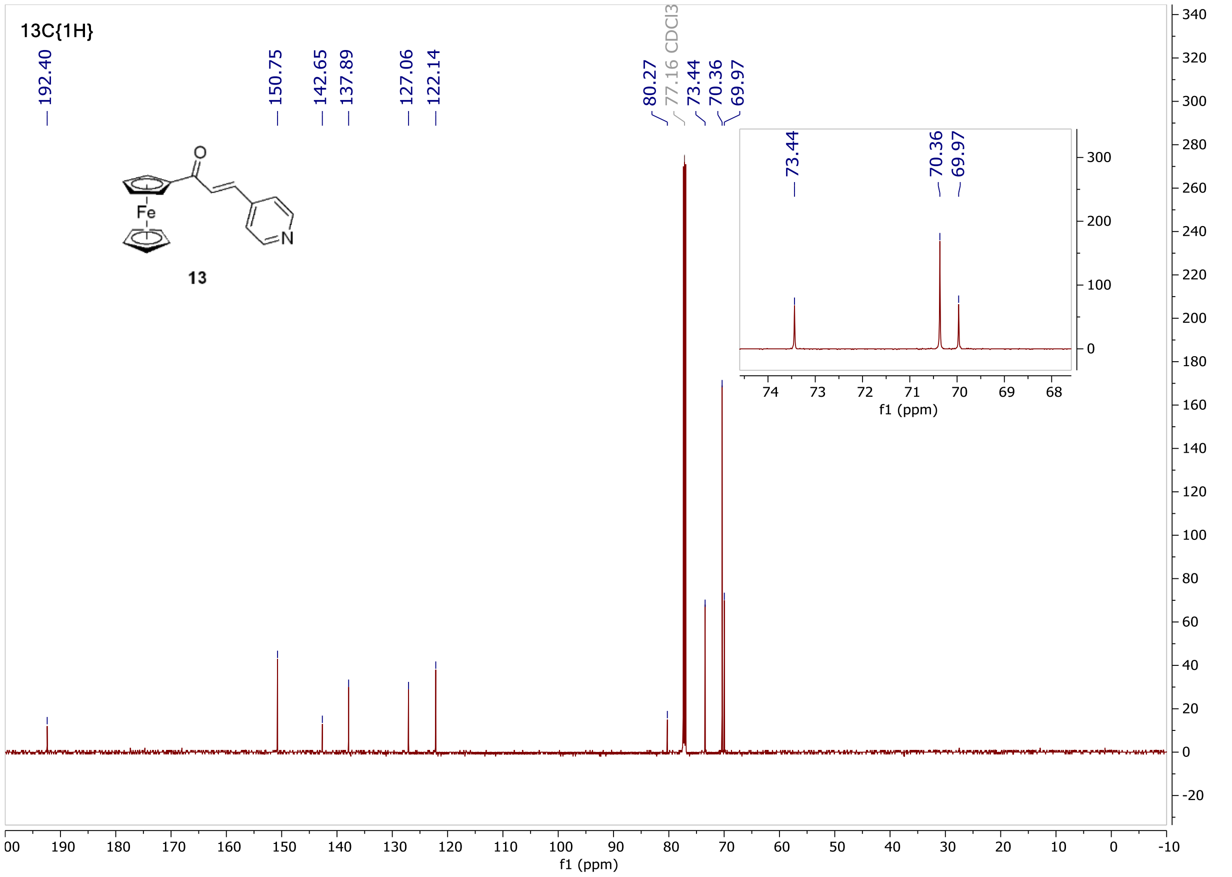
13C{1H}
192.40
150.75
142.65
137.89
127.06
122.14
80.27
77.16 CDCl3
73.44
70.36
69.97
Fe
N
13
f1 (ppm)

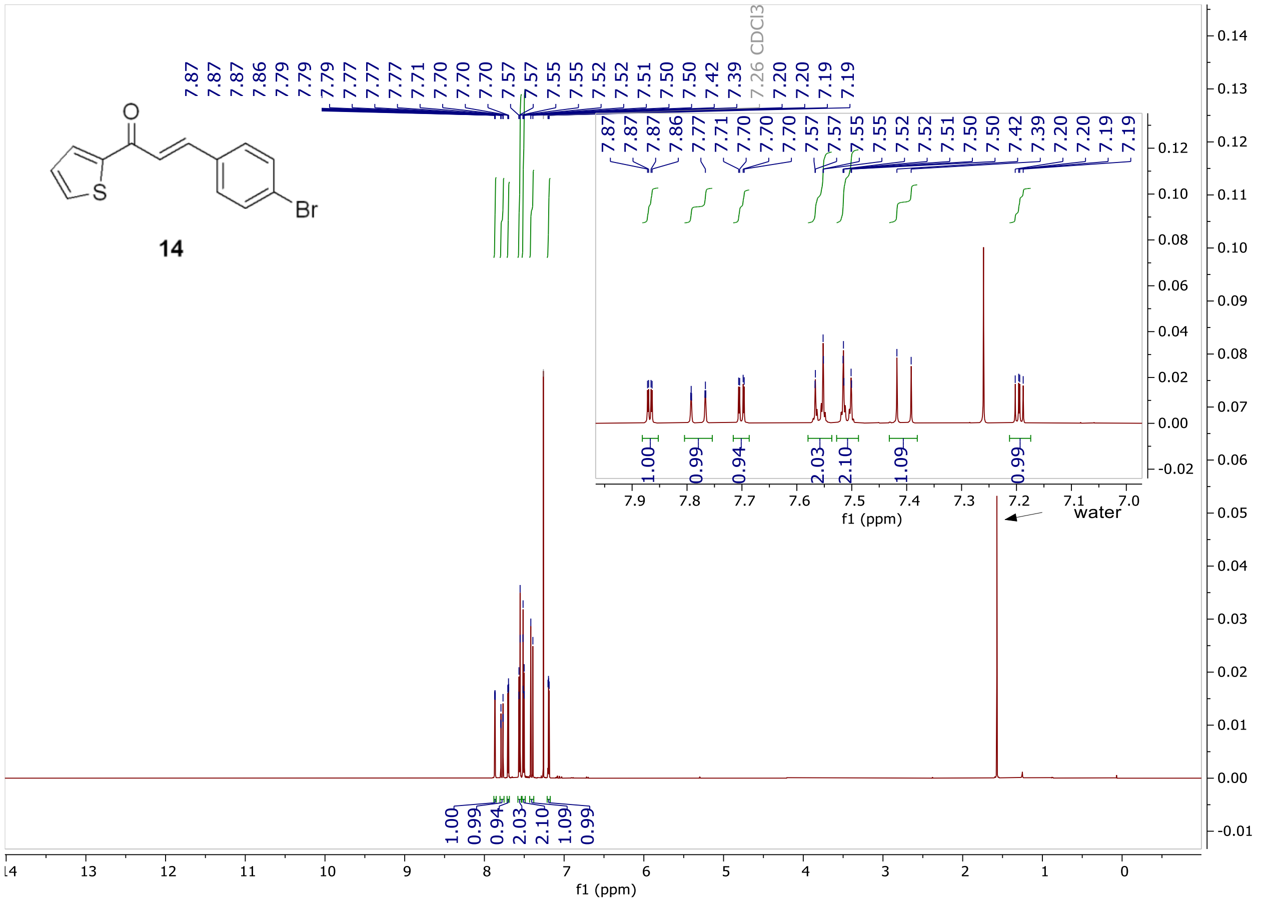
7.87 7.87 7.87 7.86 7.79 7.79 7.79 7.77 7.77 7.77 7.71 7.70 7.70 7.70 7.57 7.57 7.55 7.55 7.52 7.52 7.51 7.50 7.50 7.42 7.39 7.26 CDCl3 7.20 7.20 7.19 7.19
14
1.00 0.99 0.94 2.03 2.10 1.09 0.99
water
f1 (ppm)

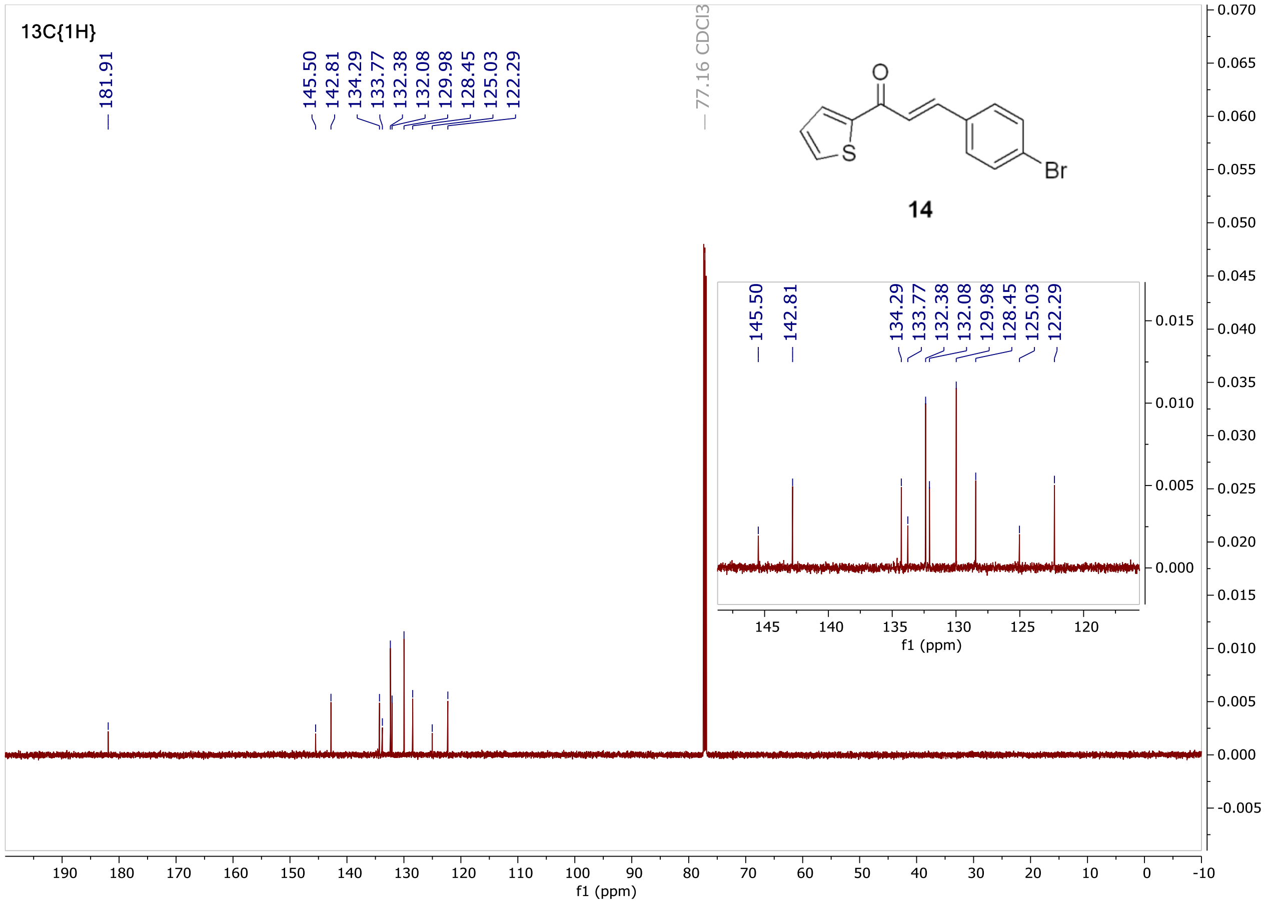
13C{1H}
181.91
145.50 142.81 134.29 133.77 132.38 132.08 129.98 128.45 125.03 122.29
77.16 CDCl3
14
f1 (ppm)

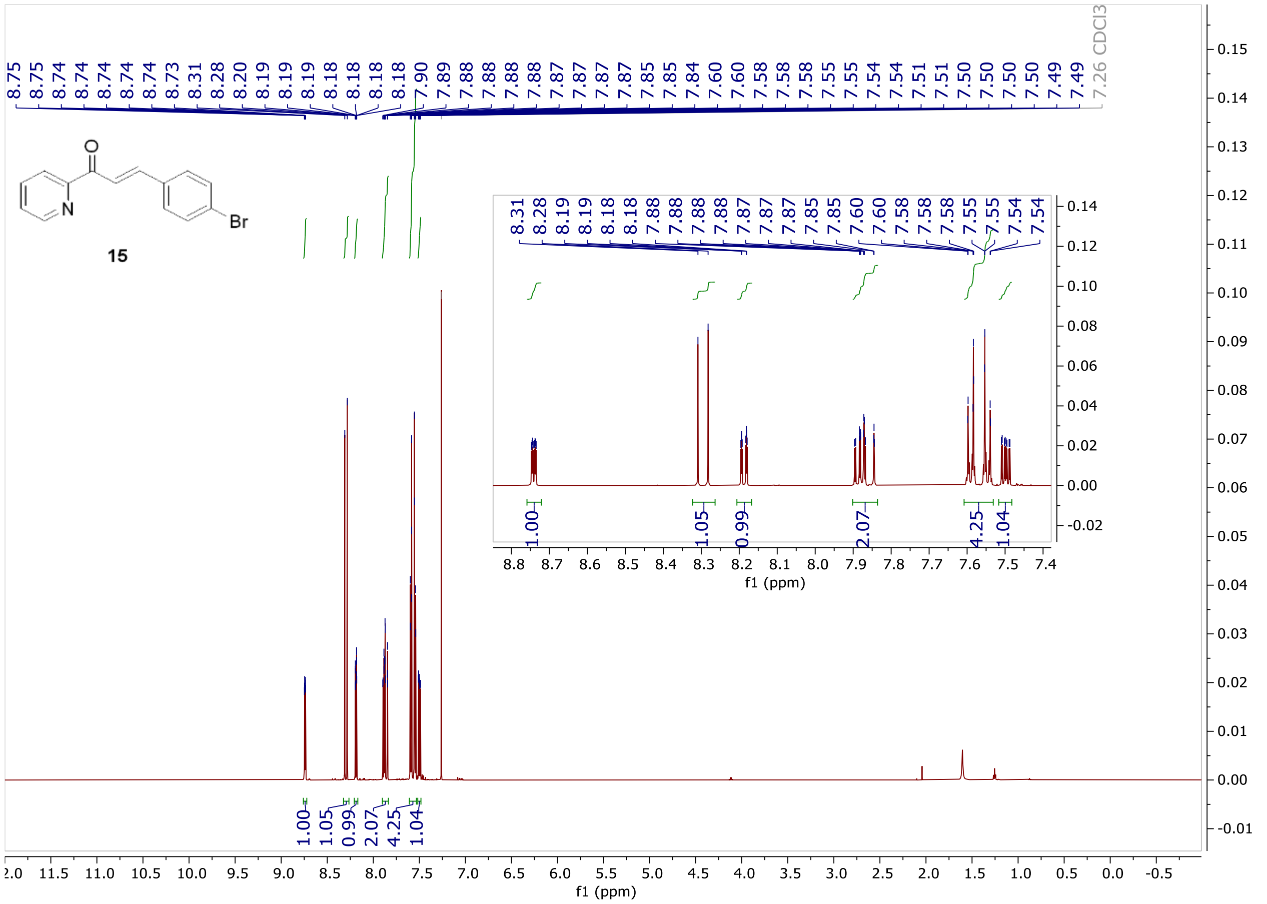
8.75 8.75 8.74 8.74 8.74 8.74 8.74 8.73 8.31 8.28 8.20 8.19 8.19 8.19 8.18 8.18 8.18 8.18 7.90 7.89 7.88 7.88 7.88 7.88 7.87 7.87 7.87 7.87 7.85 7.85 7.84 7.60 7.60 7.58 7.58 7.58 7.55 7.55 7.54 7.54 7.51 7.51 7.50 7.50 7.50 7.50 7.49 7.49
7.26 CDCl3
Br
N
O
15
1.00 1.05 0.99 2.07 4.25 1.04
f1 (ppm)

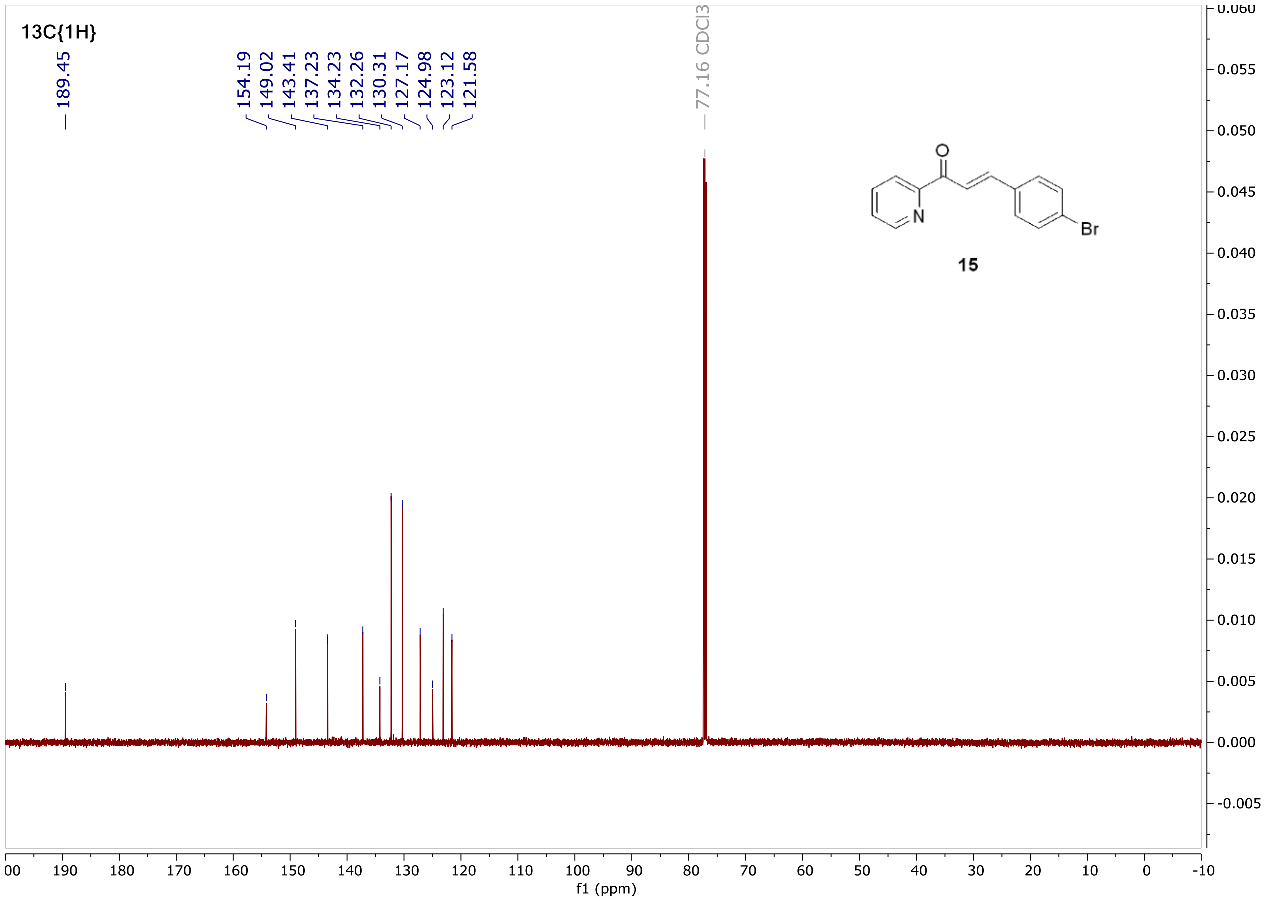
13C{1H}
189.45
154.19 149.02 143.41 137.23 134.23 132.26 130.31 127.17 124.98 123.12 121.58
77.16 CDCl3
O
N
Br
15
f1 (ppm)

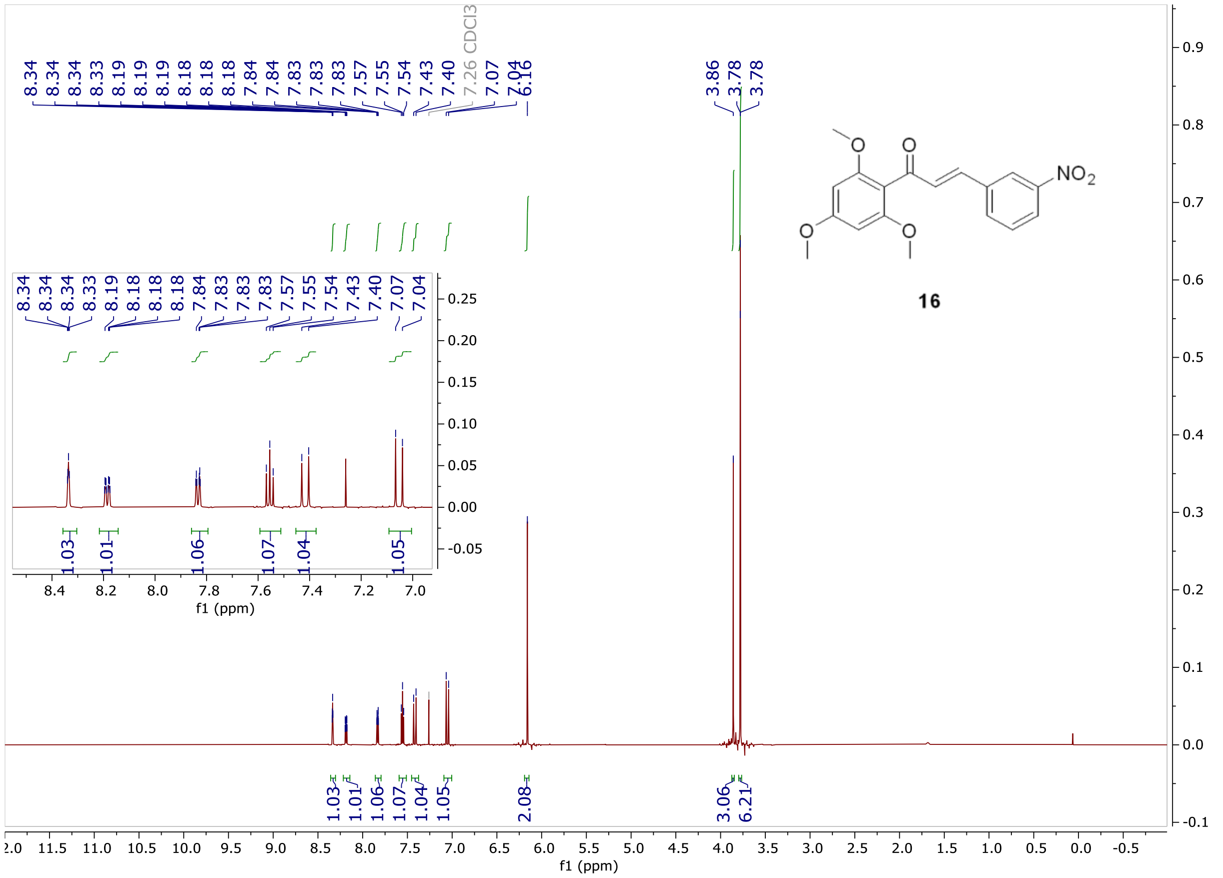
8.34
8.34
8.34
8.33
8.19
8.19
8.19
8.18
8.18
8.18
7.84
7.84
7.83
7.83
7.83
7.57
7.55
7.54
7.43
7.40
7.26 CDCl3
7.07
7.04
6.16
3.86
3.78
3.78
NO2
16
1.03
1.01
1.06
1.07
1.04
1.05
2.08
3.06
6.21
f1 (ppm)

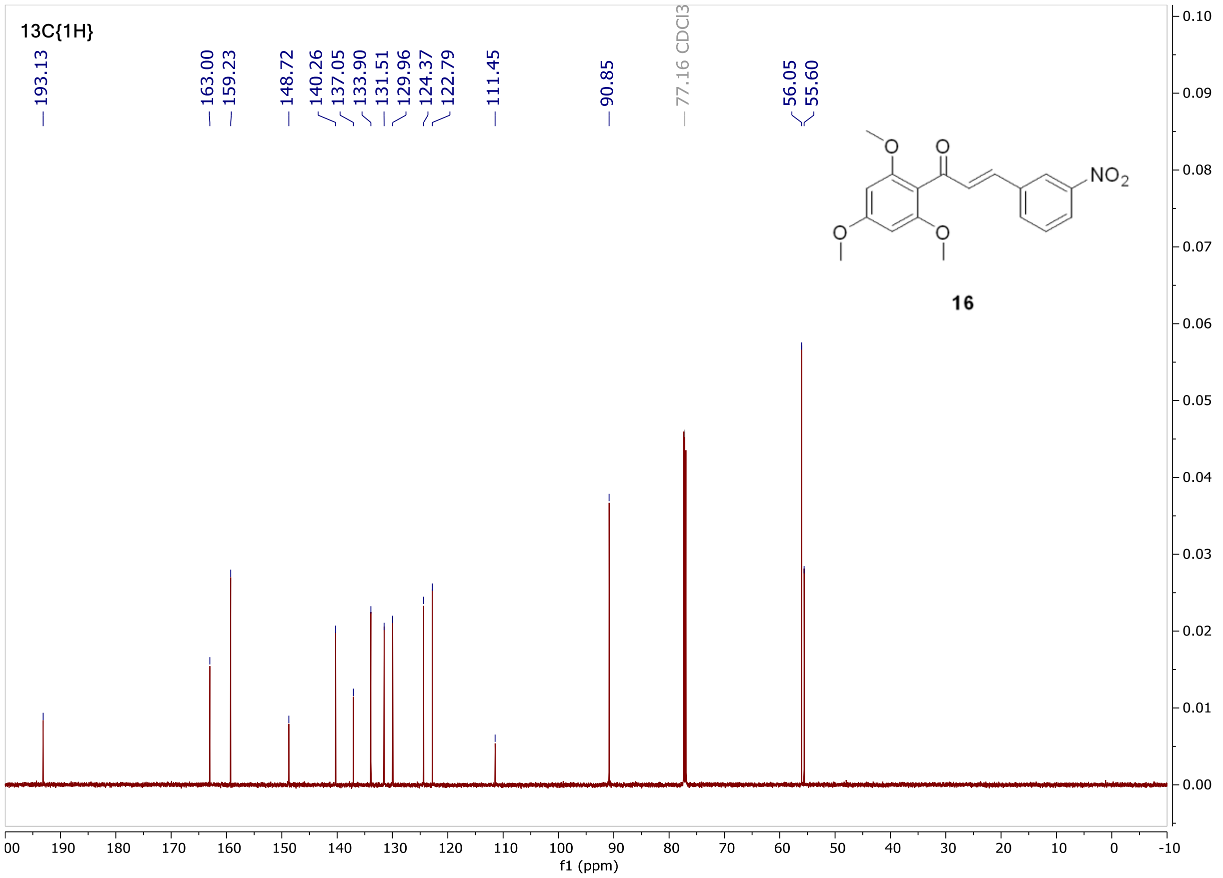
13C{1H}
193.13
163.00
159.23
148.72
140.26
137.05
133.90
131.51
129.96
124.37
122.79
111.45
90.85
77.16 CDCl3
56.05
55.60
NO2
16
f1 (ppm)

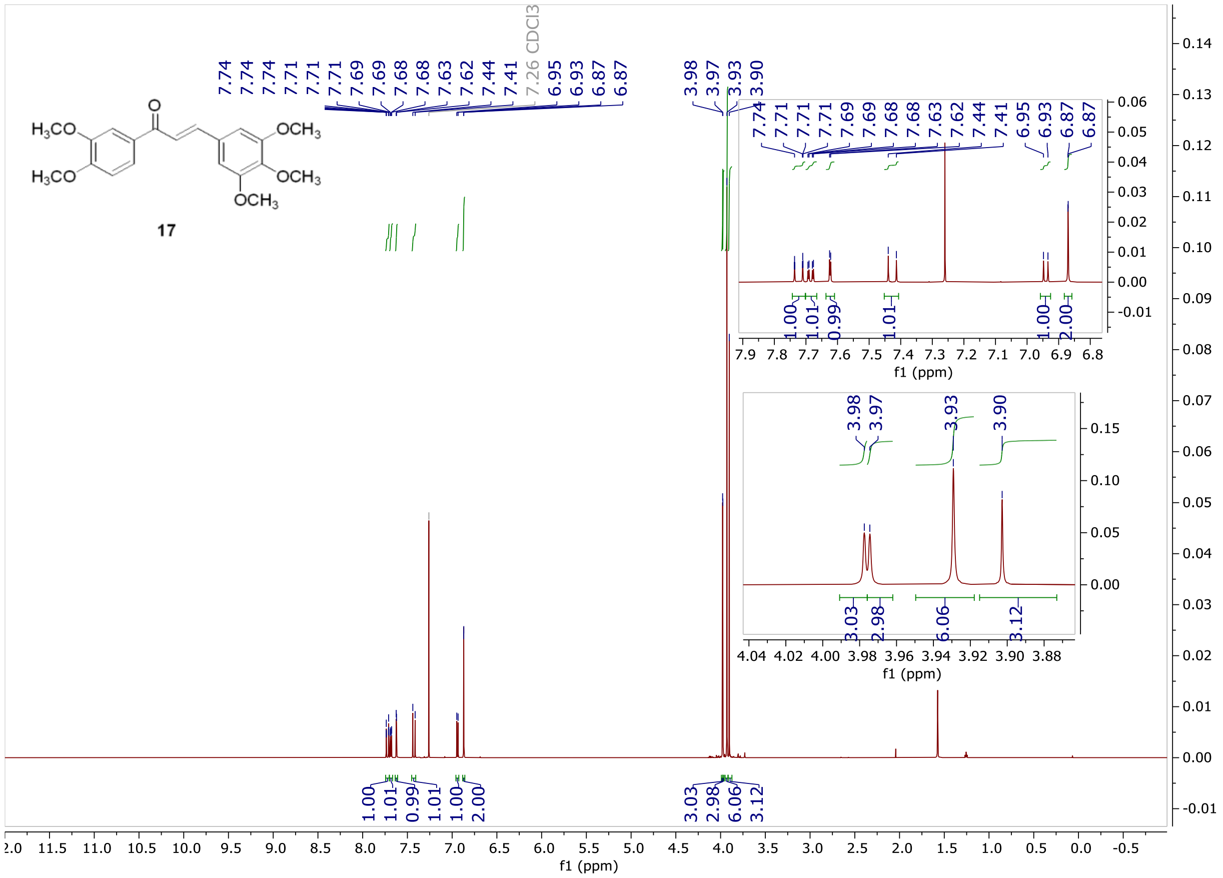
17
7.74 7.74 7.74 7.71 7.71 7.69 7.69 7.68 7.68 7.63 7.62 7.44 7.41 7.26 CDCl3 6.95 6.93 6.87 6.87
3.98 3.97 3.93 3.90
1.00 1.01 0.99 1.01 1.00 2.00
3.03 2.98 6.06 3.12
f1 (ppm)

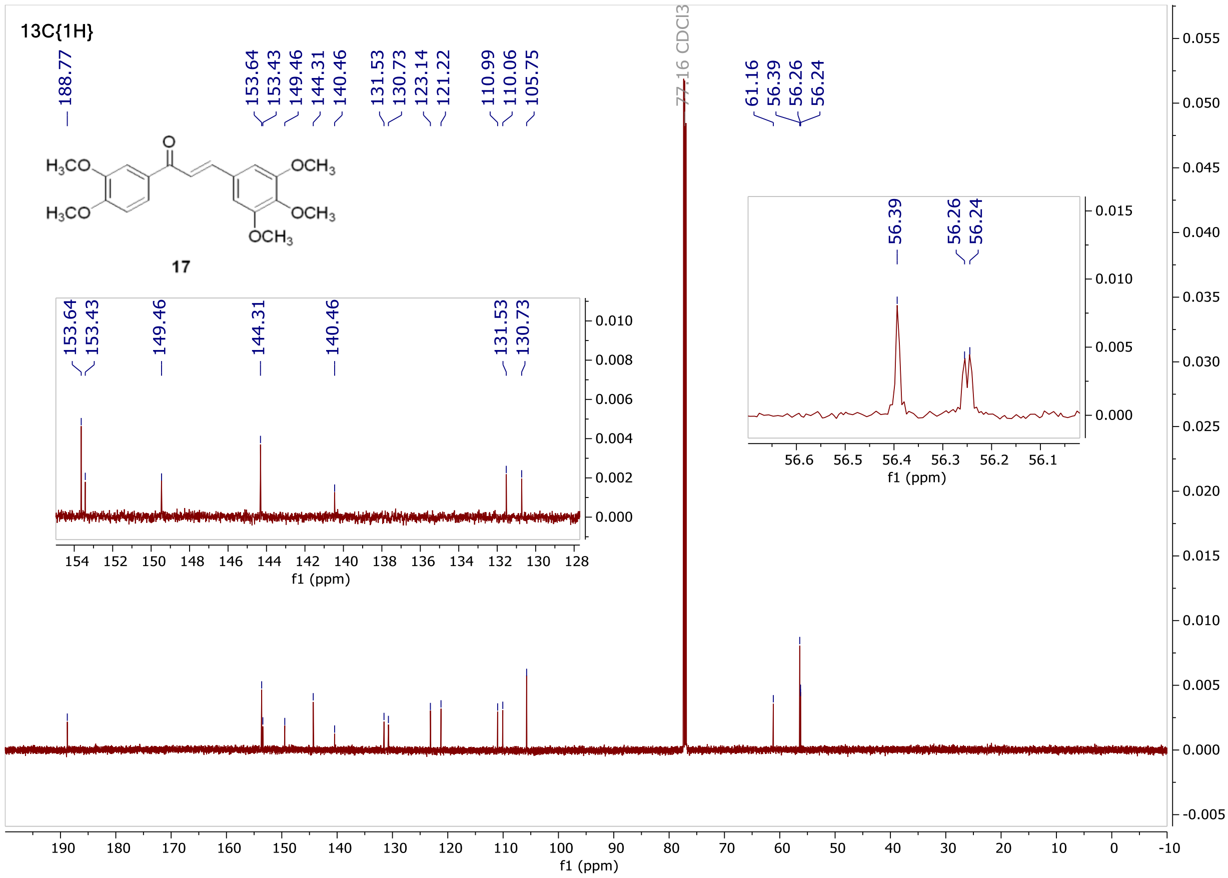
13C{1H}
188.77
153.64 153.43 149.46 144.31 140.46 131.53 130.73 123.14 121.22 110.99 110.06 105.75
77.16 CDCl3
61.16 56.39 56.26 56.24
17
f1 (ppm)

Determination of the Green Metrics

The E-factor and Process Mass Intensity (PMI) are probably the most common metrics used to assess the greenness of a process. We calculated them from our data and the results are here reported, to give an idea of the behaviour of the different reactions.

E-Factor calculation

The E-factor was calculated by taking into account the solvent used for the product extraction from the reaction mixture and the Na_2SO_4 used to dry the organic phase.

Synthesis of 3a in Ethanol

Compound **3a** in EtOH:

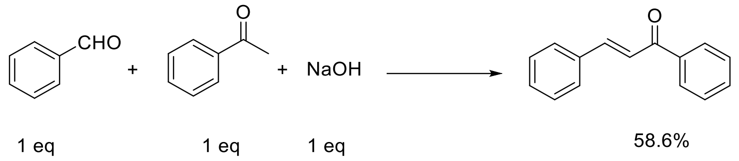

Amount of reactants:

Mass (aldehyde) = 106 mg

Mass (acetophenone) = 120 mg

Mass (NaOH) = 40 mg

Mass (EtOH) = 789 mg

Total Mass of Reactants = 1055 mg

Extraction solvent (EtOAc) = 902 mg

Na_2SO_4 = 100 mg

Total Mass of waste = 1934.1 mg

Amount of product = 122.9 mg

E-factor = (Mass of Waste)/(Mass of Product) = (1934.1) / (122.9) = 15.7

E-Factor = 15.7

Synthesis in CTAB

Chalcones from the Scope of the Aldehydes

Compound **3a**:

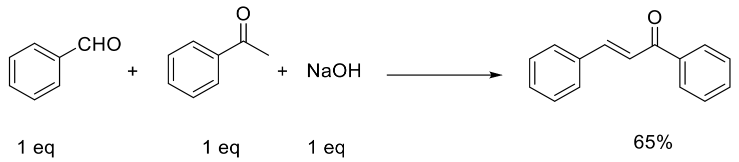

Amount of reactants:

Mass (aldehyde) = 106 mg

Mass (acetophenone) = 120 mg

Mass (NaOH) = 40 mg

Mass (CTAB) = 20 mg

Total Mass of Reactants = 286 mg

Extraction solvent (EtOAc) = 902 mg

Na_2SO_4 = 100 mg

Total Mass of waste = 1154.7

Amount of product = 134.5 mg

E-factor = (Mass of Waste)/(Mass of Product) = (1154.7) / (134.5) = 8.6

E-Factor = 8.6

Compound **3b**:

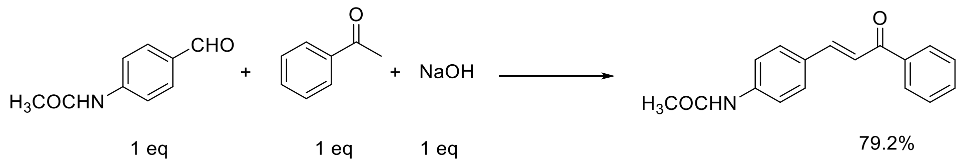

Amount of reactants:

Mass (aldehyde) = 163 mg

Mass (acetophenone) = 120 mg

Mass (NaOH) = 40 mg

Mass (CTAB) = 20 mg

Total Mass of Reactants = 343 mg

Extraction solvent (EtOAc) = 902 mg

Na_2SO_4 = 100 mg

Total Mass of waste = 1134.9

Amount of product = 210.1 mg

E-factor = (Mass of Waste)/(Mass of Product) = (1134.9) / (210.1) = 5.4

E-Factor = 5.4

Compound **3c**:

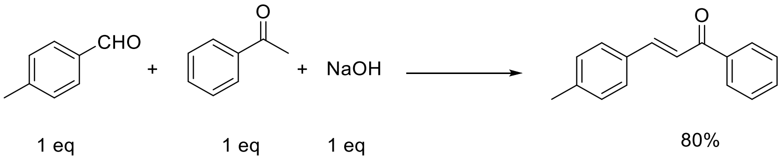

Amount of reactants:

Mass (aldehyde) = 120 mg

Mass (acetophenone) = 120 mg

Mass (NaOH) = 40 mg

Mass (CTAB) = 20 mg

Total Mass of Reactants = 300 mg

Extraction solvent (EtOAc) = 902 mg

Na_2SO_4 = 100 mg

Total Mass of waste = 1124.2

Amount of product = 177.8 mg

E-factor = (Mass of Waste)/(Mass of Product) = (1124.2) / (177.8) = 6.3

E-Factor = 6.3

Compound **3d**:

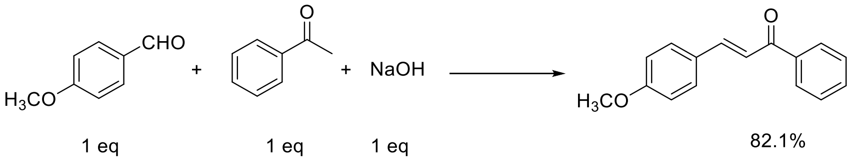

Amount of reactants:

Mass (aldehyde) = 136 mg

Mass (acetophenone) = 120 mg

Mass (NaOH) = 40 mg

Mass (CTAB) = 20 mg

Total Mass of Reactants = 316 mg

Extraction solvent (EtOAc) = 902 mg

Na_2SO_4 = 100 mg

Total Mass of waste = 1122.6

Amount of product = 195.4 mg

E-factor = (Mass of Waste)/(Mass of Product) = (1122.6) / (195.4) = 5.7

E-Factor = 5.7

Compound **3e**:

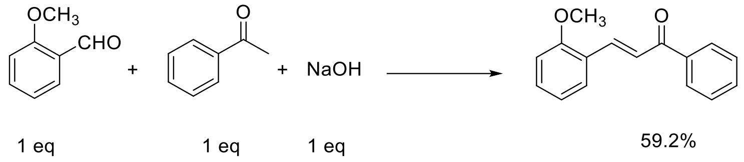

Amount of reactants:

Mass (aldehyde) = 164 mg

Mass (acetophenone) = 120 mg

Mass (NaOH) = 40 mg

Mass (CTAB) = 20 mg

Total Mass of Reactants = 316 mg

Extraction solvent (EtOAc) = 902 mg

Na_2SO_4 = 100 mg

Total Mass of waste = 1176.9

Amount of product = 141.1 mg

E-factor = (Mass of Waste)/(Mass of Product) = (1176.9) / (141.1) = 8.3

E-Factor = 8.3

Compound **3f**:

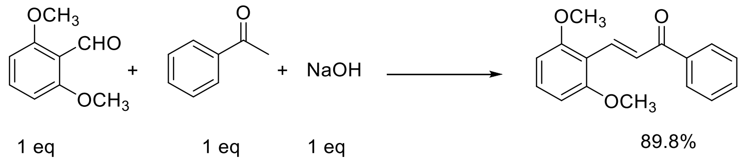

Amount of reactants:

Mass (aldehyde) = 166 mg

Mass (acetophenone) = 120 mg

Mass (NaOH) = 40 mg

Mass (CTAB) = 20 mg

Total Mass of Reactants = 346 mg

Extraction solvent (EtOAc) = 902 mg

Na_2SO_4 = 100 mg

Total Mass of waste = 1107.1 mg

Amount of product = 240.9 mg

E-factor = (Mass of Waste)/(Mass of Product) = (1107.1) / (240.9) = 4.6

E-Factor = 4.6

Compound **3g**:

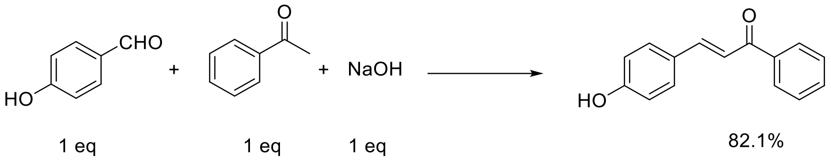

Amount of reactants:

Mass (aldehyde) = 122 mg

Mass (acetophenone) = 120 mg

Mass (NaOH) = 80 mg

Mass (CTAB) = 20 mg

Total Mass of Reactants = 342 mg

Extraction solvent (EtOAc) = 902 mg

Na_2SO_4 = 100 mg

Total Mass of Waste = 1260.8 mg

Amount of product = 83.2 mg

E-factor = (Mass of Waste)/(Mass of Product) = (1260.8) / (83.2) = 15.2

E-Factor = 15.2

Compound **3h**:

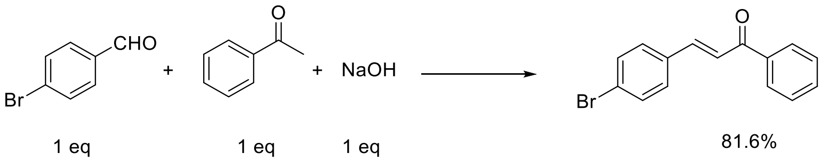

Amount of reactants:

Mass (aldehyde) = 185 mg

Mass (acetophenone) = 120 mg

Mass (NaOH) = 40 mg

Mass (CTAB) = 20 mg

Total Mass of Reactants = 365 mg

Extraction solvent (EtOAc) = 902 mg

Na_2SO_4 = 100 mg

Total Mass of Waste = 1132.7 mg

Amount of product = 234.7 mg

E-factor = (Mass of Waste)/(Mass of Product) = (1132.7) / (234.7) = 4.8

E-Factor = 4.8

Compound **3i**:

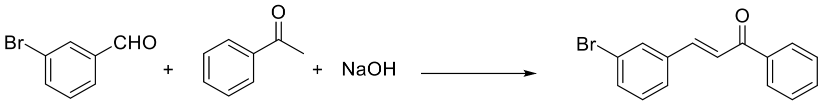

1 eq 1 eq 1 eq 90.0%

Amount of reactants:

Mass (aldehyde) = 185 mg

Mass (acetophenone) = 120 mg

Mass (NaOH) = 40 mg

Mass (CTAB) = 20 mg

Total Mass of Reactants = 365 mg

Extraction solvent (EtOAc) = 902 mg

Na_2SO_4 = 100 mg

Total Mass of Waste = 1108.6 mg

Amount of product = 258.4 mg

E-factor = (Mass of Waste)/(Mass of Product) = (1108.6) / (258.4) = 4.3

E-Factor = 4.3

Compound **3j**:

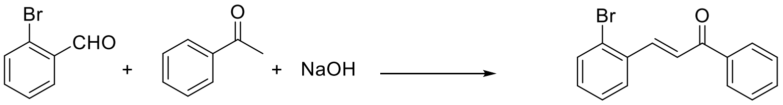

1 eq 1 eq 1 eq 54.6%

Amount of reactants:

Mass (aldehyde) = 185 mg

Mass (acetophenone) = 120 mg

Mass (NaOH) = 40 mg

Mass (CTAB) = 20 mg

Total Mass of Reactants = 365 mg

Extraction solvent (EtOAc) = 902 mg

Na_2SO_4 = 100 mg

Total Mass of Waste = 1210.2 mg

Amount of product = 156.8 mg

E-factor = (Mass of Waste)/(Mass of Product) = (1210.2) / (156.8) = 7.7

E-Factor = 7.7

Compound **3k** at 25°C:

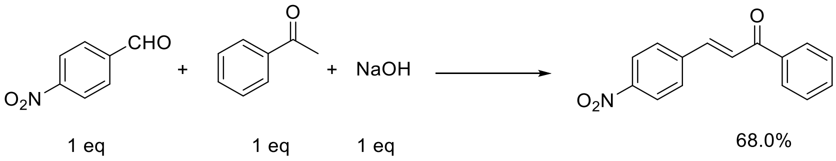

Amount of reactants:

Mass (aldehyde) = 151 mg

Mass (acetophenone) = 120 mg

Mass (NaOH) = 40 mg

Mass (CTAB) = 20 mg

Total Mass of Reactants = 331 mg

Extraction solvent (EtOAc) = 902 mg

Na_2SO_4 = 100 mg

Total Mass of Waste = 1160.8 mg

Amount of product = 172.2 mg

E-factor = (Mass of Waste)/(Mass of Product) = (1160.8) / (172.2) = 6.7

E-Factor = 6.7

Compound **3k** at 45°C:

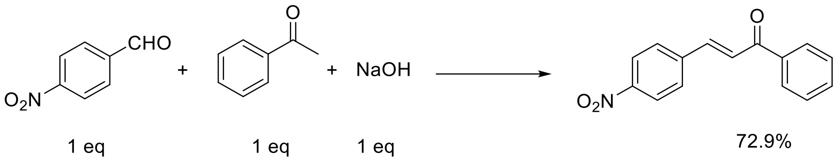

Amount of reactants:

Mass (aldehyde) = 151 mg

Mass (acetophenone) = 120 mg

Mass (NaOH) = 40 mg

Mass (CTAB) = 20 mg

Total Mass of Reactants = 331 mg

Extraction solvent (EtOAc) = 902 mg

Na_2SO_4 = 100 mg

Total Mass of Waste = 1147.6 mg

Amount of product = 184.6 mg

E-factor = (Mass of Waste)/(Mass of Product) = (1147.6) / (184.6) = 6.2

E-Factor = 6.2

Compound **3l**:

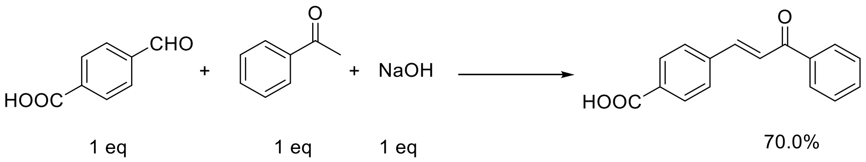

Amount of reactants:

Mass (aldehyde) = 150 mg

Mass (acetophenone) = 120 mg

Mass (NaOH) = 40 mg

Mass (CTAB) = 20 mg

Total Mass of Reactants = 330 mg

Extraction solvent (EtOAc) = 902 mg

Na_2SO_4 = 100 mg

Total Mass of Waste = 1155.4 mg

Amount of product = 176.6 mg

E-factor = (Mass of Waste)/(Mass of Product) = (1155.4) / (176.6) = 6.5

E-Factor = 6.5

Compound **3m**:

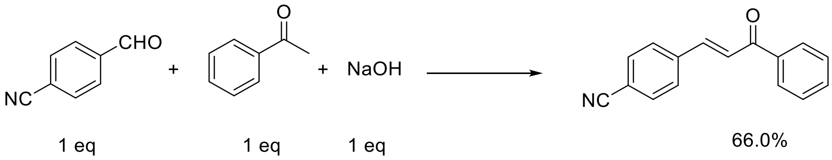

Amount of reactants:

Mass (aldehyde) = 131 mg

Mass (acetophenone) = 120 mg

Mass (NaOH) = 40 mg

Mass (CTAB) = 20 mg

Total Mass of Reactants = 311 mg

Extraction solvent (EtOAc) = 902 mg

Na_2SO_4 = 100 mg

Total Mass of Waste = 1158.1 mg

Amount of product = 154.9 mg

E-factor = (Mass of Waste)/(Mass of Product) = (1158.1) / (154.9) = 7.5

E-Factor = 7.5

Compound **3n**:

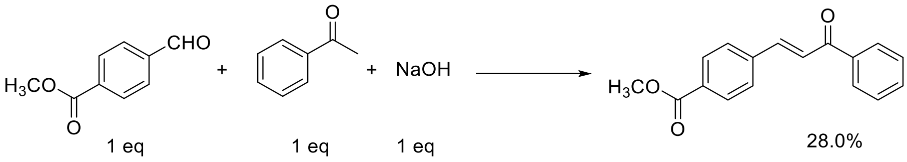

Amount of reactants:

Mass (aldehyde) = 164 mg

Mass (acetophenone) = 120 mg

Mass (NaOH) = 40 mg

Mass (CTAB) = 20 mg

Total Mass of Reactants = 344 mg

Extraction solvent (EtOAc) = 902 mg

Na_2SO_4 = 100 mg

Total Mass of Waste = 1271.4 mg

Amount of product = 74.6 mg

E-factor = (Mass of Waste)/(Mass of Product) = (1271.4) / (74.6) = 17.0

E-Factor = 17.0

Compound **3o**:

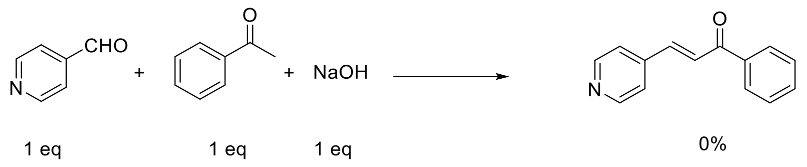

Amount of reactants:

Mass (aldehyde) = 107 mg

Mass (acetophenone) = 120 mg

Mass (NaOH) = 40 mg

Mass (CTAB) = 20 mg

Total Mass of Reactants = 287 mg

Extraction solvent (EtOAc) = 902 mg

Na_2SO_4 = 100 mg

Total Mass of Waste = 1285.3 mg

Amount of product = 0 mg

E-factor = (Mass of Waste)/(Mass of Product) = (1285.3) / (0) = -

E-Factor = -

Compound **3p**:

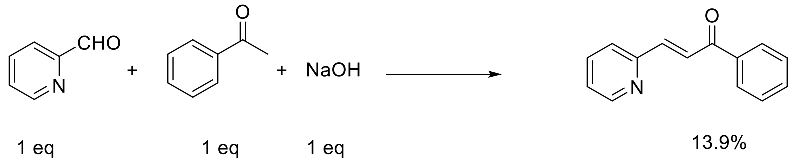

Amount of reactants:

Mass (aldehyde) = 107 mg

Mass (acetophenone) = 120 mg

Mass (NaOH) = 40 mg

Mass (CTAB) = 20 mg

Total Mass of Reactants = 287 mg

Extraction solvent (EtOAc) = 902 mg

Na_2SO_4 = 100 mg

Total Mass of Waste = 1259.9 mg

Amount of product = 29.1 mg

E-factor = (Mass of Waste)/(Mass of Product) = (1259.9) / (29.1) = 43.3

E-Factor = 43.3

Compound **3q**:

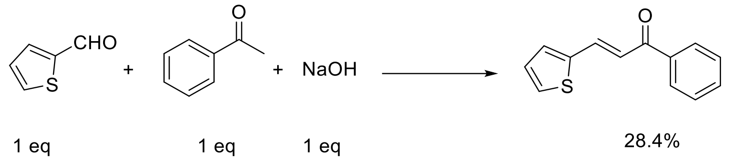

Amount of reactants:

Mass (aldehyde) = 112 mg

Mass (acetophenone) = 120 mg

Mass (NaOH) = 40 mg

Mass (CTAB) = 20 mg

Total Mass of Reactants = 292 mg

Extraction solvent (EtOAc) = 902 mg

Na_2SO_4 = 100 mg

Total Mass of Waste = 1233.1 mg

Amount of product = 60.9 mg

E-factor = (Mass of Waste)/(Mass of Product) = (1233.1) / (60.9) = 20.2

E-Factor = 20.2

Compound **3r**:

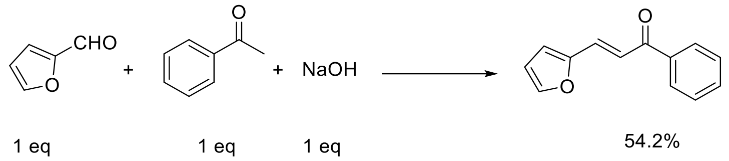

Amount of reactants:

Mass (aldehyde) = 96.1 mg

Mass (acetophenone) = 120 mg

Mass (NaOH) = 40 mg

Mass (CTAB) = 20 mg

Total Mass of Reactants = 276.1 mg

Extraction solvent (EtOAc) = 902 mg

Na_2SO_4 = 100 mg

Total Mass of Waste = 1170.7 mg

Amount of product = 107.4 mg

E-factor = (Mass of Waste)/(Mass of Product) = (1170.7) / (107.4) = 10.9

E-Factor = 10.9

Compound **3s**:

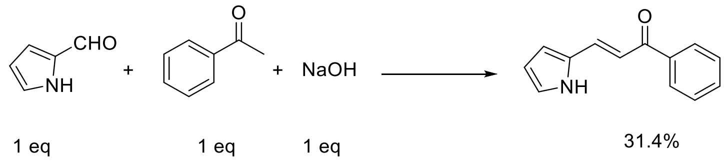

Amount of reactants:

Mass (aldehyde) = 95.1 mg

Mass (acetophenone) = 120 mg

Mass (NaOH) = 40 mg

Mass (CTAB) = 20 mg

Total Mass of Reactants = 275.1 mg

Extraction solvent (EtOAc) = 902 mg

Na_2SO_4 = 100 mg

Total Mass of Waste = 1183.4 mg

Amount of product = 61.9 mg

E-factor = (Mass of Waste)/(Mass of Product) = (1183.4) / (61.9) = 19.6

E-Factor = 19.6

Chalcones from the Scope of the Acetophenones

Compound **6b**:

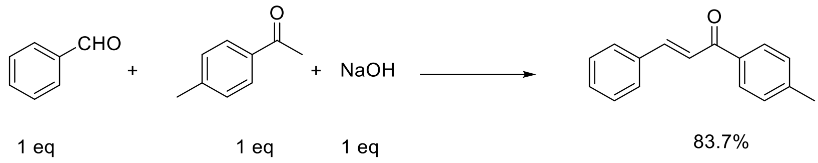

Amount of reactants:

Mass (aldehyde) = 106 mg

Mass (acetophenone) = 134 mg

Mass (NaOH) = 40 mg

Mass (CTAB) = 20 mg

Total Mass of Reactants = 300 mg

Extraction solvent (EtOAc) = 902 mg

Na_2SO_4 = 100 mg

Total Mass of Waste = 1115.9 mg

Amount of product = 186.1 mg

E-factor = (Mass of Waste)/(Mass of Product) = (1115.9) / (186.1) = 6.1

E-Factor = 6.1

Compound **6c**:

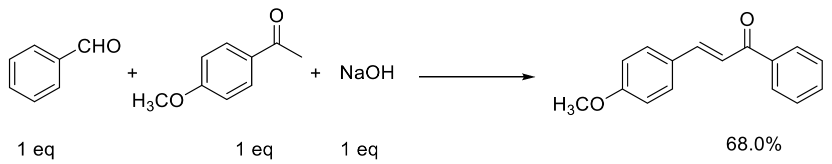

Amount of reactants:

Mass (aldehyde) = 104 mg

Mass (acetophenone) = 150 mg

Mass (NaOH) = 40 mg

Mass (CTAB) = 20 mg

Total Mass of Reactants = 316 mg

Extraction solvent (EtOAc) = 902 mg

Na_2SO_4 = 100 mg

Total Mass of Waste = 1156 mg

Amount of product = 162.0 mg

E-factor = (Mass of Waste)/(Mass of Product) = (1156.0) / (162.0) = 7.1

E-Factor = 7.1

Compound **6d**:

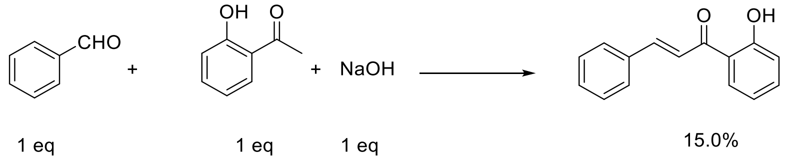

Amount of reactants:

Mass (aldehyde) = 106 mg

Mass (acetophenone) = 136 mg

Mass (NaOH) = 40 mg

Mass (CTAB) = 20 mg

Total Mass of Reactants = 302 mg

Extraction solvent (EtOAc) = 902 mg

Na_2SO_4 = 100 mg

Total Mass of Waste = 1270.4 mg

Amount of product = 33.6 mg

E-factor = (Mass of Waste)/(Mass of Product) = (1270.4) / (33.6) = 37.8

E-Factor = 37.8

Compound **6e**:

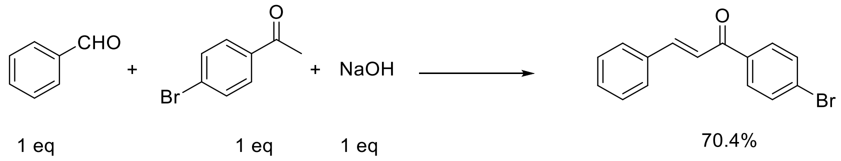

Amount of reactants:

Mass (aldehyde) = 106 mg

Mass (acetophenone) = 199 mg

Mass (NaOH) = 40 mg

Mass (CTAB) = 20 mg

Total Mass of Reactants = 365 mg

Extraction solvent (EtOAc) = 902 mg

Na_2SO_4 = 100 mg

Total Mass of Waste = 1164.8 mg

Amount of product = 202.2 mg

E-factor = (Mass of Waste)/(Mass of Product) = (1164.8) / (202.2) = 5.8

E-Factor = 5.8

Compound **6f**:

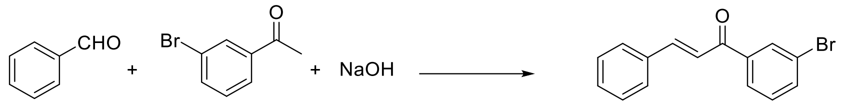

1 eq 1 eq 1 eq 66.8%

Amount of reactants:

Mass (aldehyde) = 106 mg

Mass (acetophenone) = 196 mg

Mass (NaOH) = 40 mg

Mass (CTAB) = 20 mg

Total Mass of Reactants = 365 mg

Extraction solvent (EtOAc) = 902 mg

Na_2SO_4 = 100 mg

Total Mass of Waste = 1175.4 mg

Amount of product = 191.8 mg

E-factor = (Mass of Waste)/(Mass of Product) = (1175.4) / (191.8) = 6.1

E-Factor = 6.1

Compound **6g**:

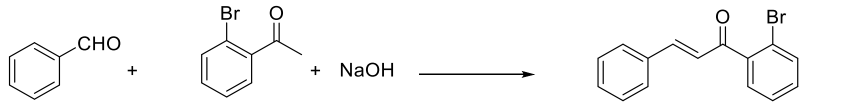

1 eq 1 eq 1 eq 80.0%

Amount of reactants:

Mass (aldehyde) = 106 mg

Mass (acetophenone) = 199 mg

Mass (NaOH) = 40 mg

Mass (CTAB) = 20 mg

Total Mass of Reactants = 365 mg

Extraction solvent (EtOAc) = 902 mg

Na_2SO_4 = 100 mg

Total Mass of Waste = 1137.3 mg

Amount of product = 229.7 mg

E-factor = (Mass of Waste)/(Mass of Product) = (1137.3) / (229.7) = 5.0

E-Factor = 5.0

Compound **6h**:

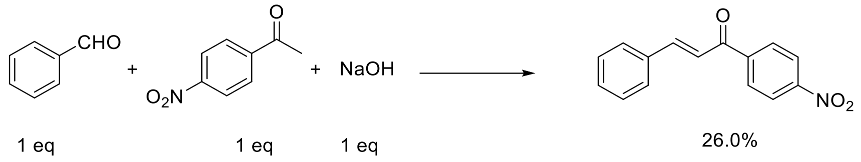

Amount of reactants:

Mass (aldehyde) = 106 mg

Mass (acetophenone) = 165 mg

Mass (NaOH) = 40 mg

Mass (CTAB) = 20 mg

Total Mass of Reactants = 331 mg

Extraction solvent (EtOAc) = 902 mg

Na_2SO_4 = 100 mg

Total Mass of Waste = 1267.2 mg

Amount of product = 65.8 mg

E-factor = (Mass of Waste)/(Mass of Product) = (1267.2) / (65.8) = 19.3

E-Factor = 19.3

Compound **6i**:

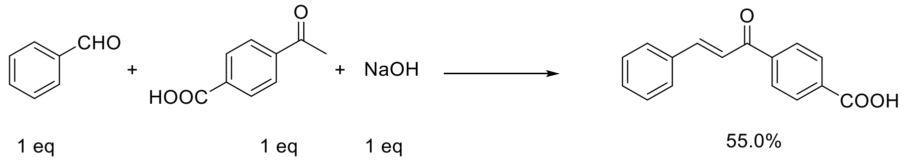

Amount of reactants:

Mass (aldehyde) = 106 mg

Mass (acetophenone) = 164 mg

Mass (NaOH) = 40 mg

Mass (CTAB) = 20 mg

Total Mass of Reactants = 330 mg

Extraction solvent (EtOAc) = 902 mg

Na_2SO_4 = 100 mg

Total Mass of Waste = 1193.3 mg

Amount of product = 138.7 mg

E-factor = (Mass of Waste)/(Mass of Product) = (1193.3) / (138.7) = 8.6

E-Factor = 8.6

Compound **6j**:

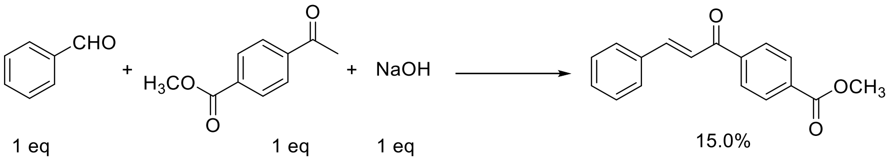

Amount of reactants:

Mass (aldehyde) = 106 mg

Mass (acetophenone) = 175 mg

Mass (NaOH) = 40 mg

Mass (CTAB) = 20 mg

Total Mass of Reactants = 344 mg

Extraction solvent (EtOAc) = 902 mg

Na_2SO_4 = 100 mg

Total Mass of Waste = 1306.1 mg

Amount of product = 39.9 mg

E-factor = (Mass of Waste)/(Mass of Product) = (1306.1) / (39.9) = 32.7

E-Factor = 32.7

Compound **6k**:

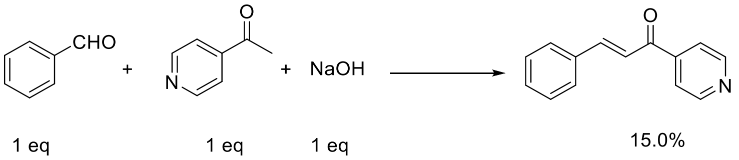

Amount of reactants:

Mass (aldehyde) = 106 mg

Mass (acetophenone) = 121 mg

Mass (NaOH) = 40 mg

Mass (CTAB) = 20 mg

Total Mass of Reactants = 287 mg

Extraction solvent (EtOAc) = 902 mg

Na_2SO_4 = 100 mg

Total Mass of Waste = 1289 mg

Amount of product = 0 mg

E-Factor = -

Compound **6l**:

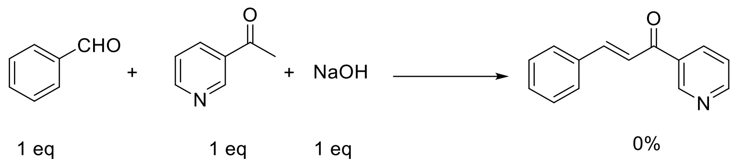

Amount of reactants:

Mass (aldehyde) = 106 mg

Mass (acetophenone) = 121 mg

Mass (NaOH) = 40 mg

Mass (CTAB) = 20 mg

Total Mass of Reactants = 287 mg

Extraction solvent (EtOAc) = 902 mg

Na_2SO_4 = 100 mg

Total Mass of Waste = 1289 mg

Amount of product = 0 mg

E-Factor = -

Compound **6m**:

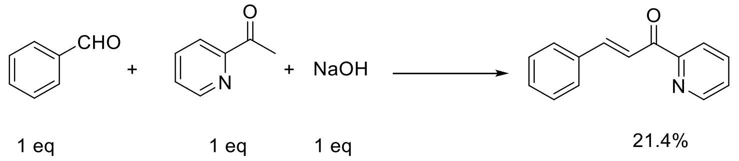

Amount of reactants:

Mass (aldehyde) = 106 mg

Mass (acetophenone) = 121 mg

Mass (NaOH) = 40 mg

Mass (CTAB) = 20 mg

Total Mass of Reactants = 287 mg

Extraction solvent (EtOAc) = 902 mg

Na_2SO_4 = 100 mg

Total Mass of Waste = 1244.2 mg

Amount of product = 44.8 mg

E-factor = (Mass of Waste)/(Mass of Product) = (1244.2) / (44.8) = 27.8

E-Factor = 27.8

Compound **6n**:

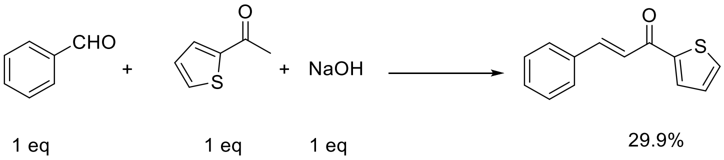

Amount of reactants:

Mass (aldehyde) = 104 mg

Mass (acetophenone) = 124 mg

Mass (NaOH) = 39.3 mg

Mass (CTAB) = 20 mg

Total Mass of Reactants = 292 mg

Extraction solvent (EtOAc) = 902 mg

Na_2SO_4 = 100 mg

Total Mass of Waste = 1229.9 mg

Amount of product = 64.1 mg

E-factor = (Mass of Waste)/(Mass of Product) = (1229.9) / (64.1) = 19.2

E-Factor = 19.2

Compound **6o**:

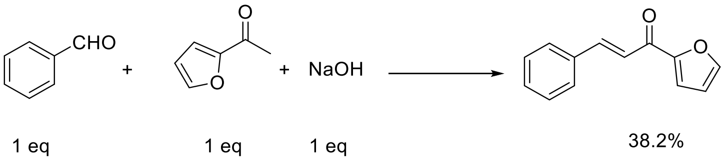

Amount of reactants:

Mass (aldehyde) = 106 mg

Mass (acetophenone) = 110 mg

Mass (NaOH) = 40 mg

Mass (CTAB) = 20 mg

Total Mass of Reactants = 276 mg

Extraction solvent (EtOAc) = 902 mg

Na_2SO_4 = 100 mg

Total Mass of Waste = 1202.3 mg

Amount of product = 75.7 mg

E-factor = (Mass of Waste)/(Mass of Product) = (1202.3) / (75.7) = 15.9

E-Factor = 15.9

Compound **6p**:

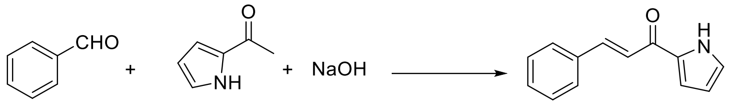

1 eq 1 eq 1 eq 92.0%

Amount of reactants:

Mass (aldehyde) = 106 mg

Mass (acetophenone) = 109 mg

Mass (NaOH) = 40 mg

Mass (CTAB) = 20 mg

Total Mass of Reactants = 275 mg

Extraction solvent (EtOAc) = 902 mg

Na_2SO_4 = 100 mg

Total Mass of Waste = 1095.5 mg

Amount of product = 181.5 mg

E-factor = (Mass of Waste)/(Mass of Product) = (1095.5) / (181.5) = 6.0

E-Factor = 6.0

Representative Chalcones

Compound **9**:

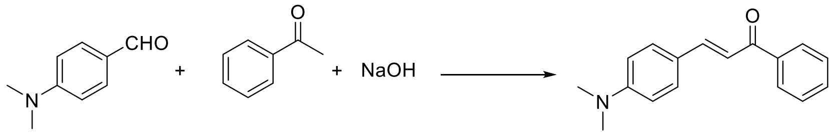

1 eq 1 eq 1 eq 92.0%

Amount of reactants:

Mass (aldehyde) = 149 mg

Mass (acetophenone) = 120 mg

Mass (NaOH) = 40 mg

Mass (CTAB) = 20 mg

Total Mass of Reactants = 329 mg

Extraction solvent (EtOAc) = 902 mg

Na_2SO_4 = 100 mg

Total Mass of Waste = 1170.4 mg

Amount of product = 158.1 mg

E-factor = (Mass of Waste)/(Mass of Product) = (1170.4) / (158.1) = 7.4

E-Factor = 7.4

Compound **10** at 25°C:

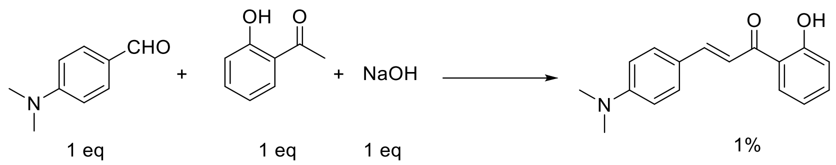

Amount of reactants:

Mass (aldehyde) = 149 mg

Mass (acetophenone) = 136 mg

Mass (NaOH) = 160 mg

Mass (CTAB) = 20 mg

Total Mass of Reactants = 465 mg

Extraction solvent (EtOAc) = 902 mg

Na_2SO_4 = 100 mg

Total Mass of Waste = 1464.2 mg

Amount of product = 2.7 mg

E-factor = (Mass of Waste)/(Mass of Product) = (1464.2) / (2.7) = 542.3

E-Factor = 542.3

Compound **10** at 45°C:

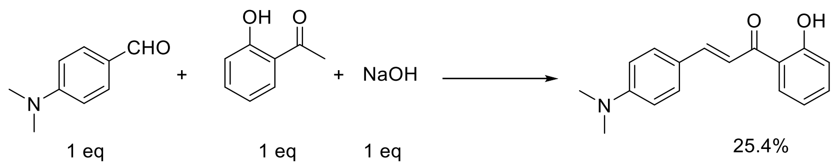

Amount of reactants:

Mass (aldehyde) = 149 mg

Mass (acetophenone) = 136 mg

Mass (NaOH) = 160 mg

Mass (CTAB) = 20 mg

Total Mass of Reactants = 465 mg

Extraction solvent (EtOAc) = 902 mg

Na_2SO_4 = 100 mg

Total Mass of Waste = 1399.1 mg

Amount of product = 67.9 mg

E-factor = (Mass of Waste)/(Mass of Product) = (1399.1) / (67.9) = 20.6

E-Factor = 20.6

Compound **11** at 25°C:

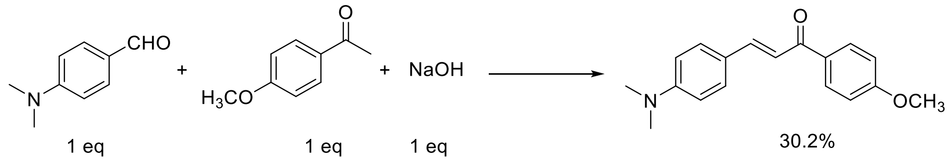

Amount of reactants:

Mass (aldehyde) = 149 mg

Mass (acetophenone) = 150 mg

Mass (NaOH) = 40 mg

Mass (CTAB) = 20 mg

Total Mass of Reactants = 359 mg

Extraction solvent (EtOAc) = 902 mg

Na_2SO_4 = 100 mg

Total Mass of Waste = 1276.9 mg

Amount of product = 85.0 mg

E-factor = (Mass of Waste)/(Mass of Product) = (1276.9) / (85.0) = 15.0

E-Factor = 15.0

Compound **11** at 45°C:

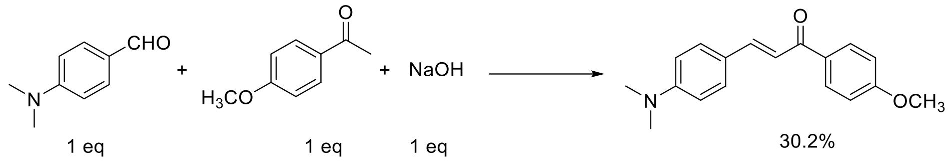

Amount of reactants:

Mass (aldehyde) = 149 mg

Mass (acetophenone) = 150 mg

Mass (NaOH) = 40 mg

Mass (CTAB) = 20 mg

Total Mass of Reactants = 359 mg

Extraction solvent (EtOAc) = 902 mg

Na_2SO_4 = 100 mg

Total Mass of Waste = 1277.4 mg

Amount of product = 83.6 mg

E-factor = (Mass of Waste)/(Mass of Product) = (1277.4) / (83.6) = 15.3

E-Factor = 16.9

Compound **12**:

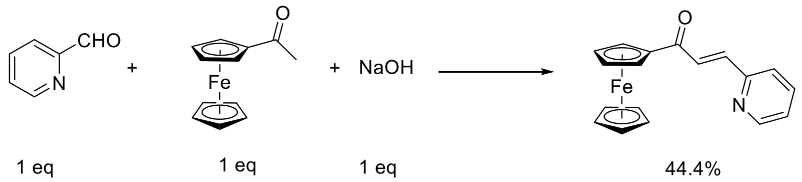

Amount of reactants:

Mass (aldehyde) = 106 mg

Mass (acetophenone) = 228 mg

Mass (NaOH) = 40 mg

Mass (CTAB) = 20 mg

Total Mass of Reactants = 394 mg

Extraction solvent (EtOAc) = 902 mg

Na_2SO_4 = 100 mg

Total Mass of Waste = 1255.2 mg

Amount of product = 140.8 mg

E-factor = (Mass of Waste)/(Mass of Product) = (1255.2) / (140.8) = 9.0

E-Factor = 9.0

Compound **13** at 25°C:

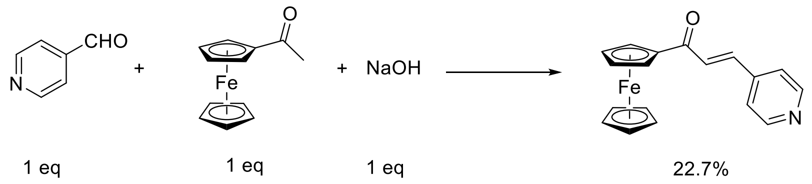

Amount of reactants:

Mass (aldehyde) = 106 mg

Mass (acetophenone) = 228 mg

Mass (NaOH) = 40 mg

Mass (CTAB) = 20 mg

Total Mass of Reactants = 394 mg

Extraction solvent (EtOAc) = 902 mg

Na_2SO_4 = 100 mg

Total Mass of Waste = 1324.0 mg

Amount of product = 72.0 mg

E-factor = (Mass of Waste)/(Mass of Product) = (1324.0) / (72.0) = 18.4

E-Factor = 18.4

Compound **13** at 45°C:

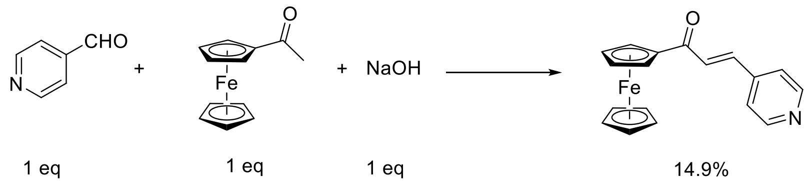

Amount of reactants:

Mass (aldehyde) = 106 mg

Mass (acetophenone) = 228 mg

Mass (NaOH) = 40 mg

Mass (CTAB) = 20 mg

Total Mass of Reactants = 394 mg

Extraction solvent (EtOAc) = 902 mg

Na_2SO_4 = 100 mg

Total Mass of Waste = 1348.7 mg

Amount of product = 47.3 mg

E-factor = (Mass of Waste)/(Mass of Product) = (1348.7) / (47.3) = 28.5

E-Factor = 28.5

Compound **14**:

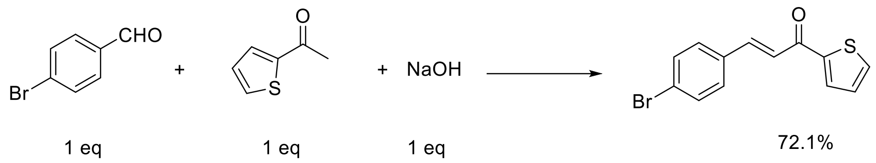

Amount of reactants:

Mass (aldehyde) = 185 mg

Mass (acetophenone) = 126 mg

Mass (NaOH) = 40 mg

Mass (CTAB) = 20 mg

Total Mass of Reactants = 371 mg

Extraction solvent (EtOAc) = 902 mg

Na_2SO_4 = 100 mg

Total Mass of Waste = 1161.6 mg

Amount of product = 211.4 mg

E-factor = (Mass of Waste)/(Mass of Product) = (1161.6) / (211.4) = 5.5

E-Factor = 5.5

Compound **15**:

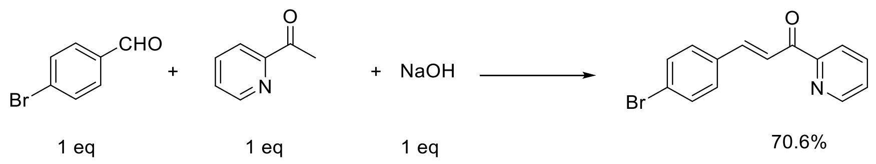

Amount of reactants:

Mass (aldehyde) = 185 mg

Mass (acetophenone) = 121 mg

Mass (NaOH) = 40 mg

Mass (CTAB) = 20 mg

Total Mass of Reactants = 366 mg

Extraction solvent (EtOAc) = 902 mg

Na_2SO_4 = 100 mg

Total Mass of Waste = 1158.6 mg

Amount of product = 203.4 mg

E-factor = (Mass of Waste)/(Mass of Product) = (1158.6) / (203.4) = 5.7

E-Factor = 5.7

Compound **16**:

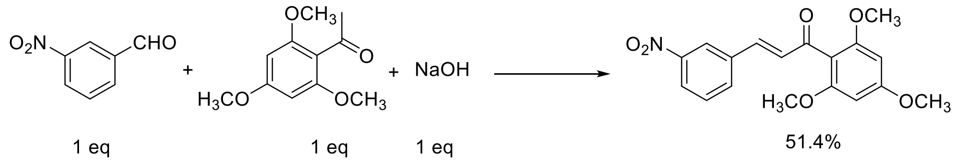

Amount of reactants:

Mass (aldehyde) = 151 mg

Mass (acetophenone) = 210 mg

Mass (NaOH) = 40 mg

Mass (CTAB) = 20 mg

Total Mass of Reactants = 421 mg

Extraction solvent (EtOAc) = 902 mg

Na_2SO_4 = 100 mg

Total Mass of Waste = 1246.6 mg

Amount of product = 176.4 mg

E-factor = (Mass of Waste)/(Mass of Product) = (1246.6) / (176.4) = 7.1

E-Factor = 7.1

Compound **17**:

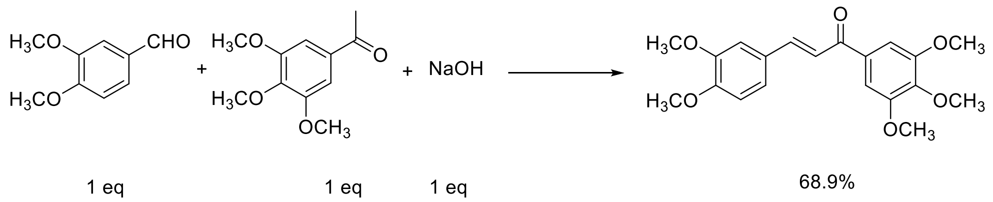

Amount of reactants:

Mass (aldehyde) = 196 mg

Mass (acetophenone) = 180 mg

Mass (NaOH) = 39.3 mg

Mass (CTAB) = 20 mg

Total Mass of Reactants = 436 mg

Extraction solvent (EtOAc) = 902 mg

Na_2SO_4 = 100 mg

Total Mass of Waste = 1191.1 mg

Amount of product = 246.9 mg

E-factor = (Mass of Waste)/(Mass of Product) = (1191.1) / (246.9) = 4.8

E-Factor = 4.8

Synthesis in TWEEN 80

Chalcones from the Scope of the Aldehydes

Compound **3a**:

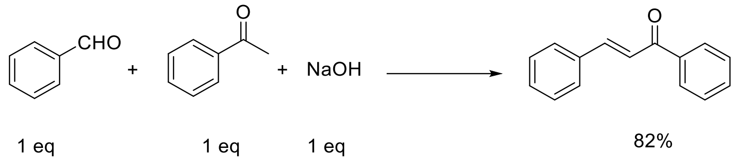

Amount of reactants:

Mass (aldehyde) = 106 mg

Mass (acetophenone) = 120 mg

Mass (NaOH) = 40 mg

Mass (Tween 80) = 20 mg

Total Mass of Reactants = 286 mg

Extraction solvent (EtOAc) = 902 mg

Na_2SO_4 = 100 mg

Total Mass of Waste = 1117.9 mg

Amount of product = 170.8 mg

E-factor = (Mass of Waste)/(Mass of Product) = (1117.9) / (170.8) = 6.6

E-Factor = 6.6

Compound **3b**:

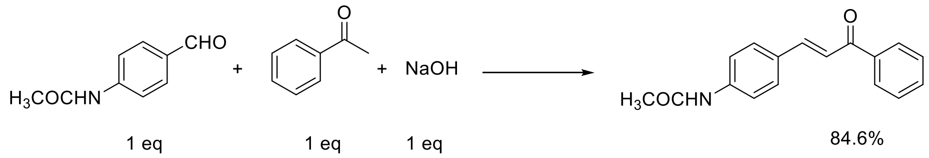

Amount of reactants:

Mass (aldehyde) = 163 mg

Mass (acetophenone) = 120 mg

Mass (NaOH) = 40 mg

Mass (Tween 80) = 20 mg

Total Mass of Reactants = 343 mg

Extraction solvent (EtOAc) = 902 mg

Na_2SO_4 = 100 mg

Total Mass of Waste = 1120.5 mg

Amount of product = 224.5 mg

E-factor = (Mass of Waste)/(Mass of Product) = (1120.5) / (224.5) = 5.0

E-Factor = 5.0

Compound **3c**:

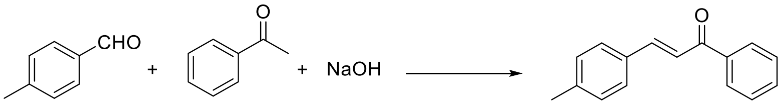

1 eq 1 eq 1 eq 80%

Amount of reactants:

Mass (aldehyde) = 118 mg

Mass (acetophenone) = 119 mg

Mass (NaOH) = 39.3 mg

Mass (Tween 80) = 20 mg

Total Mass of Reactants = 300 mg

Extraction solvent (EtOAc) = 902 mg

Na_2SO_4 = 100 mg

Total Mass of Waste = 1123.9 mg

Amount of product = 172.9 mg

E-factor = (Mass of Waste)/(Mass of Product) = (1123.9) / (172.9) = 6.5

E-Factor = 6.5

Compound **3d** at 25°C:

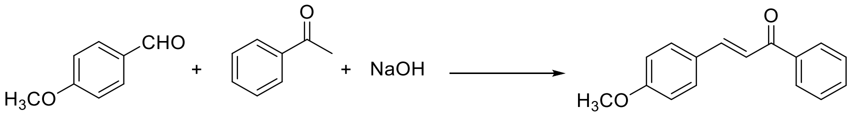

1 eq 1 eq 1 eq 34.0%

Amount of reactants:

Mass (aldehyde) = 136 mg

Mass (acetophenone) = 120 mg

Mass (NaOH) = 40 mg

Mass (Tween 80) = 20 mg

Total Mass of Reactants = 316 mg

Extraction solvent (EtOAc) = 902 mg

Na_2SO_4 = 100 mg

Total Mass of Waste = 1237 mg

Amount of product = 81.0 mg

E-factor = (Mass of Waste)/(Mass of Product) = (1237.0) / (81.0) = 15.3

E-Factor = 15.3

Compound **3d** at 45°C:

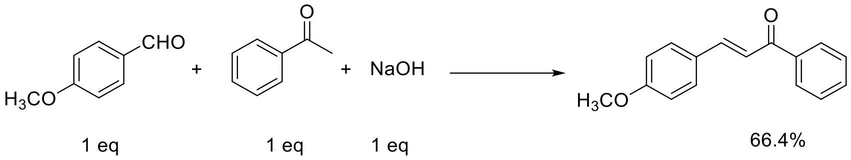

Amount of reactants:

Mass (aldehyde) = 136 mg

Mass (acetophenone) = 120 mg

Mass (NaOH) = 40 mg

Mass (Tween 80) = 20 mg

Total Mass of Reactants = 316 mg

Extraction solvent (EtOAc) = 902 mg

Na_2SO_4 = 100 mg

Total Mass of Waste = 1159.8 mg

Amount of product = 158.2 mg

E-factor = (Mass of Waste)/(Mass of Product) = (1159.8) / (158.2) = 7.3

E-Factor = 7.3

Compound **3e**:

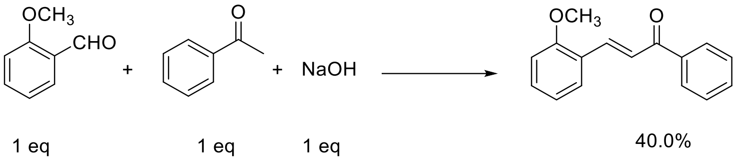

Amount of reactants:

Mass (aldehyde) = 136 mg

Mass (acetophenone) = 120 mg

Mass (NaOH) = 40 mg

Mass (Tween 80) = 20 mg

Total Mass of Reactants = 316 mg

Extraction solvent (EtOAc) = 902 mg

Na_2SO_4 = 100 mg

Total Mass of Waste = 1220.5 mg

Amount of product = 95.3 mg

E-factor = (Mass of Waste)/(Mass of Product) = (1220.5) / (95.3) = 12.8

E-Factor = 12.8

Compound **3f** at 25°C:

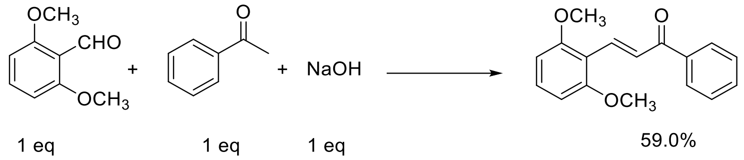

Amount of reactants:

Mass (aldehyde) = 166 mg

Mass (acetophenone) = 120 mg

Mass (NaOH) = 40 mg

Mass (Tween 80) = 20 mg

Total Mass of Reactants = 346 mg

Extraction solvent (EtOAc) = 902 mg

Na_2SO_4 = 100 mg

Total Mass of Waste = 1189.57 mg

Amount of product = 158.3 mg

E-factor = (Mass of Waste)/(Mass of Product) = (1189.7) / (158.3) = 7.5

E-Factor = 7.5

Compound **3f** at 45°C:

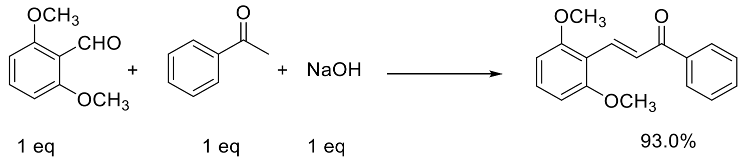

Amount of reactants:

Mass (aldehyde) = 166 mg

Mass (acetophenone) = 120 mg

Mass (NaOH) = 40 mg

Mass (Tween 80) = 20 mg

Total Mass of Reactants = 346 mg

Extraction solvent (EtOAc) = 902 mg

Na_2SO_4 = 100 mg

Total Mass of Waste = 1098.8 mg

Amount of product = 249.5 mg

E-factor = (Mass of Waste)/(Mass of Product) = (1098.8) / (249.5) = 4.4

E-Factor = 4.4

Compound **3g**:

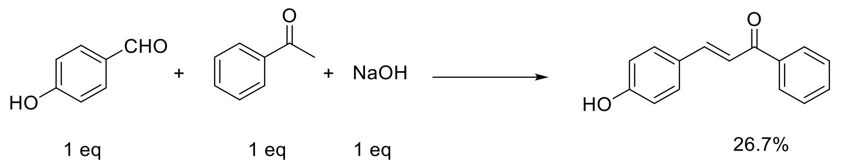

Amount of reactants:

Mass (aldehyde) = 122 mg

Mass (acetophenone) = 120 mg

Mass (NaOH) = 80 mg

Mass (Tween 80) = 20 mg

Total Mass of Reactants = 342 mg

Extraction solvent (EtOAc) = 902 mg

Na_2SO_4 = 100 mg

Total Mass of Waste = 1284.1 mg

Amount of product = 59.9 mg

E-factor = (Mass of Waste)/(Mass of Product) = (1284.1) / (59.9) = 21.4

E-Factor = 21.4

Compound **3h** at 25°C:

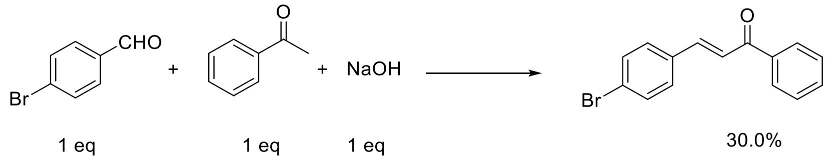

Amount of reactants:

Mass (aldehyde) = 185 mg

Mass (acetophenone) = 120 mg

Mass (NaOH) = 40 mg

Mass (Tween 80) = 20 mg

Total Mass of Reactants = 365 mg

Extraction solvent (EtOAc) = 902 mg

Na_2SO_4 = 100 mg

Total Mass of Waste = 1280.9 mg

Amount of product = 86.1 mg

E-factor = (Mass of Waste)/(Mass of Product) = (1280.9) / (86.1) = 14.9

E-Factor = 14.9

Compound **3h** at 45°C:

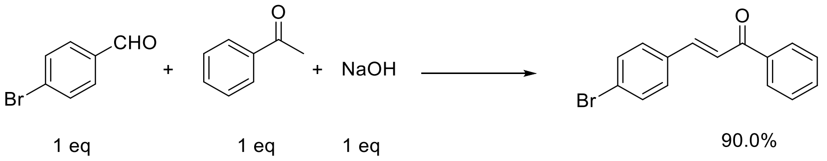

Amount of reactants:

Mass (aldehyde) = 185 mg

Mass (acetophenone) = 120 mg

Mass (NaOH) = 40 mg

Mass (Tween 80) = 20 mg

Total Mass of Reactants = 365 mg

Extraction solvent (EtOAc) = 902 mg

Na_2SO_4 = 100 mg

Total Mass of Waste = 1108.0 mg

Amount of product = 258.4 mg

E-factor = (Mass of Waste)/(Mass of Product) = (1108.0) / (258.4) = 4.3

E-Factor = 4.3

Compound **3i**:

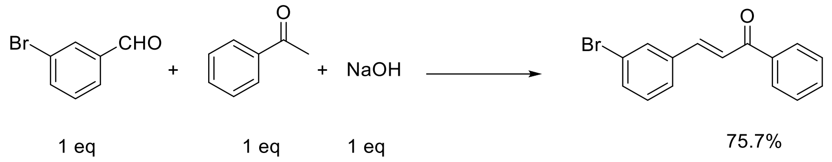

Amount of reactants:

Mass (aldehyde) = 185 mg

Mass (acetophenone) = 120 mg

Mass (NaOH) = 40 mg

Mass (Tween 80) = 20 mg

Total Mass of Reactants = 365 mg

Extraction solvent (EtOAc) = 902 mg

Na_2SO_4 = 100 mg

Total Mass of Waste = 1149.6 mg

Amount of product = 217.4 mg

E-factor = (Mass of Waste)/(Mass of Product) = (1149.6) / (217.4) = 5.3

E-Factor = 5.3

Compound **3j** at 25°C:

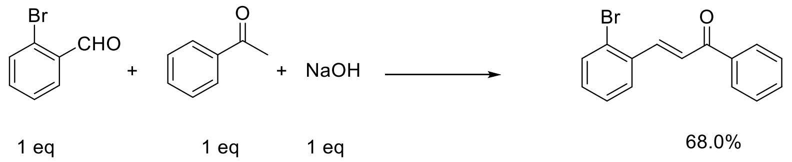

Amount of reactants:

Mass (aldehyde) = 185 mg

Mass (acetophenone) = 120 mg

Mass (NaOH) = 40 mg

Mass (Tween 80) = 20 mg

Total Mass of Reactants = 365 mg

Extraction solvent (EtOAc) = 902 mg

Na_2SO_4 = 100 mg

Total Mass of Waste = 1171.7 mg

Amount of product = 195.3 mg

E-factor = (Mass of Waste)/(Mass of Product) = (1171.7) / (195.3) = 6.0

E-Factor = 6.0

Compound **3j** at 45°C:

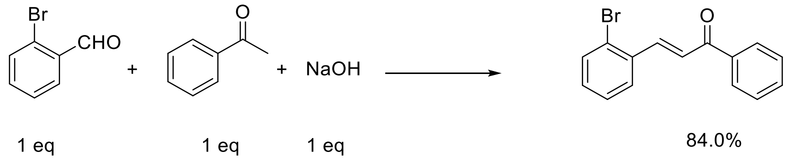

Amount of reactants:

Mass (aldehyde) = 185 mg

Mass (acetophenone) = 120 mg

Mass (NaOH) = 40 mg

Mass (Tween 80) = 20 mg

Total Mass of Reactants = 365 mg

Extraction solvent (EtOAc) = 902 mg

Na_2SO_4 = 100 mg

Total Mass of Waste = 1125.8 mg

Amount of product = 241.2 mg

E-factor = (Mass of Waste)/(Mass of Product) = (1125.8) / (241.2) = 4.7

E-Factor = 4.7

Compound **3k** at 25°C:

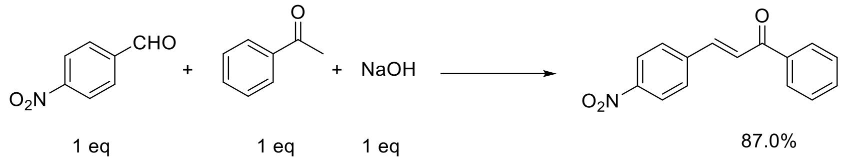

Amount of reactants:

Mass (aldehyde) = 151 mg

Mass (acetophenone) = 120 mg

Mass (NaOH) = 40 mg

Mass (Tween 80) = 20 mg

Total Mass of Reactants = 331 mg

Extraction solvent (EtOAc) = 902 mg

Na_2SO_4 = 100 mg

Total Mass of Waste = 1112.7 mg

Amount of product = 220.3 mg

E-factor = (Mass of Waste)/(Mass of Product) = (1112.7) / (220.3) = 5.1

E-Factor = 5.1

Compound **3l**:

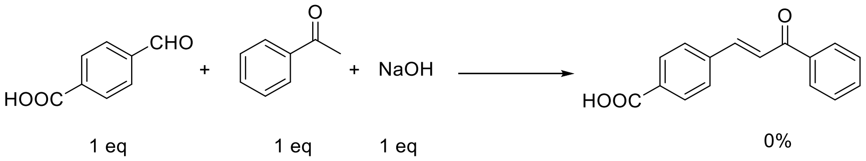

Amount of reactants:

Mass (aldehyde) = 150 mg

Mass (acetophenone) = 120 mg

Mass (NaOH) = 40 mg

Mass (Tween 80) = 20 mg

Total Mass of Reactants = 330 mg

Extraction solvent (EtOAc) = 902 mg

Na_2SO_4 = 100 mg

Total Mass of Waste = 1332 mg

Amount of product = 0 mg

E-Factor = -

Compound **3m**:

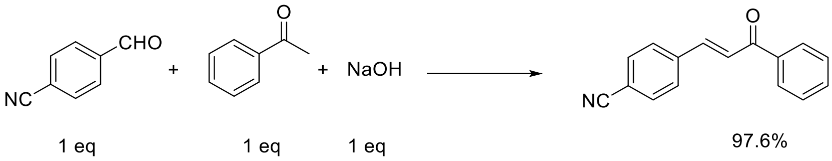

Amount of reactants:

Mass (aldehyde) = 131 mg

Mass (acetophenone) = 120 mg

Mass (NaOH) = 40 mg

Mass (Tween 80) = 20 mg

Total Mass of Reactants = 311 mg

Extraction solvent (EtOAc) = 902 mg

Na_2SO_4 = 100 mg

Total Mass of Waste = 1085.3 mg

Amount of product = 227.7 mg

E-factor = (Mass of Waste)/(Mass of Product) = (1085.3) / (227.7) = 4.8

E-Factor = 4.8

Compound **3n**:

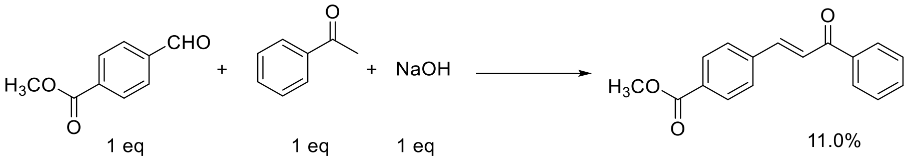

Amount of reactants:

Mass (aldehyde) = 164 mg

Mass (acetophenone) = 120 mg

Mass (NaOH) = 40 mg

Mass (Tween 80) = 20 mg

Total Mass of Reactants = 344 mg

Extraction solvent (EtOAc) = 902 mg

Na_2SO_4 = 100 mg

Total Mass of Waste = 1316.7 mg

Amount of product = 29.3 mg

E-factor = (Mass of Waste)/(Mass of Product) = (1316.7) / (29.3) = 44.9

E-Factor = 44.9

Compound **3o**:

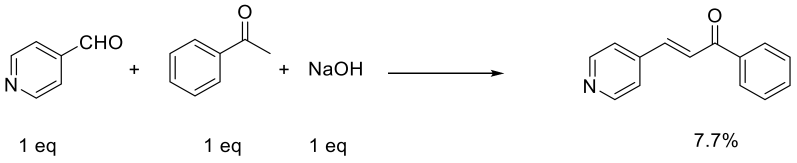

Amount of reactants:

Mass (aldehyde) = 107 mg

Mass (acetophenone) = 120 mg

Mass (NaOH) = 40 mg

Mass (Tween 80) = 20 mg

Total Mass of Reactants = 287 mg

Extraction solvent (EtOAc) = 902 mg

Na_2SO_4 = 100 mg

Total Mass of Waste = 1272.9 mg

Amount of product = 16.1 mg

E-factor = (Mass of Waste)/(Mass of Product) = (1272.9) / (16.1) = 79.1

E-Factor = 79.1

Compound **3p**:

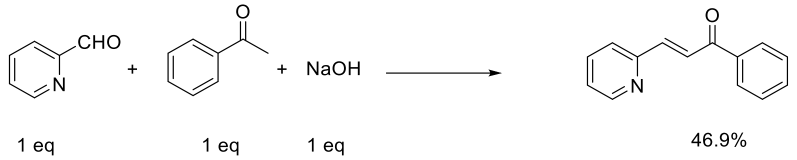

Amount of reactants:

Mass (aldehyde) = 107 mg

Mass (acetophenone) = 120 mg

Mass (NaOH) = 40 mg

Mass (Tween 80) = 20 mg

Total Mass of Reactants = 287 mg

Extraction solvent (EtOAc) = 902 mg

Na_2SO_4 = 100 mg

Total Mass of Waste = 1190.9 mg

Amount of product = 98.1 mg

E-factor = (Mass of Waste)/(Mass of Product) = (1190.9) / (98.1) = 12.1

E-Factor = 12.1

Compound **3q**:

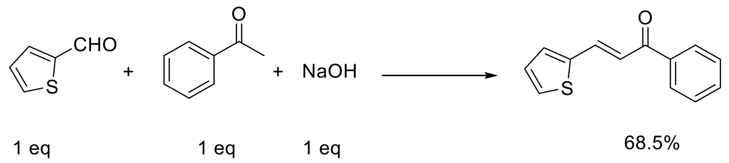

Amount of reactants:

Mass (aldehyde) = 112 mg

Mass (acetophenone) = 120 mg

Mass (NaOH) = 40 mg

Mass (Tween 80) = 20 mg

Total Mass of Reactants = 292 mg

Extraction solvent (EtOAc) = 902 mg

Na_2SO_4 = 100 mg

Total Mass of Waste = 1145.7 mg

Amount of product = 146.8 mg

E-factor = (Mass of Waste)/(Mass of Product) = (1145.7) / (146.8) = 7.8

E-Factor = 7.8

Compound **3r**:

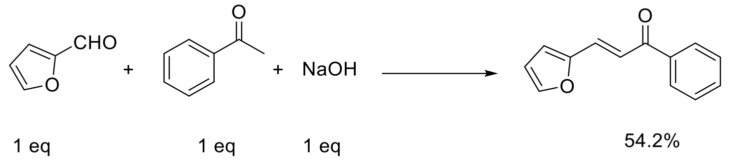

Amount of reactants:

Mass (aldehyde) = 96.1 mg

Mass (acetophenone) = 120 mg

Mass (NaOH) = 40 mg

Mass (Tween 80) = 20 mg

Total Mass of Reactants = 276.1 mg

Extraction solvent (EtOAc) = 902 mg

Na_2SO_4 = 100 mg

Total Mass of Waste = 1181.0 mg

Amount of product = 97.1 mg

E-factor = (Mass of Waste)/(Mass of Product) = (1181.0) / (97.1) = 12.2

E-Factor = 12.2

Compound **3s**:

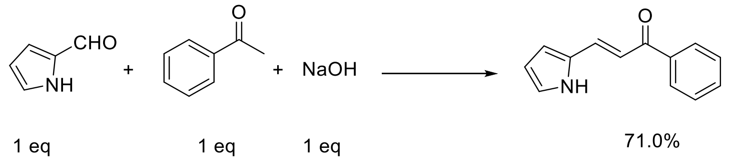

Amount of reactants:

Mass (aldehyde) = 95.1 mg

Mass (acetophenone) = 120 mg

Mass (NaOH) = 40 mg

Mass (Tween 80) = 20 mg

Total Mass of Reactants = 275.1 mg

Extraction solvent (EtOAc) = 902 mg

Na_2SO_4 = 100 mg

Total Mass of Waste = 1137.1 mg

Amount of product = 140.0 mg

E-factor = (Mass of Waste)/(Mass of Product) = (1137.1) / (140.0) = 8.1

E-Factor = 8.1

Chalcones from the Scope of the Acetophenones

Compound **6b** at 25°C:

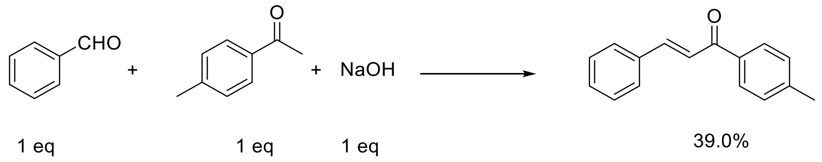

Amount of reactants:

Mass (aldehyde) = 106 mg

Mass (acetophenone) = 134 mg

Mass (NaOH) = 40 mg

Mass (Tween 80) = 20 mg

Total Mass of Reactants = 300 mg

Extraction solvent (EtOAc) = 902 mg

Na_2SO_4 = 100 mg

Total Mass of Waste = 1215.3 mg

Amount of product = 86.7 mg

E-factor = (Mass of Waste)/(Mass of Product) = (1215.3) / (86.7) = 14.0

E-Factor = 14.0

Compound **6b** at 45°C:

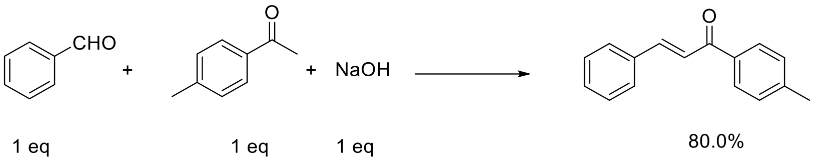

Amount of reactants:

Mass (aldehyde) = 106 mg

Mass (acetophenone) = 134 mg

Mass (NaOH) = 40 mg

Mass (Tween 80) = 20 mg

Total Mass of Reactants = 300 mg

Extraction solvent (EtOAc) = 902 mg

Na_2SO_4 = 100 mg

Total Mass of Waste = 1124.2 mg

Amount of product = 177.8 mg

E-factor = (Mass of Waste)/(Mass of Product) = (1124.2) / (177.8) = 6.3

E-Factor = 6.3

Compound **6c** at 25°C:

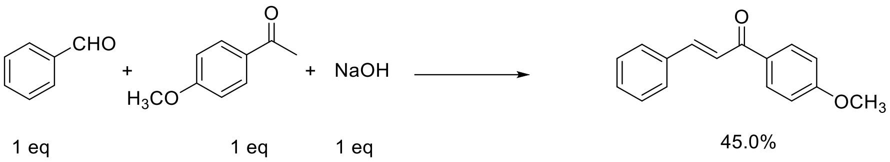

Amount of reactants:

Mass (aldehyde) = 106 mg

Mass (acetophenone) = 150 mg

Mass (NaOH) = 40 mg

Mass (Tween 80) = 20 mg

Total Mass of Reactants = 316 mg

Extraction solvent (EtOAc) = 902 mg

Na_2SO_4 = 100 mg

Total Mass of Waste = 1210.8 mg

Amount of product = 107.2 mg

E-factor = (Mass of Waste)/(Mass of Product) = (1210.8) / (107.2) = 11.3

E-Factor = 11.3

Compound **6c** at 45°C:

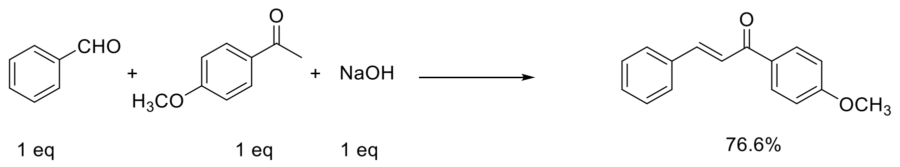

Amount of reactants:

Mass (aldehyde) = 106 mg

Mass (acetophenone) = 150 mg

Mass (NaOH) = 40 mg

Mass (Tween 80) = 20 mg

Total Mass of Reactants = 316 mg

Extraction solvent (EtOAc) = 902 mg

Na_2SO_4 = 100 mg

Total Mass of Waste = 1135.5 mg

Amount of product = 182.5 mg

E-factor = (Mass of Waste)/(Mass of Product) = (1135.5) / (182.5) = 6.2

E-Factor = 6.2

Compound **6d**:

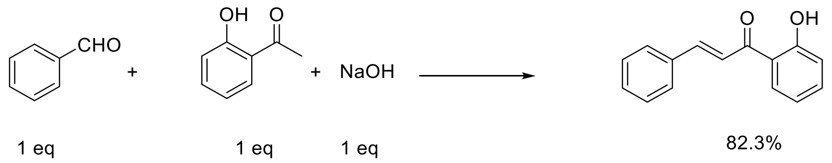

Amount of reactants:

Mass (aldehyde) = 106 mg

Mass (acetophenone) = 136 mg

Mass (NaOH) = 80 mg

Mass (Tween 80) = 20 mg

Total Mass of Reactants = 342 mg

Extraction solvent (EtOAc) = 902 mg

Na_2SO_4 = 100 mg

Total Mass of Waste = 1159.4 mg

Amount of product = 184.6 mg

E-factor = (Mass of Waste)/(Mass of Product) = (1159.4) / (184.6) = 6.3

E-Factor = 6.3

Compound **6e**:

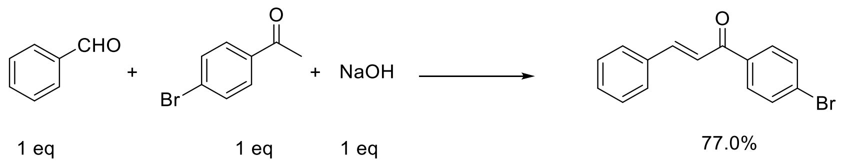

Amount of reactants:

Mass (aldehyde) = 104 mg

Mass (acetophenone) = 199 mg

Mass (NaOH) = 40 mg

Mass (Tween 80) = 20 mg

Total Mass of Reactants = 365 mg

Extraction solvent (EtOAc) = 902 mg

Na_2SO_4 = 100 mg

Total Mass of Waste = 1145.9 mg

Amount of product = 221.1 mg

E-factor = (Mass of Waste)/(Mass of Product) = (1145.9) / (221.1) = 5.2

E-Factor = 5.2

Compound **6f**:

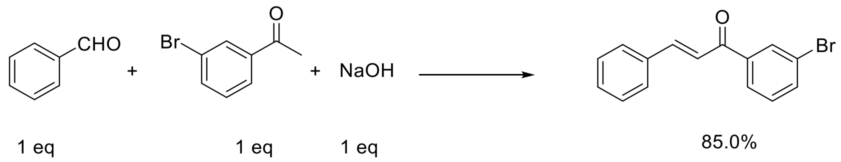

Amount of reactants:

Mass (aldehyde) = 104 mg

Mass (acetophenone) = 199 mg

Mass (NaOH) = 40 mg

Mass (Tween 80) = 20 mg

Total Mass of Reactants = 365 mg

Extraction solvent (EtOAc) = 902 mg

Na_2SO_4 = 100 mg

Total Mass of Waste = 1122.9 mg

Amount of product = 244.1 mg

E-factor = (Mass of Waste)/(Mass of Product) = (1122.9) / (244.1) = 4.6

E-Factor = 4.6

Compound **6g**:

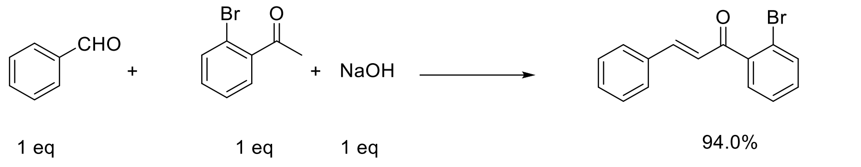

Amount of reactants:

Mass (aldehyde) = 104 mg

Mass (acetophenone) = 199 mg

Mass (NaOH) = 40 mg

Mass (Tween 80) = 20 mg

Total Mass of Reactants = 365 mg

Extraction solvent (EtOAc) = 902 mg

Na_2SO_4 = 100 mg

Total Mass of Waste = 1098.5 mg

Amount of product = 268.5 mg

E-factor = (Mass of Waste)/(Mass of Product) = (1098.5) / (268.5) = 4.1

E-Factor = 4.1

Compound **6h**:

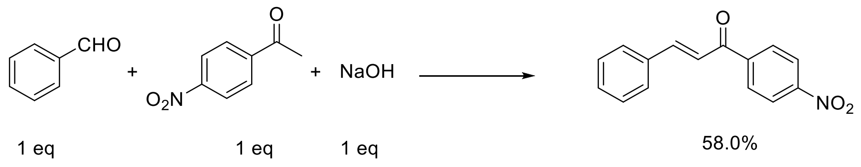

Amount of reactants:

Mass (aldehyde) = 106 mg

Mass (acetophenone) = 165 mg

Mass (NaOH) = 40 mg

Mass (Tween 80) = 20 mg

Total Mass of Reactants = 331 mg

Extraction solvent (EtOAc) = 902 mg

Na_2SO_4 = 100 mg

Total Mass of Waste = 1186.1 mg

Amount of product = 146.9 mg

E-factor = (Mass of Waste)/(Mass of Product) = (1186.1) / (146.9) = 8.1

E-Factor = 8.1

Compound **6i**:

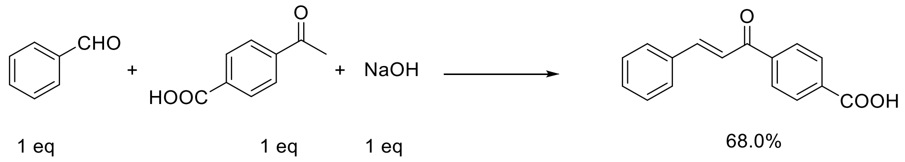

Amount of reactants:

Mass (aldehyde) = 106 mg

Mass (acetophenone) = 164 mg

Mass (NaOH) = 40 mg

Mass (Tween 80) = 20 mg

Total Mass of Reactants = 330 mg

Extraction solvent (EtOAc) = 902 mg

Na_2SO_4 = 100 mg

Total Mass of Waste = 1160.5 mg

Amount of product = 171.5 mg

E-factor = (Mass of Waste)/(Mass of Product) = (1160.5) / (171.5) = 6.8

E-Factor = 6.8

Compound **6j**:

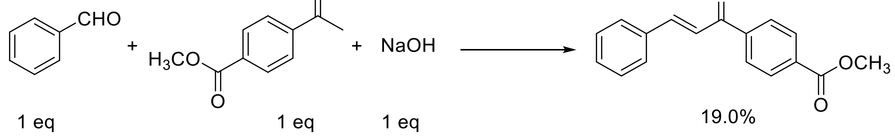

Amount of reactants:

Mass (aldehyde) = 106 mg

Mass (acetophenone) = 178 mg

Mass (NaOH) = 40 mg

Mass (Tween 80) = 20 mg

Total Mass of Reactants = 344 mg

Extraction solvent (EtOAc) = 902 mg

Na_2SO_4 = 100 mg

Total Mass of Waste = 1295.4 mg

Amount of product = 50.6 mg

E-factor = (Mass of Waste)/(Mass of Product) = (1295.4) / (50.6) = 25.6

E-Factor = 25.6

Compound **6k**:

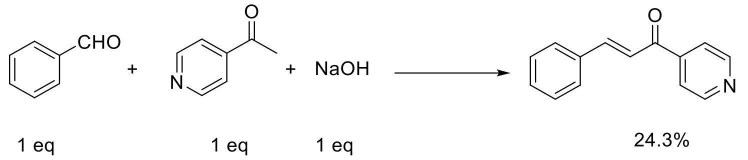

Amount of reactants:

Mass (aldehyde) = 104 mg

Mass (acetophenone) = 121 mg

Mass (NaOH) = 40 mg

Mass (Tween 80) = 20 mg

Total Mass of Reactants = 287 mg

Extraction solvent (EtOAc) = 902 mg

Na_2SO_4 = 100 mg

Total Mass of Waste = 1238.2 mg

Amount of product = 50.8 mg

E-factor = (Mass of Waste)/(Mass of Product) = (1238.2) / (50.8) = 24.4

E-Factor = 24.4

Compound **6l**:

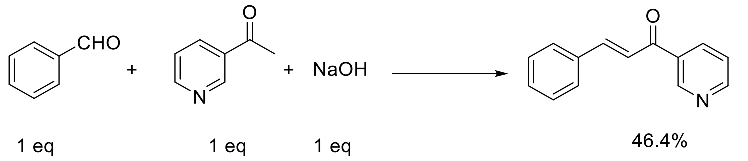

Amount of reactants:

Mass (aldehyde) = 104 mg

Mass (acetophenone) = 121 mg

Mass (NaOH) = 40 mg

Mass (Tween 80) = 20 mg

Total Mass of Reactants = 287 mg

Extraction solvent (EtOAc) = 902 mg

Na_2SO_4 = 100 mg

Total Mass of Waste = 1191.9 mg

Amount of product = 97.1 mg

E-factor = (Mass of Waste)/(Mass of Product) = (1191.9) / (97.1) = 12.3

E-Factor = 12.3

Compound **6m**:

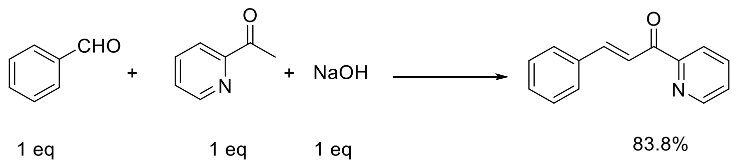

Amount of reactants:

Mass (aldehyde) = 104 mg

Mass (acetophenone) = 121 mg

Mass (NaOH) = 40 mg

Mass (Tween 80) = 20 mg

Total Mass of Reactants = 287 mg

Extraction solvent (EtOAc) = 902 mg

Na_2SO_4 = 100 mg

Total Mass of Waste = 1113.6 mg

Amount of product = 175.4 mg

E-factor = (Mass of Waste)/(Mass of Product) = (1113.6) / (175.4) = 6.4

E-Factor = 6.4

Compound **6n**:

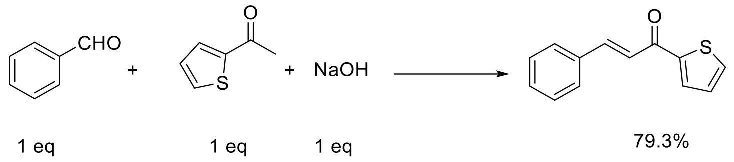

Amount of reactants:

Mass (aldehyde) = 106 mg

Mass (acetophenone) = 126 mg

Mass (NaOH) = 40 mg

Mass (Tween 80) = 20 mg

Total Mass of Reactants = 292 mg

Extraction solvent (EtOAc) = 902 mg

Na_2SO_4 = 100 mg

Total Mass of Waste = 1124.1 mg

Amount of product = 169.9 mg

E-factor = (Mass of Waste)/(Mass of Product) = (1124.1) / (169.9) = 6.6

E-Factor = 6.6

Compound **6o**:

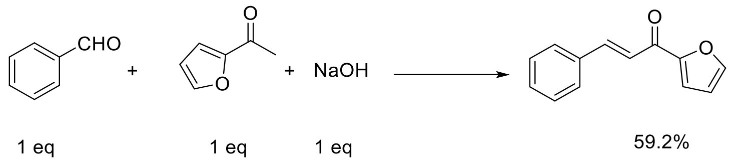

Amount of reactants:

Mass (aldehyde) = 106 mg

Mass (acetophenone) = 110 mg

Mass (NaOH) = 40 mg

Mass (Tween 80) = 20 mg

Total Mass of Reactants = 276 mg

Extraction solvent (EtOAc) = 902 mg

Na_2SO_4 = 100 mg

Total Mass of Waste = 1160.7 mg

Amount of product = 117.3 mg

E-factor = (Mass of Waste)/(Mass of Product) = (1160.7) / (117.3) = 9.9

E-Factor = 9.9

Compound **6p**:

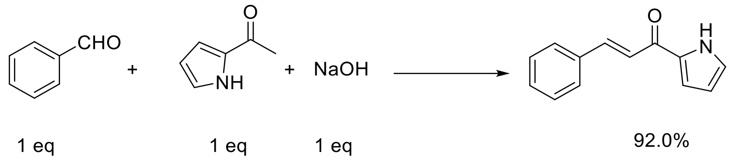

Amount of reactants:

Mass (aldehyde) = 106 mg

Mass (acetophenone) = 109 mg

Mass (NaOH) = 40 mg

Mass (Tween 80) = 20 mg

Total Mass of Reactants = 275 mg

Extraction solvent (EtOAc) = 902 mg

Na_2SO_4 = 100 mg

Total Mass of Waste = 1105.4 mg

Amount of product = 171.6 mg

E-factor = (Mass of Waste)/(Mass of Product) = (1105.4) / (171.6) = 6.4

E-Factor = 6.4

Representative Chalcones

Compound **9**:

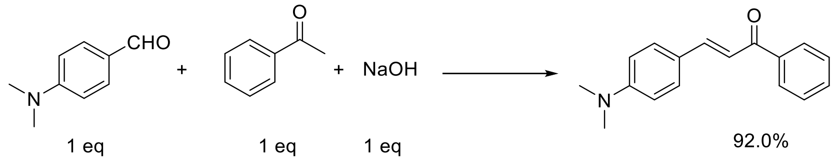

Amount of reactants:

Mass (aldehyde) = 149 mg

Mass (acetophenone) = 120 mg

Mass (NaOH) = 40 mg

Mass (Tween 80) = 20 mg

Total Mass of Reactants = 329 mg

Extraction solvent (EtOAc) = 902 mg

Na_2SO_4 = 100 mg

Total Mass of Waste = 1322.8 mg

Amount of product = 8.8 mg

E-factor = (Mass of Waste)/(Mass of Product) = (1322.8) / (8.8) = 150.3

E-Factor = 150.3

Compound **10** at 25°C:

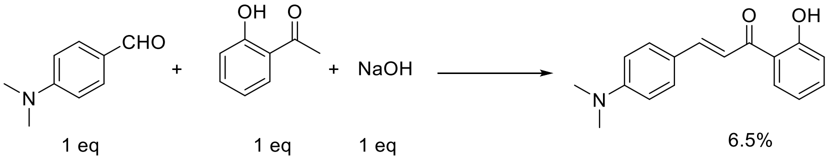

Amount of reactants:

Mass (aldehyde) = 149 mg

Mass (acetophenone) = 134 mg

Mass (NaOH) = 80 mg

Mass (Tween 80) = 20 mg

Total Mass of Reactants = 383 mg

Extraction solvent (EtOAc) = 902 mg

Na_2SO_4 = 100 mg

Total Mass of Waste = 1369.9 mg

Amount of product = 17.4 mg

E-factor = (Mass of Waste)/(Mass of Product) = (1443.1) / (17.4) = 78.6

E-Factor = 78.6

Compound **10** at 45°C:

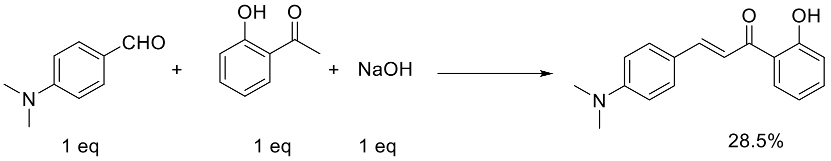

Amount of reactants:

Mass (aldehyde) = 149 mg

Mass (acetophenone) = 134 mg

Mass (NaOH) = 160 mg

Mass (Tween 80) = 20 mg

Total Mass of Reactants = 455 mg

Extraction solvent (EtOAc) = 902 mg

Na_2SO_4 = 100 mg

Total Mass of Waste = 1390.8.3

Amount of product = 76.2 mg

E-factor = (Mass of Waste)/(Mass of Product) = (1390.8) / (76.2) = 18.3

E-Factor = 18.3

Compound **11** at 25°C:

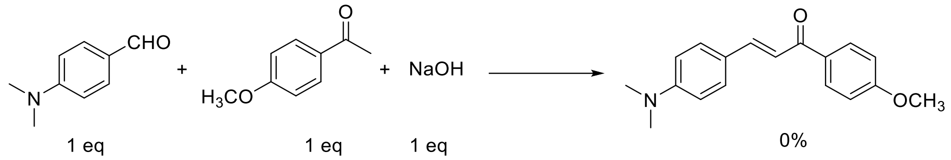

Amount of reactants:

Mass (aldehyde) = 149 mg

Mass (acetophenone) = 150 mg

Mass (NaOH) = 40 mg

Mass (Tween 80) = 20 mg

Total Mass of Reactants = 359 mg

Extraction solvent (EtOAc) = 902 mg

Na_2SO_4 = 100 mg

Total Mass of Waste = 1391.9 mg

Amount of product = 0 mg

E-factor = (Mass of Waste)/(Mass of Product) = (1391.9) / (0) = -

E-Factor = -

Amount of product = 0 mg

E-Factor = -

Compound **11** at 45°C:

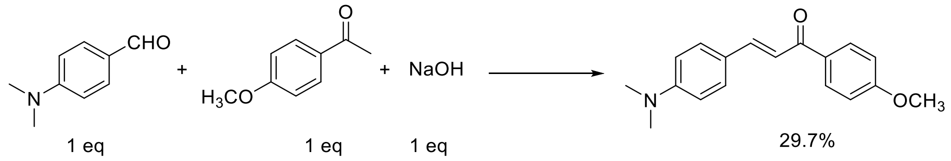

Amount of reactants:

Mass (aldehyde) = 149 mg

Mass (acetophenone) = 150 mg

Mass (NaOH) = 40 mg

Mass (Tween 80) = 20 mg

Total Mass of Reactants = 359 mg

Extraction solvent (EtOAc) = 902 mg

Na_2SO_4 = 100 mg

Total Mass of Waste = 1391.9 mg

Amount of product = 82.3 mg

E-factor = (Mass of Waste)/(Mass of Product) = (1391.9) / (82.3) = 16.9

E-Factor = 16.9

Compound **12**:

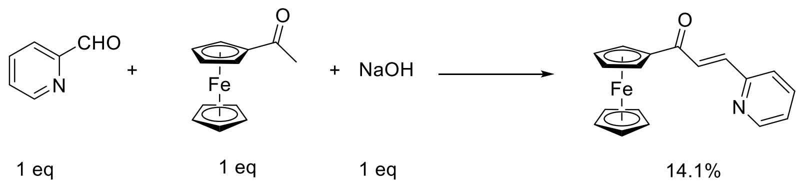

Amount of reactants:

Mass (aldehyde) = 107 mg

Mass (acetophenone) = 228 mg

Mass (NaOH) = 40 mg

Mass (Tween 80) = 20 mg

Total Mass of Reactants = 395 mg

Extraction solvent (EtOAc) = 902 mg

Na_2SO_4 = 100 mg

Total Mass of Waste = 1352.3 mg

Amount of product = 44.7 mg

E-factor = (Mass of Waste)/(Mass of Product) = (1352.3) / (44.7) = 30.3

E-Factor = 30.3

Compound **13** at 25°C:

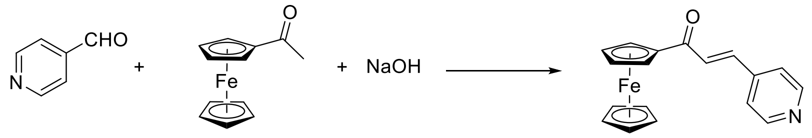

1 eq 1 eq 1 eq 12.1%

Amount of reactants:

Mass (aldehyde) = 107 mg

Mass (acetophenone) = 228 mg

Mass (NaOH) = 40 mg

Mass (Tween 80) = 20 mg

Total Mass of Reactants = 395 mg

Extraction solvent (EtOAc) = 902 mg

Na_2SO_4 = 100 mg

Total Mass of Waste = 1358.6 mg

Amount of product = 38.4 mg

E-factor = (Mass of Waste)/(Mass of Product) = (1358.6) / (38.4) = 35.4

E-Factor = 35.4

Compound **13** at 25°C:

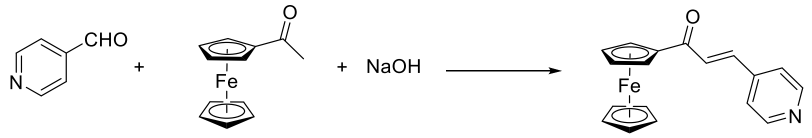

1 eq 1 eq 1 eq 12.1%

Amount of reactants:

Mass (aldehyde) = 107 mg

Mass (acetophenone) = 228 mg

Mass (NaOH) = 40 mg

Mass (Tween 80) = 20 mg

Total Mass of Reactants = 395 mg

Extraction solvent (EtOAc) = 902 mg

Na_2SO_4 = 100 mg

Total Mass of Waste = 1358.6 mg

Amount of product = 38.4 mg

E-factor = (Mass of Waste)/(Mass of Product) = (1358.6) / (38.4) = 35.4

E-Factor = 35.4

Compound **13** at 45°C:

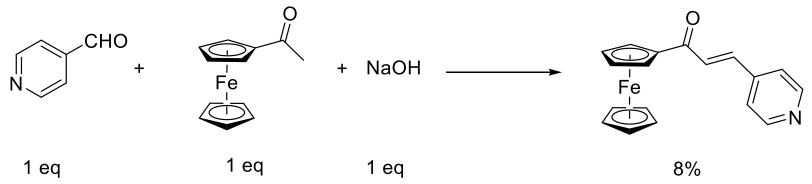

Amount of reactants:

Mass (aldehyde) = 107 mg

Mass (acetophenone) = 228 mg

Mass (NaOH) = 40 mg

Mass (Tween 80) = 20 mg

Total Mass of Reactants = 395 mg

Extraction solvent (EtOAc) = 902 mg

Na_2SO_4 = 100 mg

Total Mass of organic waste = 1358.6 mg

Amount of product = 38.4 mg

E-factor = (Mass of Organic Waste)/(Mass of Product) = (1358.6) / (38.4) = 35.4

E-Factor = 35.4

Compound **14**:

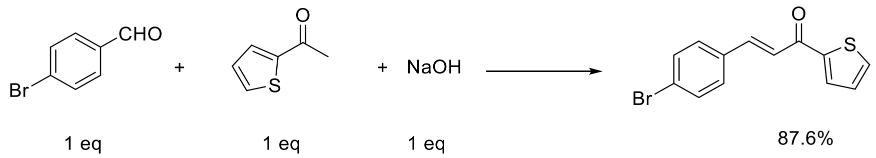

Amount of reactants:

Mass (aldehyde) = 185 mg

Mass (acetophenone) = 126 mg

Mass (NaOH) = 40 mg

Mass (Tween 80) = 20 mg

Total Mass of Reactants = 371 mg

Extraction solvent (EtOAc) = 902 mg

Na_2SO_4 = 100 mg

Total Mass of Waste = 1116.2 mg

Amount of product = 256.8 mg

E-factor = (Mass of Waste)/(Mass of Product) = (1116.2) / (256.8) = 4.3

E-Factor = 4.3

Compound **15**:

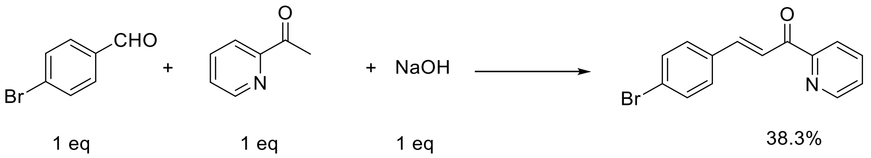

Amount of reactants:

Mass (aldehyde) = 185 mg

Mass (acetophenone) = 121 mg

Mass (NaOH) = 40 mg

Mass (Tween 80) = 20 mg

Total Mass of Reactants = 366 mg

Extraction solvent (EtOAc) = 902 mg

Na_2SO_4 = 100 mg

Total Mass of Waste = 1257.6 mg

Amount of product = 110.4 mg

E-factor = (Mass of Waste)/(Mass of Product) = (1257.6) / (110.4) = 11.4

E-Factor = 11.4

Compound **16**:

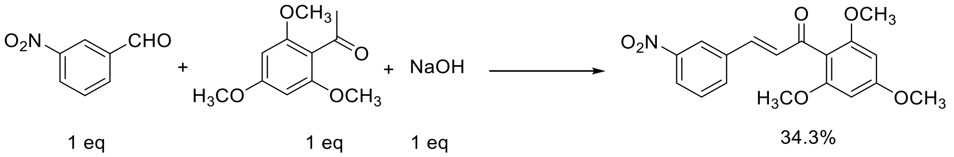

Amount of reactants:

Mass (aldehyde) = 151 mg

Mass (acetophenone) = 210 mg

Mass (NaOH) = 40 mg

Mass (Tween 80) = 20 mg

Total Mass of Reactants = 421 mg

Extraction solvent (EtOAc) = 902 mg

Na_2SO_4 = 100 mg

Total Mass of Waste = 1305.2 mg

Amount of product = 117.8 mg

E-factor = (Mass of Waste)/(Mass of Product) = (1305.2) / (117.8) = 11.1

E-Factor = 11.1

Compound **17**:

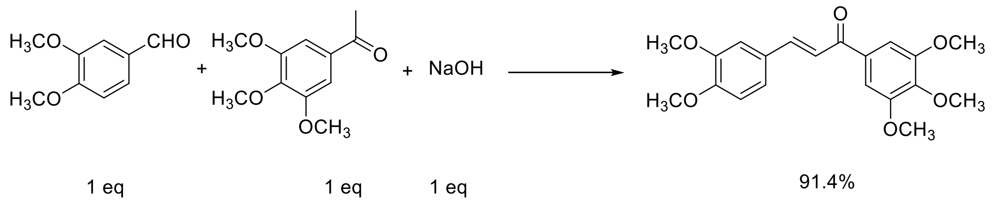

Amount of reactants:

Mass (aldehyde) = 196 mg

Mass (acetophenone) = 180 mg

Mass (NaOH) = 40 mg

Mass (Tween 80) = 20 mg

Total Mass of Reactants = 436 mg

Extraction solvent (EtOAc) = 902 mg

Na_2SO_4 = 100 mg

Total Mass of Waste = 1115.8 mg

Amount of product = 327.6 mg

E-factor = (Mass of Waste)/(Mass of Product) = (1115.8) / (327.6) = 3.4

E-Factor = 3.4

E-factor from recycling experiments

Compound **3h** in CTAB:

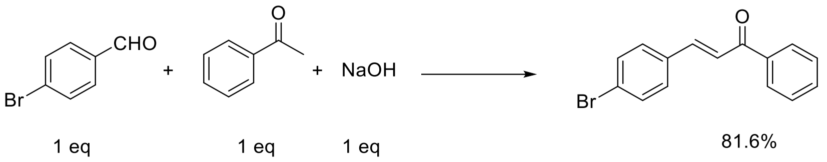

Amount of reactants:

Mass (aldehyde) = 925 mg

Mass (acetophenone) = 601 mg

Mass (NaOH) = 200 mg

Mass (CTAB) = 100 mg

Total Mass of Reactants = 1.826 g

Extraction solvent (MTBE) = 3.700 g

Na_2SO_4 = 500 mg

Total Mass of Waste = 4.856 g

Amount of product = 1.170 g

E-factor = (Mass of Waste)/(Mass of Product) = (4.856) / (1.170) = 4.2

E-Factor = 4.2

PMI: 5.2

Compound **3h** in Tween 80 at 45°C:

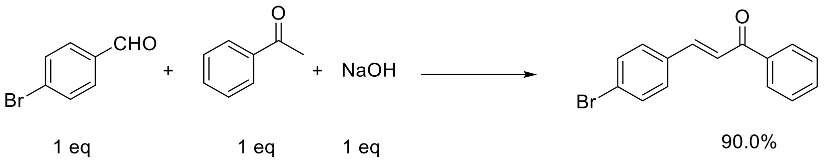

Amount of reactants:

Mass (aldehyde) = 985 mg

Mass (acetophenone) = 601 mg

Mass (NaOH) = 200 mg

Mass (Tween 80) = 100 mg

Total Mass of Reactants = 365 mg

Extraction solvent (EtOAc) = 902 mg

Na_2SO_4 = 100 mg

Total Mass of Waste = 4.747 g

Amount of product = 1.279 g

E-factor = (Mass of Waste)/(Mass of Product) = (4.747) / (1.279) = 3.7

E-Factor = 3.7

PMI: 4.7

Table S1 - E-Factor calculated for the chalcones obtained from the scope of the aldehydes.

Surf.	3a EtOH	3a	3b	3c	3c	3d	3d	3e	3f	3f	3g	3h	3h	3i	3j	3j	3k	3k	3l	3m	3n	3o	3p	3q	3r	3s
					45°C		45°C			45°C			45°C			45°C		45°C								
	15.7																									
CTAB		8.6	5.4	6.3	-[a]	5.7	-[a]	8.3	4.6	-[a]	15.2	4.8	-[a]	4.3	7.7	-[a]	6.7	6.2	6.5	7.5	17	-[b]	43.3	20.2	10.9	19.6
Tween 80		6.6	5.0	15.3	7.3	15.3	7.3	12.8	7.5	4.4	21.4	14.9	4.3	5.3	6.0	4.7	5.1	-[a]	-[b]	4.8	44.9	79.1	12.1	7.8	12.2	8.1

[a] Not available since the reaction was not performed (See the table reporting the yields in the paper); [b] the yield is 0 and the E-Factor cannot be determined.

Table S2 - E-Factor calculated for the chalcones obtained from the scope of the acetophenones.

Surf.	3a EtOH	3a	6b	6b	6c	6c	6d	6e	6f	6g	6h	6i	6j	6k	6l	6m	6n	6o	6p
				45°C		45°C													
	15.7																		
CTAB		8.6	6.1	-[a]	7.1	-[a]	37.8	5.8	6.1	5.0	19.3	6i	32.7	-[b]	-[b]	27.8	19.2	15.9	6.0
Tween 80		6.6	14.0	6.3	11.3	6.2	6.3	5.2	4.6	4.1	8.1	6.8	25.6	24.4	12.3	6.4	6.6	9.9	6.4

[a] Not available since the reaction was not performed (See the table reporting the yields in the paper); [b] the yield is 0 and the E-Factor cannot be determined.

Table S3 - E-Factor calculated for the representative chalcones.

Surf.	9	10	10	11	11	12	13	13	14	15	16	17
			45°C		45°C			45°C				
CTAB	7.4	542.3	20.6	15	15.4	9.0	18.4	28.5	5.5	5.7	7.1	4.8
Tween 80	150.3	78.7	18.2	-[a]	18.2	30.3	35.4	54	4.3	11.4	11.1	3.4

[a] The yield is 0 and the E-Factor cannot be determined.

Process Mass Intensity calculation

In Tables S4, S5, S6, S7, S8, S9 are reported the PMI values calculated by taking into consideration all the reactants, water and solvents used for the reaction and during the quenching.

The amounts of reactants used for the reaction and the solvent extraction are those reported in the section “E factor calculation”.

Table S4 - PMI calculations for the chalcones obtained from the scope of the aldehydes in CTAB.

Compound	3a EtOH	3a	3b	3c	3d	3e	3f	3g	3h	3i	3j	3k	3k	3l	3m	3n	3o	3p	3q	3r	3s
													45°C								
**E-Factor**	**15.7**	**8.6**	**5.4**	**6.3**	**5.7**	**8.3**	**4.6**	**15.2**	**4.8**	**4.3**	**7.7**	**6.7**	**6.2**	**6.5**	**7.5**	**17**	**-[a]**	**43.3**	**20.2**	**10.9**	**19.6**
**PMI**	**25.7**	**17.2**	**11.2**	**12.9**	**11.9**	**16.4**	**9.7**	**28.2**	**10.1**	**9.2**	**15.1**	**13.5**	**12.6**	**13.2**	**14.9**	**31.4**	**-[a]**	**78.7**	**37.7**	**21.2**	**36.8**
**PMI Substrate, Reagents, Solvents**	**17.6**	**9.6**	**6.4**	**7.3**	**6.7**	**9.3**	**5.6**	**16.2**	**5.8**	**5.3**	**8.7**	**7.7**	**7.2**	**7.5**	**8.5**	**18.0**	**-[a]**	**44.3**	**21.2**	**11.9**	**20.6**
**PMI Substrates and Reagents**	**3.8**	**2.9**	**2.1**	**2.2**	**2.1**	**2.9**	**1.9**	**5.3**	**2.0**	**1.8**	**3.0**	**2.5**	**2.3**	**2.4**	**2.7**	**6.0**	**-[a]**	**13.3**	**6.4**	**3.5**	**6.1**
**PMI Solvents**	**13.8**	**6.8**	**4.3**	**5.1**	**4.6**	**6.4**	**3.7**	**10.8**	**3.8**	**3.5**	**5.8**	**5.2**	**4.9**	**5.1**	**5.8**	**12.1**	**-[a]**	**31.0**	**14.8**	**8.4**	**14.6**
**PMI Water**	**8.1**	**7.5**	**4.8**	**5.6**	**5.1**	**7.1**	**4.2**	**12.0**	**4.3**	**3.9**	**6.4**	**5.8**	**5.4**	**5.7**	**6.5**	**13.4**	**-[a]**	**34.4**	**16.4**	**9.3**	**16.2**

[a] The yield is 0 and the E-Factor cannot be determined.

Table S5 - PMI calculations for the chalcones obtained from the scope of the aldehydes in Tween 80.

Surf.	3a EtOH	3a	3b	3c	3d	3d	3e	3f	3f	3g	3h	3h	3i	3j	3j	3k	3l	3m	3n	3o	3p	3q	3r	3s
						45°C			45°C			45°C			45°C									
**E-Factor**	**25.7**	**6.6**	**5.0**	**6.5**	**15.3**	**7.3**	**12.8**	**7.5**	**4.4**	**21.4**	**14.9**	**4.3**	**5.3**	**6.0**	**4.7**	**5.1**	**-[a]**	**4.8**	**44.9**	**79.1**	**12.1**	**7.8**	**12.2**	**8.1**
**PMI**	**17.6**	**13.5**	**10.4**	**13.3**	**28.6**	**14.7**	**24.3**	**14.8**	**9.4**	**39.1**	**27.5**	**9.2**	**10.9**	**12.1**	**9.8**	**10.6**	**-[a]**	**10.2**	**80.1**	**142.2**	**23.3**	**15.6**	**23.5**	**16.3**

**PMI Substrate, Reagents, Solvents**	**3.8**	**7.6**	**6.0**	**7.5**	**16.3**	**8.3**	**13.8**	**8.5**	**5.4**	**22.4**	**15.9**	**5.3**	**6.3**	**7.0**	**5.7**	**6.1**	**-[a]**	**5.8**	**45.9**	**80.1**	**13.1**	**8.8**	**13.2**	**9.1**
**PMI Substrates and Reagents**	**13.8**	**2.3**	**2.0**	**2.3**	**5.1**	**2.6**	**4.4**	**2.8**	**1.8**	**7.4**	**5.4**	**1.8**	**2.1**	**2.4**	**1.9**	**2.0**	**-[a]**	**1.8**	**15.2**	**24.0**	**3.9**	**2.7**	**3.9**	**2.7**
**PMI Solvents**	**8.1**	**5.3**	**4.0**	**5.2**	**11.1**	**5.7**	**9.5**	**5.7**	**3.6**	**15.1**	**10.5**	**3.5**	**4.1**	**4.6**	**3.7**	**4.1**	**-[a]**	**4.0**	**30.8**	**56.0**	**9.2**	**6.1**	**9.3**	**6.4**
**PMI Water**	**25.7**	**5.9**	**4.5**	**5.8**	**12.3**	**6.3**	**10.5**	**6.3**	**4.0**	**16.7**	**11.6**	**3.9**	**4.6**	**5.1**	**4.1**	**4.5**	**-[a]**	**4.4**	**34.1**	**62.1**	**10.2**	**6.8**	**10.3**	**7.1**

[a] The yield is 0 and the E-Factor cannot be determined.

Table S6 - PMI calculations for the chalcones obtained from the scope of the acetophenones in CTAB.

**Compound**	**3a**	**6b**	**6c**	**6d**	**6e**	**6f**	**6g**	**6h**	**6i**	**6j**	**6k**	**6l**	**6m**	**6n**	**6o**	**6p**
**E-Factor**	**8.6**	**6.1**	**7.1**	**37.8**	**5.8**	**6.1**	**5.0**	**19.3**	**6i**	**32.7**	**-[a]**	**-[a]**	**27.8**	**19.2**	**15.9**	**6.0**
**PMI**	**17.2**	**12.4**	**14.3**	**68.6**	**11.7**	**12.3**	**10.3**	**35.5**	**16.8**	**58.8**	**-[a]**	**-[a]**	**51.1**	**35.8**	**30.1**	**12.5**
**PMI Substrate, Reagents, Solvents**	**9.6**	**7.1**	**8.1**	**38.8**	**6.8**	**7.1**	**6.0**	**20.3**	**9.6**	**33.7**	**-[a]**	**-[a]**	**28.8**	**20.2**	**16.9**	**7.0**
**PMI Substrates and Reagents**	**2.9**	**2.1**	**2.6**	**12.0**	**2.3**	**2.4**	**2.0**	**6.6**	**3.1**	**11.1**	**-[a]**	**-[a]**	**8.6**	**6.1**	**5.0**	**2.1**
**PMI Solvents**	**6.8**	**4.8**	**5.6**	**26.8**	**4.5**	**4.7**	**3.9**	**13.7**	**6.5**	**22.6**	**-[a]**	**-[a]**	**20.1**	**14.1**	**11.9**	**5.0**
**PMI Water**	**7.5**	**5.4**	**6.2**	**29.8**	**4.9**	**5.2**	**4.4**	**15.2**	**7.2**	**25.1**	**-[a]**	**-[a]**	**22.3**	**15.6**	**13.2**	**5.5**

[a] The yield is 0 and the E-Factor cannot be determined.

Table S7 - E-Factor calculations for the chalcones obtained from the scope of the acetophenones in Tween 80.

**Surf.**	**3a**	**6b**	**6b**	**6c**	**6c**	**6d**	**6e**	**6f**	**6g**	**6h**	**6i**	**6j**	**6k**	**6l**	**6m**	**6n**	**6o**	**6p**
			**45°C**		**45°C**													
**Tween 80**	**6.6**	**14.0**	**6.3**	**11.3**	**6.2**	**6.3**	**5.2**	**4.6**	**4.1**	**8.1**	**6.8**	**25.6**	**24.4**	**12.3**	**6.4**	**6.6**	**9.9**	**6.4**

**PMI**	**13.5**	**26.6**	**12.9**	**21.6**	**12.7**	**12.7**	**10.7**	**9.7**	**8.8**	**15.9**	**13.6**	**46.4**	**45.1**	**23.6**	**13.1**	**13.5**	**19.4**	**13.3**
**PMI Substrate, Reagents, Solvents**	**7.6**	**15.0**	**7.3**	**12.3**	**7.2**	**7.3**	**6.2**	**5.6**	**5.1**	**9.1**	**7.8**	**26.6**	**25.4**	**13.3**	**7.3**	**7.6**	**10.9**	**7.4**
**PMI Substrates and Reagents**	**2.3**	**4.6**	**2.2**	**3.9**	**2.3**	**2.4**	**2.1**	**1.9**	**1.7**	**2.9**	**2.5**	**8.8**	**7.6**	**4.0**	**2.2**	**2.3**	**3.2**	**2.2**
**PMI Solvents**	**5.3**	**10.4**	**5.1**	**8.4**	**4.9**	**4.9**	**4.1**	**3.7**	**3.4**	**6.1**	**5.3**	**17.8**	**17.8**	**9.3**	**5.1**	**5.3**	**7.7**	**5.3**
**PMI Water**	**5.9**	**11.5**	**5.6**	**9.3**	**5.5**	**5.4**	**4.5**	**4.1**	**3.7**	**6.8**	**5.8**	**19.8**	**19.7**	**10.3**	**5.7**	**5.9**	**8.5**	**5.8**

Table S8 - PMI calculations for the representative chalcones obtained in CTAB.

**Compound**	**9**	**10**	**10**	**11**	**11**	**12**	**13**	**13**	**14**	**15**	**16**	**17**
			**45°C**		**45°C**			**45°C**				
**E-Factor**	**7.4**	**542.3**	**20.6**	**15**	**15.2**	**9.0**	**18.4**	**28.5**	**5.5**	**5.7**	**7.1**	**4.8**
**PMI**	**14.7**	**913.7**	**36.3**	**29.2**	**28.2**	**17.0**	**33.3**	**50.7**	**11.2**	**11.6**	**13.7**	**9.9**
**PMI Substrate, Reagents, Solvents**	**8.4**	**543.3**	**21.6**	**17.4**	**16.3**	**9.9**	**19.4**	**29.5**	**6.5**	**6.7**	**8.1**	**5.8**
**PMI Substrates and Reagents**	**2.7**	**209.3**	**8.3**	**6.8**	**5.5**	**3.5**	**6.9**	**10.5**	**2.2**	**2.3**	**3.0**	**2.2**
**PMI Solvents**	**5.7**	**334.1**	**13.3**	**10.6**	**10.8**	**6.4**	**12.5**	**19.1**	**4.3**	**4.4**	**5.1**	**3.7**
**PMI Water**	**6.3**	**370.4**	**14.7**	**11.8**	**12.0**	**7.1**	**13.9**	**21.1**	**4.7**	**4.9**	**5.7**	**4.1**

Table S9 - PMI calculations for the representative chalcones obtained in Tween 80.

**Compound**	**9**	**10**	**10**	**11**	**11**	**12**	**13**	**13**	**14**	**15**	**16**	**17**
			**45°C**		**45°C**			**45°C**				

**E-Factor**	**150.3**	**78.7**	**18.3**	**-[a]**	**18.2**	**30.3**	**35.4**	**94.4**	**4.3**	**11.4**	**11.1**	**3.4**
**PMI**	**264.9**	**137.2**	**32.4**	**-[a]**	**33.3**	**17.0**	**62.4**	**55.0**	**9.2**	**21.4**	**20.6**	**7.4**
**PMI Substrate, Reagents, Solvents**	**151.3**	**79.7**	**19.3**	**-[a]**	**19.2**	**9.9**	**36.4**	**19.5**	**5.3**	**12.4**	**12.1**	**4.4**
**PMI Substrates and Reagents**	**48.8**	**27.9**	**7.4**	**-[a]**	**6.5**	**3.5**	**12.9**	**35.5**	**1.8**	**4.2**	**4.4**	**1.6**
**PMI Solvents**	**102.5**	**51.8**	**11.8**	**-[a]**	**12.7**	**6.4**	**23.5**	**39.4**	**3.5**	**8.2**	**7.7**	**2.8**
**PMI Water**	**113.6**	**57.5**	**13.1**	**-[a]**	**14.1**	**7.1**	**26.0**	**94.4**	**3.9**	**9.1**	**8.5**	**3.1**

[a] The yield is 0 and the E-Factor and PMI cannot be determined.

Scale-up for Aldehydes

Table S10 - Scope of aldehydes in a scale-up to 5 mmol, in CTAB 2%.

Entry	Aldehyde	Yield (%)[a]			
1	H	**3a**	87	**4a**	5
2	4-AcNH	**3b**	90	**4b**	2
3	4-CH_3	**3c**	93	**4c**	4
4	4-OCH_3	**3d**	90	**4d**	7
5	2-OCH_3	**3e**	79	**4e**	19.4
6	2,6-diOCH_3	**3f**	97.0	**4f**	0
7	4-OH	**3g**	36	**4g**	1
8	4-Br	**3h**	81	**4h**	3
9	3-Br	**3i**	83	**4i**	4
10	2-Br	**3j**	64	**4j**	15
11	4-NO_2	**3k**	53	**4k**	11
12	CN	**3l**	77	**4l**	7
13	COOH	**3m**	56	**4m**	0
14	4-CHO-Pyr	**3o**	0	**4o**	52
15	2-CHO-Pyr	**3p**	69	**4p**	0
16	2-CHO-Th	**3q**	95	**4q**	2
17	2-CHO-Fur	**3r**	99	**4r**	0
18	2-CHO-Pir	**3s**	55	**4s**	0

Reaction conditions: 5 mL vial, benzaldehyde (5 mmol), Acetophenone (5 mmol), NaOH (base) (5 eq.); surfactant solution (5 mL), 25°C, 24h. [a] Isolated yield.

Further Material and Discussions

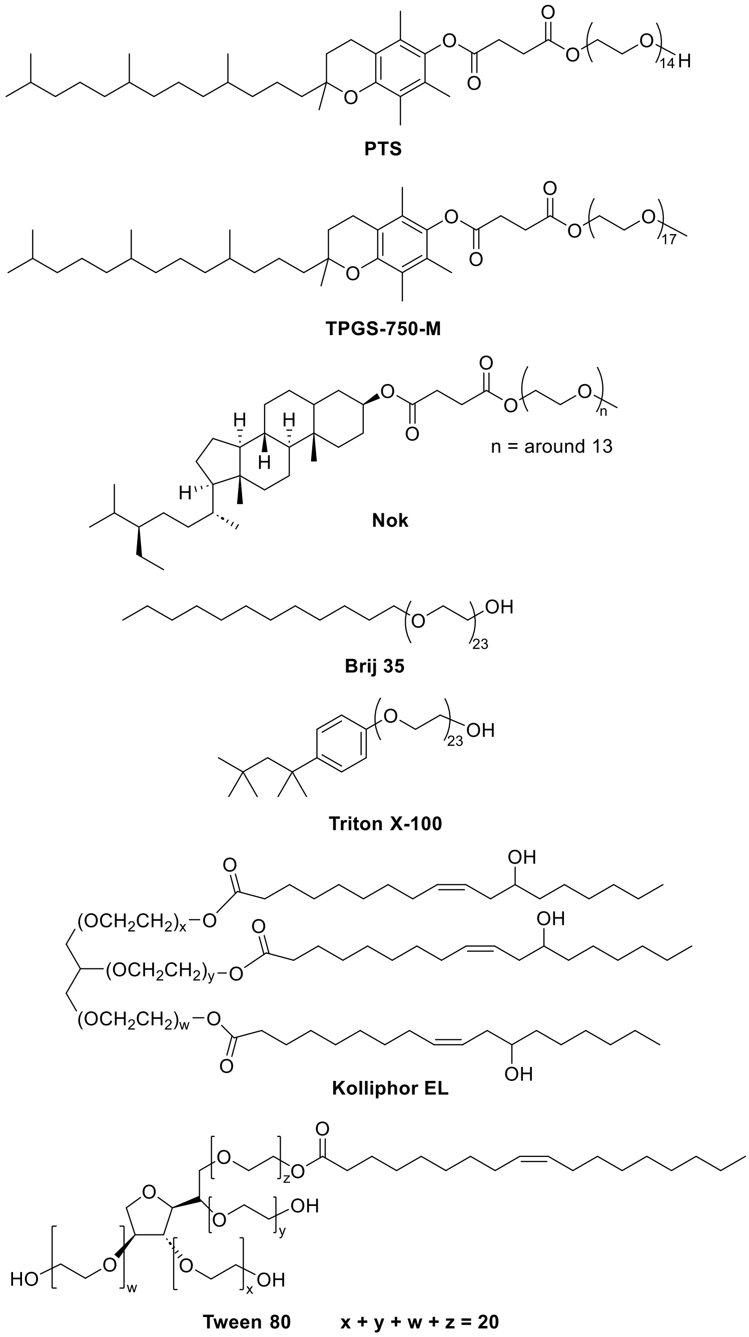

Figure S1 - Structures of TPGS, PTS, Nok, Brij 35, Triton X-100, Kolliphor EL, and Tween 80 surfactants, used in this study.

Structure of side products

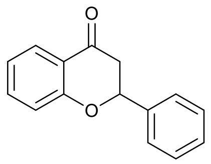

Figure S2 - Flavanone (8d) is produced as a byproduct during the reaction of 2-hydroxyacetophenone with benzaldehyde.

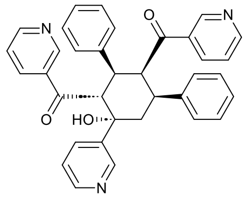

Figure S3 – The represented 3:2 adduct is the hypothesized product obtained from the reaction of 3-acetylpyridine with benzaldehyde. The product could not be isolated since very difficult to purify.

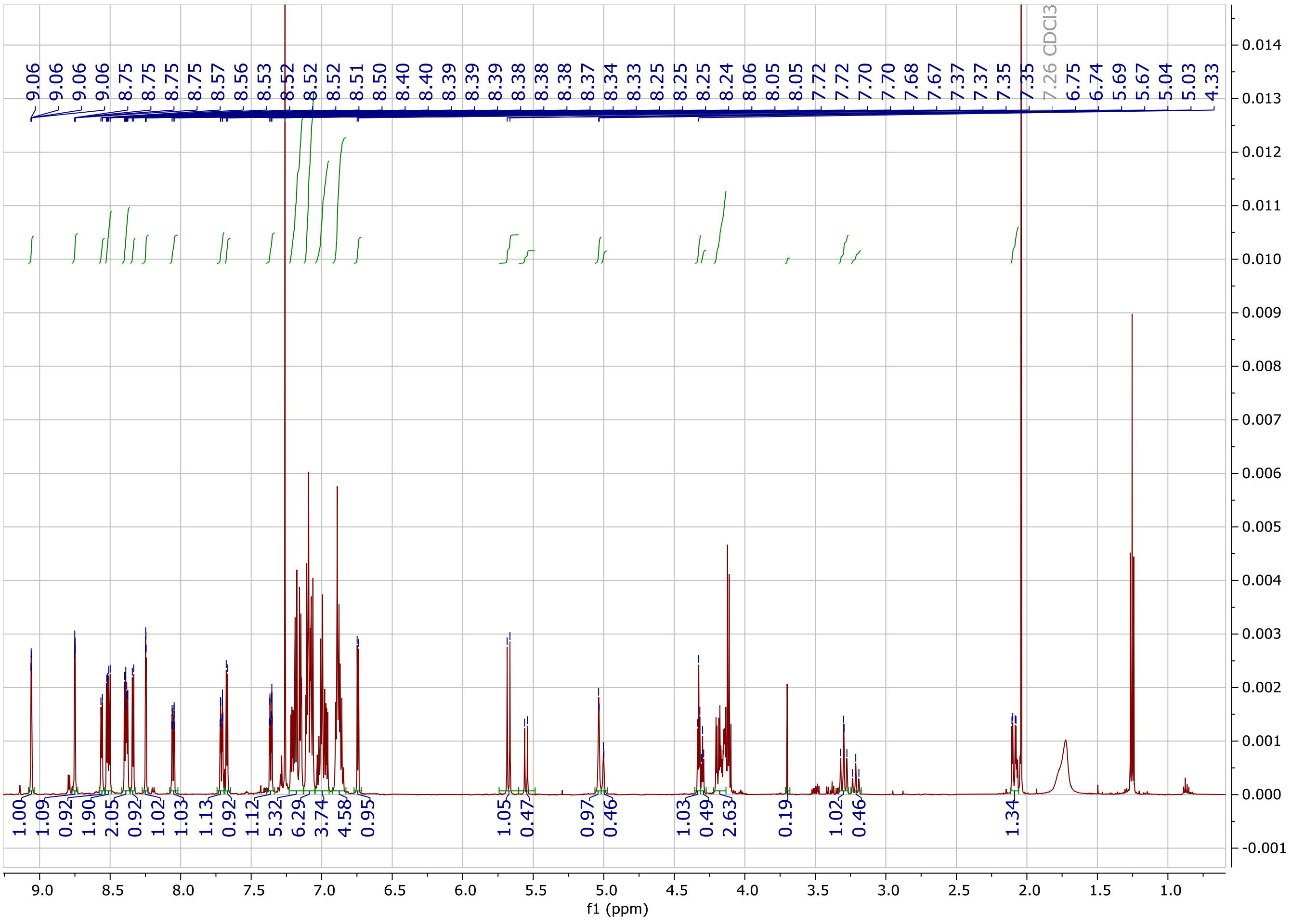

Figure S4 – 1-H NMR (600 MHz) for the crude of the reaction whose product can tentatively proposed as that represented in Fig. S3, or one of its stereoisomers.

Tentative identification of a side-product (Fig S3) by NMR of the reaction crude.

The crude was difficult to be purified and the isolation of the compopund was outside the scope of the work. However, in a paper by Constable et al,[80] the formation of similar compounds is very well described and demonstrated. The pattern of most peaks found in the crude is very similar to the one found in the cited

paper,[80] and peaks in the aliphatic region support the formation of the cyclohexanol. The appearance of similar peaks in the same region, e.g. two doublets at 5.68 and 5.55 ppm, and singlets at 5.03 and 5.00 ppm, support the hypothesis of the formation of two regioisomers of the cyclohexanol (see the reference,[80] for more details). The peaks in the aromatic region closely resembles the pattern found by Constable et al. for a similar compound where the two phenyl rings were substituted in para position with propoxy groups. Besides, for a very similar compound obtained from 4-acetylpyridine and benzaldehyde, most of the spectrum is very similar to the peaks of the crude we obtained.[81] All this information let to propose a structure like that represented in Figure S3 (or one stereoisomer of it) as the possible product, while difficult to purify.

Optimization of the base concentration

Because Tween 80 is considered a green surfactant being a food-grade emulsifier ingredient (under the code E433),[82] and SDS is a well-established industrial surfactant, we considered them good options to CTAB and tried to optimize the NaOH concentration for all these three surfactants at 2% concentration (Table 3). No variation was observed for CTAB; at the contrary, in the case of Tween 80 and SDS a decreasing of yield was observed if NaOH concentration varies from 1 to 0.5 equivalents. The further decreasing of NaOH concentration with Tween 80 determined an increasing formation of aldol derivative **5a**. This means that the base concentration influences the rate of the transformation of the aldol into chalcone, in agreement to literature.[83] The dehydration step has been identified as the slower step for the formation of chalcones in organic solvents,[84] and this seems to apply also to the experimental results of our study in micellar solution.

The results reported in Table S11 suggested that the optimal quantity of the base is 1 equivalent. The study was thus continued with only CTAB and Tween 80 which was the best performing non-ionic surfactant. SDS promoted the reaction showing a counterintuitive interesting behaviour, but a specific study should be devoted to it.

Table S11 - Optimization of the base (NaOH) concentration for the chalcone synthesis (1 mmol scale).

Entry	Surfactant (2%)	Base (mmol)	Yield (%)[a]		
			**3a**	**4a**	**5a**
1	CTAB	1	65	9	0
2	CTAB (5 x CMC)	0.5	66	9	0
3	SDS	1	83	0	0
4	SDS	0.5	75	0	0
5	Tween 80	1	85	2	0
6	Tween 80	0.5	81	0	0
7[b]	Tween 80	0.25	38	0	27
8[b]	Tween 80	0.1	18	0	37

Reaction conditions: 5 ml vial, benzaldehyde (1 mmol), acetophenone (1 mmol), NaOH (base) (0.1-1.0 eq.); surfactant solution (1 ml), rt, 24 h. [a] Calculated by NMR with heptane as internal standard; [b] aldol was detected.

Characterization of the micellar solutions

DLS Measurements

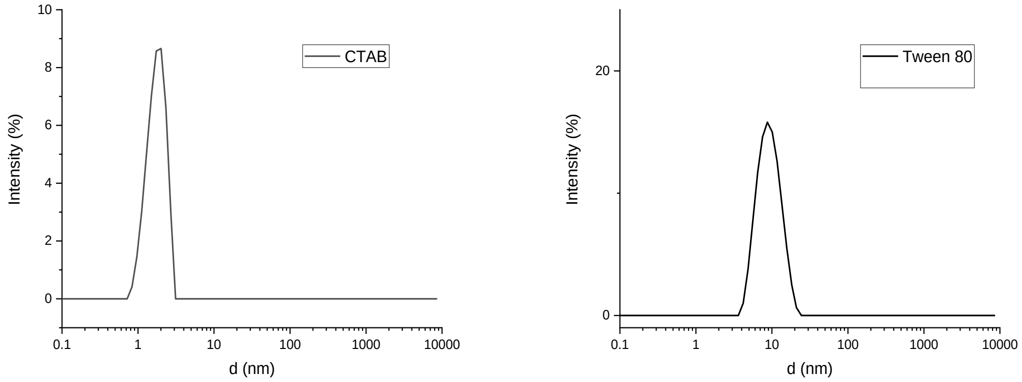

Figure S5 - Dynamic Light Scattering plots for CTAB 2% (up) and Tween 80 2% (down).

NMR characterization: chemical shift

The NMR spectrum can reveal information about the micelles and its interaction with solutes.[85, 86] In the past, the micellization was studied by the variation of the surfactant's protons chemical shifts and by the widening of the proton NMR signals.[87]

Recently, the Diffusion Ordered SpectroscopY (DOSY) technique was used to estimate the diffusion coefficient of micelles and the binding percentage of solutes to the micelles.[88] We used some of these features to study the interaction of benzaldehyde and acetophenone with CTAB and Tween 80 micellar solutions.

A first analysis was performed on the chemical shifts of the surfactants, upon micellization. NMR spectra of benzaldehyde and acetophenone were recorded in D_2O.

We analysed: i) the chemical shifts of ^{1}H-NMR spectra of CTAB and Tween 80 in D_2O alone or in presence of benzaldehyde and acetophenone, at different surfactant concentrations above and below the CMC; ii) the chemical shifts of ^{1}H-NMR spectra of benzaldehyde and acetophenone in D_2O without surfactant and at surfactant different concentrations above and below the CMC; iii) the chemical shifts of acetophenone and CTAB or Tween 80 ^{1}H-NMR spectra in presence of one equivalent of NaOD. The data are reported in Tables S12-S15.

CTAB

A monomeric CTAB solution (0.5 mM < CMC (0.9 mM) gives a well resolved spectrum, adopted as a reference for chemical shift of the different signals. Upon micellization all signals are deshielded and widened. When the CTAB concentration is further increased the protons are further deshielded but less than when micellization starts.

When the benzaldehyde is added into each CTAB solution its protons were consistently deshielded with respect to the benzaldehyde alone in D_2O. The CHO proton was the most affected one, *e.g.* with a deshielding effect of 1.15 ppm in the case of 2 % CTAB concentration.

Similar but less pronounced deshielding effect has been observed for aromatic protons, in particular for ortho (D = 0.38 ppm) and para (D = 0.33 ppm) protons in comparison to meta one (D = 0.05 ppm). This suggests an increasing of the carbonyl bond polarization due to the internalization of benzaldehyde into the micelle and to the specific interaction with the positive charge of the surfactant headgroup. Consequentially, the electrophilicity of the carbonyl carbon increases explaining why the reaction occurs better in CTAB than in only water.

In the same conditions, a limited shielding shift was observed for CTAB protons (around 0.01-0.04 ppm), due to its interaction with the benzaldehyde and its diamagnetic anisotropy effect, depending on the benzaldehyde/CTAB ratio: slightly larger at 5 mM and 10 mM than at 54.8 mM. The shielding effect decreases with the distance of the methylenes of the chain from the charged headgroup: the main affected signals are the N^+CH_3 and the N^+CH_2, $N^+CH_2CH_2$ and $N^+CH_2CH_2CH_2CH_2$ protons.

As far as the acetophenone and CTAB solution interaction is concerned similar effects were observed with comparable shielding shifts for CTAB protons but only slight minimal shifts for the aromatic protons of acetophenone; the methyl group of acetophenone was substantially unaffected.

Table S12 - Chemical shifts of protons of micellized CTAB upon benzaldehyde addition, in D_2O.

[CTAB]	Benzaldehyde				CTAB					
(mM)	CHO	H_{ortho}	H_{para}	H_{meta}	$N^+\underline{CH_2}$	$N^+\underline{CH_3}$	$N^+CH_2\underline{CH_2}$	$N^+CH_2CH_2\underline{CH_2}CH_2$	$-(\underline{CH_2})_{11}-$	$\underline{CH_3}$
0	8.92	7.63	7.32	7.59						
0.5					3.35	3.13	1.78	1.37	1.29	0.87
5	9.97	7.97	7.76	7.63	3.39	3.17	1.76	1.35	1.30	0.89
					3.42	3.19	1.79	1.39	1.32	0.90
	1.05 [a]	0.34 [a]	0.34 [a]	0.04 [a]	-0.03 [b]	-0.02 [b]	-0.03 [b]	-0.04 [b]	-0.02 [b]	-0.01 [b]
10	9.98	7.98	7.76	7.63	3.42	3.19	1.78	1.38	1.31	0.90
					3.44	3.20	1.80	1.40	1.32	0.90
	1.06 [a]	0.35 [a]	0.34 [a]	0.04 [a]	-0.02 [b]	-0.01 [b]	-0.02 [b]	-0.02 [b]	-0.01 [b]	0.00 [b]
54.8 (2%)	10.07	8.01	7.75	7.64	3.46	3.21	1.79	1.39	1.32	0.90
					3.47	3.22	1.81	1.41	1.33	0.90
	1.15 [a]	0.38 [a]	0.33 [a]	0.05 [a]	-0.01 [b]	-0.01 [b]	-0.02 [b]	-0.02 [b]	-0.01 [b]	0.00 [b]

[a] δ of benzaldehyde protons in CTAB solution at specific concentration - δ of benzaldhyde alone; [b] δ of CTAB protons at specific concentration in presence of benzaldehyde - δ of CTAB protons at specific concentration without benzaldehyde.

Table S13 - Chemical shifts of protons of micellized CTAB upon acetophenone addition, in D_2O.

[CTAB] (mM)	Acetophenone H_{ortho}	H_{para}	H_{meta}	$COCH_3$	CTAB N^+CH_2	N^+CH_3	$N^+CH_2CH_2$	$N^+CH_2CH_2CH_2CH_2$	$-(CH_2)_{11}-$	CH_3
0	**8.02**	**7.70**	**7.57**	**2.68**						
0.5					**3.35**	**3.13**	**1.78**	**1.37**	**1.29**	**0.87**
5	**8.02**	**7.69**	**7.57**	**2.68**	**3.39**	**3.17**	**1.76**	**1.35**	**1.30**	**0.89**
					**3.42**	**3.19**	**1.79**	**1.39**	**1.32**	**0.90**
	**0.00**[a]	**-0.01**[a]	**0.00**[a]	**0.00**[a]	**-0.03**[b]	**-0.02**[b]	**-0.03**[b]	**-0.04**[b]	**-0.02**[b]	**-0.01**[b]
10	**8.02**	**7.68**	**7.55**	**2.67**	**3.40**	**3.17**	**1.76**	**1.36**	**1.29**	**0.89**
					**3.44**	**3.20**	**1.80**	**1.40**	**1.32**	**0.9**
	**0.00**[a]	**-0.02**[a]	**-0.02**[a]	**-0.01**[a]	**-0.04**[b]	**-0.03**[b]	**-0.02**[b]	**-0.04**[b]	**-0.03**[b]	**-0.01**[b]
10 (+NaOD)	**8.01**	**7.67**	**7.55**	**-**[c]	**3.38**	**3.16**	**1.75**	**1.34**	**1.28**	**0.88**
	**-0.01**[a]	**-0.03**[a]	**-0.02**[a]	**-** [a,c]	**-0.06**[b]	**-0.04**[b]	**-0.04**[b]	**-0.04**[b]	**-0.04**[b]	**-0.02**[b]
54.8 (2%)	**8.05**	**7.67**	**7.56**	**2.69**	**3.45**	**3.20**	**1.79**	**1.39**	**1.31**	**0.90**
					**3.47**	**3.22**	**1.81**	**1.41**	**1.33**	**0.90**
	**0.03**[a]	**-0.03**[a]	**-0.01**[a]	**0.01**[a]	**-0.02**[b]	**-0.02**[b]	**-0.02**[b]	**-0.02**[b]	**-0.02**[b]	**0.00**[b]

[a] δ of acetophenone protons in CTAB solution at specific concentration - δ of acetophenone alone; [b] δ of CTAB protons at specific concentration in presence of acetophenone - δ of CTAB protons at specific concentration without acetophenone; [c] upon addition of NaOD, the methyl groups of acetophenone disappears completely.

When one equivalent of NaOD was added with respect to the acetophenone, the disappearance of the methyl ketone signal at around 2.6 ppm showed that the acetophenone enolate was formed. Consistently, the acetophenone ortho and para protons were slightly shielded while the meta protons were unaffected.

A slight additional shielding effect was observed for the CTAB protons suggesting that a stronger interaction of the enolate with CTAB becomes by coulombic interactions. In particular, the N^+CH2 first methylene and the N^+CH_3 methyl groups showed a D = -0.06 and -0.04 ppm shielding effect, respectively.

The present analysis of ^1H-NMR supports the idea that in CTAB solution the reaction can take place at the micellar surface with the cationic groups that promote the reaction by attracting benzaldehyde and enolate keeping them closer and favouring the reaction in a sort of organocatalytic phenomenon.

Tween 80

Analogously to the CTAB case, monomeric Tween 80 solution (0.005 mM < Tween 80 CMC (0.010-0.015 mM[89-91]) gave a well resolved spectrum that was the reference spectrum to evaluate the variation of chemical shift of the different signals of Tween 80 owing to micellization. The chemical shifts of Tween 80 at different concentrations (below and above the CMC) are reported in Tables S14 and S15. The Tween 80 micellization caused a slight deshielding effect also for some protons of Tween 80.

Table S14 - Chemical shifts of protons of micellized Tween 80 upon benzaldehyde addition, in D_2O.

[T80] (mM)	Benzaldeyde CHO	H_{ortho}	H_{para}	H_{meta}	Tween 80 CH=	OCH$OCH_2\underline{CH_2CO}$-	$O\underline{CH_2}CH_2\underline{C}O$-	CO-$\underline{CH_2}$	$\underline{CH_2}$-CH=	$COCH_2\underline{CH_2}$	$(CH_2)_{15}$	CH_3
0	**8.92**	**7.63**	**7.32**	**7.59**								
0.5					**5.32**	**4.20**	**3.70**	**2.31**	**2.01**	**1.58**	**1.31-1.28**	**0.88**
5	**9.95**	**7.95**	**7.77**	**7.62**	**5.32**	**4.20**	**3.70**	**2.30**	**2.01**	**1.57**	**1.28**	**0.88**
					**5.33**	**4.21**	**3.70**	**2.32**	**2.02**	**1.59**	**1.32-1.29**	**0.89**
	**1.03[a]**	**0.32[a]**	**0.45[a]**	**0.03[a]**	**-0.0[b]**	**-0.0[b]**	**0.00[b]**	**-0.02[b]**	**-0.01[b]**	**-0.02[b]**	**-0.04[b]**	**-0.01[b]**
10	**9.94**	**7.95**	**7.74**	**7.61**	**5.31**	**4.19**	**3.69**	**2.30**	**2.00**	**1.57**	**1.27**	**0.87**
					**5.33**	**4.21**	**3.70**	**2.32**	**2.02**	**1.59**	**1.32-1.29**	**0.89**
	**1.02[a]**	**0.32[a]**	**0.42[a]**	**0.02[a]**	**-0.02[b]**	**-0.02[b]**	**-0.01[b]**	**-0.02[b]**	**-0.02[b]**	**-0.02[b]**	**-0.05[b]**	**-0.02[b]**
15 (2%)	**9.95**	**7.95**	**7.73**	**7.61**	**5.31**	**4.20**	**3.69**	**2.30**	**2.00**	**1.57**	**1.28**	**0.87**
					**5.33**	**4.21**	**3.70**	**2.32**	**2.02**	**1.59**	**1.32-1.29**	**0.89**
	**1.03[a]**	**0.32[a]**	**0.41[a]**	**0.02[a]**	**-0.02[b]**	**-0.01[b]**	**-0.01[b]**	**-0.02[b]**	**-0.02[b]**	**-0.02[b]**	**-0.04[b]**	**-0.02[b]**

[a] δ of benzaldehyde protons in Tween 80 solution at specific concentration - δ of benzaldehyde alone; [b] δ of Tween 80 protons at specific concentration in presence of benzaldehyde - δ of Tween 80 protons at specific concentration without benzaldehyde.

Table S15 - Chemical shifts of protons of micellized Tween 80 upon acetophenone addition, in D_2O.

[T80] (mM)	Acetophenone H_{ortho}	H_{para}	H_{meta}	$COCH_3$	Tween 80 CH=	$OCH_2\underline{CH_2}CO$-	$\underline{OCH_2}$ $\underline{CH_2}$CO-	CO-$\underline{CH_2}$	$\underline{CH_2}$-CH=	$COCH_2\underline{CH_2}$	$(CH_2)_{15}$	CH_3
0	**8.02**	**7.70**	**7.57**	**2.68**								
0.5					**5.32**	**4.20**	**3.70**	**2.31**	**2.01**	**1.58**	**1.31-1.28**	**0.88**
5	**8.01**	**7.68**	**7.56**	**2.66**	**5.32**	**4.20**	**3.70**	**2.30**	**2.01**	**1.57**	**1.28**	**0.88**
					**5.33**	**4.21**	**3.70**	**2.32**	**2.02**	**1.59**	**1.32-1.29**	**0.89**
	**-0.01[a]**	**-0.02[a]**	**-0.01[a]**	**-0.02[a]**	**-0.01[b]**	**-0.01[b]**	**0.00[b]**	**-0.02[b]**	**-0.01[b]**	**-0.02[b]**	**-0.04[b]**	**-0.01[b]**
10	**7.99**	**7.66**	**7.55**	**2.65**	**5.31**	**4.19**	**3.69**	**2.30**	**2.00**	**1.56**	**1.27**	**0.87**
					**5.33**	**4.21**	**3.70**	**2.32**	**2.02**	**1.59**	**1.32-1.29**	**0.89**
	**-0.03[a]**	**-0.04[a]**	**-0.02[a]**	**-0.03[a]**	**-0.02[b]**	**-0.02[b]**	**-0.01[b]**	**-0.02[b]**	**-0.02[b]**	**-0.03[b]**	**-0.05[b]**	**-0.02[b]**
10[c]	**7.98**	**7.65**	**7.52**	**-[d]**	**5.30**	**4.18**	**3.68**	**2.29**	**2.00**	**1.55**	**1.26**	**0.86**
	**-0.04[a]**	**-0.05[a]**	**-0.05[a]**	**-[a, d]**	**-0.03[b]**	**-0.03[b]**	**-0.02[b]**	**-0.03[b]**	**-0.02[b]**	**-0.04[b]**	**-0.06[b]**	**-0.03[b]**
15 (2%)	**8**	**7.66**	**7.54**	**2.65**	**5.32**	**4.20**	**3.70**	**2.30**	**2.01**	**1.57**	**1.27**	**0.88**
					**5.33**	**4.21**	**3.70**	**2.32**	**2.02**	**1.59**	**1.32-1.29**	**0.89**
	**-0.02[a]**	**-0.04[a]**	**-0.03[a]**	**-0.03[a]**	**-0.01[b]**	**-0.01[b]**	**0.00[b]**	**-0.02[b]**	**-0.01[b]**	**-0.02[b]**	**-0.05[b]**	**-0.01[b]**

[a] δ of acetophenone protons in Tween 80 solution at specific concentration - δ of acetophenone alone; [b] δ of Tween 80 protons at specific concentration in presence of acetophenone - δ of Tween 80 protons at specific concentration without acetophenone; [c] NaOD was added; [d] upon addition of NaOD, the methyl groups of acetophenone disappears completely.

The signals referred to the ethylene oxide and sorbate portions are nearly collapsed into a one huge and large peak, so any effect on them is lost by the averaging of the multitude of overlapped signals. Some effects are appreciable for the hydrophobic chain, even though the magnitude of the effect is very small, about D = 0.01-0.02 ppm, while becomes more appreciable in the case of the 10 mM concentration.

As seen in the CTAB case, the interaction of benzaldehyde with Tween 80 caused deshielding effect, stronger for CHO proton and ortho and para-aromatic protons and lower for the meta protons (Table S14). At high concentration of Tween 80 solution the deshielding effect of the CHO proton is lower of about 0.1 ppm than in CTAB solution.

The chemical shift of the benzaldehyde CHO was reported in several solvents.[92] The chemical shift of benzaldehyde CHO in Tween 80 was similar to its value reported in apolar solvents as CCl_4 and CS_2 (9.95 ppm[92]), while in CTAB it appeared to be similar to values recorded in more polar solvents as acetonitrile and benzonitrile (10.04 and 10.09 ppm, respectively[92]).

While in CTAB the deshielding effect can be attributed mainly to the interaction of the carbonyl oxygen with cationic CTAB headgroup, the deshielding effect in Tween 80 can be attributed to the substantial dehydration of the CHO group of benzaldehyde which is transferred into the more hydrophobic core of micelles.

The chemical shift can thus give hints to evaluate the polarity of the micellar site in which the benzaldehyde is localised. This is consistent with a location of benzaldehyde at the micellar surface or first micellar palisade layer for CTAB and in a more internal region for Tween 80.

Consistently, the Tween 80 protons were more shielded due to the interaction with the benzaldehyde aromatic ring than those of the alkyl chain (D = -0.04 ppm), and the first methylene of the ester group., i.e. the -OCH_2CH_2O-CO-R protons. As consequence of the interaction of the aromatic ring with the more internal methylenes of the chain, the broad singlet (large and with no apparent components in micelles of Tween 80 alone), is splitted in two signals and some methylenes were shielded by about 0.04 – 0.05 ppm. This could lead to suppose that the solubilization site of benzaldehyde is close to the ester group, probably with its carbonyl group, while the aromatic ring protrudes towards the hydrophobic core, affecting the 3rd / 4th methylenes of hydrophobic chain which showed a shielding effect. This is substantially in agreement with the observation of the aldehyde proton chemical shift, discussed above, where a substantially apolar region was estimated as the solubilization site for benzaldehyde in Tween 80.

Differently from the case of CTAB, the acetophenone protons (Table S15) were affected by the interaction with Tween 80 micelles showing in general shielded shifts. Methylenes of the alkyl chain of Tween 80 showed shielding effect comparable to benzaldehyde case suggesting that also the acetophenone is probably located deeper into the micelle.

The formation of enolate is confirmed when NaOD was added from the general slight shielding effect of aromatic protons (-0.04/-0.05 ppm). As in CTAB solution, the acetophenone methyl signal disappeared. A largest deshielding effect on the Tween 80 protons was observed for the 2nd, 3rd and 4th methylene groups of the alkyl chain, for the alkene protons and the terminal CH_3, in agreement to localization of enolate in the hydrophobic core of micelles.

Further attempts to detect the interaction of reactants with both CTAB and Tween 80 by NOESY/ROESY were not conclusive.

NMR Characterization: DOSY

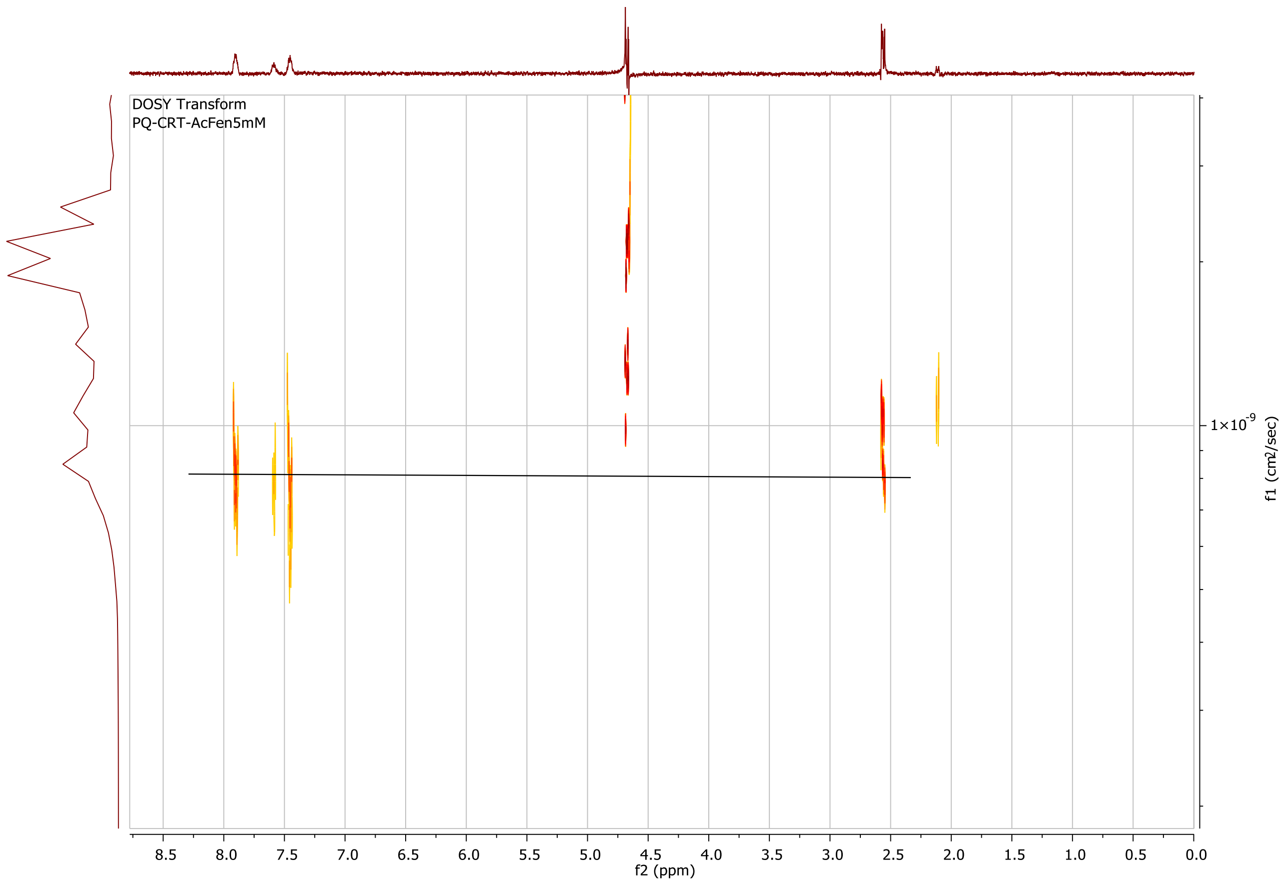

Figure S6 - Example of the DOSY plot for the Acetophenone (5 mM) in D_2O.

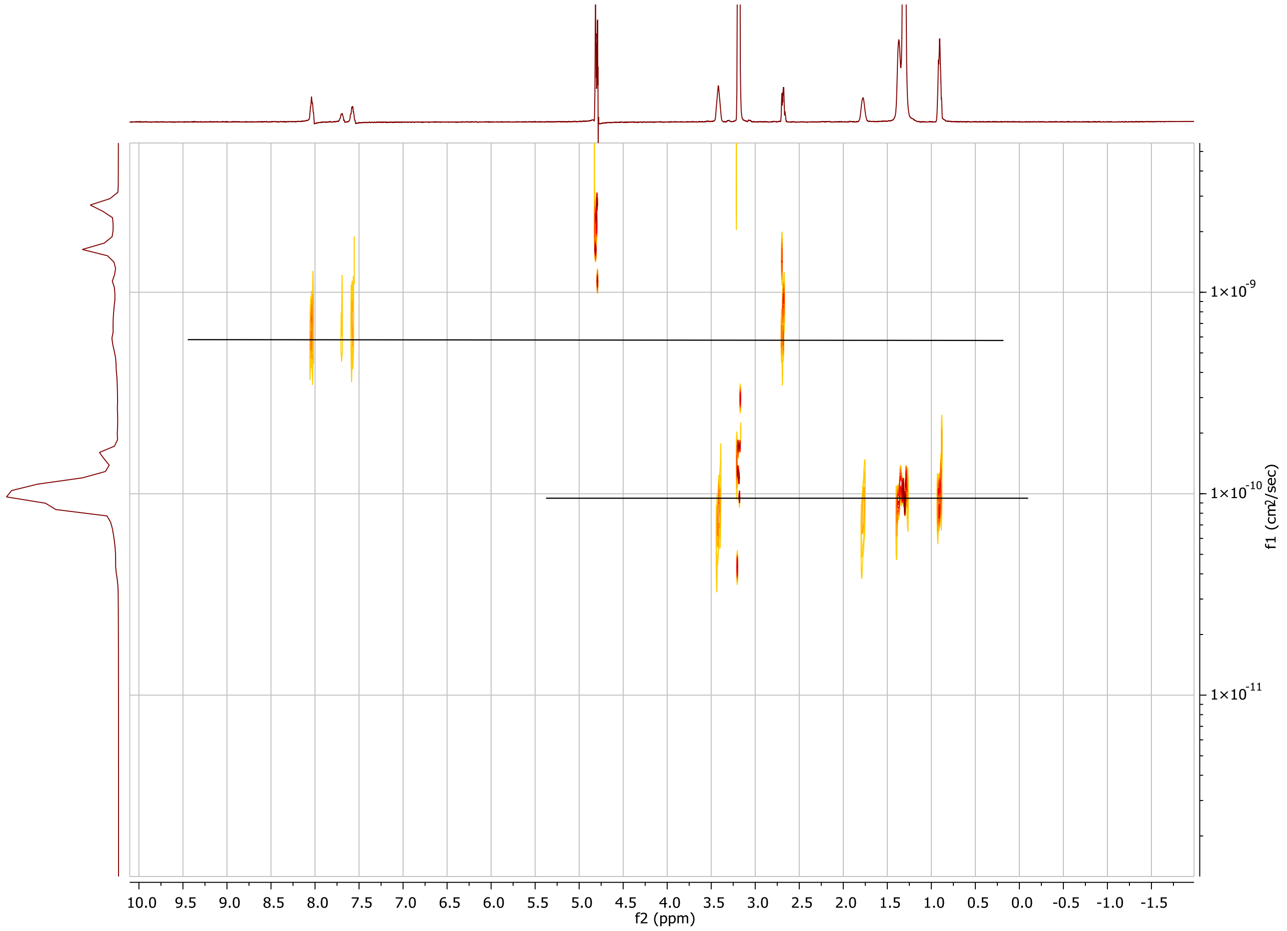

Figure S7 - Example of the DOSY plot for the Acetophenone (5 mM) in D_2O and in presence of CTAB (10 mM).

References

1. Coppola, L.; Gianferri, R.; Nicotera, I.; Oliviero, C.; Antonio Ranieri, G., Structural changes in CTAB/H2O mixtures using a rheological approach. *Phys. Chem. Chem. Phys.* **2004,** *6* (9).

2. Kumar, J.; Chawla, G.; Kumar, U.; Sahu, K., Design and syntheses of some new quinoxaline derivatives containing pyrazoline residue as potential antimicrobial agents. *Med. Chem. Res.* **2014,** *23* (9), 3929-3940.

3. Hall, M. J.; McDonnell, S. O.; Killoran, J.; O'Shea, D. F., A modular synthesis of unsymmetrical tetraarylazadipyrromethenes. *J. Org. Chem.* **2005,** *70* (14), 5571-8.

4. Tamuly, C.; Saikia, I.; Hazarika, M.; Bordoloi, M.; Hussain, N.; Das, M. R.; Deka, K., Bio-derived ZnO nanoflower: a highly efficient catalyst for the synthesis of chalcone derivatives. *RSC Adv.* **2015,** *5* (12), 8604-8608.

5. Umesha, B.; Basavaraju, Y. B.; Mahendra, C., Synthesis and biological screening of pyrazole moiety containing analogs of podophyllotoxin. *Med. Chem. Res.* **2014,** *24* (1), 142-151.

6. Tiecco, M.; Germani, R.; Cardellini, F., Carbon–carbon bond formation in acid deep eutectic solvent: chalcones synthesis via Claisen–Schmidt reaction. *RSC Adv.* **2016,** *6* (49), 43740-43747.

7. Hasan, A.; Khan, K. M.; Sher, M.; Maharvi, G. M.; Nawaz, S. A.; Choudhary, M. I.; Atta ur, R.; Supuran, C. T., Synthesis and inhibitory potential towards acetylcholinesterase, butyrylcholinesterase and lipoxygenase of some variably substituted chalcones. *J. Enzyme Inhib. Med. Chem.* **2005,** *20* (1), 41-7.

8. Kumar, D.; Suresh; Sandhu, J. S., An efficient green protocol for the synthesis of chalcones by a Claisen–Schmidt reaction using bismuth(III)chloride as a catalyst under solvent-free condition. *Green Chem. Lett. Rev.* **2010,** *3* (4), 283-286.

9. Sashidhara, K. V.; Rosaiah, J. N.; Kumar, A., Iodine-Catalyzed Mild and Efficient Method for the Synthesis of Chalcones. *Synth. Commun.* **2009,** *39* (13), 2288-2296.

10. Kachadourian, R.; Day, B. J.; Pugazhenti, S.; Franklin, C. C.; Genoux-Bastide, E.; Mahaffey, G.; Gauthier, C.; Di Pietro, A.; Boumendjel, A., A synthetic chalcone as a potent inducer of glutathione biosynthesis. *J. Med. Chem.* **2012,** *55* (3), 1382-8.
11. Gorman, A.; Killoran, J.; O'Shea, C.; Kenna, T.; Gallagher, W. M.; O'Shea, D. F., In vitro demonstration of the heavy-atom effect for photodynamic therapy. *J. Am. Chem. Soc.* **2004,** *126* (34), 10619-31.
12. Stroba, A.; Schaeffer, F.; Hindie, V.; Lopez-Garcia, L.; Adrian, I.; Frohner, W.; Hartmann, R. W.; Biondi, R. M.; Engel, M., 3,5-Diphenylpent-2-enoic acids as allosteric activators of the protein kinase PDK1: structure-activity relationships and thermodynamic characterization of binding as paradigms for PIF-binding pocket-targeting compounds. *J. Med. Chem.* **2009,** *52* (15), 4683-93.
13. Yadav, H. L.; Gupta, P.; Pawar, R. S.; Singour, P. K.; Patil, U. K., Synthesis and biological evaluation of anti-inflammatory activity of 1,3 diphenyl propenone derivatives. *Med. Chem. Res.* **2010,** *20* (4), 461-465.
14. Zhao, Y.; Song, Q., Copper-catalyzed tandem A3-coupling–isomerization–hydrolysis reactions of aldehydes and terminal alkynes leading to chalcones. *Org. Chem. Front.* **2016,** *3* (3), 294-297.
15. Soĺăniová, E.; Toma, Š.; Gronowitz, S., Investigation of substituent effects of chalcones by 13C n.m.r. spectroscopy. *Org. Magn. Res.* **2005,** *8* (9), 439-443.
16. Chaphekar, S. S.; Samant, S. D., Novel gel-entrapped base catalysts for the Claisen-Schmidt reaction. *J. Chem. Technol. Biotechnol.* **2004,** *79* (7), 769-773.
17. Maejima, T.; Shimoda, Y.; Nozaki, K.; Mori, S.; Sawama, Y.; Monguchi, Y.; Sajiki, H., One-pot aromatic amination based on carbon–nitrogen coupling reaction between aryl halides and azido compounds. *Tetrahedron* **2012,** *68* (6), 1712-1722.
18. Davey, W.; Gwilt, J. R., 196. Chalcones and related compounds. Part I. Preparation of nitro-, amino-, and halogeno-chalcones. *J. Chem. Soc.* **1957**.
19. Fiorilli, S.; Onida, B.; Barolo, C.; Viscardi, G.; Brunel, D.; Garrone, E., Tethering of modified Reichardt's dye on SBA-15 mesoporous silica: the effect of the linker flexibility. *Langmuir* **2007,** *23* (4), 2261-8.
20. Weber, W. M.; Hunsaker, L. A.; Abcouwer, S. F.; Deck, L. M.; Vander Jagt, D. L., Anti-oxidant activities of curcumin and related enones. *Bioorg. Med. Chem.* **2005,** *13* (11), 3811-20.
21. Kazi, I.; Guha, S.; Sekar, G., CBr(4) as a Halogen Bond Donor Catalyst for the Selective Activation of Benzaldehydes to Synthesize alpha,beta-Unsaturated Ketones. *Org. Lett.* **2017,** *19* (5), 1244-1247.
22. Yang, X.-Y.; Jia, Y.-X.; Tay, W. S.; Li, Y.; Pullarkat, S. A.; Leung, P.-H., Mechanistic insights into the role of PC- and PCP-type palladium catalysts in asymmetric hydrophosphination of activated alkenes incorporating potential coordinating heteroatoms. *Dalton Trans.* **2016,** *45* (34), 13449-13455.
23. Yang, X. Y.; Jia, Y. X.; Tay, W. S.; Li, Y.; Pullarkat, S. A.; Leung, P. H., Mechanistic insights into the role of PC- and PCP-type palladium catalysts in asymmetric hydrophosphination of activated alkenes incorporating potential coordinating heteroatoms. *Dalton Trans.* **2016,** *45* (34), 13449-55.
24. Dalla Via, L.; Gia, O.; Chiarelotto, G.; Ferlin, M. G., DNA-targeting pyrroloquinoline-linked butenone and chalcones: synthesis and biological evaluation. *Eur. J. Med. Chem.* **2009,** *44* (7), 2854-61.
25. Kurpil, B.; Otte, K.; Mishchenko, A.; Lamagni, P.; Lipinski, W.; Lock, N.; Antonietti, M.; Savateev, A., Carbon nitride photocatalyzes regioselective aminium radical addition to the carbonyl bond and yields N-fused pyrroles. *Nat. Commun.* **2019,** *10* (1), 945.
26. Liu, S.; Liebeskind, L. S., A simple, modular synthesis of substituted pyridines. *J. Am. Chem. Soc.* **2008,** *130* (22), 6918-9.
27. Shibata, K.; Katsuyama, I.; Matsui, M.; Muramatsu, H., Synthesis of 3-cyano-2-methylpyridines substituted with heteroaromatics. *J., Heterocycl. Chem.* **2009,** *28* (1), 161-165.
28. Rajasekaran, H.; Jerome, P.; Eliseenkov, E. V.; Boyarskiy, V. P.; Bhuvanesh, N.; Karvembu, R., Half-sandwich Ru(II)-thioamide complexes as catalysts for one pot synthesis of aromatic 1,5-diketones. *J. Organomet. Chem.* **2022,** *965-966*.
29. Rao, H. S. P.; Jothilingam, S., Solvent-free microwave-mediated Michael addition reactions. *J. Chem. Sci.* **2005,** *117* (4), 323-328.
30. Waldmann, H.; Karunakar, G. V.; Kumar, K., Gold(III)-mediated aldol condensations provide efficient access to nitrogen heterocycles. *Org. Lett.* **2008,** *10* (11), 2159-62.
31. Li, Z.; Wen, G.; He, L.; Li, J.; Jia, X.; Yang, J., Copper-catalyzed synthesis of 1,3,5-triarylpentane-1,5-diones from α,β-unsaturated ketones. *RSC Adv.* **2015,** *5* (64), 52121-52125.

32. Yin, Z.; Xiong, C.; Guo, J.; Hu, X.; Shan, Z.; Borovkov, V., Highly Chemoselective Solvent-Free Synthesis of 1,3,5-Triaryl-1,5-diketones: Crystallographic Investigation and Intramolecular Weak Bifurcated H Bonds Involving Aliphatic C–H Group. *Synlett* **2019,** *30* (19), 2143-2147.
33. Asressu, K. H.; Chan, C. K.; Wang, C. C., One-Pot Synthesis of 1,5-Diketones under a Transition-Metal-Free Condition: Application in the Synthesis of 2,4,6-Triaryl Pyridine Derivatives. *ACS Omega* **2021,** *6* (11), 7296-7311.
34. Sodhi, R. K.; Paul, S.; Gupta, V. K.; Kant, R., Conversion of α,β-unsaturated ketones to 1,5-diones via tandem retro-Aldol and Michael addition using Co(acac)2 covalently anchored onto amine functionalized silica. *Tetrahedron Lett.* **2015,** *56* (15), 1944-1948.
35. Yanagisawa, A.; Takahashi, H.; Arai, T., 1,5-Diketone Synthesis Promoted by Barium Hydride or Barium Alkoxides. *Synlett* **2006,** *2006* (17), 2833-2835.
36. Clennan, E. L.; Liao, C.; Ayokosok, E., Pyrylogens: synthesis, structural, electrochemical, and photophysical characterization of a new class of electron transfer sensitizers. *J. Am. Chem. Soc.* **2008,** *130* (24), 7552-3.
37. Wachter-Jurcsak, N.; Radu, C.; Redin, K., Addressing the unusual reactivity of 2-pyridinecarboxaldehyde and 2-quinolinecarboxaldehyde in base-catalyzed aldol reactions with acetophenone. *Tetrahedron Lett.* **1998,** *39* (23), 3903-3906.
38. Clennan, E. L.; Warrier, A. K. S., Syntheses and Properties of the New Electron Transfer Sensitizers 4,2′-Pyrylogens. *Organic Letters* **2008,** *11* (3), 685-688.
39. Xianqiang, H.; Qiu, F.; Yanliang, S.; Qingpeng, H.; Yong, W., NaOH-Al2O3 Catalyzed Synthesis of 1, 3, 5-triarylpentane-1, 5-diones Derivatives Under Solvent-Free Condition and Crystal Structure of 3-(2', 4'-dichlorophenyl)-1, 5-diphenylpentane-1, 5-dione. *Lett. Org. Chem.* **2012,** *9* (4), 280-286.
40. Romanelli, G.; Pasquale, G.; Sathicq, Á.; Thomas, H.; Autino, J.; Vázquez, P., Synthesis of chalcones catalyzed by aminopropylated silica sol–gel under solvent-free conditions. *J. Mol. Catal. A: Chem.* **2011,** *340* (1-2), 24-32.
41. Schmink, J. R.; Holcomb, J. L.; Leadbeater, N. E., Testing the validity of microwave-interfaced, in situ Raman spectroscopy as a tool for kinetic studies. *Org. Lett.* **2009,** *11* (2), 365-8.
42. Ma, Z.; Zhang, X.; Wang, H.; Han, Z.; Zheng, X.; Lin, J., Syntheses, structures and catalytic activity for Friedel-Crafts reactions of substituted indenyl rhenium carbonyl complexes. *J. Coord. Chem.* **2017,** *70* (4), 709-721.
43. Le Bras, G.; Provot, O.; Peyrat, J.-F.; Alami, M.; Brion, J.-D., Rapid microwave assisted hydration of internal arylalkynes in the presence of PTSA: an efficient regioselective access to carbonyl compounds. *Tetrahedron Lett.* **2006,** *47* (31), 5497-5501.
44. Maiti, G.; Karmakar, R.; Bhattacharya, R. N.; Kayal, U., A novel one pot route to flavones under dual catalysis, an organo- and a Lewis acid catalyst. *Tetrahedron Lett.* **2011,** *52* (43), 5610-5612.
45. Zhang, M. J.; Li, H. X.; Young, D. J.; Li, H. Y.; Lang, J. P., Reaction condition controlled nickel(ii)-catalyzed C-C cross-coupling of alcohols. *Org. Biomol. Chem.* **2019,** *17* (14), 3567-3574.
46. Minatti, A.; Zheng, X.; Buchwald, S. L., Synthesis of chiral 3-substituted indanones via an enantioselective reductive-Heck reaction. *J. Org. Chem.* **2007,** *72* (24), 9253-8.
47. Kumar, N.; Drabu, S.; Shalini, K., Synthesis and pharmacological screening of 4, 6-substituted di-(phenyl) pyrimidin-2-amines. *Arabian J. Chem.* **2017,** *10*, S877-S880.
48. Rajkiewicz, A. A.; Kalek, M., N-Heterocyclic Carbene-Catalyzed Olefination of Aldehydes with Vinyliodonium Salts To Generate alpha,beta-Unsaturated Ketones. *Org. Lett.* **2018,** *20* (7), 1906-1909.
49. Wu, X. F.; Neumann, H.; Spannenberg, A.; Schulz, T.; Jiao, H.; Beller, M., Development of a general palladium-catalyzed carbonylative Heck reaction of aryl halides. *J. Am. Chem. Soc.* **2010,** *132* (41), 14596-602.
50. Agarwal, A.; Srivastava, K.; Puri, S. K.; Chauhan, P. M., Synthesis of 4-pyrido-6-aryl-2-substituted amino pyrimidines as a new class of antimalarial agents. *Bioorg. Med. Chem.* **2005,** *13* (22), 6226-32.
51. Mubofu, E. B.; Engberts, J. B. F. N., Specific acid catalysis and Lewis acid catalysis of Diels–Alder reactions in aqueous media. *J. Phys. Org. Chem.* **2004,** *17* (3), 180-186.
52. Ciupa, A.; Mahon, M. F.; De Bank, P. A.; Caggiano, L., Simple pyrazoline and pyrazole "turn on" fluorescent sensors selective for Cd2+ and Zn2+ in MeCN. *Org. Biomol. Chem.* **2012,** *10* (44), 8753-7.

53. Parveen, H.; Iqbal, P. F.; Azam, A., Synthesis and Characterization of a New Series of Hydroxy Pyrazolines. *Synth. Commun.* **2008,** *38* (22), 3973-3983.
54. Liu, W.; Shi, H. M.; Jin, H.; Zhao, H. Y.; Zhou, G. P.; Wen, F.; Yu, Z. Y.; Hou, T. P., Design, synthesis and antifungal activity of a series of novel analogs based on diphenyl ketones. *Chem. Biol. Drug Des.* **2009,** *73* (6), 661-7.
55. Budhiraja, A.; Kadian, K.; Kaur, M.; Aggarwal, V.; Garg, A.; Sapra, S.; Nepali, K.; Suri, O. P.; Dhar, K. L., Synthesis and biological evaluation of naphthalene, furan and pyrrole based chalcones as cytotoxic and antimicrobial agents. *Med. Chem. Res.* **2011,** *21* (9), 2133-2140.
56. Paul, N.; Shanmugam, M. J.; Muthusubramanian, S., Facile Microwave-Assisted Michael Addition of Diphenacyl Sulfides to Chalcones Under Solvent-Free Conditions: Generation of Symmetrical and Unsymmetrical 1,5-Diketones. *Synth. Commun.* **2012,** *43* (1), 129-138.
57. Cave, G. W. V.; Raston, C. L., Efficient synthesis of pyridines via a sequential solventless aldol condensation and Michael addition. *J. Chem. Soc., Perkin Trans. 1* **2001,** (24), 3258-3264.
58. Harrison, D. P.; Lapides, A. M.; Binstead, R. A.; Concepcion, J. J.; Mendez, M. A.; Torelli, D. A.; Templeton, J. L.; Meyer, T. J., Coordination chemistry of single-site catalyst precursors in reductively electropolymerized vinylbipyridine films. *Inorg. Chem.* **2013,** *52* (9), 4747-9.
59. Mondal, P. C.; Manna, A. K., Synthesis of heteroleptic terpyridyl complexes of Fe(ii) and Ru(ii): optical and electrochemical studies. *New J. Chem.* **2016,** *40* (7), 5775-5781.
60. James, L.; Maguire, G. E. M.; Martincigh, B. S.; McKee, V.; Ndlovu, N., 3-Phenyl-1,5-di-2-pyridylpentane-1,5-dione. *Acta Crystallographica Section E Structure Reports Online* **2006,** *63* (1), o153-o155.
61. Laroche, B.; Saito, Y.; Ishitani, H.; Kobayashi, S., Basic Anion-Exchange Resin-Catalyzed Aldol Condensation of Aromatic Ketones with Aldehydes in Continuous Flow. *Org. Process Res. Dev.* **2019,** *23* (5), 961-967.
62. Katritzky, A. R., Heterocyclic analogs of 2,4,6-triphenylpyrylium and 1-benzyl-2,4,6-triphenylpyridinium. *J. Chem, Res. Synopses* **1980,** (9), 312-13.
63. Kumari, P.; Poonam; Chauhan, S. M., Efficient cobalt(II) phthalocyanine-catalyzed reduction of flavones with sodium borohydride. *Chem. Commun.* **2009,** (42), 6397-9.
64. Zhou, B.; Jiang, P.; Lu, J.; Xing, C., Characterization of the Fluorescence Properties of 4-Dialkylaminochalcones and Investigation of the Cytotoxic Mechanism of Chalcones. *Arch. Pharm.* **2016,** *349* (7), 539-552.
65. Li, R.; Yan, L.; Wang, Z.; Qi, Z., An aggregation-induced emissive NIR luminescent based on ESIPT and TICT mechanisms and its application to the detection of Cys. *J. Mol. Struct.* **2017,** *1136*, 1-6.
66. Sukumaran, S. D.; Chee, C. F.; Viswanathan, G.; Buckle, M. J.; Othman, R.; Abd Rahman, N.; Chung, L. Y., Synthesis, Biological Evaluation and Molecular Modelling of 2'-Hydroxychalcones as Acetylcholinesterase Inhibitors. *Molecules* **2016,** *21* (7).
67. Tang, Q.; Xiao, W.; Huang, C.; Si, W.; Shao, J.; Huang, W.; Chen, P.; Zhang, Q.; Dong, X., pH-Triggered and Enhanced Simultaneous Photodynamic and Photothermal Therapy Guided by Photoacoustic and Photothermal Imaging. *Chem. Mater.* **2017,** *29* (12), 5216-5224.
68. Vaidya, S. S.; Vinaya, H.; Mahajan, S. S., Microwave-assisted synthesis, pharmacological evaluation, and QSAR studies of 1,3-diaryl-2-propen-1-ones. *Med. Chem. Res.* **2012,** *21* (12), 4311-4323.
69. Raj, A. R. N.; Hussaini, S. S.; Huq, C. A. M. A.; Babu, A. R. S., Solvent-free microwave-expedited synthesis of ferrocenylenones and ferrocenyl-1,5-diketones using hydrotalcite. *J. Indian Chem. Soc.* **2009,** *86* (11), 1218-1223.
70. Cardona, R. A.; Hernandez, K.; Pedro, L. E.; Otano, M. R.; Montes, I.; Guadalupe, A. R., Electrochemical and Spectroscopical Characterization of Ferrocenyl Chalcones. *J. Electrochem. Soc.* **2010,** *157* (8), F104.
71. de Vasconcelos, A.; Campos, V. F.; Nedel, F.; Seixas, F. K.; Dellagostin, O. A.; Smith, K. R.; de Pereira, C. M.; Stefanello, F. M.; Collares, T.; Barschak, A. G., Cytotoxic and apoptotic effects of chalcone derivatives of 2-acetyl thiophene on human colon adenocarcinoma cells. *Cell Biochem. Funct.* **2013,** *31* (4), 289-297.
72. Rasheed, L.; Hasan, A., Synthesis of some benzalacetophenones and their imino derivatives. *Rasheed, Lubna; Hasan, Aurangzeb* **2007,** *19* (7), 5057-5067.

73. Roman, G., Critical assessment of two classical synthetic methods for preparation of thiophene-substituted isoxazoles. *Res. Chem. Intermed.* **2013,** *40* (5), 2039-2057.
74. Budak, Y.; Ceylan, M., Synthesis and Characterization of α-Bromo Chalcone Derivatives. *Chin. J. Chem.* **2009,** *27* (8), 1575-1581.
75. Shih, T.-L.; Liu, M.-H.; Li, C.-W.; Kuo, C.-F., Halo-substituted chalcones and azachalcones inhibited lipopolysaccharited-stimulated pro-inflammatory responses through the TLR4-mediated pathway. *Molecules* **2018,** *23* (3), 597.
76. Fan, C.; Wang, X.; Ding, P.; Wang, J.; Liang, Z.; Tao, X., Synthesis, photophysical and iron-sensing properties of terpyridyl-based triphenylamine derivatives. *Dyes and Pigments* **2012,** *95* (3), 757-767.
77. Zhang, B.; Duan, D.; Ge, C.; Yao, J.; Liu, Y.; Li, X.; Fang, J., Synthesis of xanthohumol analogues and discovery of potent thioredoxin reductase inhibitor as potential anticancer agent. *J. Med. Chem.* **2015,** *58* (4), 1795-1805.
78. Chiaradia, L. D.; dos Santos, R.; Vitor, C. E.; Vieira, A. A.; Leal, P. C.; Nunes, R. J.; Calixto, J. B.; Yunes, R. A., Synthesis and pharmacological activity of chalcones derived from 2,4,6-trimethoxyacetophenone in RAW 264.7 cells stimulated by LPS: quantitative structure-activity relationships. *Bioorg Med Chem* **2008,** *16* (2), 658-67.
79. Zhang, Y.; Wu, J.; Ying, S.; Chen, G.; Wu, B.; Xu, T.; Liu, Z.; Liu, X.; Huang, L.; Shan, X.; Dai, Y.; Liang, G., Discovery of new MD2 inhibitor from chalcone derivatives with anti-inflammatory effects in LPS-induced acute lung injury. *Sci. Rep.* **2016,** *6*, 25130.
80. Rocco, D.; Housecroft, C. E.; Constable, E. C., Synthesis of Terpyridines: Simple Reactions-What Could Possibly Go Wrong? *Molecules* **2019,** *24* (9), 1799.
81. Vatsadze, S. Z.; Nuriev, V. N.; Leshcheva, I. F.; Zyk, N. V., New aspects of the aldol condensation of acetylpyridines with aromatic aldehydes. *Russ. Chem. Bull.* **2004,** *53* (4), 911-915.
82. Hall, M. J., Determination of Polysorbate 80 in Bakery Products and Frozen Desserts. *J. AOAC Int.* **1964,** *47* (4), 685-688.
83. Guthrie, J. P.; Cossar, J.; Cullimore, P. A.; Kamkar, N. M.; Taylor, K. F., The retroaldol reaction of chalcone. *Can. J. Chem.* **1983,** *61* (11), 2621-2626.
84. Perrin, C. L.; Chang, K. L., The Complete Mechanism of an Aldol Condensation. *J. Org. Chem.* **2016,** *81* (13), 5631-5.
85. Szutkowski, K.; Kolodziejska, Z.; Pietralik, Z.; Zhukov, I.; Skrzypczak, A.; Materna, K.; Kozak, M., Clear distinction between CAC and CMC revealed by high-resolution NMR diffusometry for a series of bis-imidazolium gemini surfactants in aqueous solutions. *RSC Adv.* **2018,** *8* (67), 38470-38482.
86. Persson, B. O.; Drakenberg, T.; Lindman, B., Carbon-13 NMR of micellar solutions. Micellar aggregation number from the concentration dependence of the carbon-13 chemical shifts. *J. Phys. Chem.* **2002,** *83* (23), 3011-3015.
87. Shimizu, S.; Pires, P. A. R.; Fish, H.; Halstead, T. K.; El Seoud, O. A., Proton and carbon-13 NMR study of the aggregation of benzyl(2-acylaminoethyl)dimethylammonium chloride surfactants in D2O. *Phys. Chem. Chem. Phys.* **2003,** *5* (16), 3489-3497.
88. Sabatino, P.; Szczygiel, A.; Sinnaeve, D.; Hakimhashemi, M.; Saveyn, H.; Martins, J. C.; Van der Meeren, P., NMR study of the influence of pH on phenol sorption in cationic CTAB micellar solutions. *Colloids Surf., A* **2010,** *370* (1-3), 42-48.
89. de Campo, L.; Yaghmur, A.; Garti, N.; Leser, M. E.; Folmer, B.; Glatter, O., Five-component food-grade microemulsions: structural characterization by SANS. *J Colloid Interface Sci* **2004,** *274* (1), 251-67.
90. Tummino, P. J.; Gafni, A., Determination of the aggregation number of detergent micelles using steady-state fluorescence quenching. *Biophys. J.* **1993,** *64* (5), 1580-7.
91. Mahmood, M. E.; Al-Koofee, D. A. F., Effect of Temperature Changes on Critical Micelle Concentration for Tween Series Surfactant. *Global J. Sci. Front. Res. Chem.* **2013,** *13*, 1-7.
92. Bertrán, J. F.; Rodríguez, M., Study of the intrinsic and conformational solvent effects on the chemical shift of the aldehydic proton in furfural and thiophenealdehyde. *Org. Magn. Res.* **2005,** *6* (10), 525-527.
